# Supplementary material for: Drp1 splice variants regulate ovarian cancer mitochondrial dynamics and tumor progression
Source: EMBO Rep. 2024 Aug 27;25(10):16. doi: 10.1038/s44319-024-00232-4 (PMC11467262; doi:10.1038/s44319-024-00232-4)
Supplement: Supplementary file 1 — Appendix [file 44319_2024_232_MOESM1_ESM.pdf]

**Appendix**

**Table of Content:**

**Appendix Figure S1:** Identification of Drp1 transcript variants in ovarian cancer cell lines by rapid amplification of cDNA ends (RACE)……………………………………………………………….………Page 2

**Appendix Figure S2:** Expression of *DNM1L* splice variants in matched patient specimens…….……...Page 4

**Appendix Figure S3:** TEM images of OVCA433 expressing Drp1(-17), Drp1(16/17) or GFP control…Page 5

**Appendix Figure S4:** Metabolite analysis of OVC433 cells expressing Drp1(-17), Drp1(16/17) or GFP control………………………………………………………………………………………………...Page 6

**Appendix Figure S5:** S616 phosphorylation of recombinant expressed Drp1 following cisplatin and paclitaxel treatment, as assessed by western blotting………….………………………………..………..Page 7

**Appendix Figure S6.** Tumor morphology and lymph node metastases after Cisplatin treatment……………………………………………………………………………………..………...Page 7

**Appendix Figure S7.** Splice variant specific siRNA mediated knock down demonstrates the presence of Drp1(16/17) and Drp1(-/17) protein variants in OVCA433 cells……………………………….………...Page 8

**Appendix Figure S8.** *DNM1L* gene copy number alterations and CDK12 mutation status relative to Drp1 splice variant expression from TCGA…………………………………………………………….………...Page 8

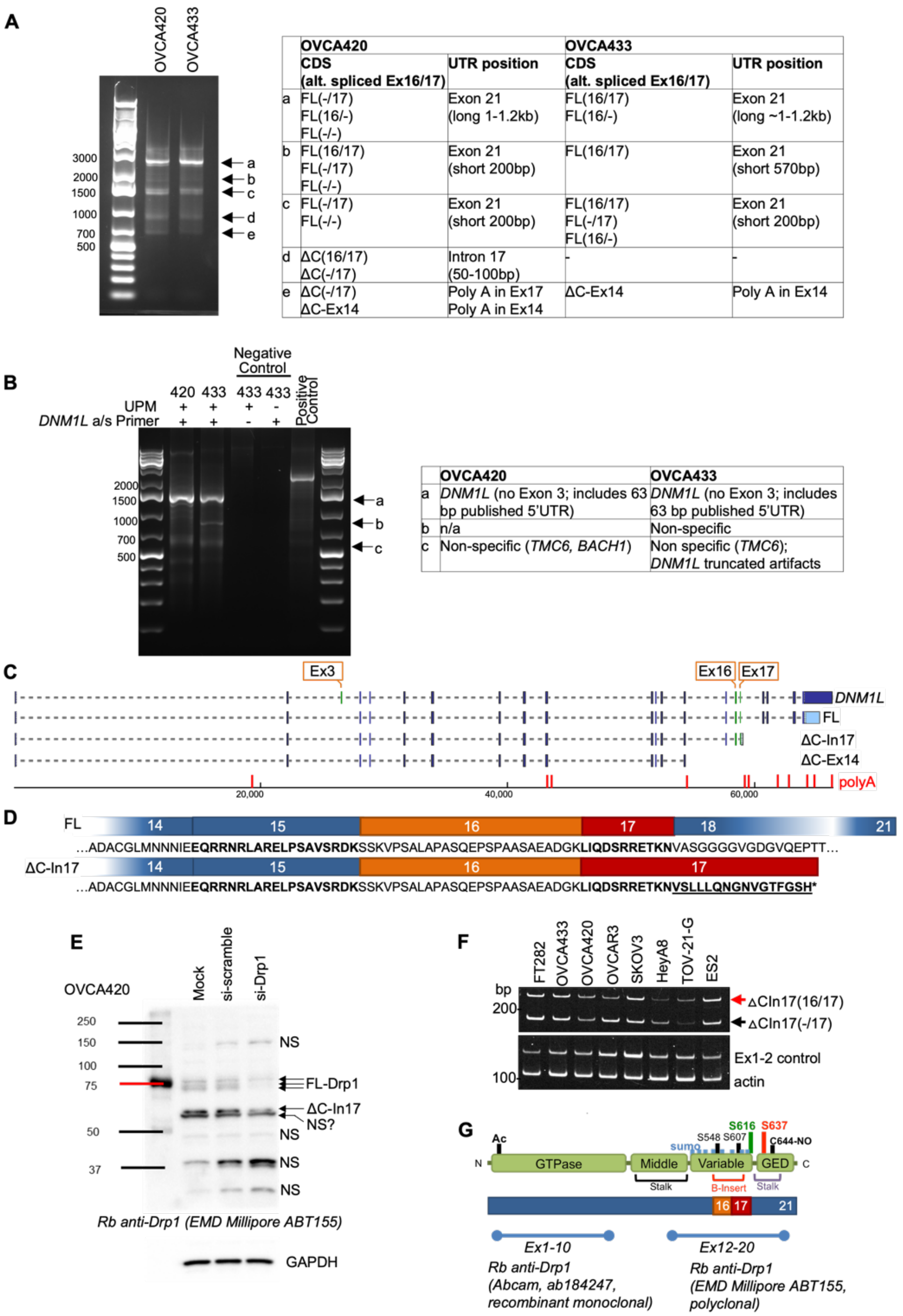


| | OVCA420 CDS (alt. spliced Ex16/17) | OVCA420 UTR position | OVCA433 CDS (alt. spliced Ex16/17) | OVCA433 UTR position |
|---|---|---|---|---|
| a | FL(-/17)<br>FL(16/-)<br>FL(-/-) | Exon 21<br>(long 1-1.2kb) | FL(16/17)<br>FL(16/-) | Exon 21<br>(long ~1-1.2kb) |
| b | FL(16/17)<br>FL(-/17)<br>FL(-/-) | Exon 21<br>(short 200bp) | FL(16/17) | Exon 21<br>(short 570bp) |
| c | FL(-/17)<br>FL(-/-) | Exon 21<br>(short 200bp) | FL(16/17)<br>FL(-/17)<br>FL(16/-) | Exon 21<br>(short 200bp) |
| d | ΔC(16/17)<br>ΔC(-/17) | Intron 17<br>(50-100bp) | - | - |
| e | ΔC(-/17)<br>ΔC-Ex14 | Poly A in Ex17<br>Poly A in Ex14 | ΔC-Ex14 | Poly A in Ex14 |

| | OVCA420 | OVCA433 |
|---|---|---|
| a | *DNM1L* (no Exon 3; includes 63 bp published 5'UTR) | *DNM1L* (no Exon 3; includes 63 bp published 5'UTR) |
| b | n/a | Non-specific |
| c | Non-specific (*TMC6, BACH1*) | Non specific (*TMC6*); *DNM1L* truncated artifacts |

**Appendix Figure S1. Identification of Drp1 transcript variants in ovarian cancer cell lines by rapid amplification of cDNA ends (RACE).**

A. Table shows details of the 3' RACE products derived from ovarian cancer cell lines OVCA420 and OVCA433, which represent multiple Drp1/*DNM1L* transcripts variants, including full length (FL) transcripts with alternatively spliced exons 16 and 17, and C terminal truncated transcripts at exon 14 (ΔC-Ex14) and intron 17 (ΔC-In17; blot from Figure 1C shown again to illustrate position of 3'RACE products). 3' RACE was carried out using SMARTer 3'5'RACE kit (Takara). PCR products from each cell line (a-e) were gel-extracted and cloned into the in-Fusion pRACE vector. 3-5 colonies per clone were selected for sequencing to determine the major5'RACE products in OVCA420 and OVCA433 cells (Table).
B. 5' RACE reveals that *DNM1L* transcripts expressed in OVCA420 and OVCA433 cell share the same 5'UTR and lack exon 3. 5' RACE was carried out using SMARTer 3'RACE kit (Takara) the with the Universal Primer A Mix (UPM) and the *DNM1L* specific antisense primer, positioned in Exon 12. PCR

products from each cell line (a-c) were gel-extracted and cloned into the in-Fusion pRACE vector. 3-5 colonies per clone were selected for sequencing to determine the major 5'RACE products in OVCA420 and OVCA433 cells (Table).

C. Transcript variants identified in OVCA420 and OVCA433 cells include alternative splicing of the variable domain exons 16 and 17; variable lengths of 3'UTRs, and utilization of proximal polyadenylation, resulting in two C terminal truncation variants, terminating in Intron 17 (ΔC-In17) and exon 14 (ΔC-Ex14; indicated in red; PolyA_DB v.3.2; PolyASites). 5'RACE demonstrates that ovarian cancer cell lines share the same 5'UTR and lack exon 3.
D. Schematic of the *DNM1L* variable domain Exons 16 and 17 alternatively spliced in ovarian cancer cells and corresponding amino acid sequences. The variant terminating in Intron 17 (ΔC-In17) also displays variable domain exon 16 alternative splicing and is predicted to encode an additional 16 amino acids from the adjacent intron to terminate at an alternate STOP codon.
E. Protein variants identified near the 75kDa molecular weight marker by western blotting (anti-Drp1 ABT155) are verified to be Drp1 using siRNA mediated knock-down (Dharmacon siRNA pool). Non-specific bands (NS) are not affected by siRNA targeting *DNM1L/Drp1*.
F. RT-PCR with primers designed to detect the intronic retention of the 3' region of ΔC-In17 illustrates that the *DNM1L* C terminal truncation variant terminating in Intron 17 can be detected in most ovarian cancer cell lines to variable degrees and that these transcripts can vary in their splicing of exon 16. Same samples were used for RT-PCR as those depicted in Figure 1F (loading control blot same as Fig. 1F).
G. Regions of Drp1 protein targeted by commercially available Drp1 antibodies.

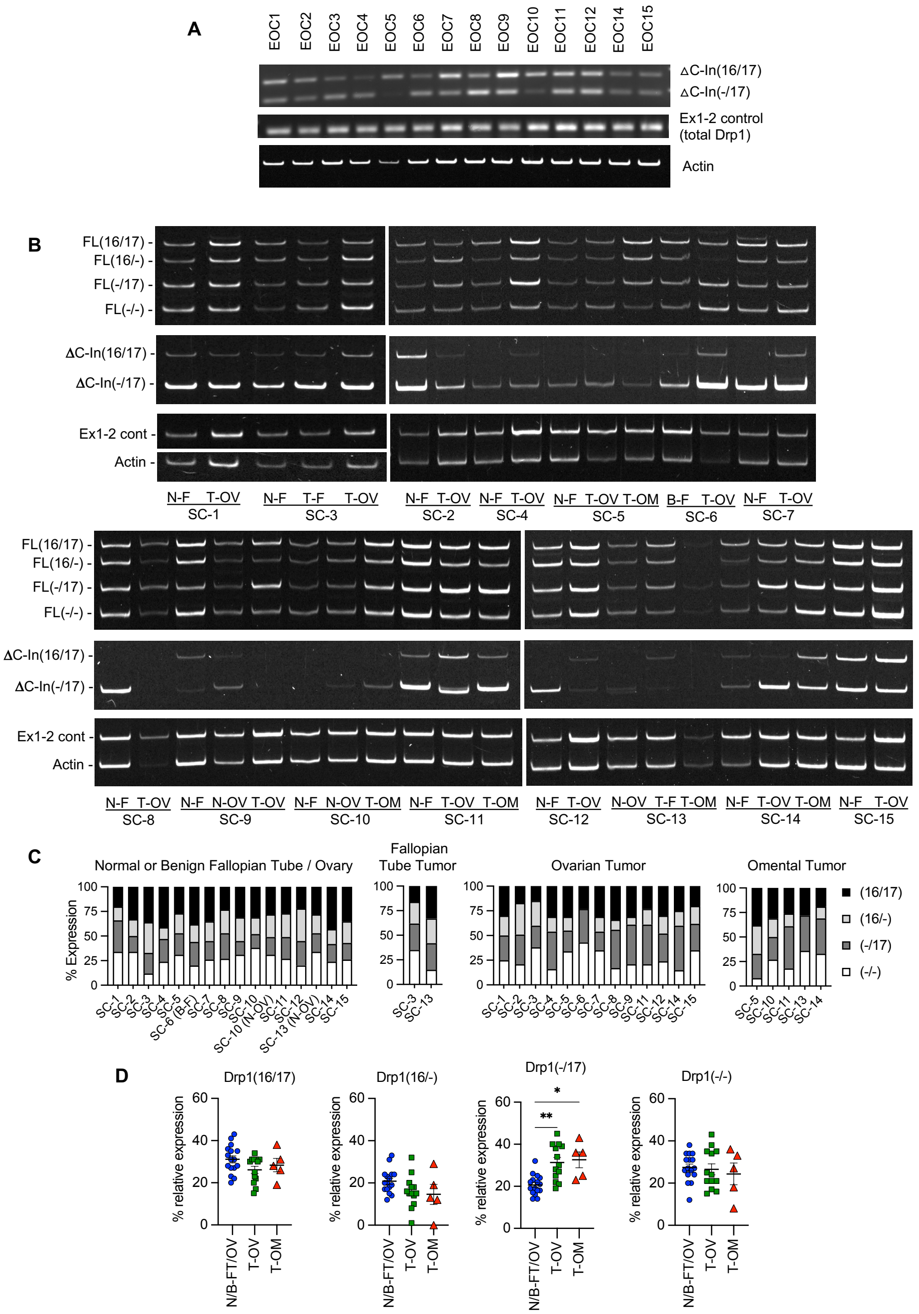


**Appendix Figure S2. Expression of *DNM1L* splice variants in matched patient specimens.**

A. RT-PCR was used to show relative expression of ∆C-In17(-/17) and ∆C-In17(16/17) truncated transcripts of DNM1L in a panel of patient ascites derived EOCs. (EOC9 & 11: carcinosarcoma; EOC 2,3,4 HGSA high grade serous adenocarcinoma; GI: gastrointestinal; tumor stage is indicated in Figure 2C. RT-PCR run in parallel with same samples as in 2C; thus actin loading same as in 2C).
B. RT-PCR of *DNM1L* variable domain splice variant expression from normal fallopian tube (N-F), and matched ovarian (T-OV) and omental tumors (T-OM). The relative expression of splice variant transcript Drp1(-/17) is consistently higher in ovarian tumor and omental tumor compared to matched normal fallopian tube specimens N=normal, T=tumor, B=benign, F=fallopian tube, OV= ovary, OM= omentum. All specimens were classified as HGSA, and the following stage: SC-1: IIIC, SC-2: IIIB, SC-3: IIIC, SC-4: IB, SC-5: IIIC, SC-6: IC3, SC-7: IIIB, SC-8: IIIC, SC-9: IVB, SC-10: IIIC, SC-11: IIIB, SC-12: IC2, SC-13: IIIC, SC-14: IIIC, SC-15: IIIB. (Sample SC-14 blot has been reproduced in Figure 1D as a representative example of these data).
C. Quantification of relative Drp1 variable domain splice variant expression from panel B.

D. Comparison of relative expression of full length variable domain variants between normal fallopian tube/ovary and ovarian tumor or omental tumor. Relative expression of Drp1(-/17) is significantly increased in ovarian tumors and omental tumors compared to matched normal tissues. (Mixed Effects Analysis with Tukey's post test. *p<0.05, **p<0.01; Same data depicted as relative ratios in Figure 2D).

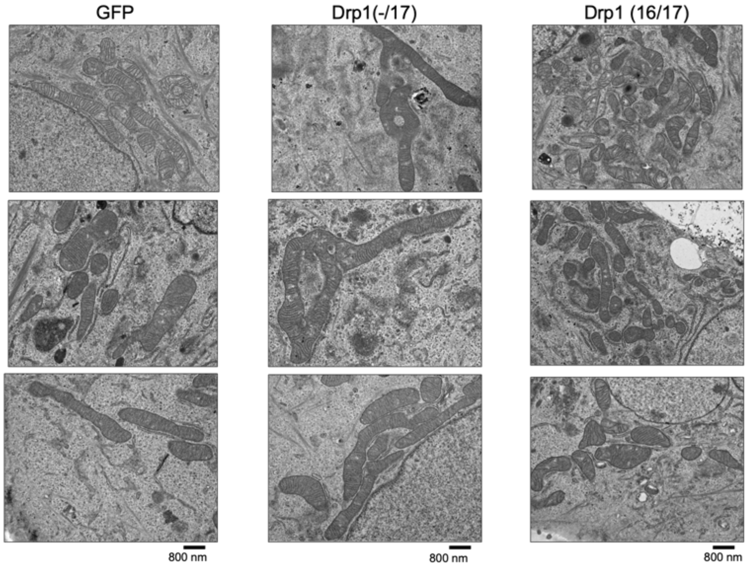


**Appendix Figure S3. TEM images of OVCA433 expressing Drp1(-17), Drp1(16/17) or GFP control.** Representative TEM images from 3 individual biological replicates demonstrate a more fused mitochondrial morphology in Drp1(-/17) cells compared to the smaller, fragmented mitochondria characteristic of Drp1(16/17) cells.

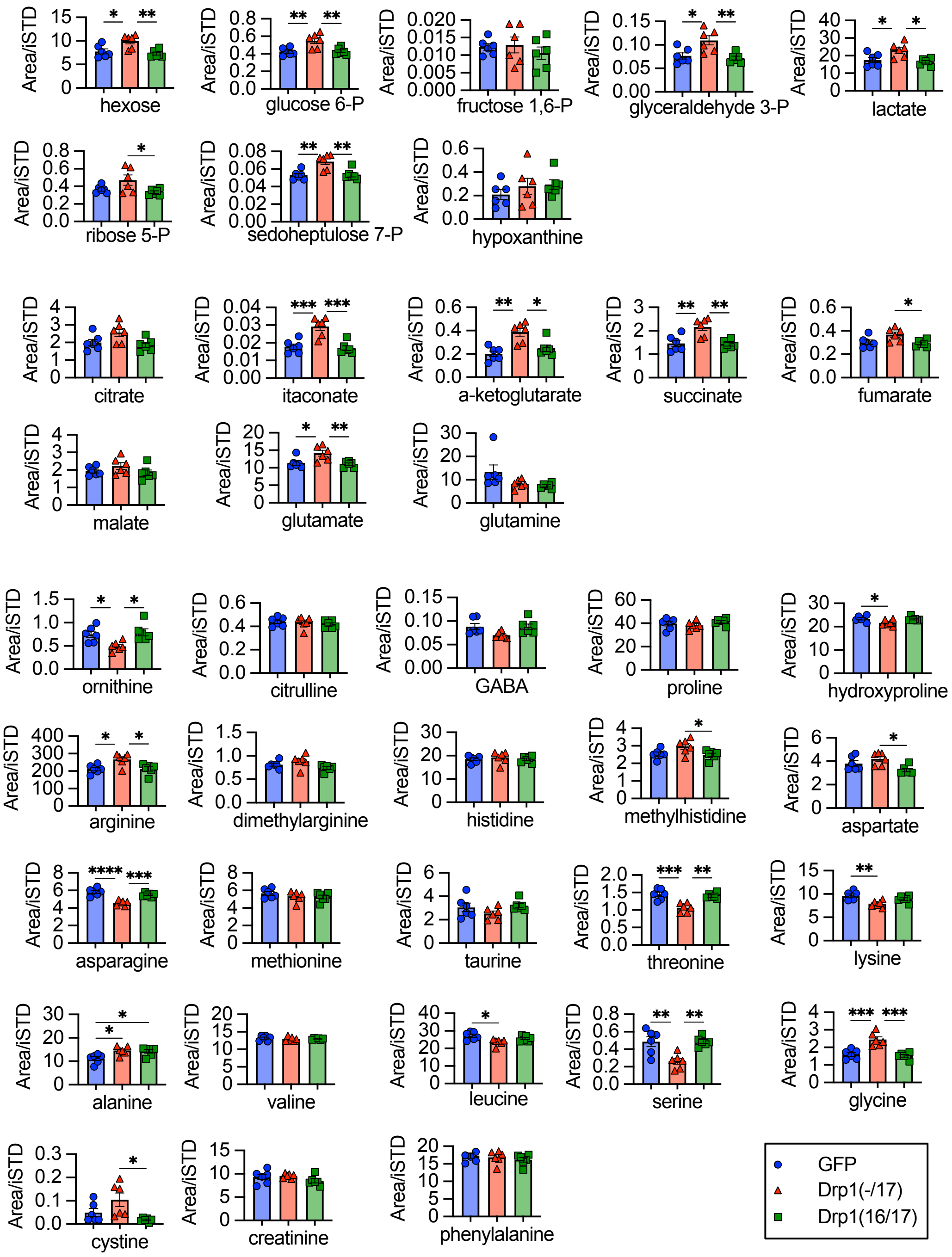


**Appendix Figure S4. Metabolite analysis of OVC433 cells expressing Drp1(-17), Drp1(16/17) or GFP control.** OVCA433 lysates were subjected to untargeted liquid chromatography high resolution mass spectrometry (LC-HRMS) analysis. Metabolite amounts are reported as a ratio of the analyte peak area/internal standard peak area (same data depicted in heatmap, Figure 4).

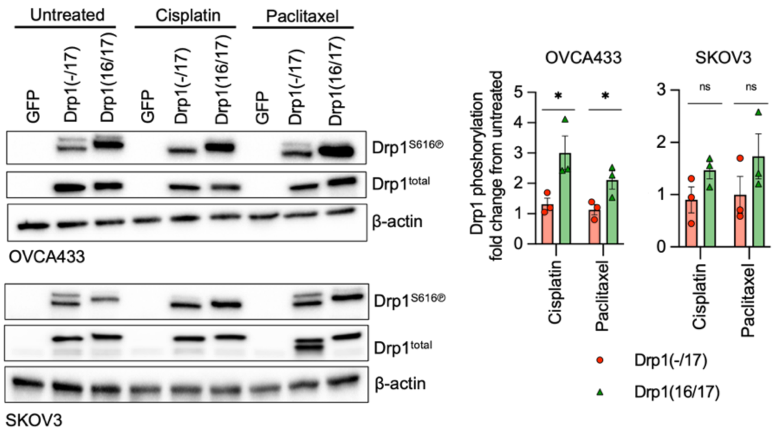


**Appendix Figure S5. S616 phosphorylation of recombinant expressed Drp1 following cisplatin and paclitaxel treatment, as assessed by western blotting.**

Changes in Drp1 phosphorylation (S616) in response to cisplatin (5 μM) and paclitaxel (1 nM) was assessed using western blotting (Densitometry quantification was carried out using ImageJ and normalizing to total Drp1, data are expressed relative to untreated control, n=3, unpaired t test *p<0.05).

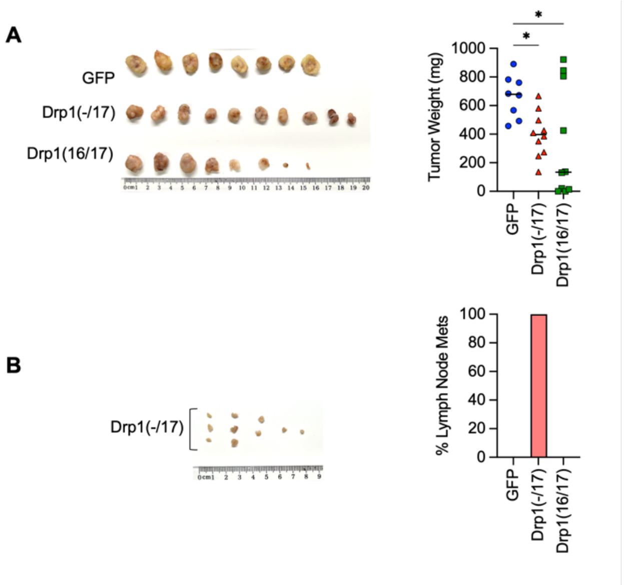


**Appendix Figure S6. Tumor morphology and lymph node metastases after Cisplatin treatment.**

A. Final tumor weight of SKOV3 subcutaneous tumors from cisplatin treated groups as mice reached endpoints; n=8, GFP; n=10, Drp1(-/17);n=10, Drp1(16/17); median shown, Kruskal-Wallis P=0.0377 uncorrected Dunn's test *P<0.05).
B. Mice injected with Drp1(-/17) expressing SKOV3 cells were the only group that develop lymph node metastases in the subcutaneous tumor model under cisplatin treatment. Lymph node metastases were resected as mice reached endpoints. Graph shows percentage of mice with lymph node metastases.

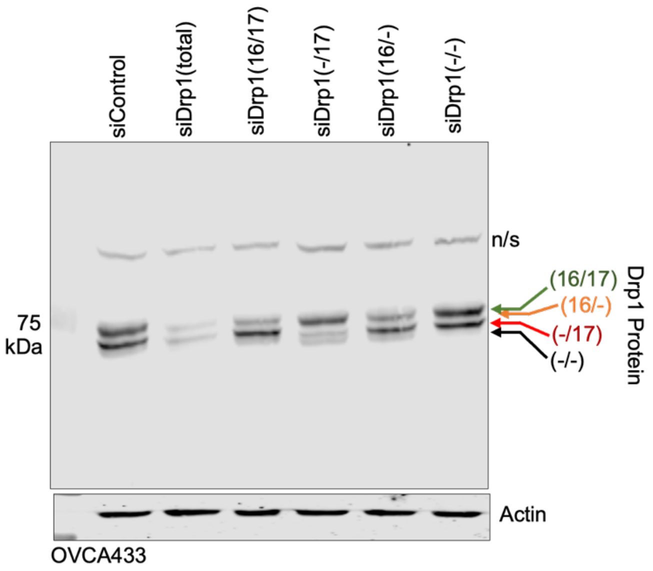


**Appendix Figure S7. Splice variant specific siRNA mediated knock down demonstrates the presence of Drp1(16/17) and Drp1(-/17) protein variants in OVCA433 cells.** OVCA433 cells were transfected with scramble non-targeting siRNA control or Drp1 splice variant specific siRNAs (see methods) and Drp1 protein expression analyzed 48 hours post-transfection by resolving proteins on 7.5% SDS-PAGE followed by western blotting using Drp1 antibody (ab184247).

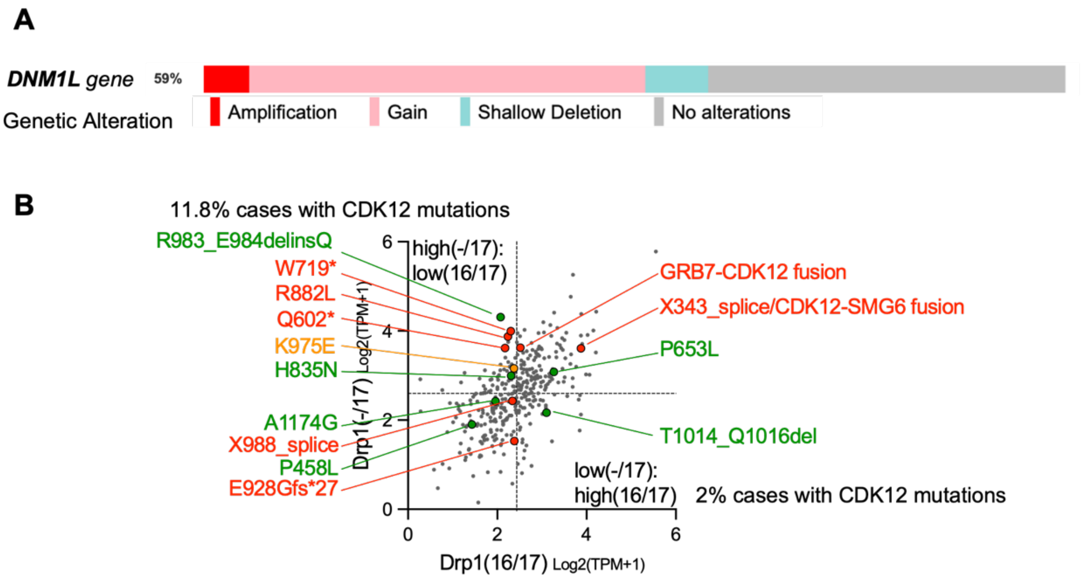


**Appendix Figure S8. *DNM1L* gene copy number alterations and CDK12 mutation status relative to Drp1 splice variant expression from TCGA.**

A. *DNM1L* gene copy number alterations (CNA) in 233 Ovarian Serous Cystadenocarcinoma cases with complete CNA data (TCGA, PanCancer Atlas). 5% of cases display high level amplification, 46% low level gain, and 7% shallow deletion (Data obtained from cbioportal).
B. Drp1(-/17) mRNAs splice variant expression relative to Drp1(16/17) in TCGA high grade serous ovarian cancer specimens is shown on XY plot (log2 TPM+1). Mutually exclusive high and low expression is based on median log2 TPM+1 expression cut offs indicated by dotted line. Specimens that have CDK12 mutations are highlighted (red: likely oncogenic; green: unknown consequence; orange: inconclusive; *= truncation; fs= frameshift). 11.8% of tumors displaying mutually exclusive high Drp1(-/17)/low Drp1(16/17) (n=52) expression display CDK12 mutations, while tumors with low Drp1(-/17)/high Drp1(16/17) (n=52) expression display only 2% CDK12 mutations (Data obtained from cbioportal).
