# Supplementary figures and images for: Drp1 splice variants regulate ovarian cancer mitochondrial dynamics and tumor progression
Source: EMBO Rep. 2024 Aug 27;25(10):16. doi: 10.1038/s44319-024-00232-4 (PMC11467262; doi:10.1038/s44319-024-00232-4)

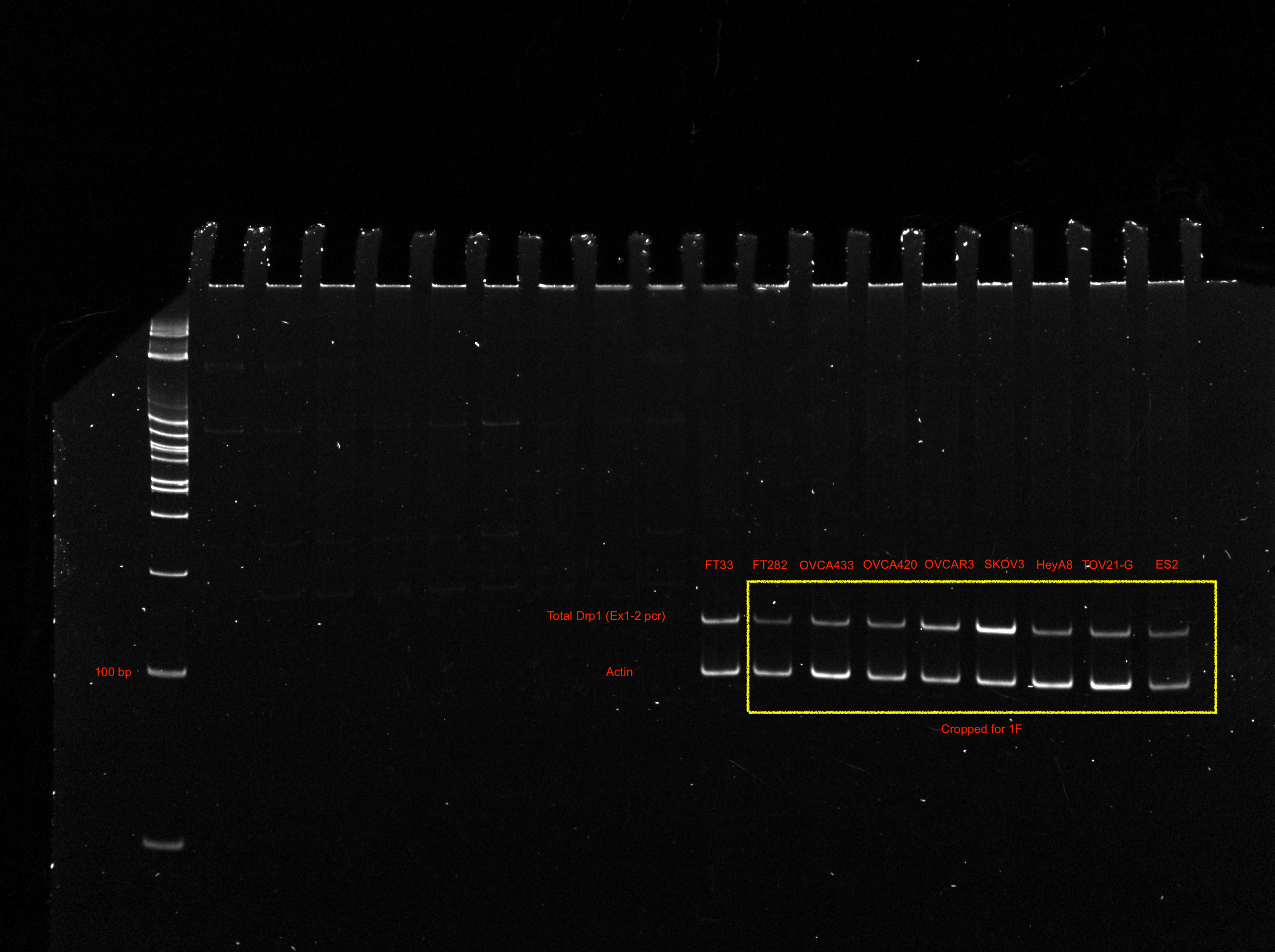

Supplement: Supplementary file 3 — Source data Fig. 1 [file 44319_2024_232_MOESM3_ESM.zip › Figure 1/1F/1F_OVCACelllines_TotalDrp1_Actin PCR.tif]

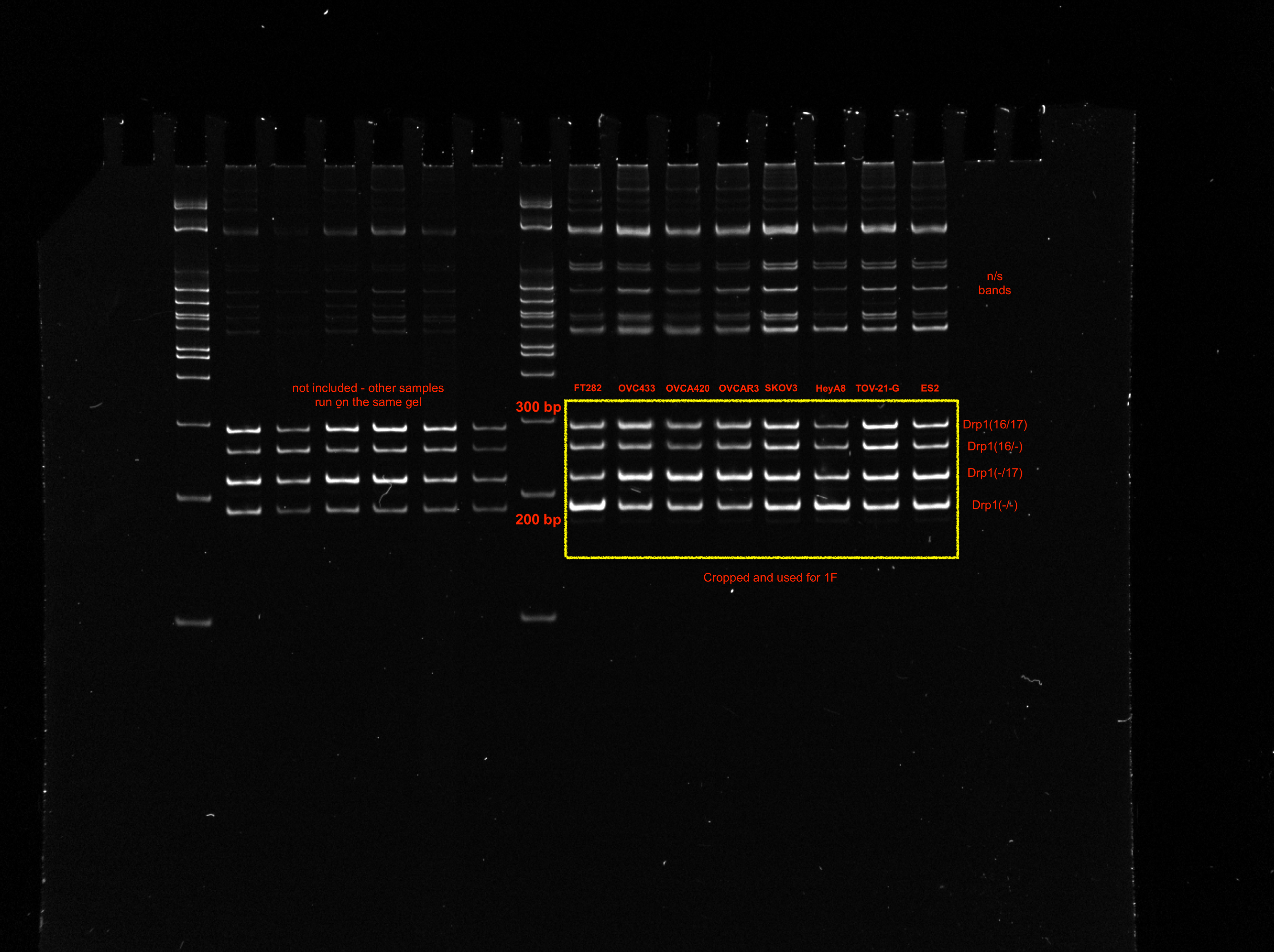

Supplement: Supplementary file 3 — Source data Fig. 1 [file 44319_2024_232_MOESM3_ESM.zip › Figure 1/1F/1F_OVCACelllines_Drp1Variant PCR.tif]

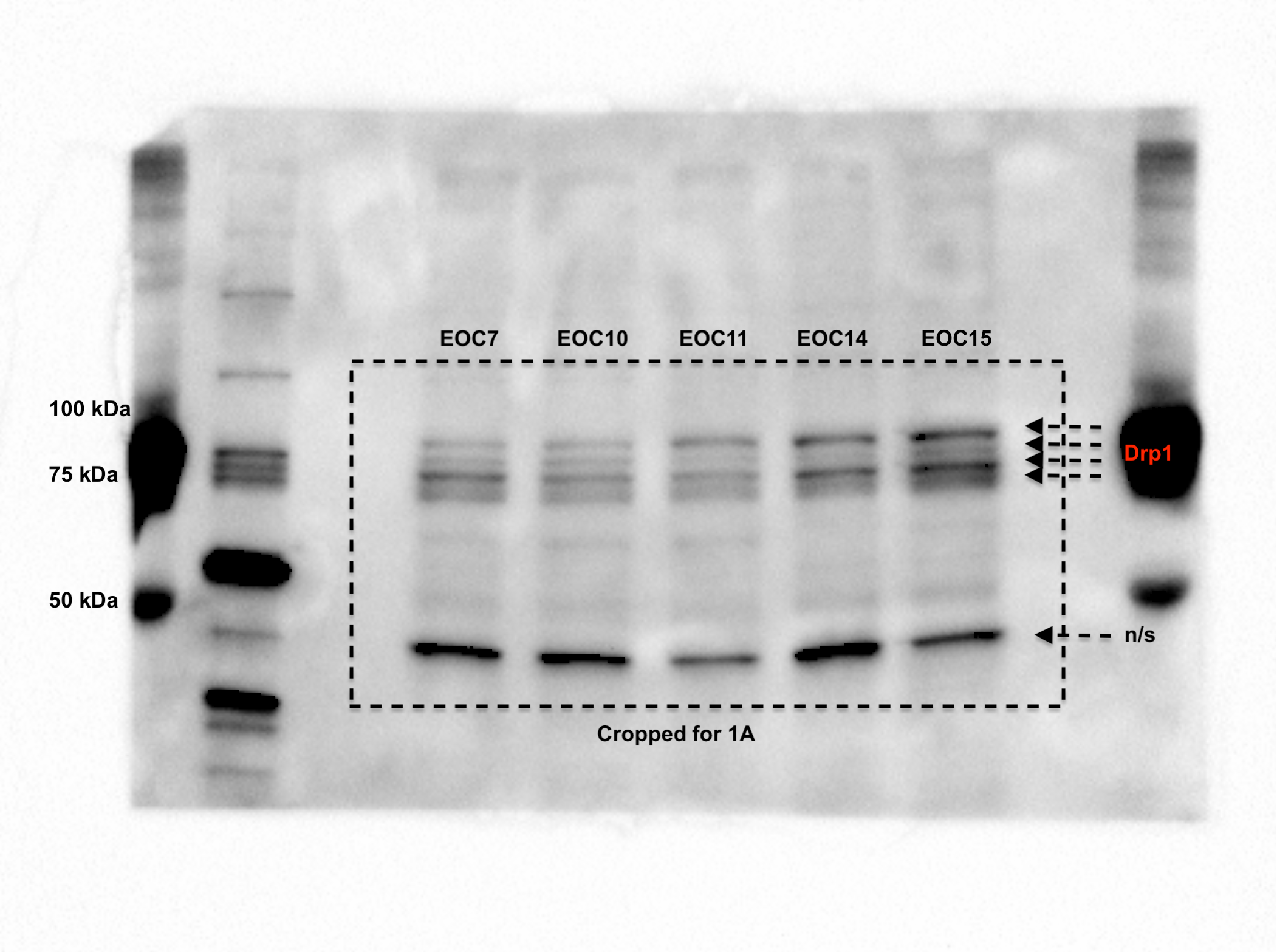

Supplement: Supplementary file 3 — Source data Fig. 1 [file 44319_2024_232_MOESM3_ESM.zip › Figure 1/1A/1A_EOC 7.10.11.14.15. poly drp1.tif]

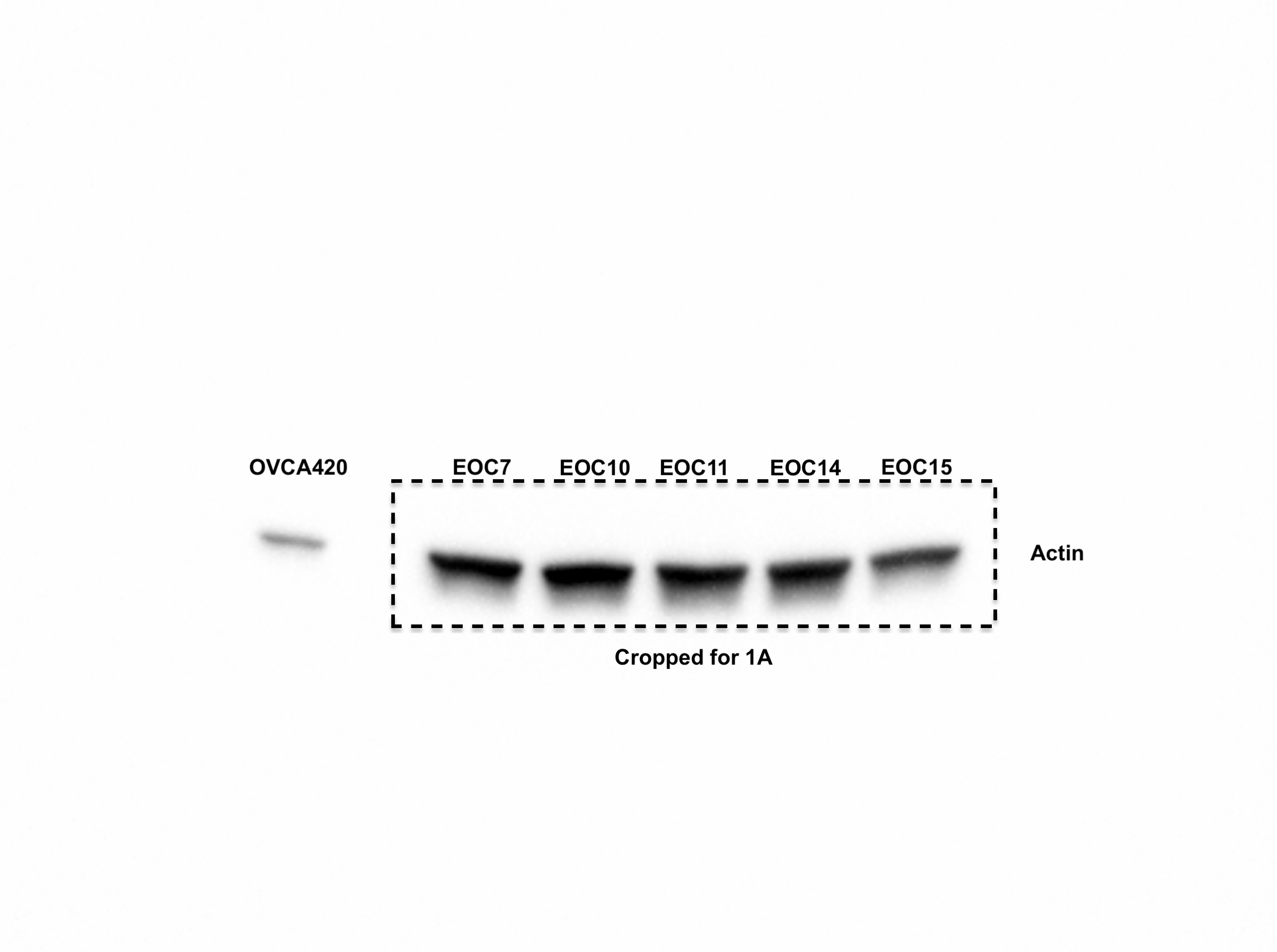

Supplement: Supplementary file 3 — Source data Fig. 1 [file 44319_2024_232_MOESM3_ESM.zip › Figure 1/1A/1A_EOC 7.10.11.14.15.actin.tif]

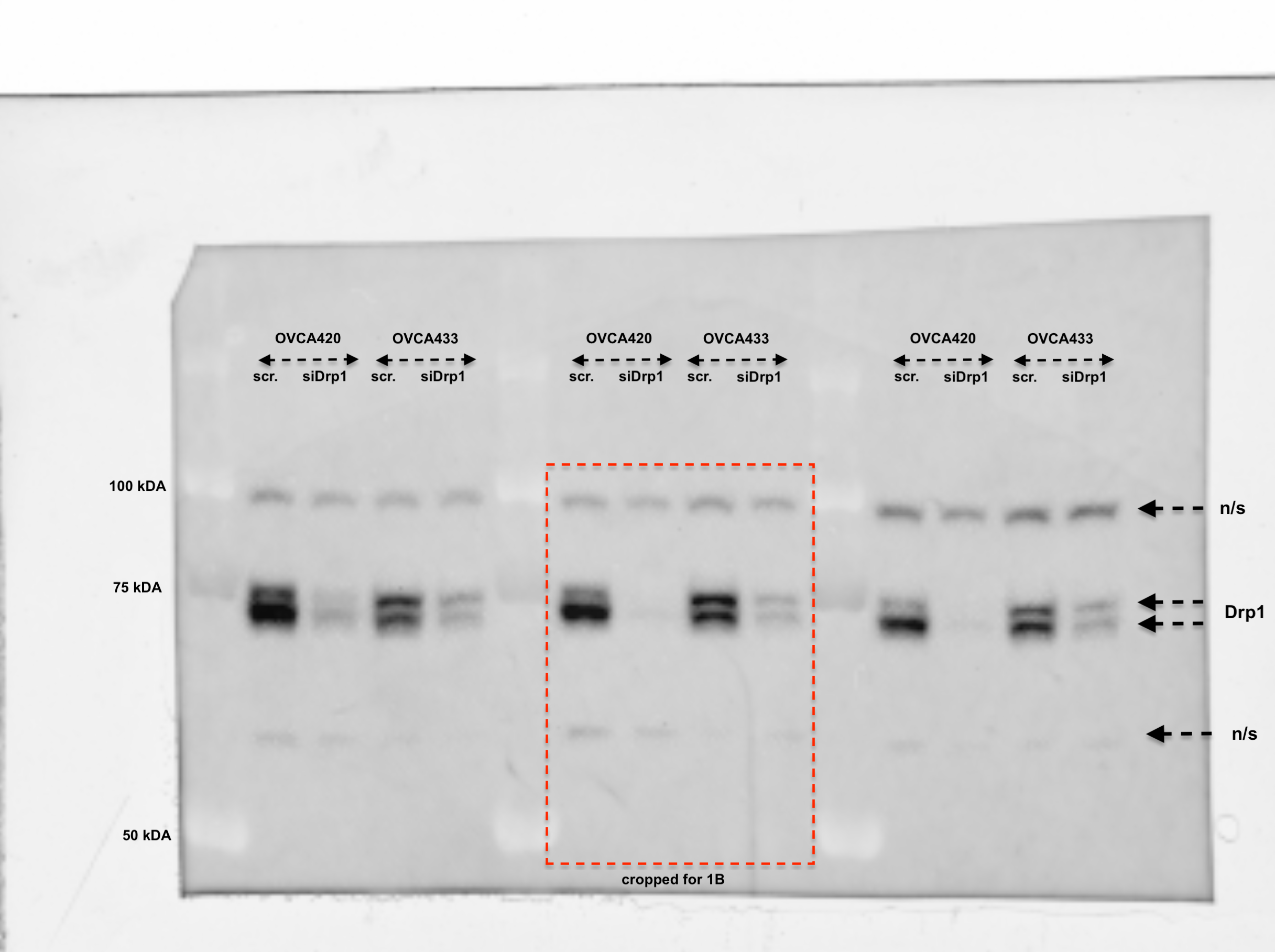

Supplement: Supplementary file 3 — Source data Fig. 1 [file 44319_2024_232_MOESM3_ESM.zip › Figure 1/1B/1B_Drp1_OVCA420_OVCA433.tif]

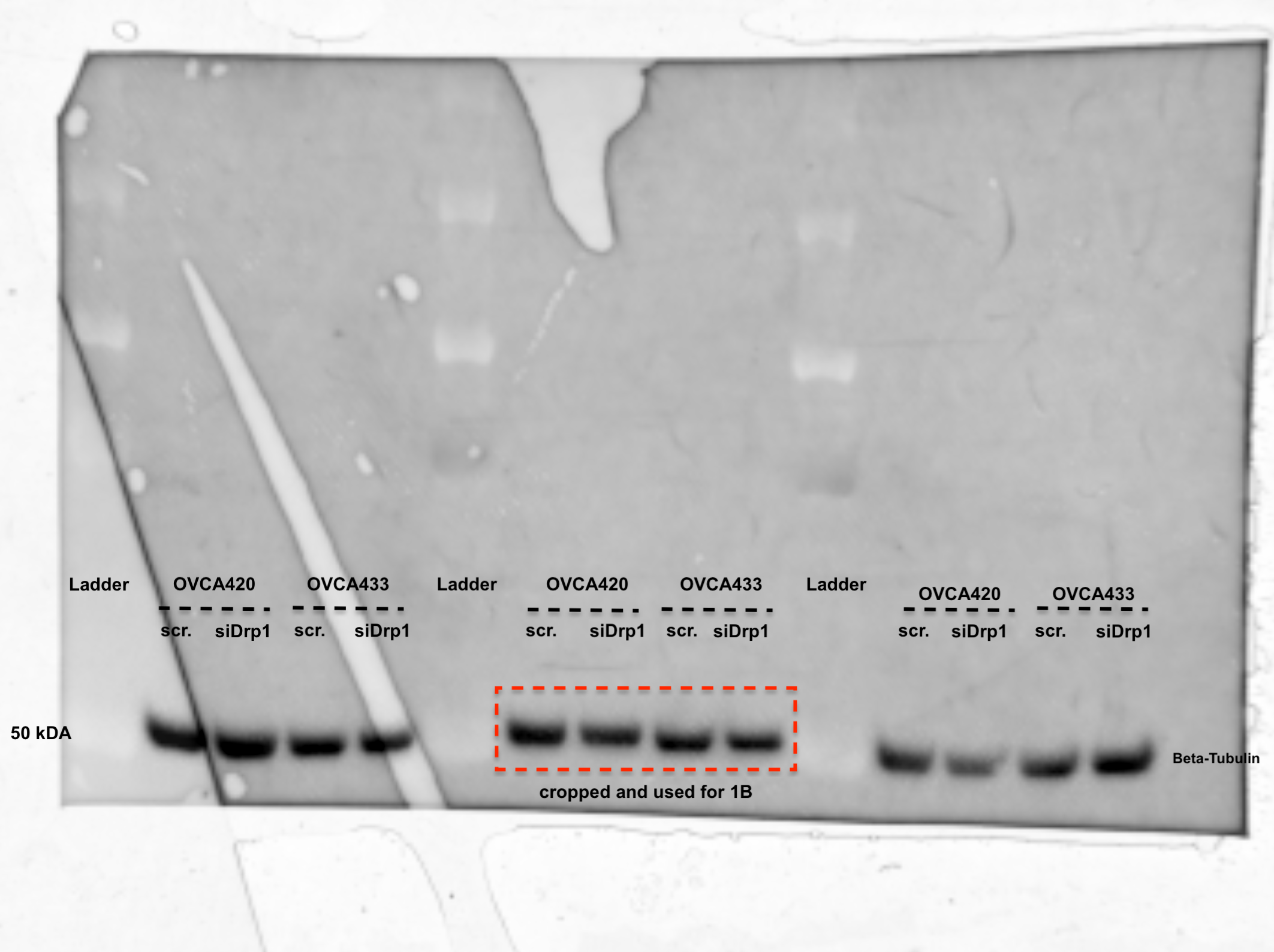

Supplement: Supplementary file 3 — Source data Fig. 1 [file 44319_2024_232_MOESM3_ESM.zip › Figure 1/1B/1B_Tubulin_OVCA420_OVCA433.tif]

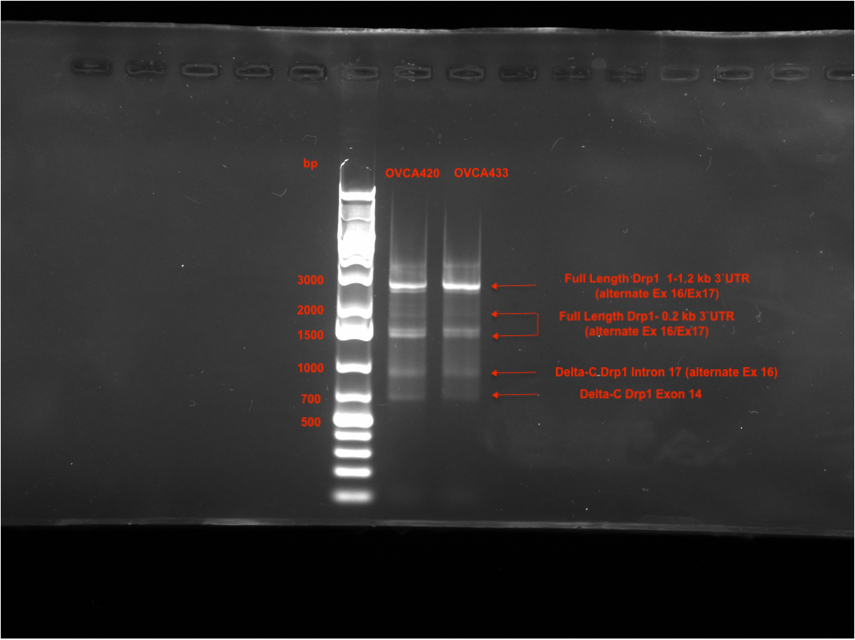

Supplement: Supplementary file 3 — Source data Fig. 1 [file 44319_2024_232_MOESM3_ESM.zip › Figure 1/1C/1C_3RACE_PCR.tif]

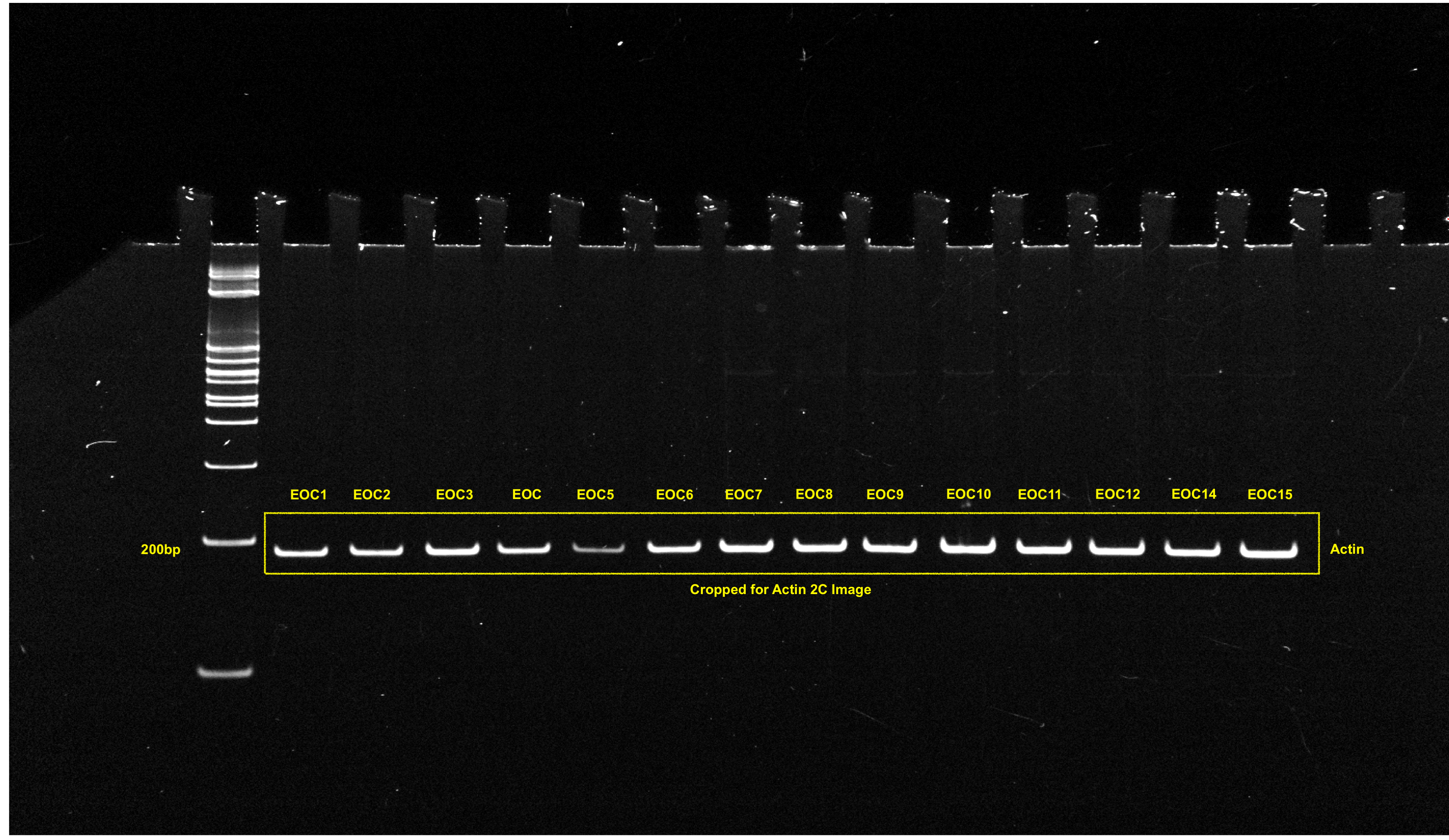

Supplement: Supplementary file 4 — Source data Fig. 2 [file 44319_2024_232_MOESM4_ESM.zip › Figure 2/2C/2C_Actin_RTpcr.png]

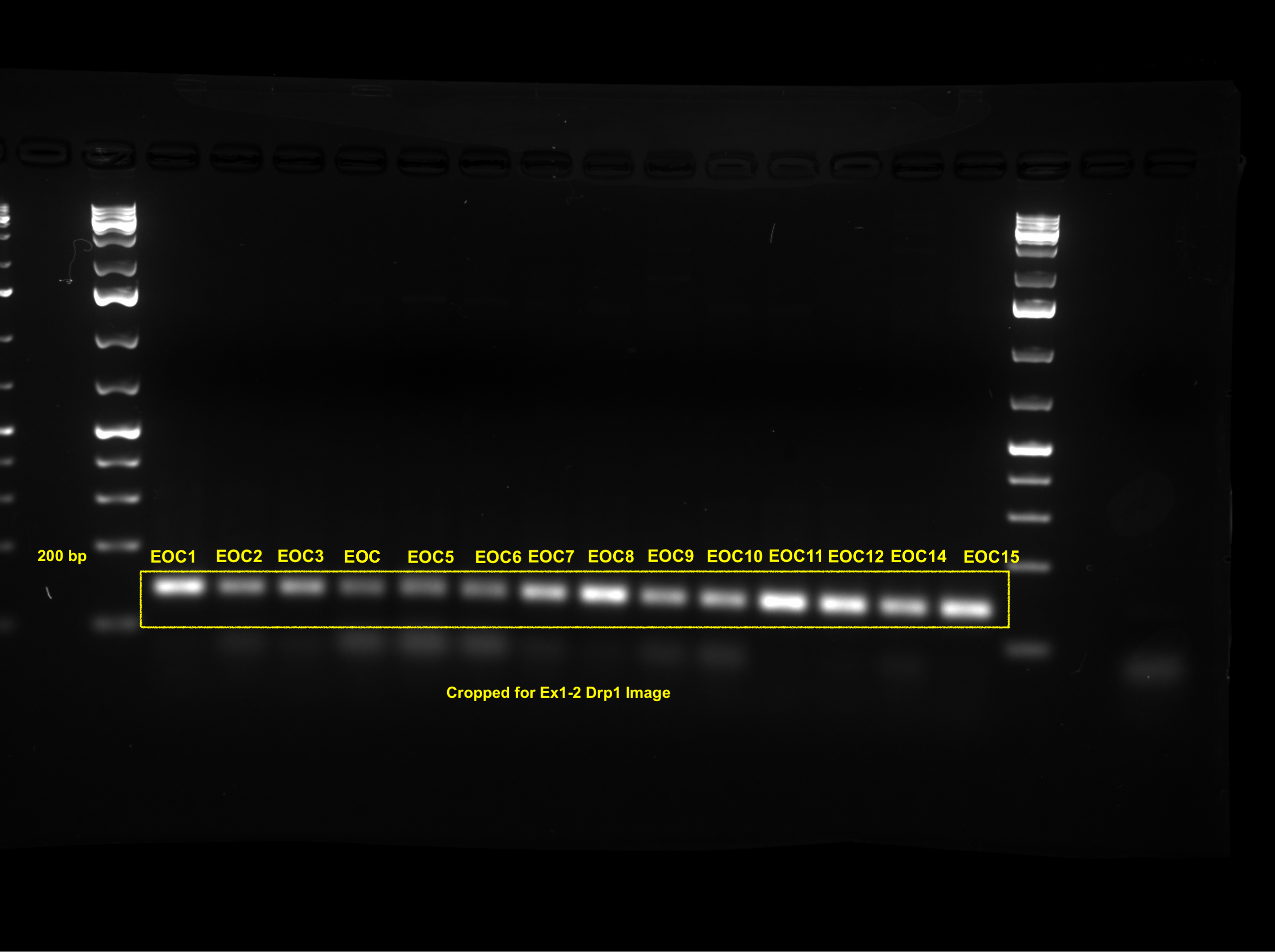

Supplement: Supplementary file 4 — Source data Fig. 2 [file 44319_2024_232_MOESM4_ESM.zip › Figure 2/2C/2C_Ex1_2Drp1RTpcr_EOC.tif]

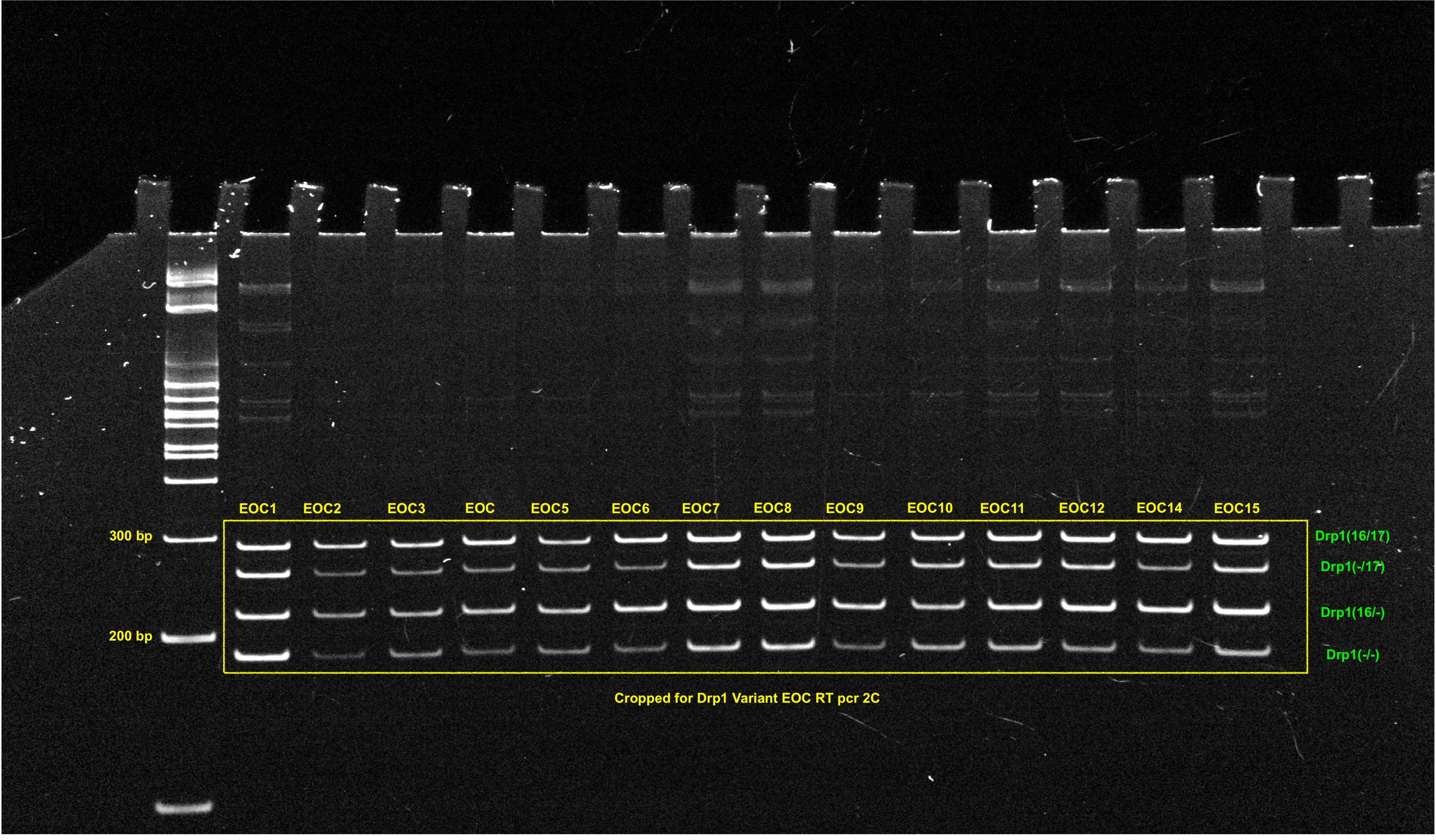

Supplement: Supplementary file 4 — Source data Fig. 2 [file 44319_2024_232_MOESM4_ESM.zip › Figure 2/2C/2C_Drp1variantsRTpcr_EOC.png]

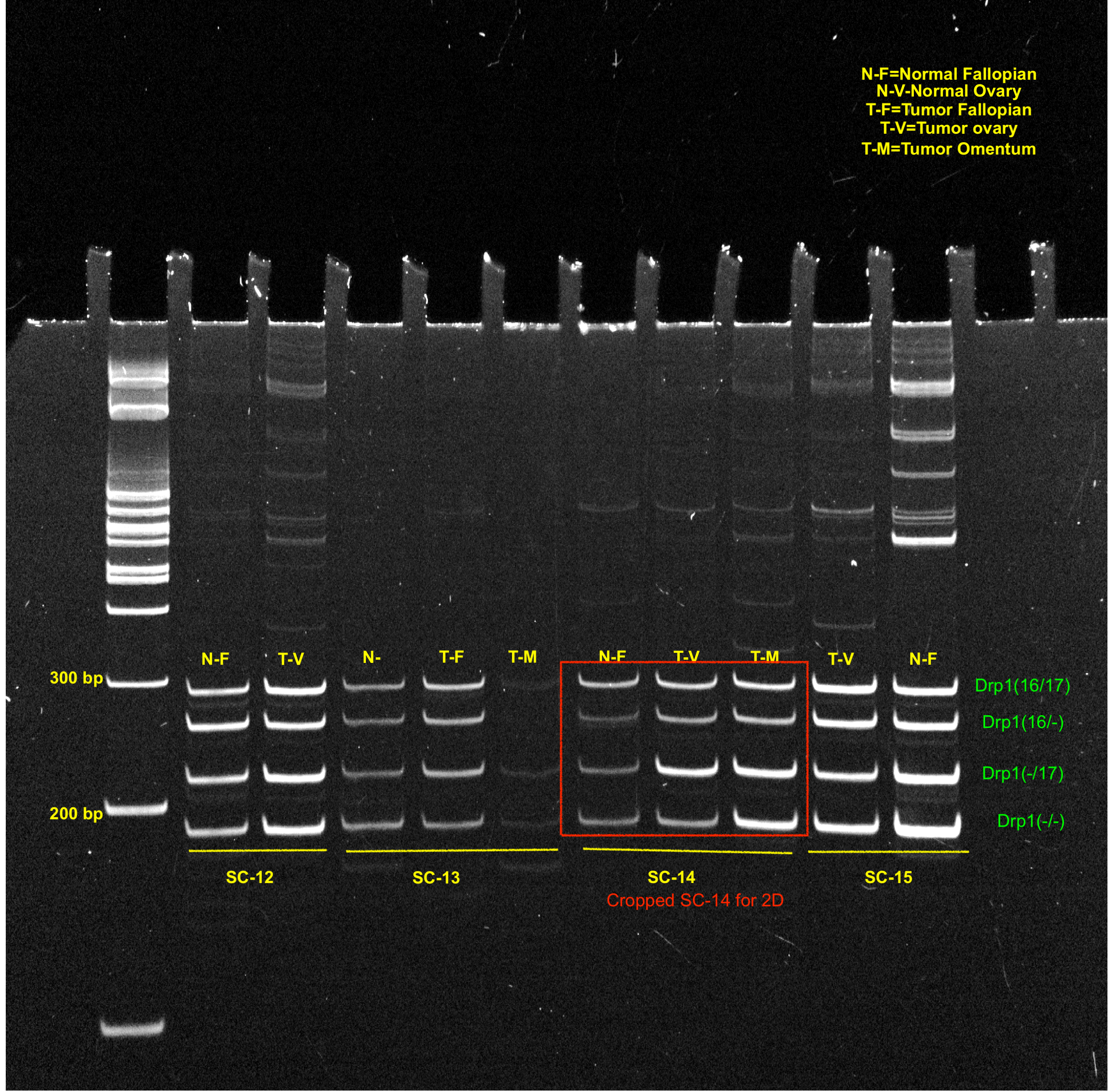

Supplement: Supplementary file 4 — Source data Fig. 2 [file 44319_2024_232_MOESM4_ESM.zip › Figure 2/2D/2D_Drp1Variants_RTPCR_SC14.png]

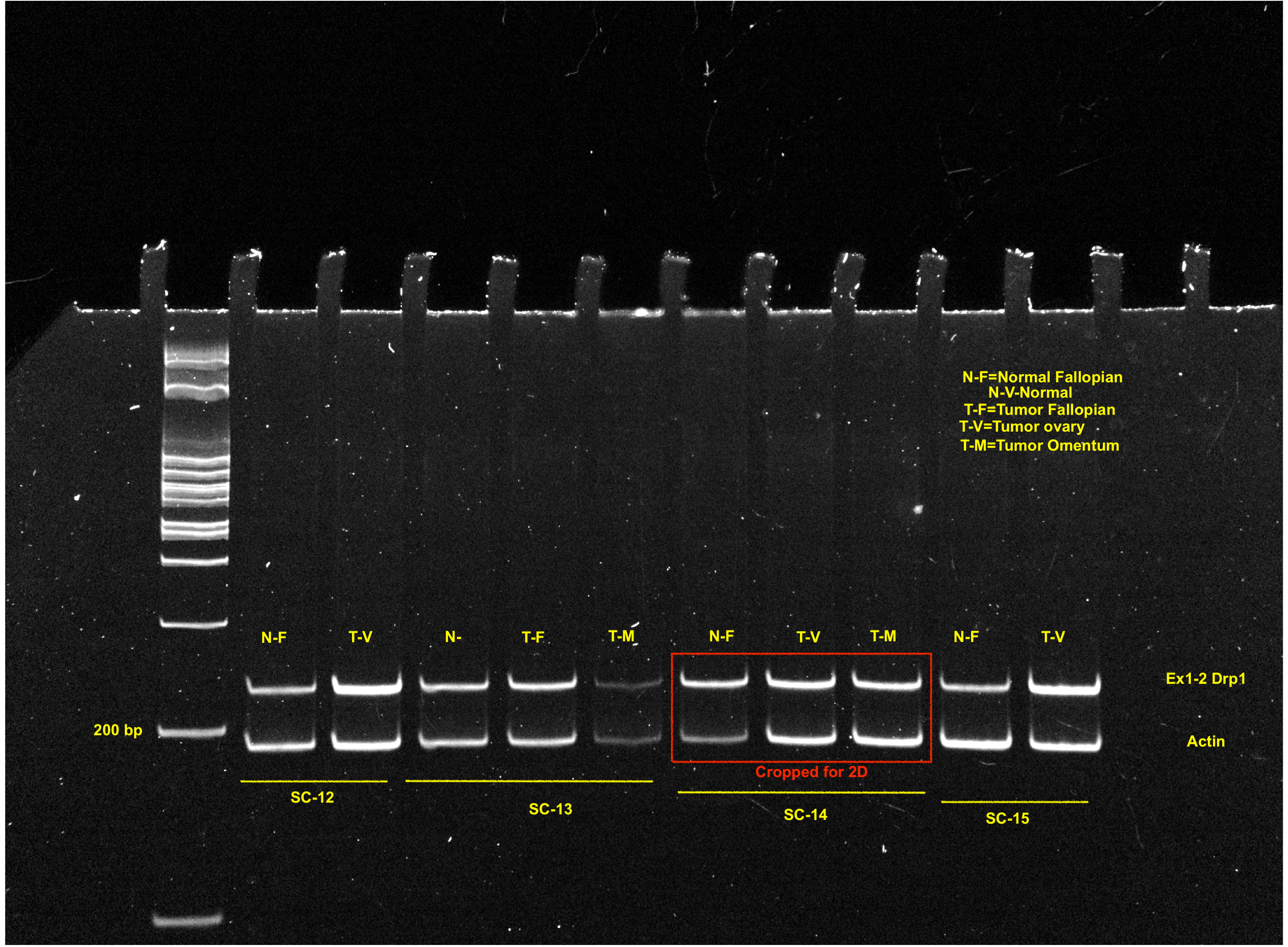

Supplement: Supplementary file 4 — Source data Fig. 2 [file 44319_2024_232_MOESM4_ESM.zip › Figure 2/2D/2D_Actin_Ex1-2_RTPCR_SC14.png]

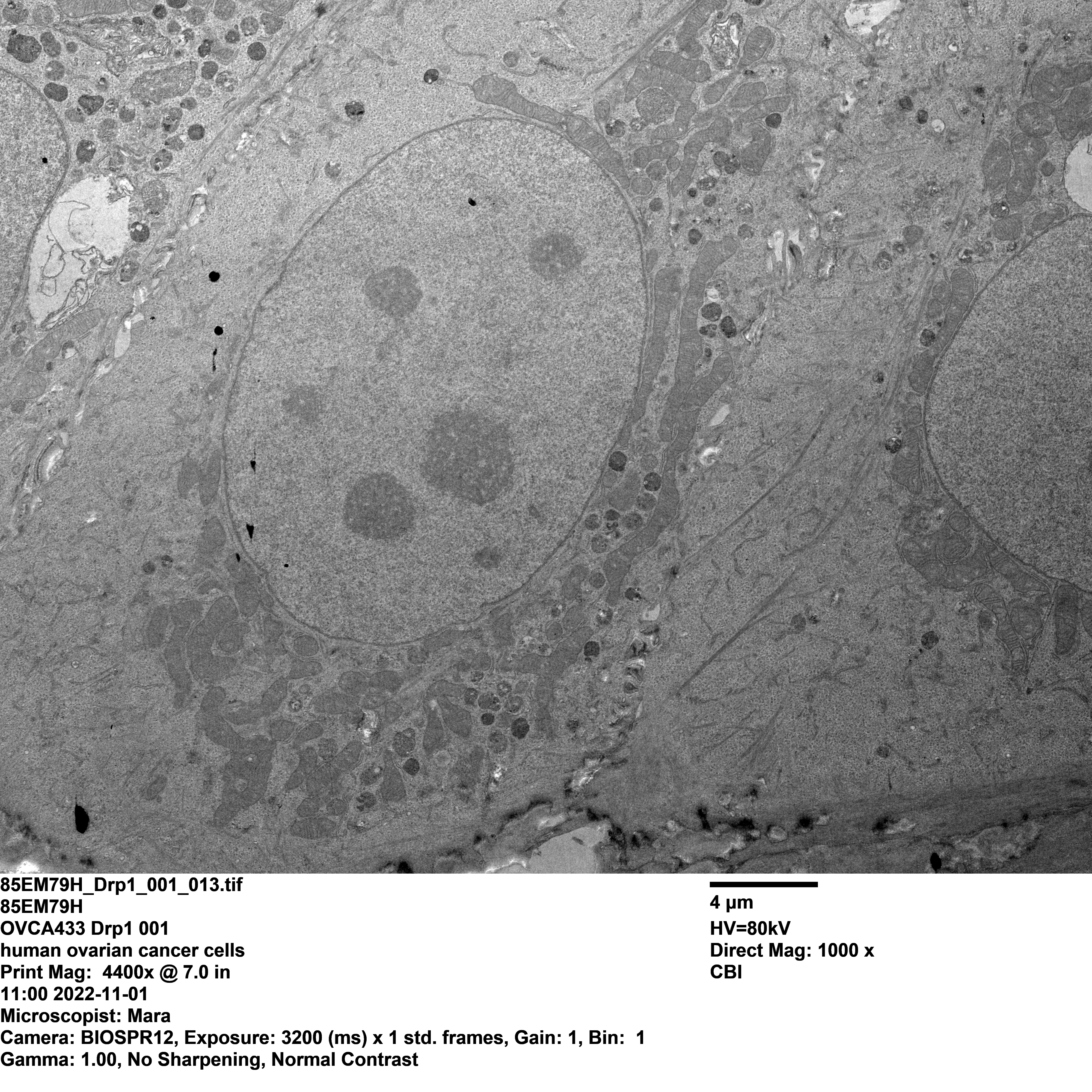

Supplement: Supplementary file 5 — Source data Fig. 3 [file 44319_2024_232_MOESM5_ESM.zip › Figure 3/3E/3E_Drp1(-17)_TEM.tif]

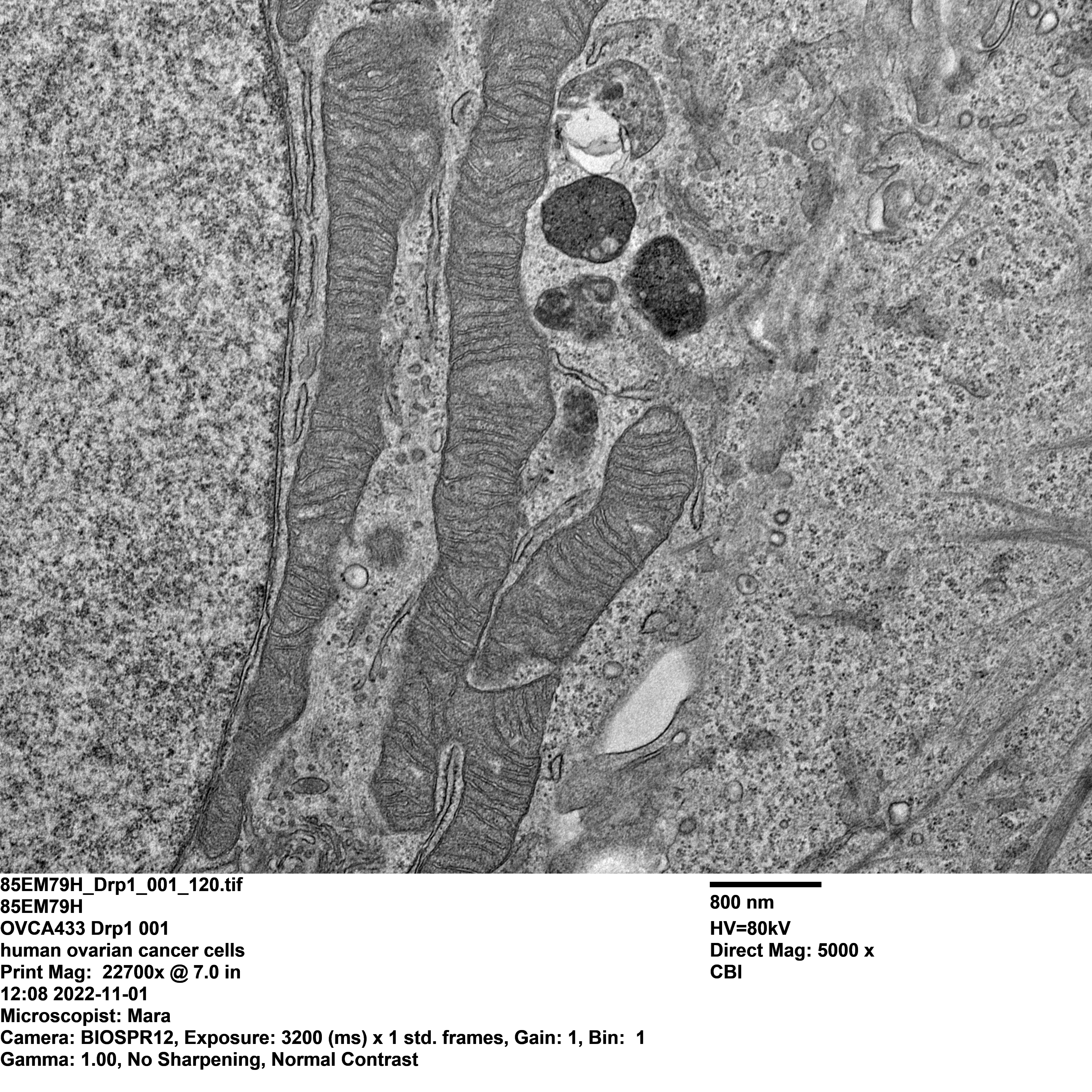

Supplement: Supplementary file 5 — Source data Fig. 3 [file 44319_2024_232_MOESM5_ESM.zip › Figure 3/3E/3E_Drp1(-17)_mitoTEM.tif]

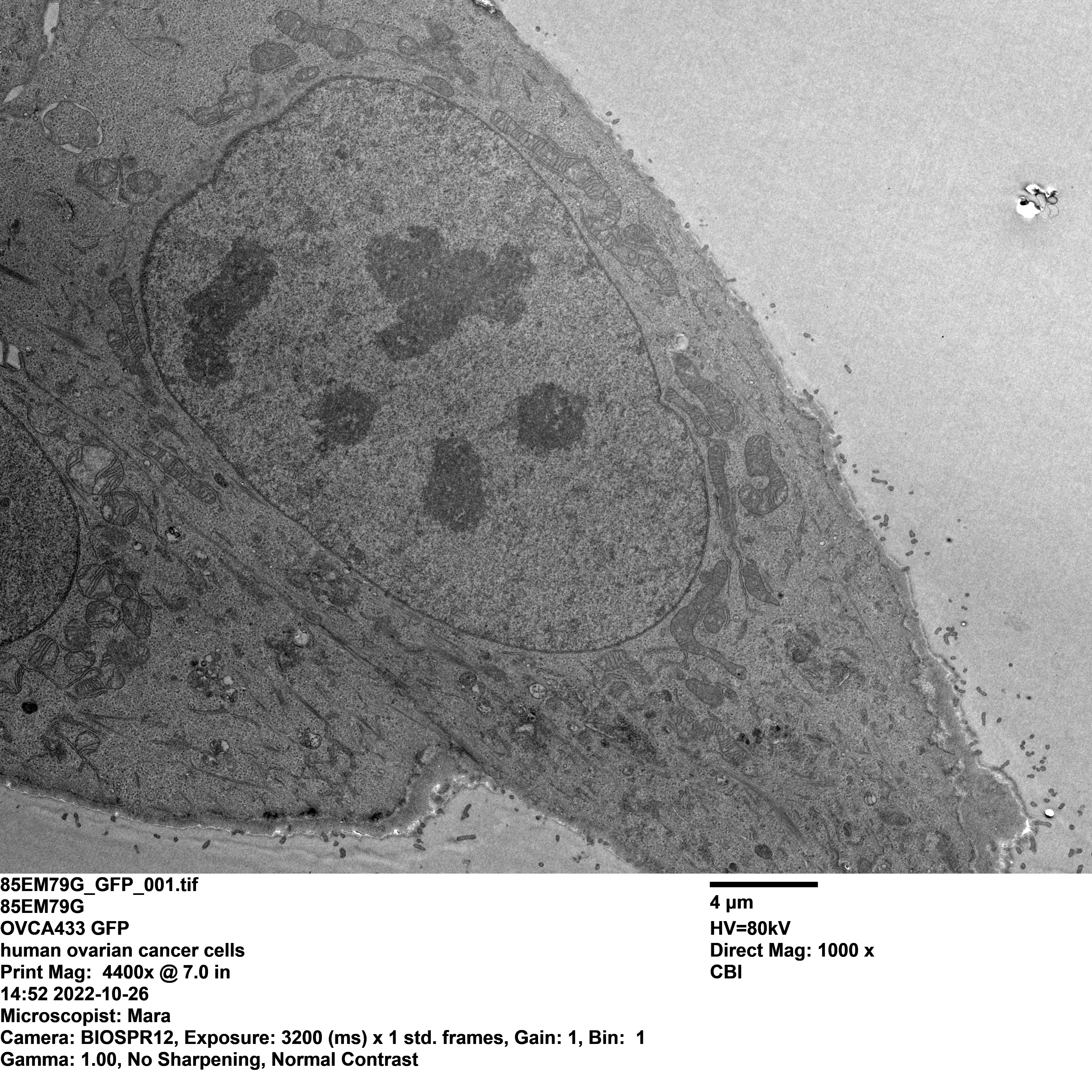

Supplement: Supplementary file 5 — Source data Fig. 3 [file 44319_2024_232_MOESM5_ESM.zip › Figure 3/3E/3E_GFP_TEM.tif]

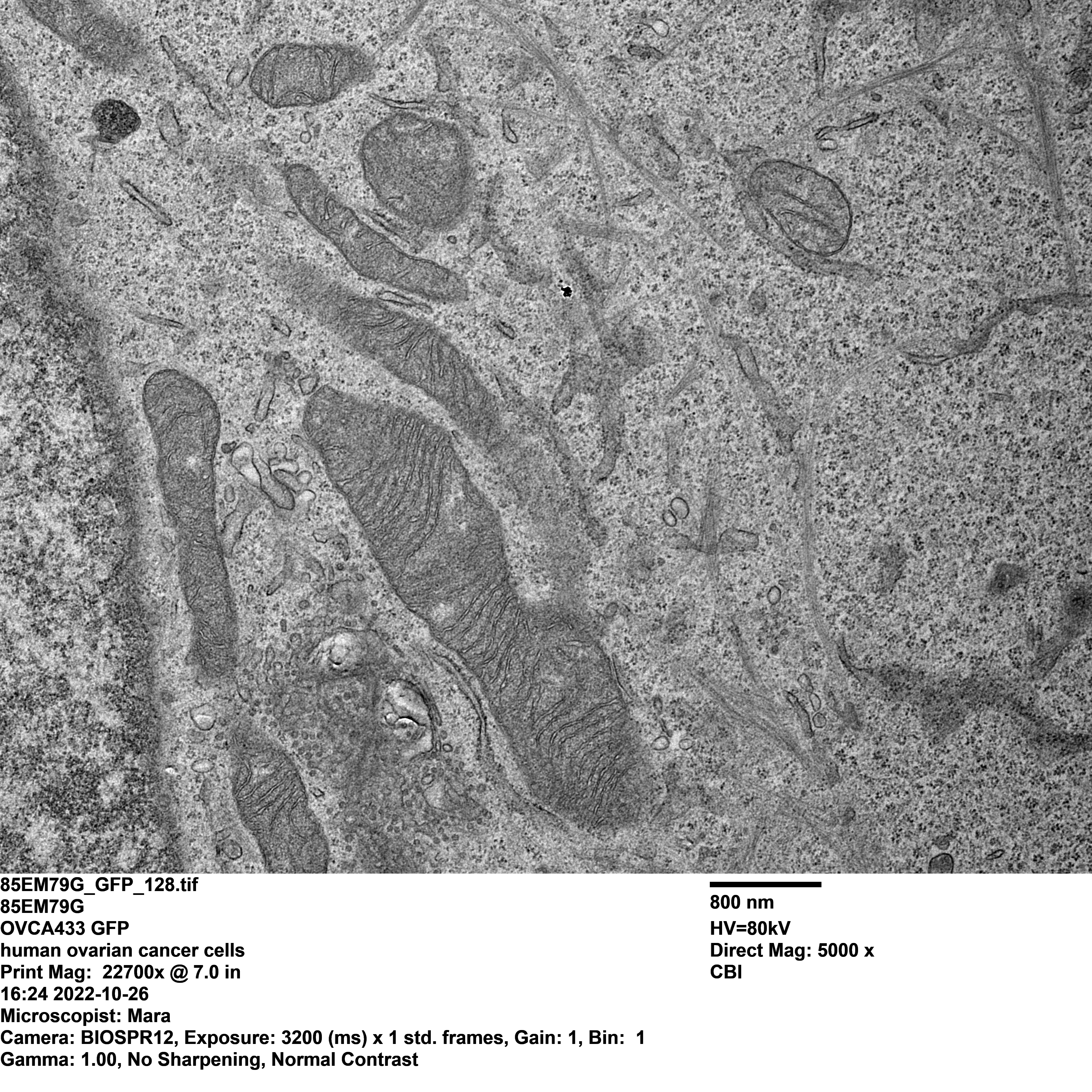

Supplement: Supplementary file 5 — Source data Fig. 3 [file 44319_2024_232_MOESM5_ESM.zip › Figure 3/3E/3E_GFP_mitoTEM.tif]

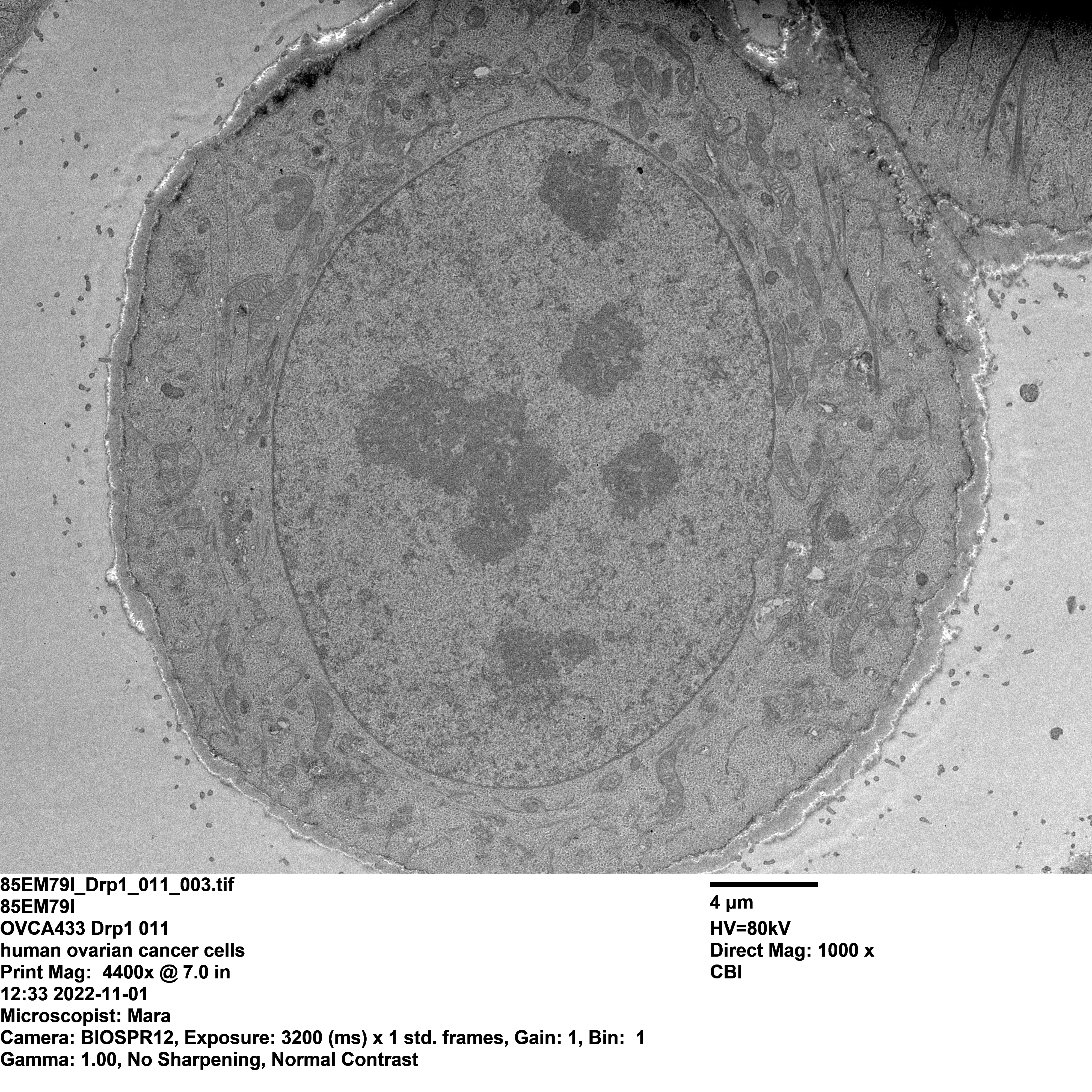

Supplement: Supplementary file 5 — Source data Fig. 3 [file 44319_2024_232_MOESM5_ESM.zip › Figure 3/3E/3E_Drp1(1617)_TEM.tif]

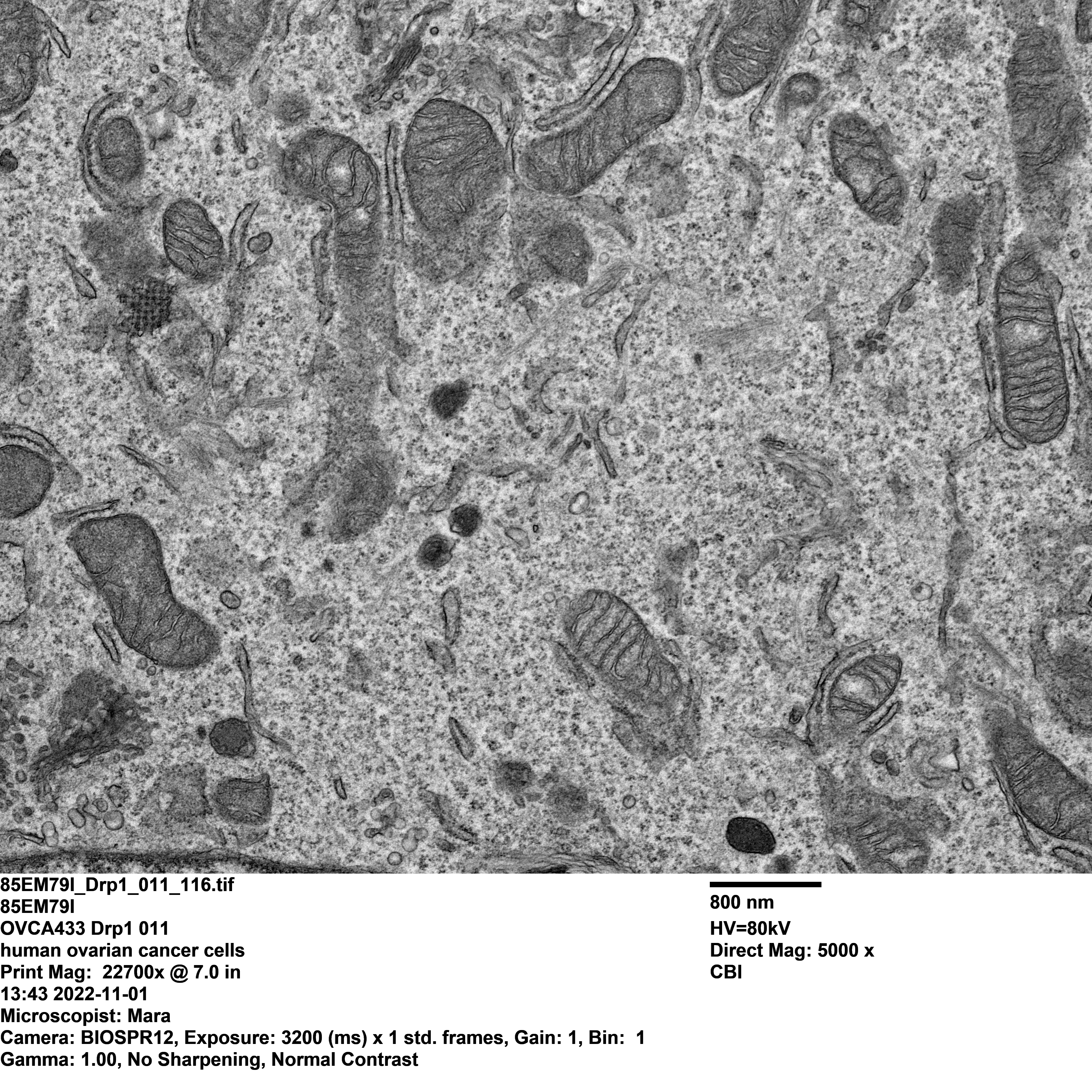

Supplement: Supplementary file 5 — Source data Fig. 3 [file 44319_2024_232_MOESM5_ESM.zip › Figure 3/3E/3E_Drp1(1617)_mitoTEM.tif]

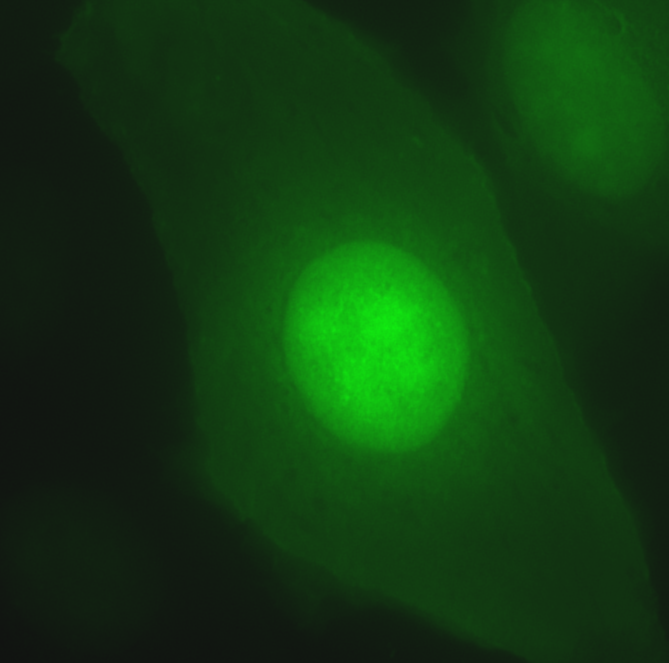

Supplement: Supplementary file 5 — Source data Fig. 3 [file 44319_2024_232_MOESM5_ESM.zip › Figure 3/3B/3B_GFP_GFPonlyl.tif]

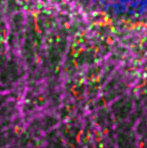

Supplement: Supplementary file 5 — Source data Fig. 3 [file 44319_2024_232_MOESM5_ESM.zip › Figure 3/3B/3B_Drp1(1617)_zoomed.png]

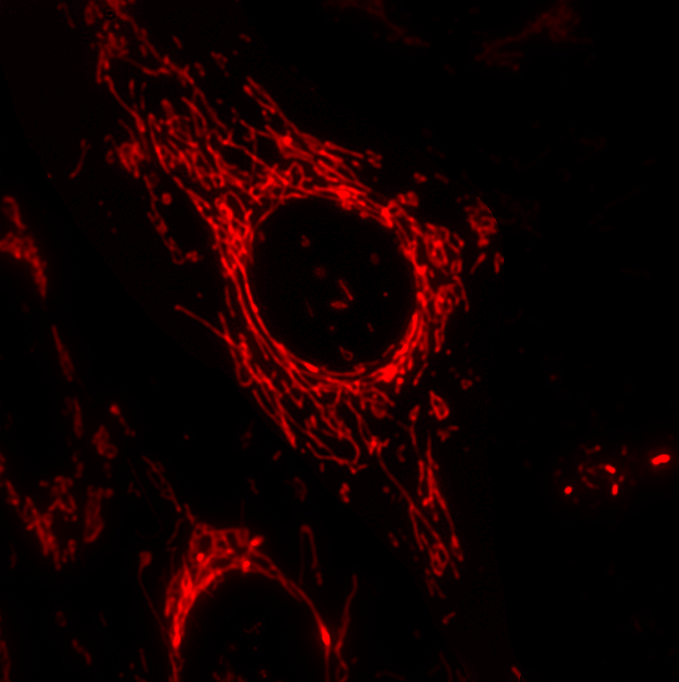

Supplement: Supplementary file 5 — Source data Fig. 3 [file 44319_2024_232_MOESM5_ESM.zip › Figure 3/3B/3B_Drp1(-17)_mitotrackeronly.tif]

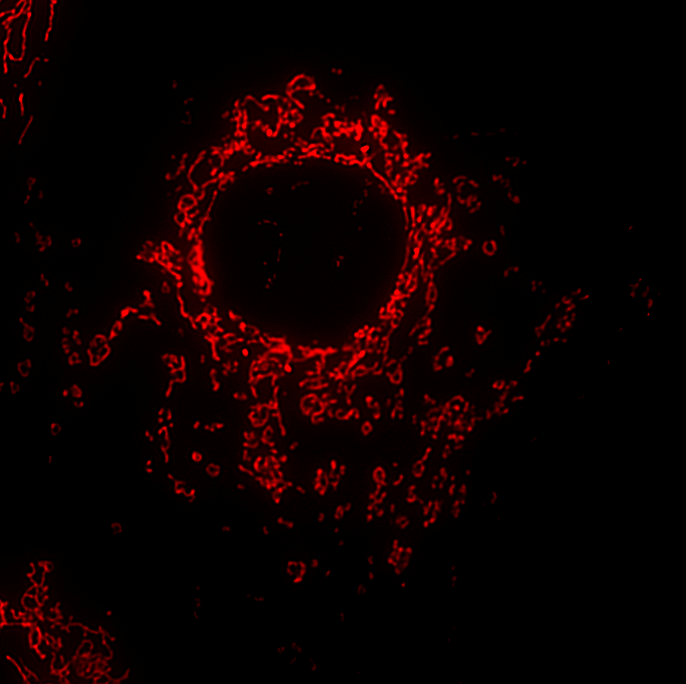

Supplement: Supplementary file 5 — Source data Fig. 3 [file 44319_2024_232_MOESM5_ESM.zip › Figure 3/3B/3B_Drp1(1617)_mitotrackeronly.tif]

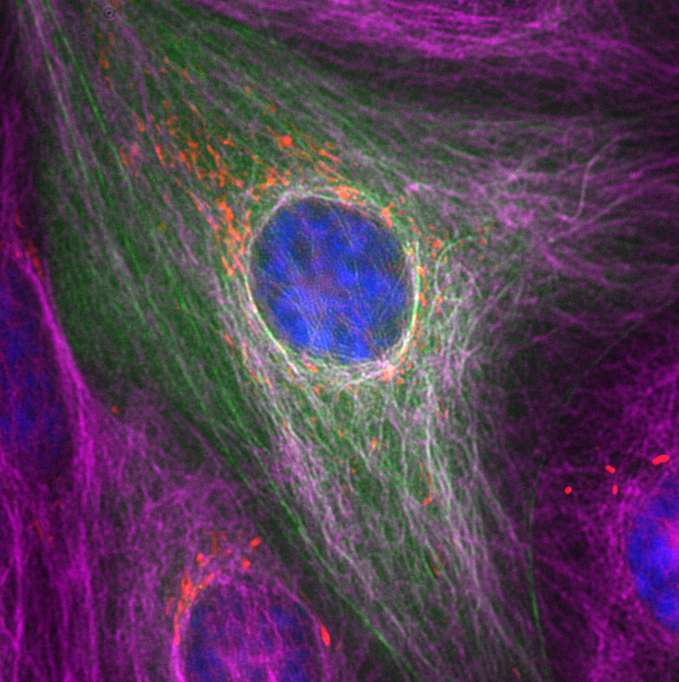

Supplement: Supplementary file 5 — Source data Fig. 3 [file 44319_2024_232_MOESM5_ESM.zip › Figure 3/3B/3B_Drp1(-17)_GFP_mitotracker_tubulin.png]

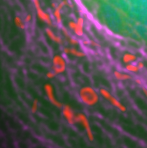

Supplement: Supplementary file 5 — Source data Fig. 3 [file 44319_2024_232_MOESM5_ESM.zip › Figure 3/3B/3B_GFP_Zoomed.png]

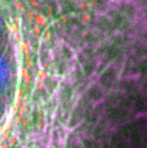

Supplement: Supplementary file 5 — Source data Fig. 3 [file 44319_2024_232_MOESM5_ESM.zip › Figure 3/3B/3B_Drp1(-17)_zoomed.png]

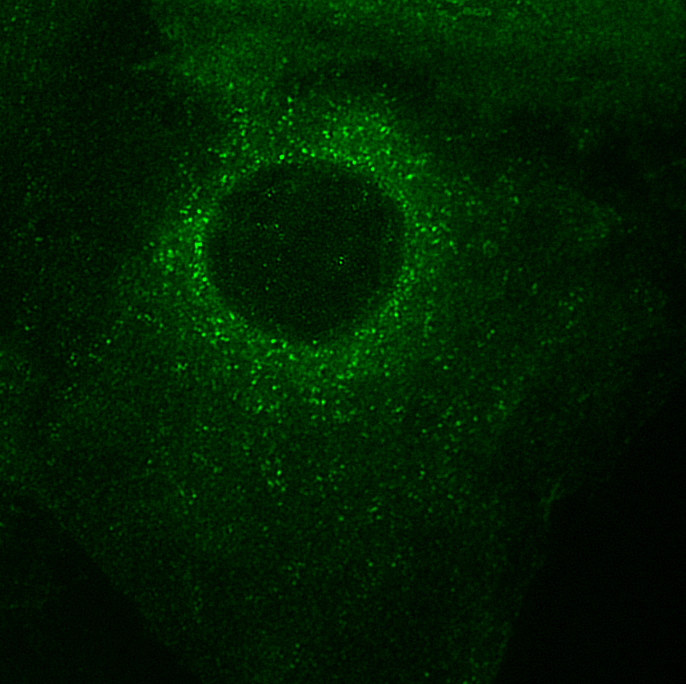

Supplement: Supplementary file 5 — Source data Fig. 3 [file 44319_2024_232_MOESM5_ESM.zip › Figure 3/3B/3B_Drp1(1617)_GFPonly.tif]

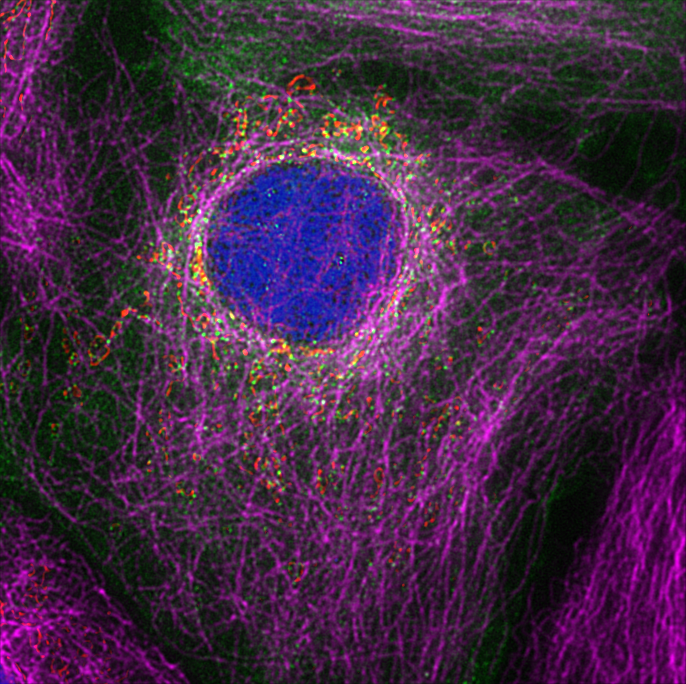

Supplement: Supplementary file 5 — Source data Fig. 3 [file 44319_2024_232_MOESM5_ESM.zip › Figure 3/3B/3B_Drp1(1617)_GFP_mitotracker_tubulin.png]

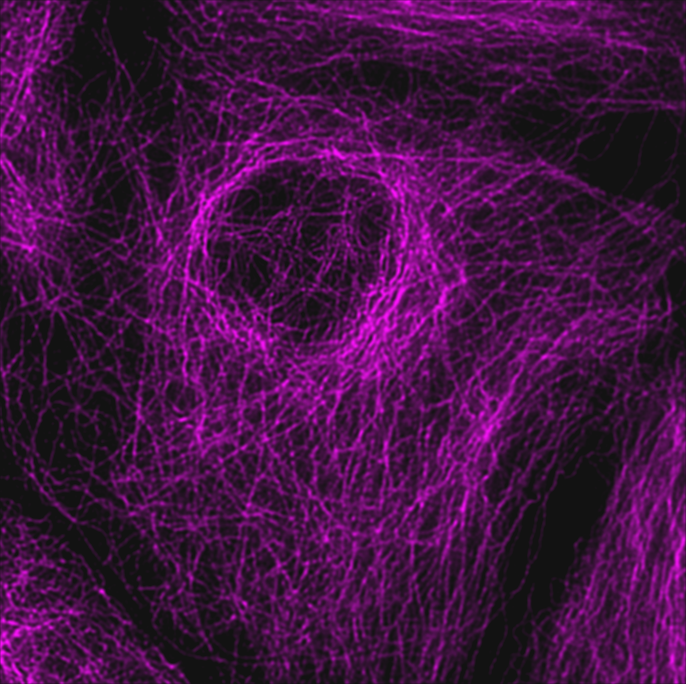

Supplement: Supplementary file 5 — Source data Fig. 3 [file 44319_2024_232_MOESM5_ESM.zip › Figure 3/3B/3B_Drp1(1617)_tubulinonly.tif]

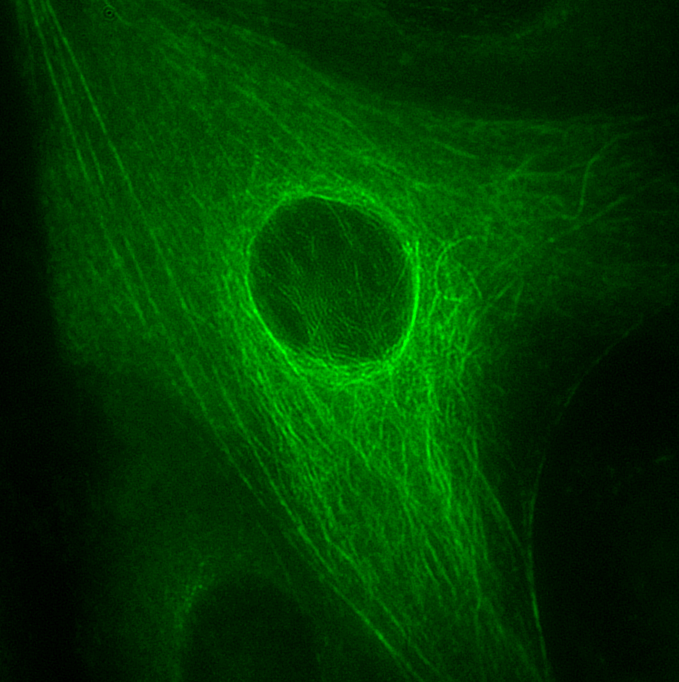

Supplement: Supplementary file 5 — Source data Fig. 3 [file 44319_2024_232_MOESM5_ESM.zip › Figure 3/3B/3B_Drp1(-17)_GFPonly.tif]

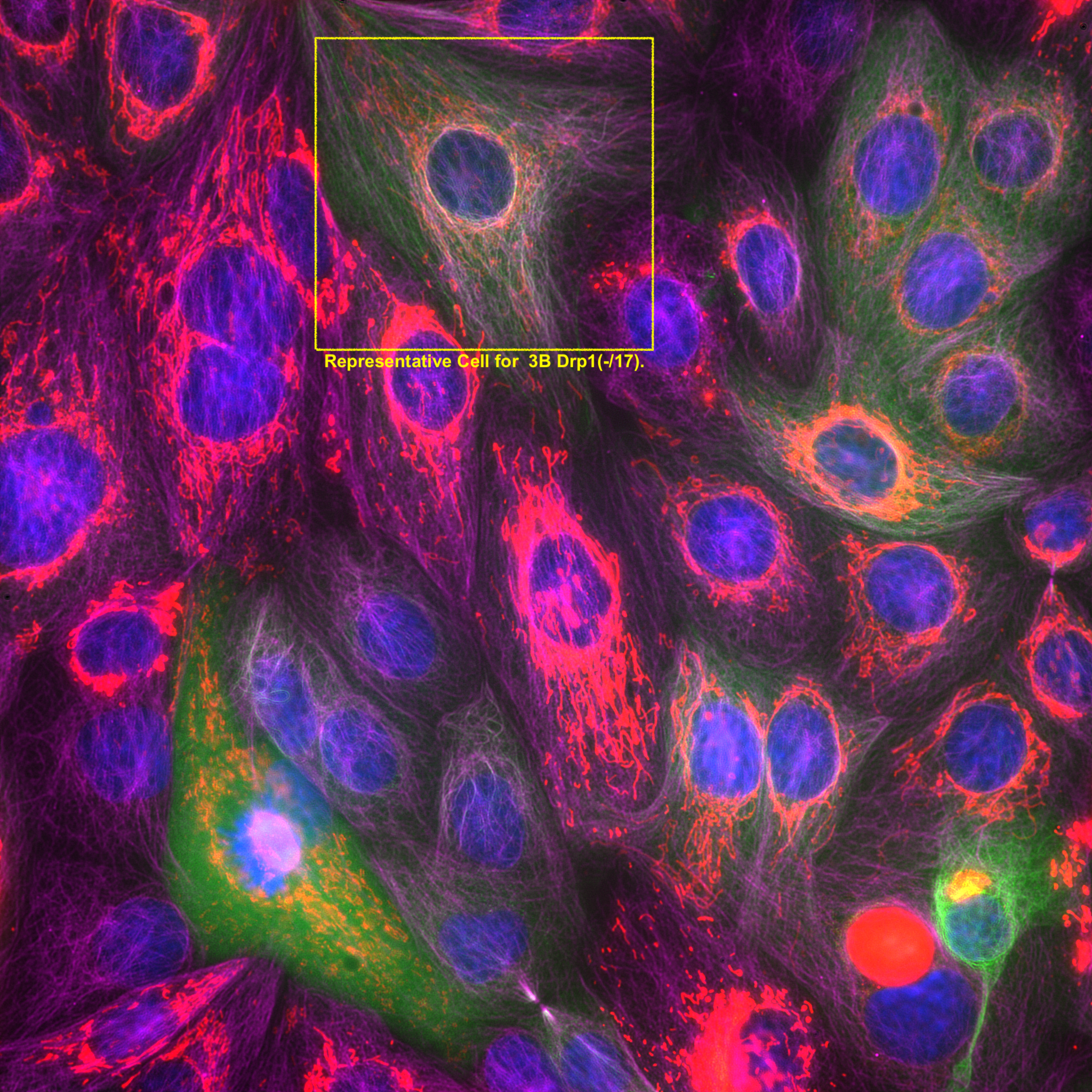

Supplement: Supplementary file 5 — Source data Fig. 3 [file 44319_2024_232_MOESM5_ESM.zip › Figure 3/3B/3B_Drp1(-17)_Orginal.tif]

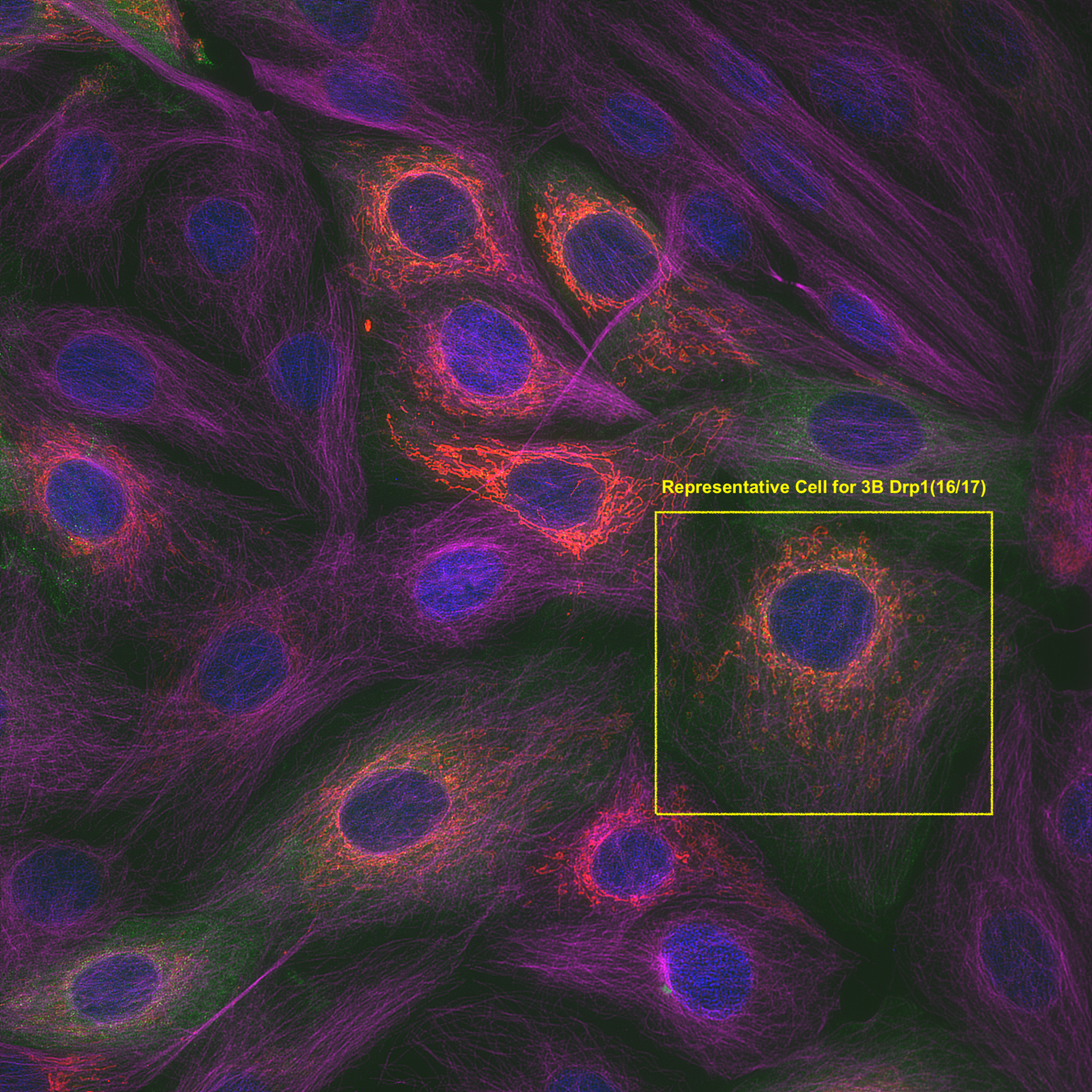

Supplement: Supplementary file 5 — Source data Fig. 3 [file 44319_2024_232_MOESM5_ESM.zip › Figure 3/3B/3B_Drp1(1617)_Orginal.tif]

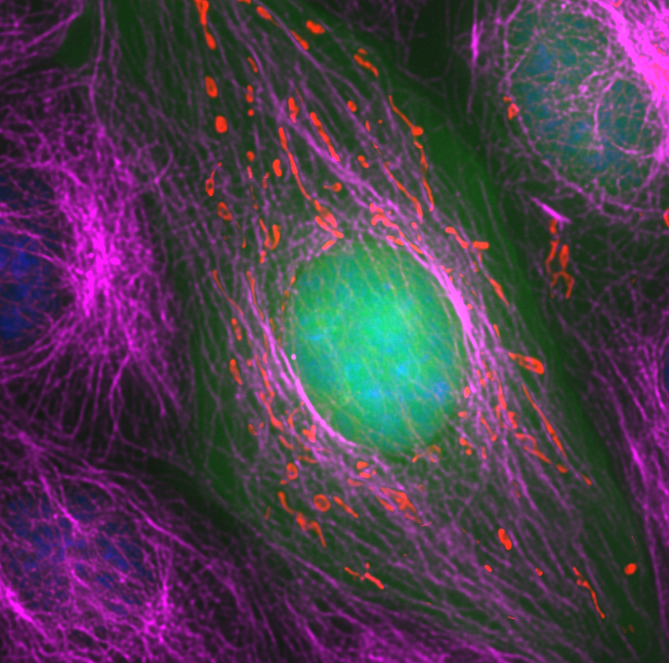

Supplement: Supplementary file 5 — Source data Fig. 3 [file 44319_2024_232_MOESM5_ESM.zip › Figure 3/3B/3B_GFP_GFP_mitotracker_tubulin.png]

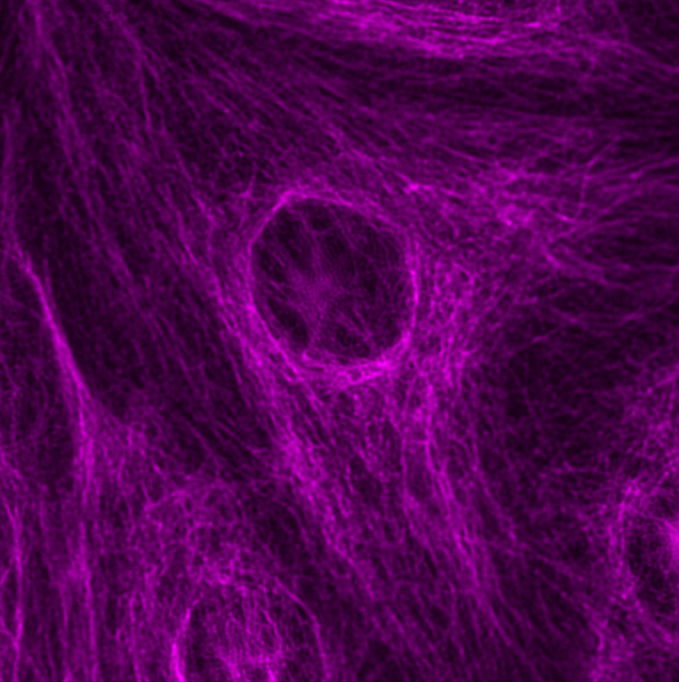

Supplement: Supplementary file 5 — Source data Fig. 3 [file 44319_2024_232_MOESM5_ESM.zip › Figure 3/3B/3B_Drp1(-17)_tubulinonly.tif]

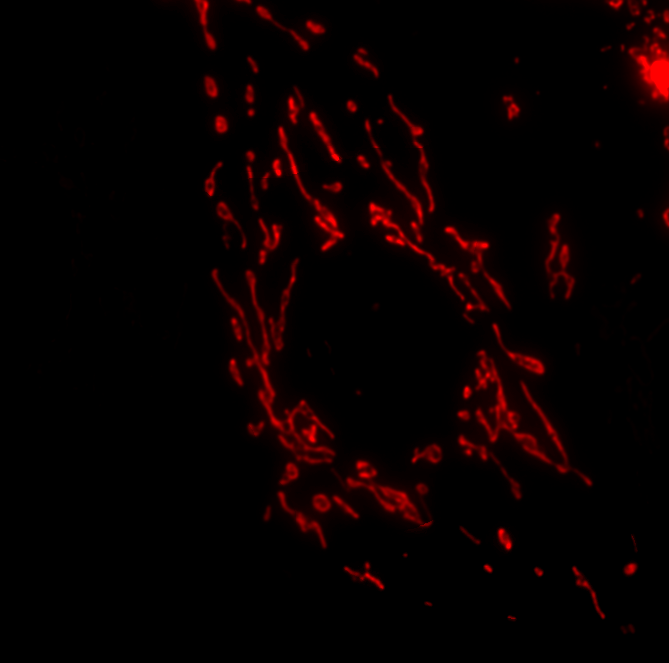

Supplement: Supplementary file 5 — Source data Fig. 3 [file 44319_2024_232_MOESM5_ESM.zip › Figure 3/3B/3B_GFP_mitotrackeronly.tif]

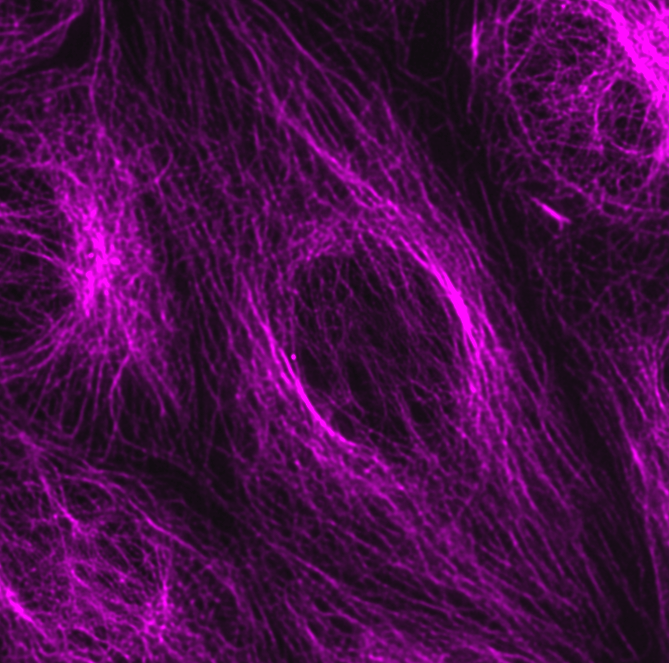

Supplement: Supplementary file 5 — Source data Fig. 3 [file 44319_2024_232_MOESM5_ESM.zip › Figure 3/3B/3B_GFP_tubulinonly.tif]

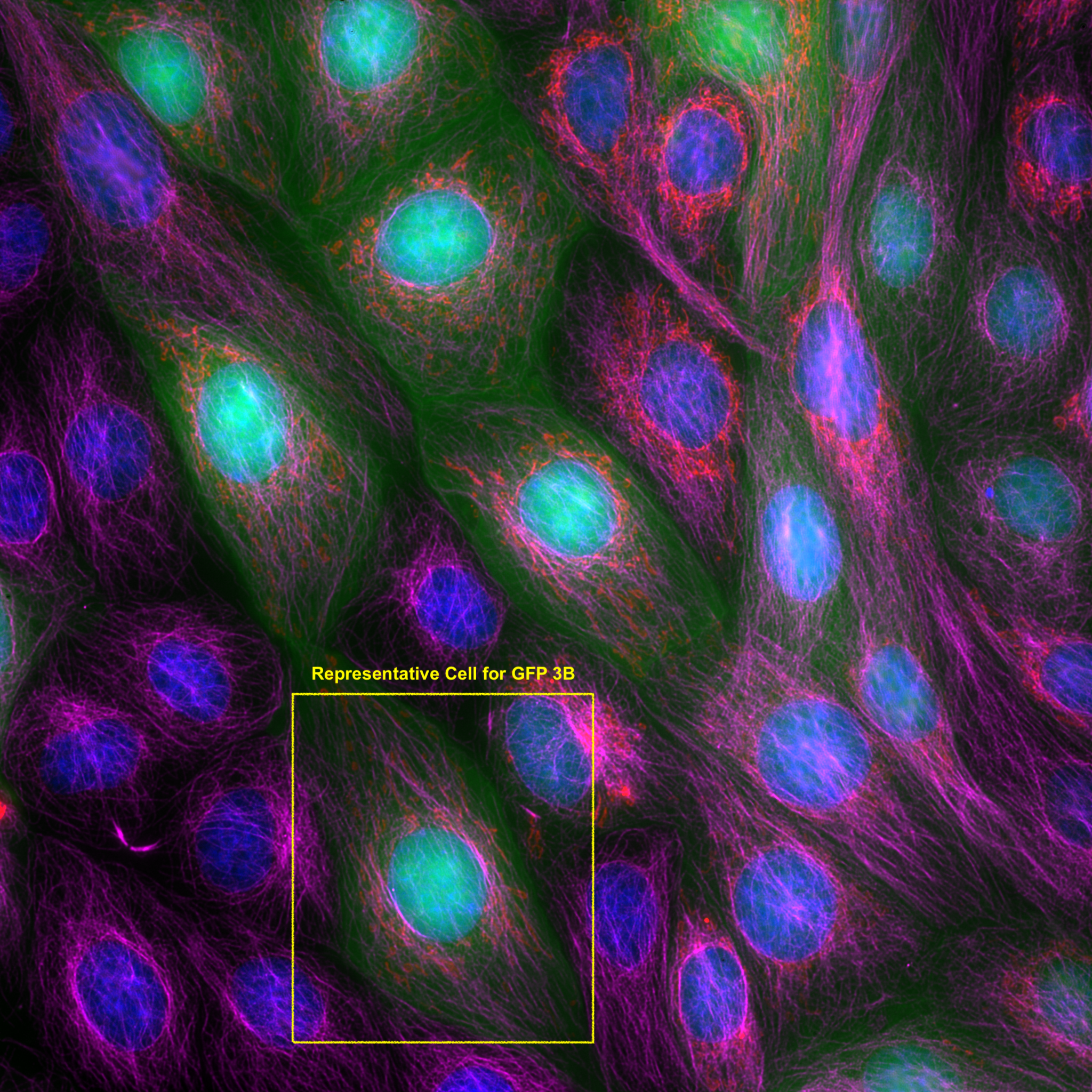

Supplement: Supplementary file 5 — Source data Fig. 3 [file 44319_2024_232_MOESM5_ESM.zip › Figure 3/3B/3B_GFP_Orginal.tif]

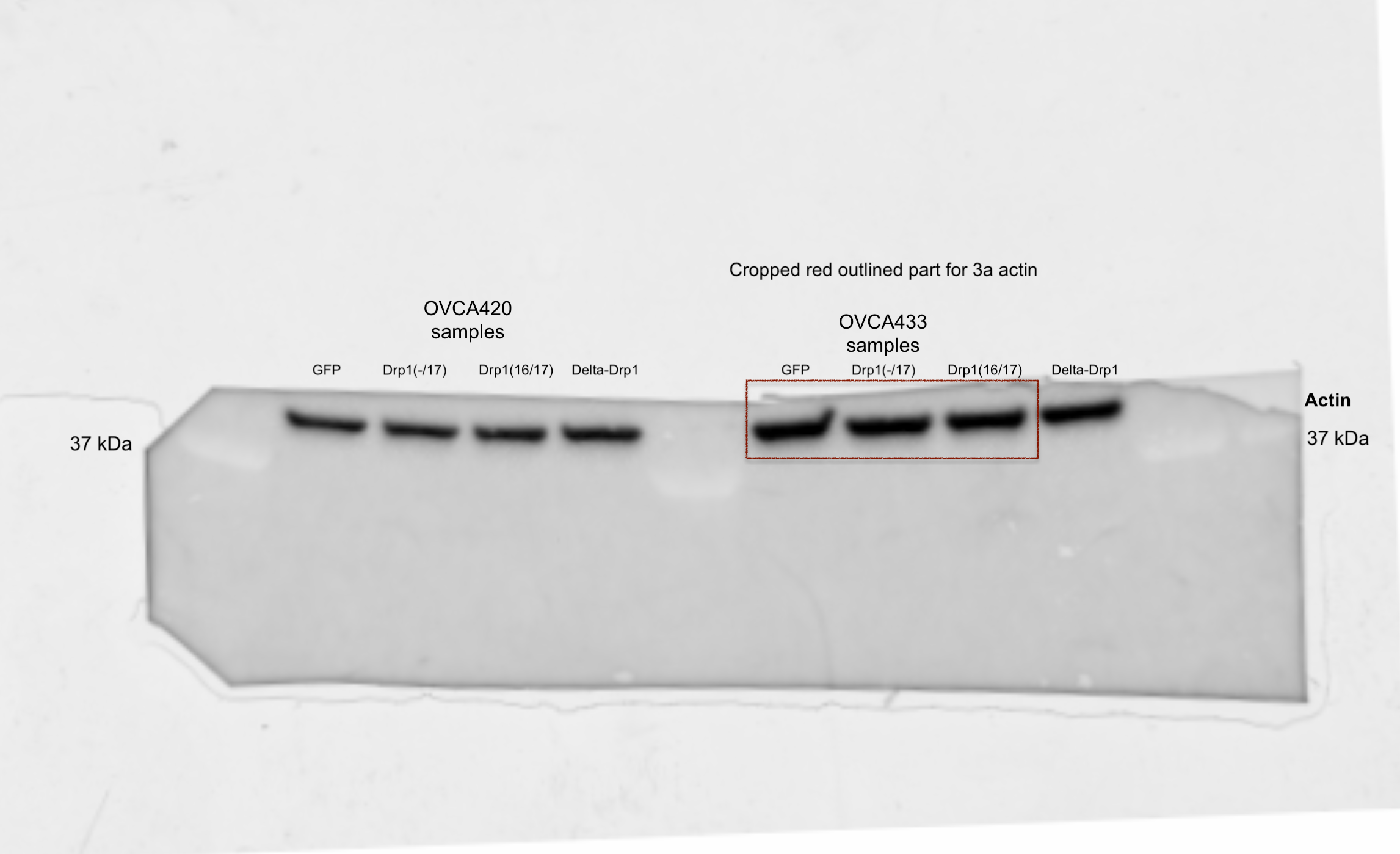

Supplement: Supplementary file 5 — Source data Fig. 3 [file 44319_2024_232_MOESM5_ESM.zip › Figure 3/3A/3A_western Actin.tif]

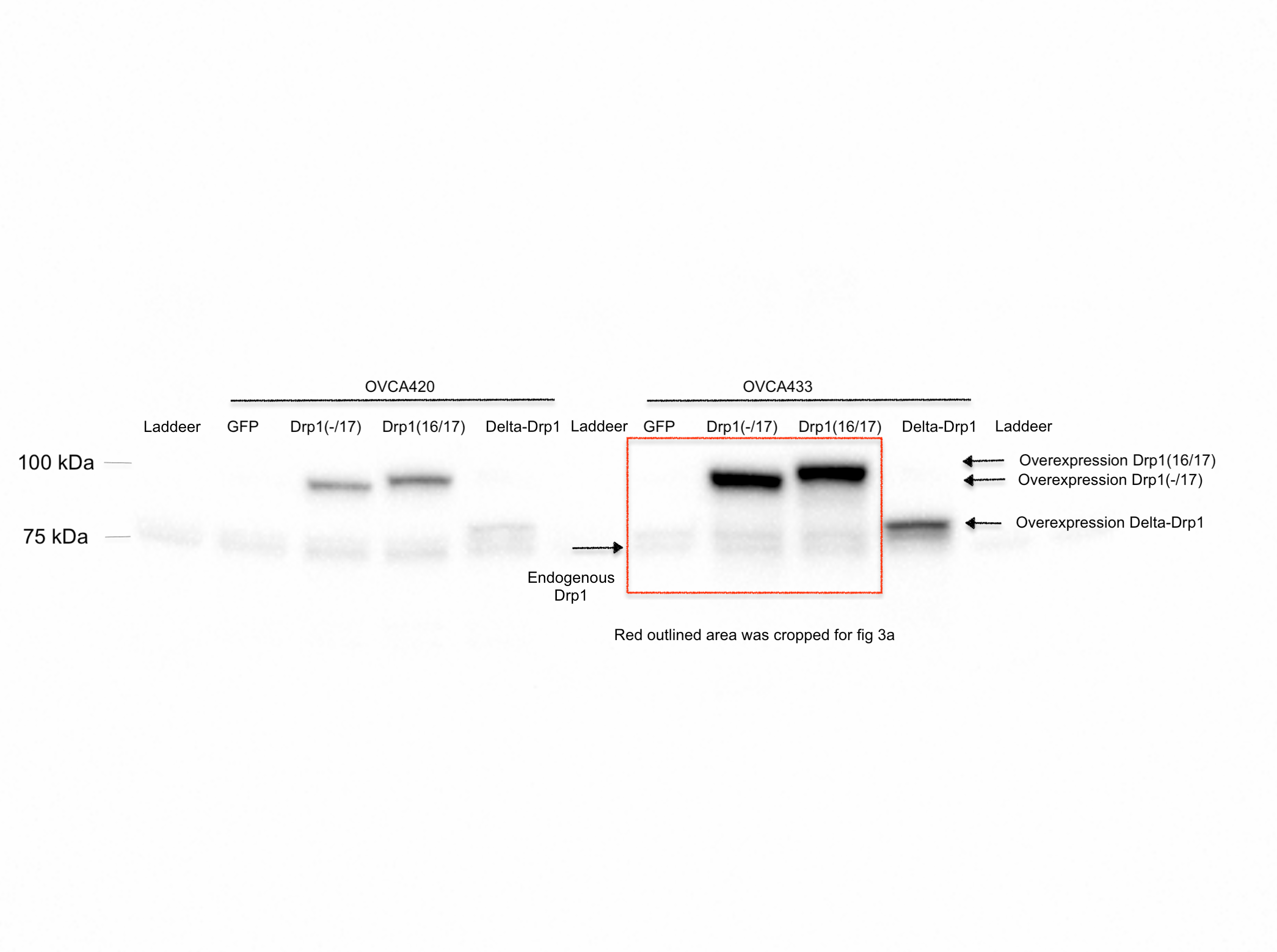

Supplement: Supplementary file 5 — Source data Fig. 3 [file 44319_2024_232_MOESM5_ESM.zip › Figure 3/3A/3A_western Drp1.tif]

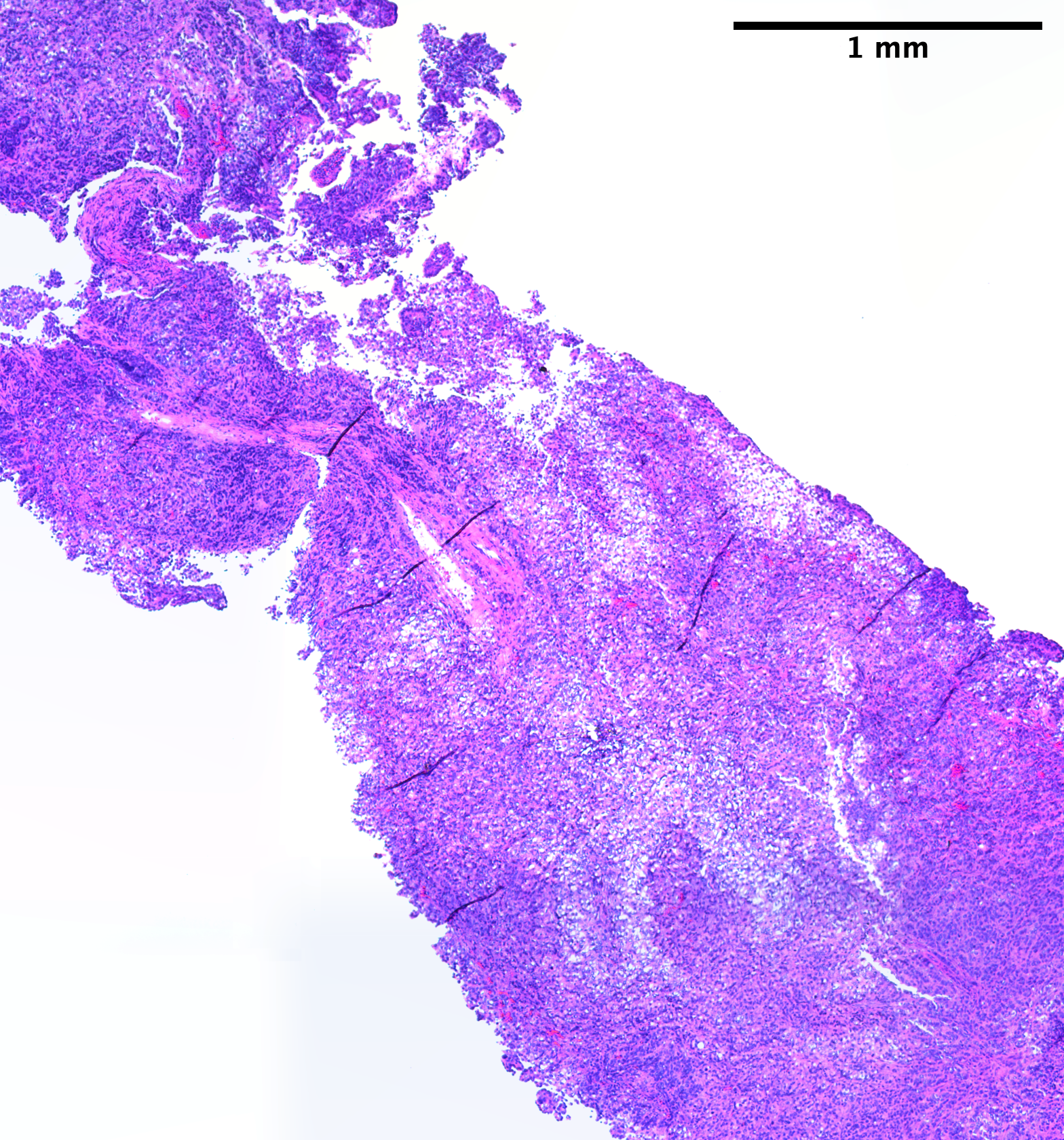

Supplement: Supplementary file 7 — Source data Fig. 5 [file 44319_2024_232_MOESM7_ESM.zip › Figure 5/5H/5H_iv_Drp1(1617)_Zoom1Omentum.png]

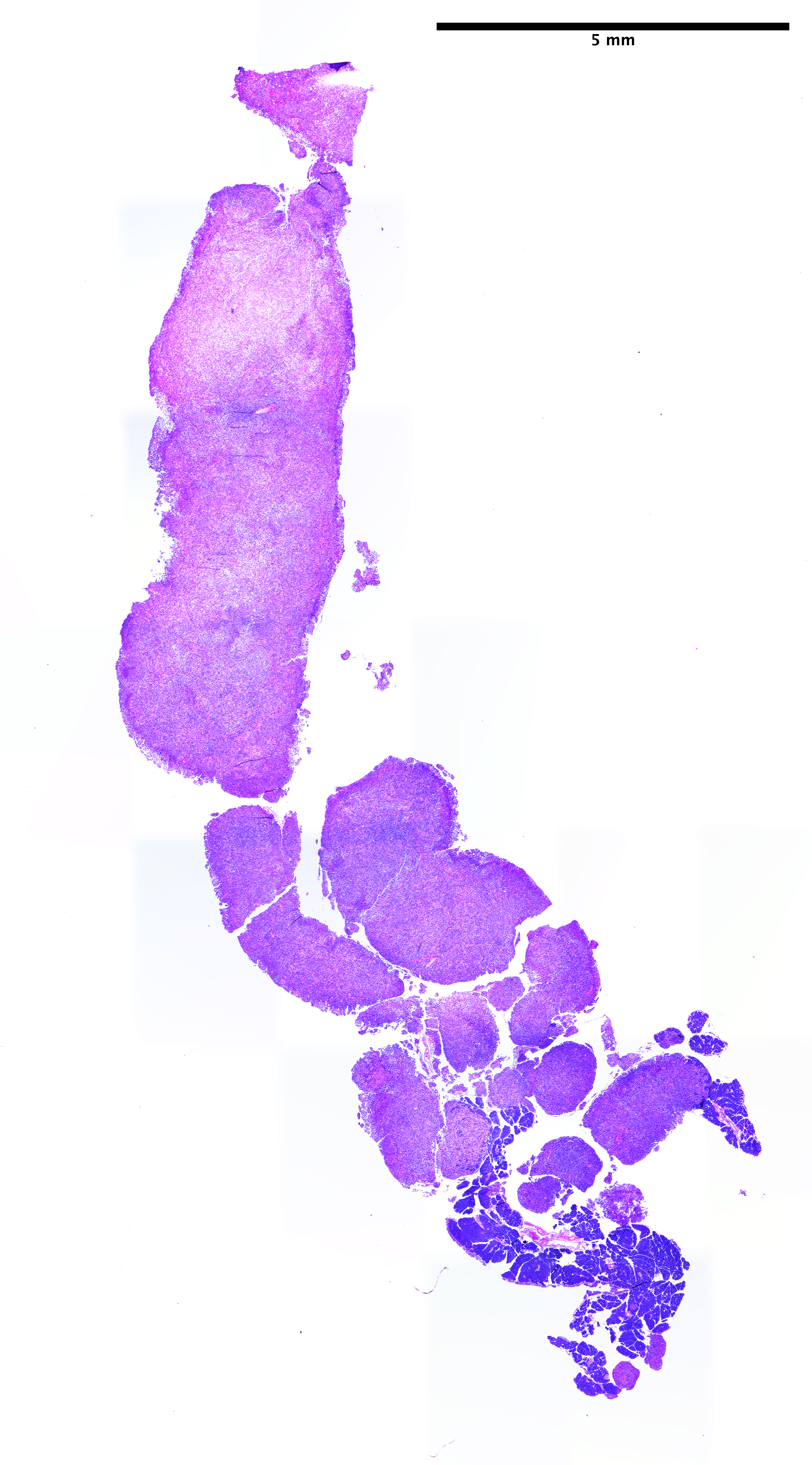

Supplement: Supplementary file 7 — Source data Fig. 5 [file 44319_2024_232_MOESM7_ESM.zip › Figure 5/5H/5H_iii_GFP_Omentum.tiff]

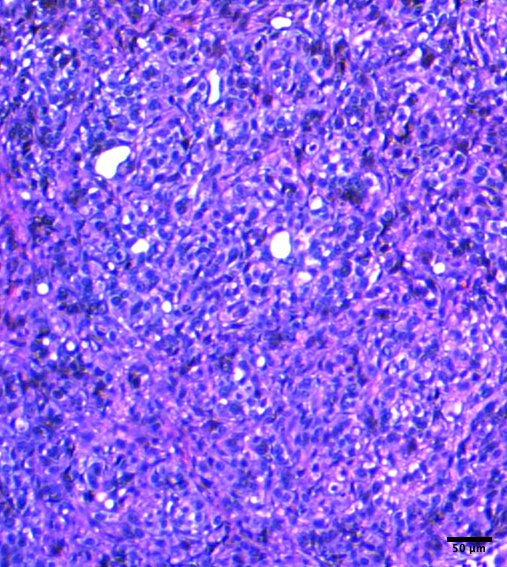

Supplement: Supplementary file 7 — Source data Fig. 5 [file 44319_2024_232_MOESM7_ESM.zip › Figure 5/5H/5H_v_Drp1(-17)_Zoom2Omentum.png]

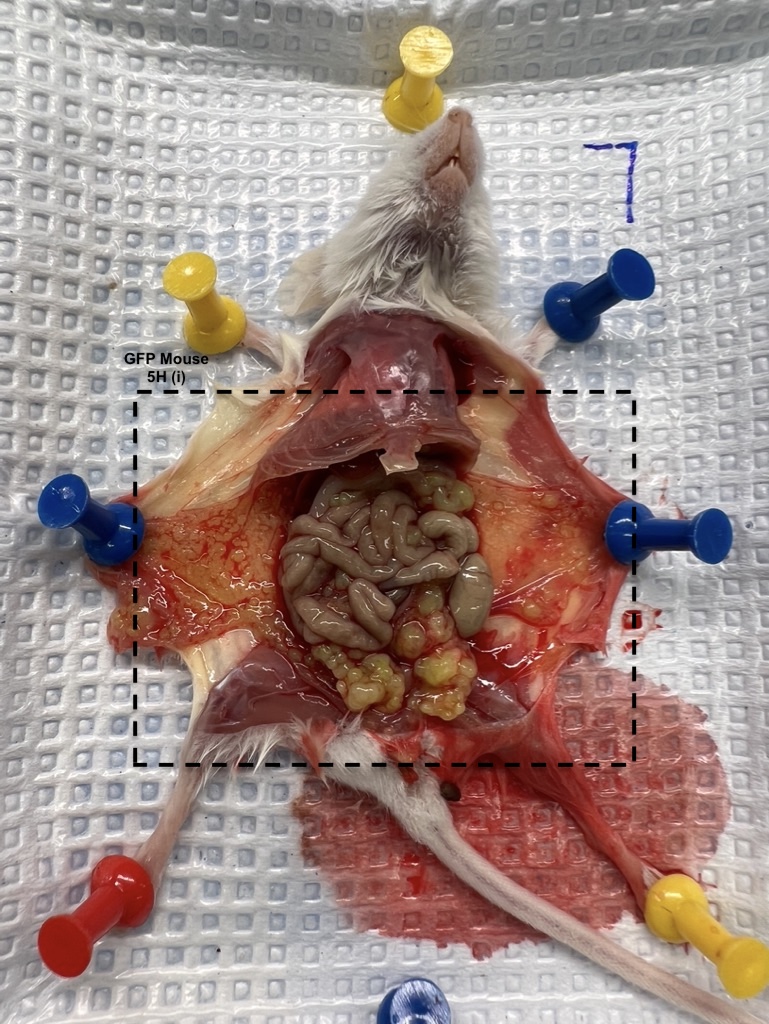

Supplement: Supplementary file 7 — Source data Fig. 5 [file 44319_2024_232_MOESM7_ESM.zip › Figure 5/5H/5H_i_GFP.jpeg]

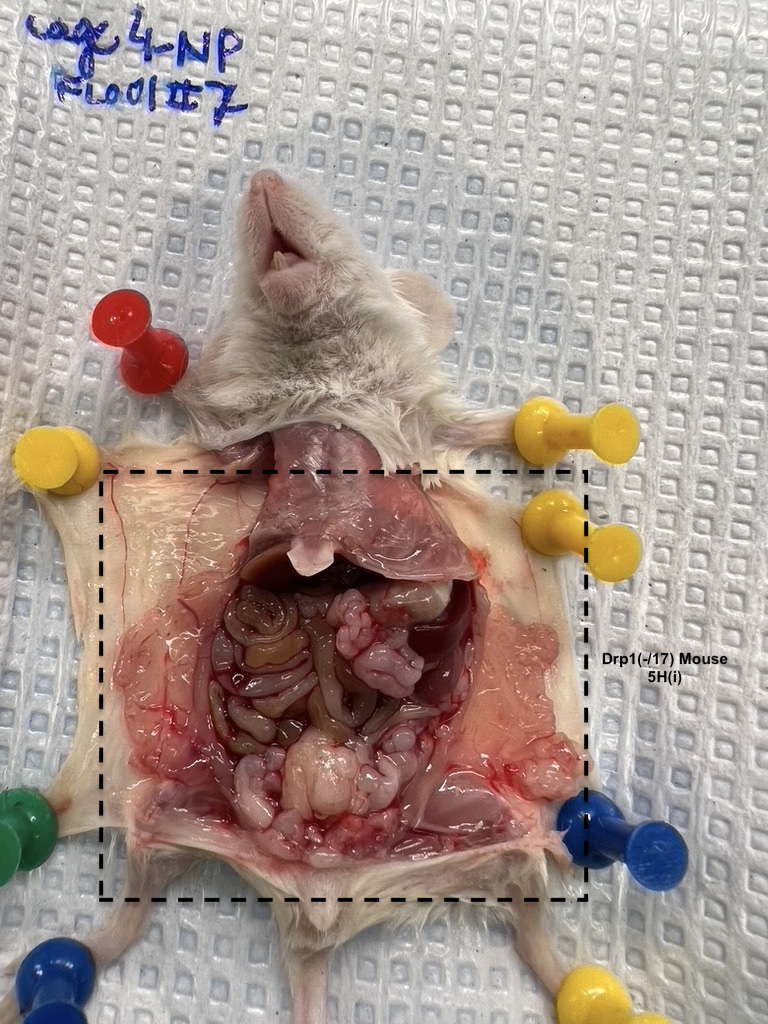

Supplement: Supplementary file 7 — Source data Fig. 5 [file 44319_2024_232_MOESM7_ESM.zip › Figure 5/5H/5H_i_Drp1(-17).jpeg]

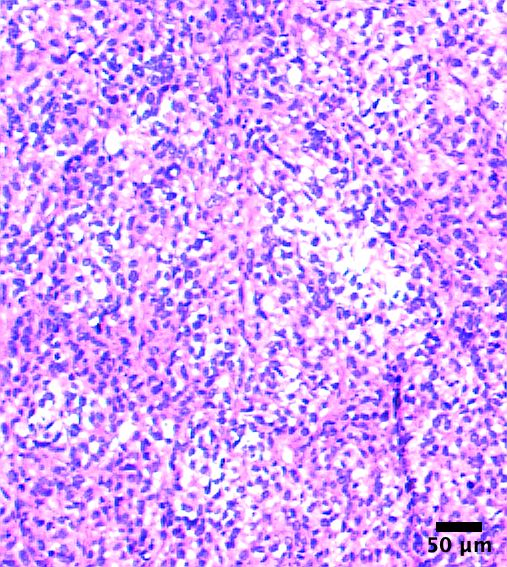

Supplement: Supplementary file 7 — Source data Fig. 5 [file 44319_2024_232_MOESM7_ESM.zip › Figure 5/5H/5H_v_GFP_Zoom2Omentum.png]

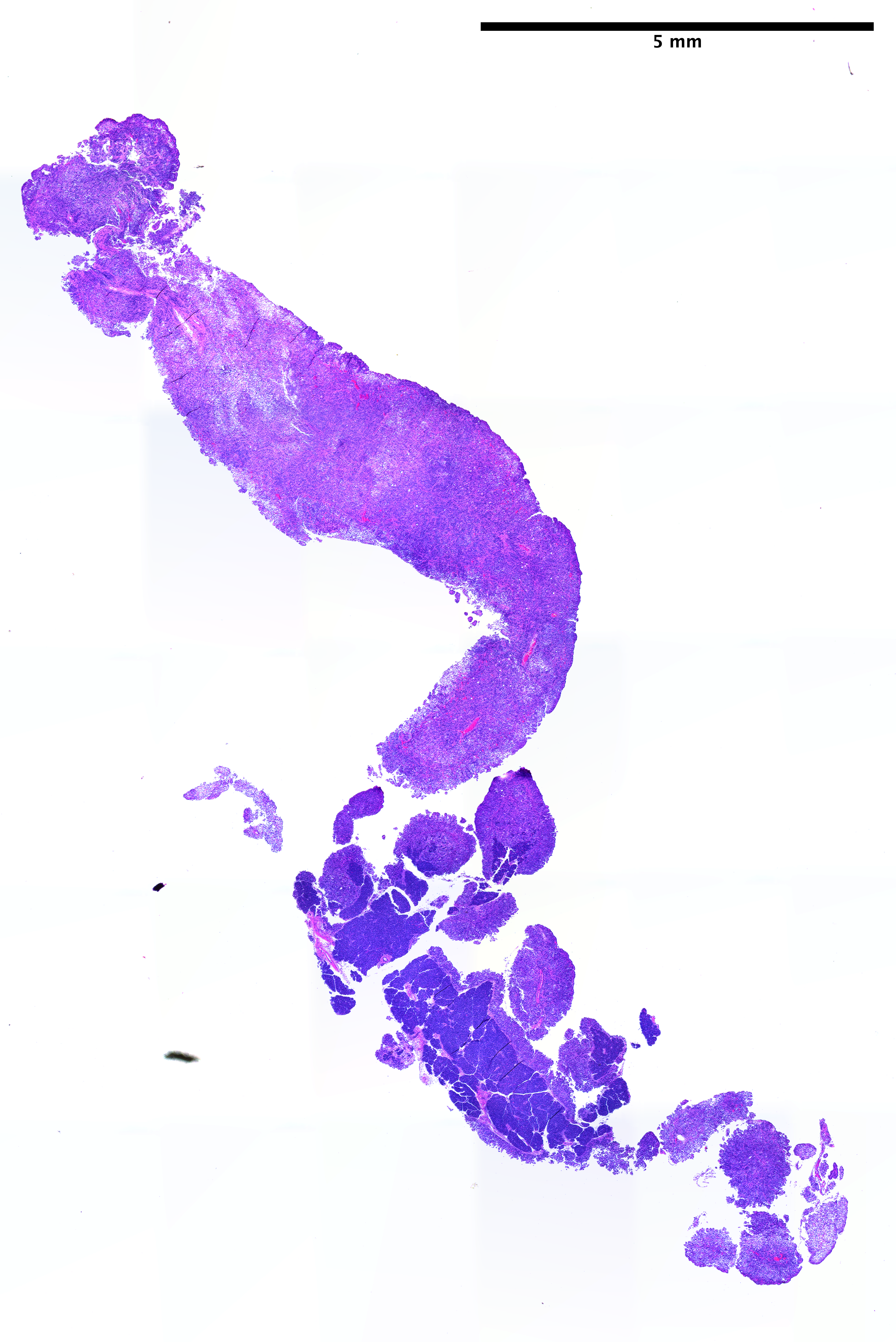

Supplement: Supplementary file 7 — Source data Fig. 5 [file 44319_2024_232_MOESM7_ESM.zip › Figure 5/5H/5H_iii_Drp1(1617)_Omentum.tiff]

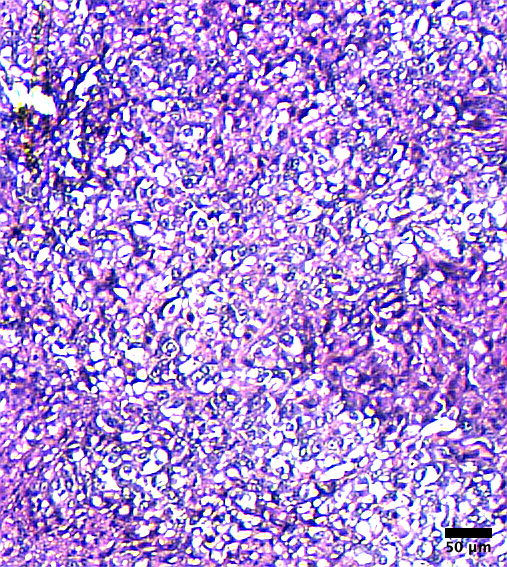

Supplement: Supplementary file 7 — Source data Fig. 5 [file 44319_2024_232_MOESM7_ESM.zip › Figure 5/5H/5H_v_Drp1(1617)_Zoom2Omentum.png]

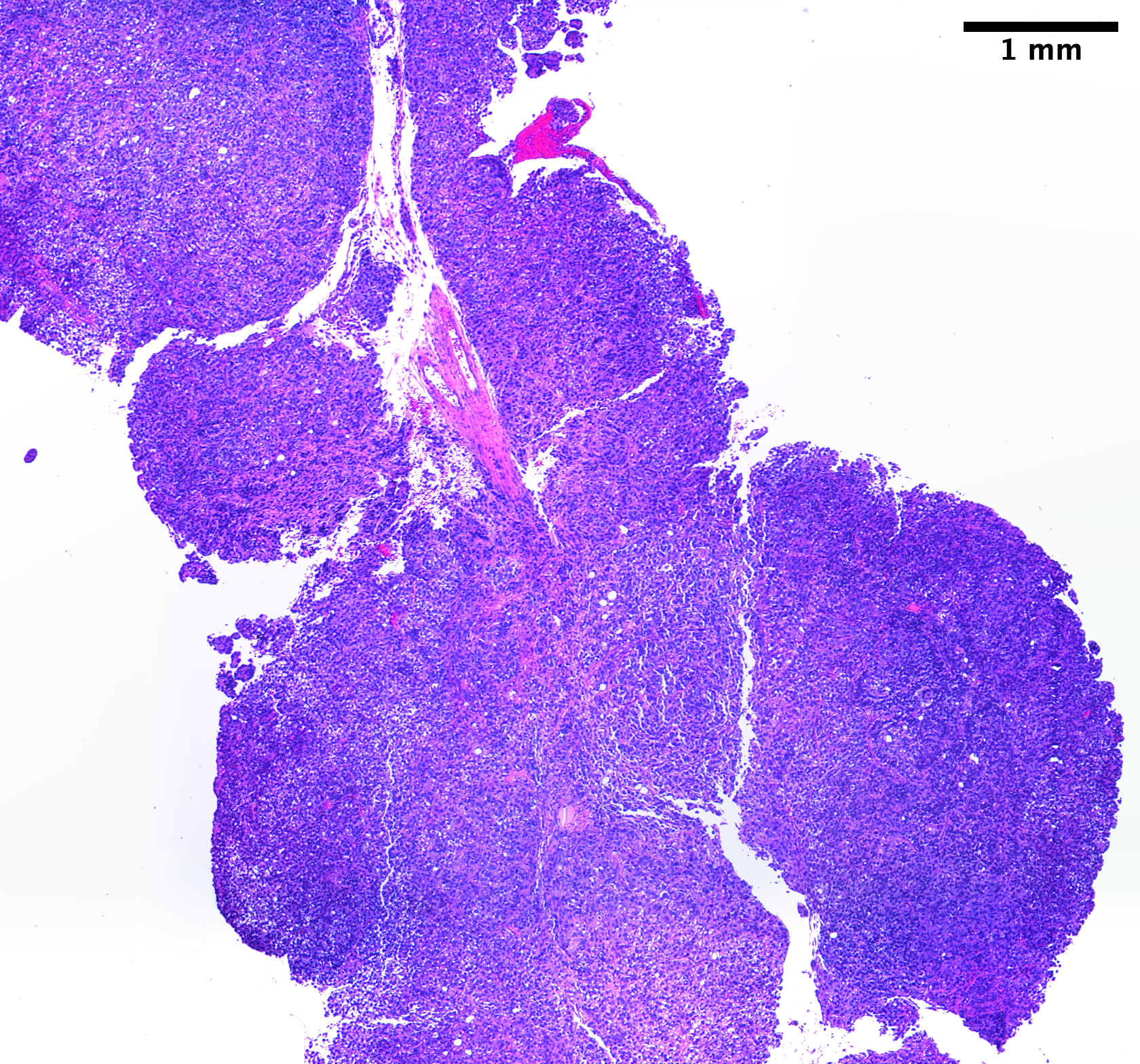

Supplement: Supplementary file 7 — Source data Fig. 5 [file 44319_2024_232_MOESM7_ESM.zip › Figure 5/5H/5H_iv_Drp1(-17)_Zoom1Omentum.png]

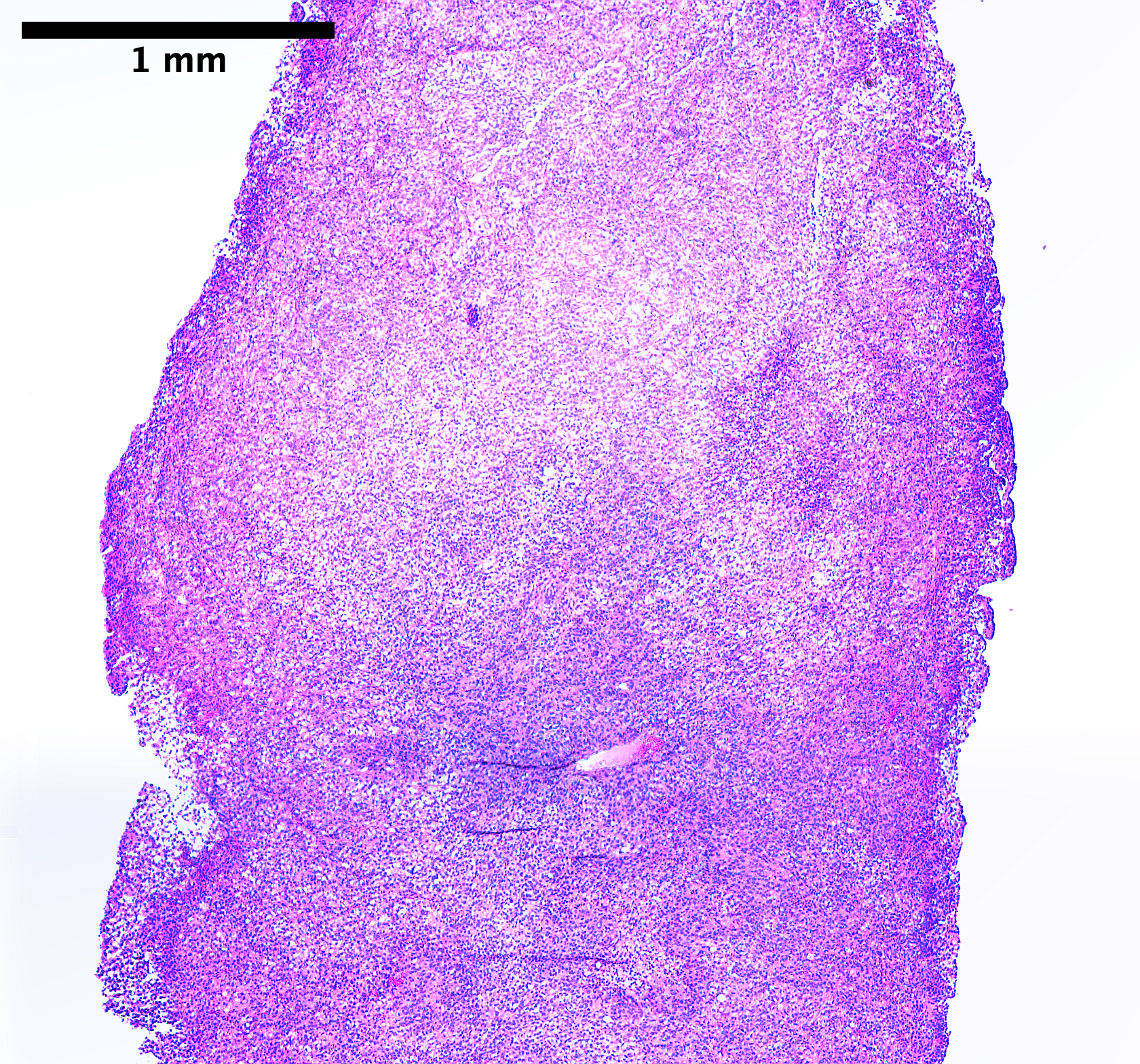

Supplement: Supplementary file 7 — Source data Fig. 5 [file 44319_2024_232_MOESM7_ESM.zip › Figure 5/5H/5H_iv_GFP_Zoom1Omentum.png]

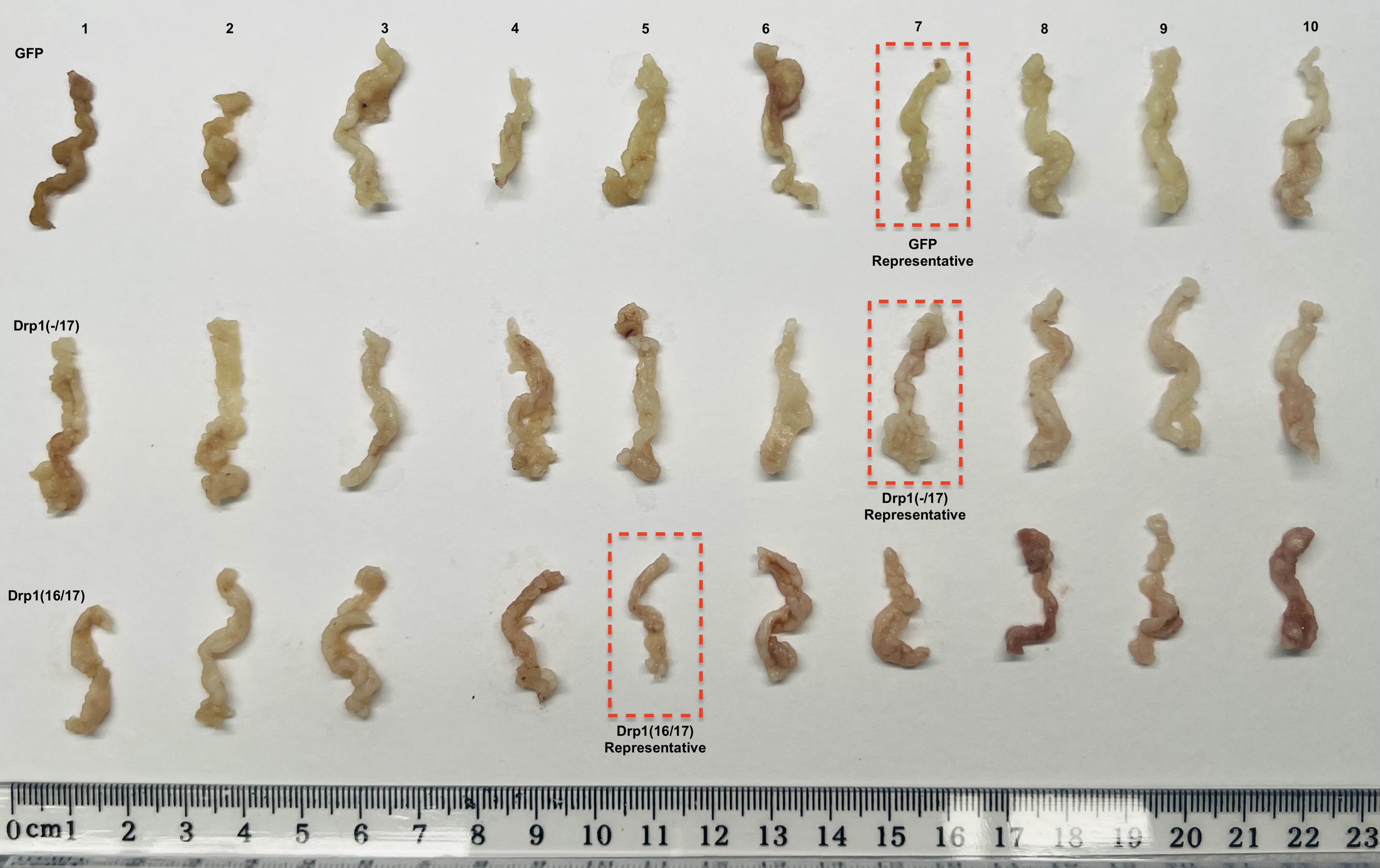

Supplement: Supplementary file 7 — Source data Fig. 5 [file 44319_2024_232_MOESM7_ESM.zip › Figure 5/5H/5H_ii_GFP_Drp1(-17)_Drp1(1617)_OmentalTissue.jpeg]

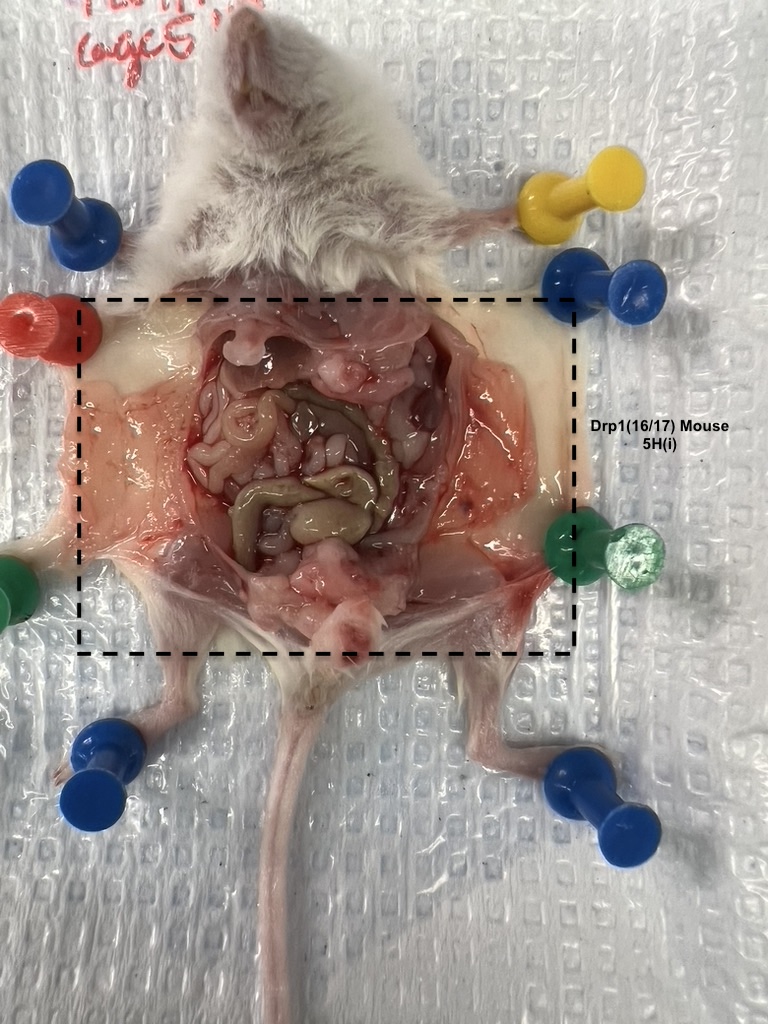

Supplement: Supplementary file 7 — Source data Fig. 5 [file 44319_2024_232_MOESM7_ESM.zip › Figure 5/5H/5H_i_Drp1(1617).jpeg]

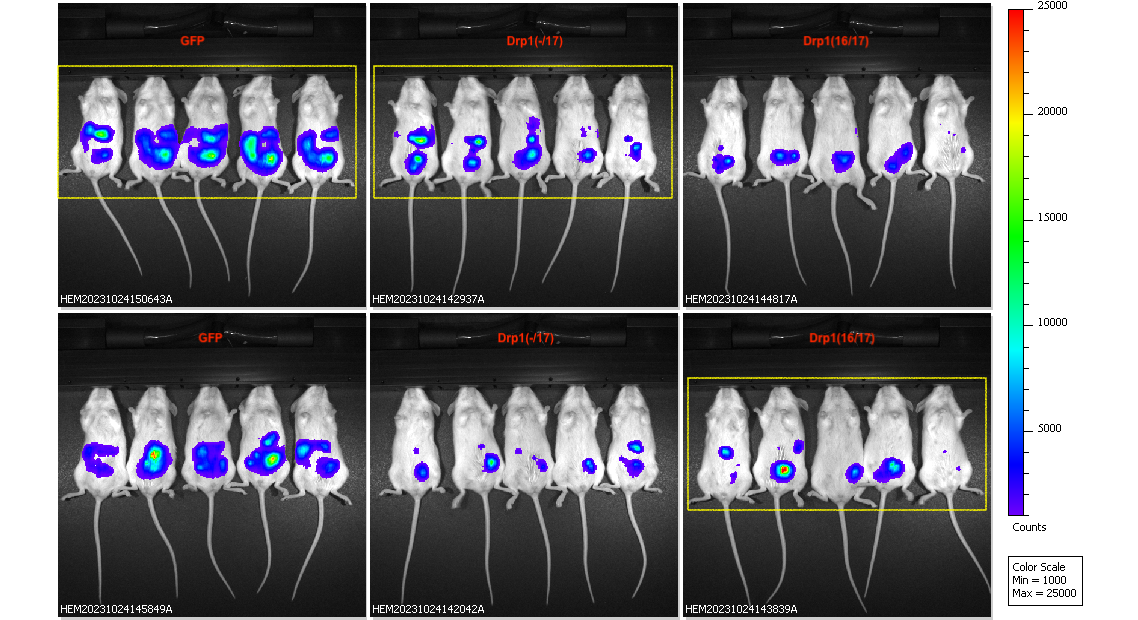

Supplement: Supplementary file 7 — Source data Fig. 5 [file 44319_2024_232_MOESM7_ESM.zip › Figure 5/5D/5D_Day20_MouseLuciferase.png]

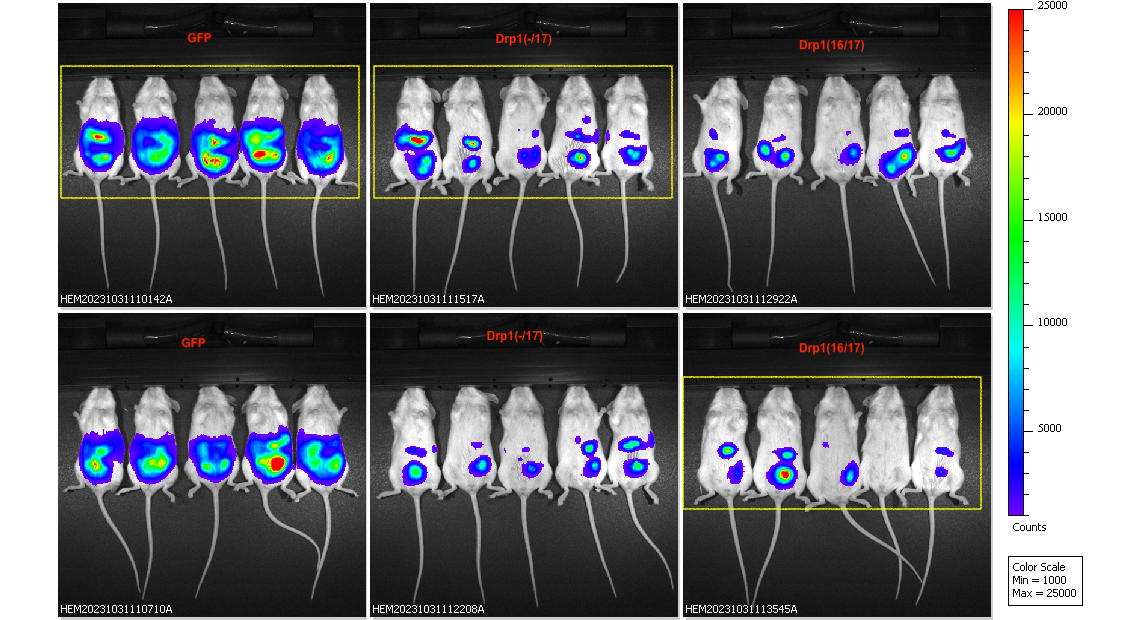

Supplement: Supplementary file 7 — Source data Fig. 5 [file 44319_2024_232_MOESM7_ESM.zip › Figure 5/5D/5D_Day27_MouseLuciferase.png]

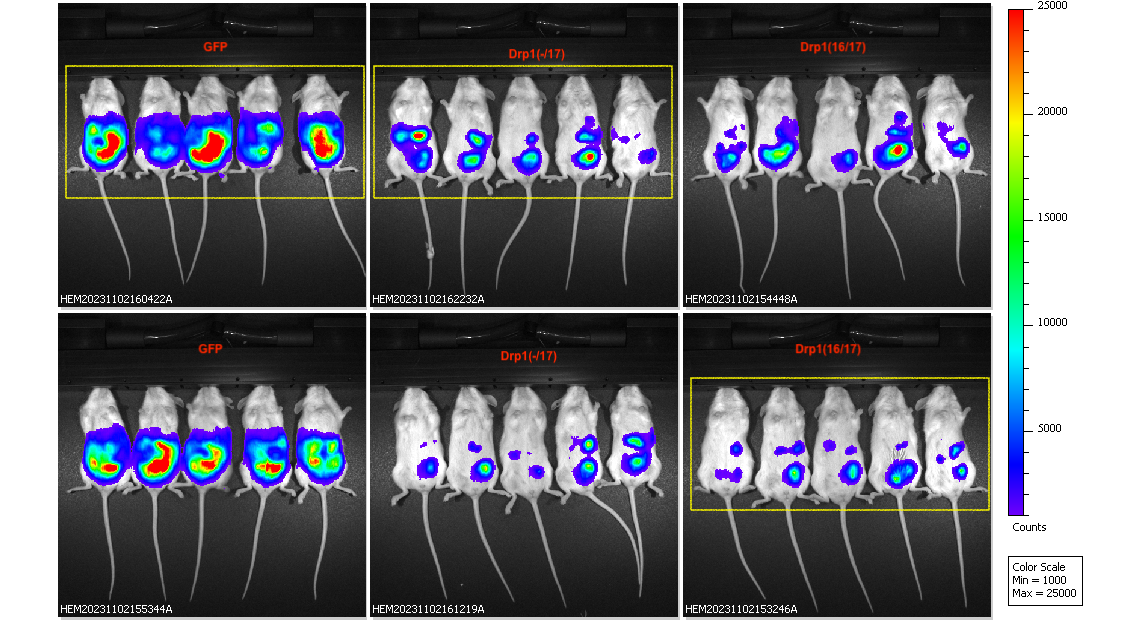

Supplement: Supplementary file 7 — Source data Fig. 5 [file 44319_2024_232_MOESM7_ESM.zip › Figure 5/5D/5D_Day30_MouseLuciferase.png]

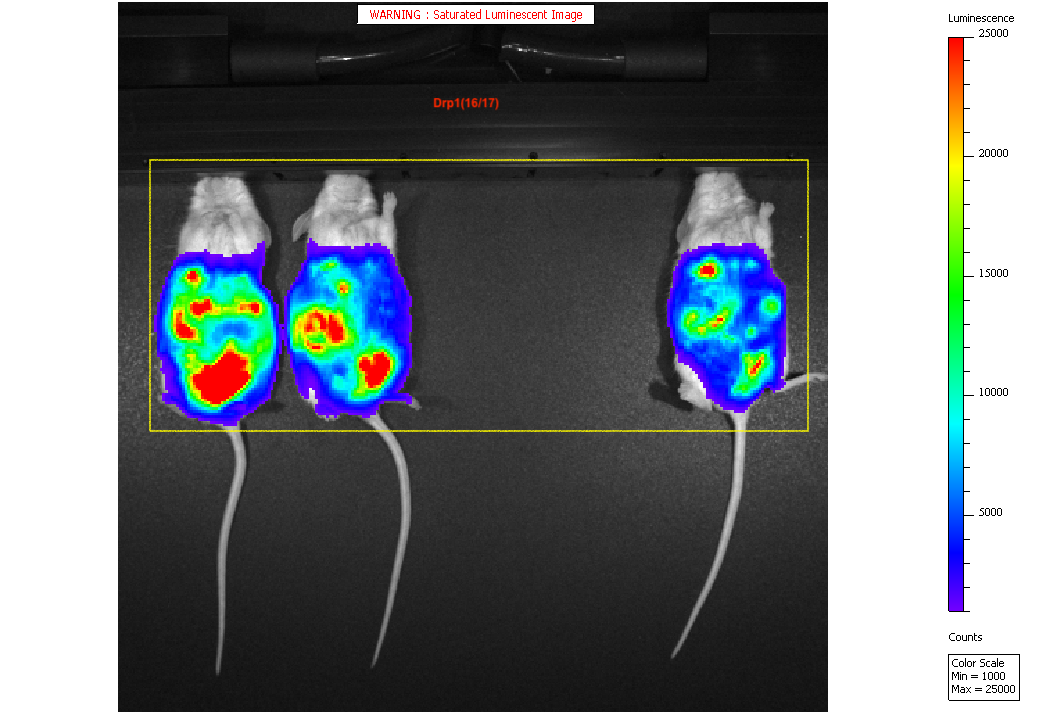

Supplement: Supplementary file 7 — Source data Fig. 5 [file 44319_2024_232_MOESM7_ESM.zip › Figure 5/5D/5D_Day51_MouseLuciferase.png]

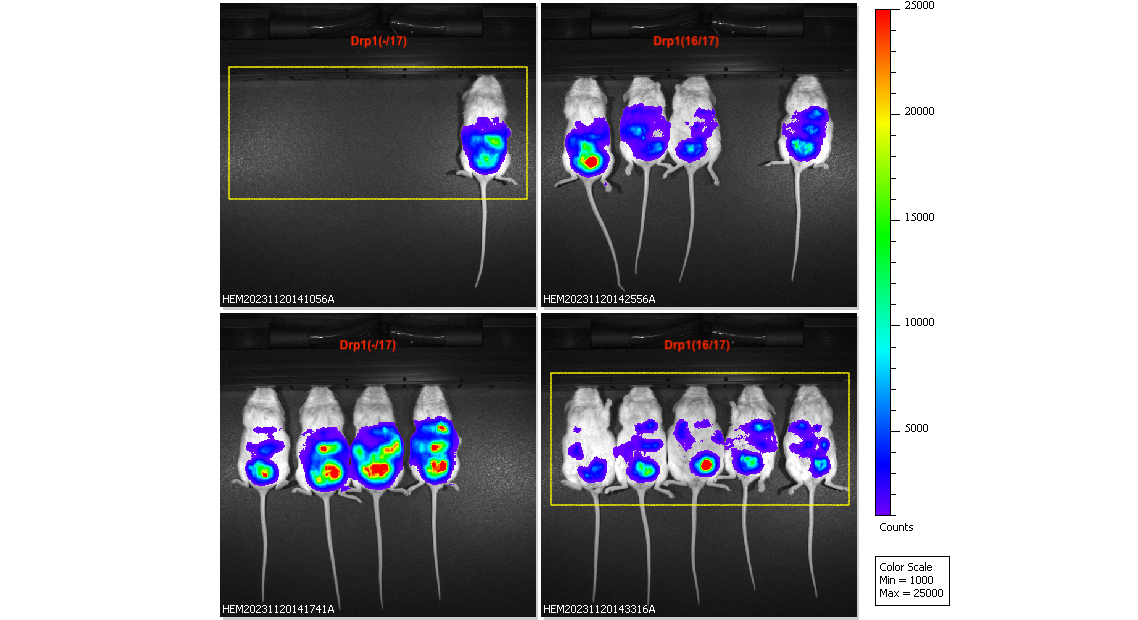

Supplement: Supplementary file 7 — Source data Fig. 5 [file 44319_2024_232_MOESM7_ESM.zip › Figure 5/5D/5D_Day47_MouseLuciferase.png]

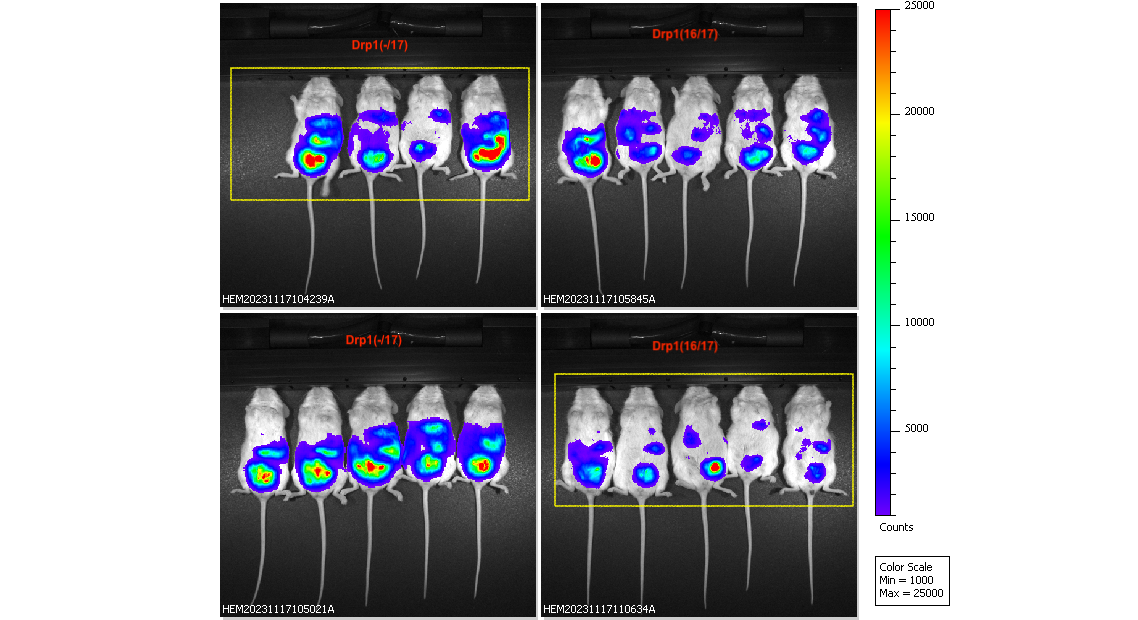

Supplement: Supplementary file 7 — Source data Fig. 5 [file 44319_2024_232_MOESM7_ESM.zip › Figure 5/5D/5D_Day44_MouseLuciferase.png]

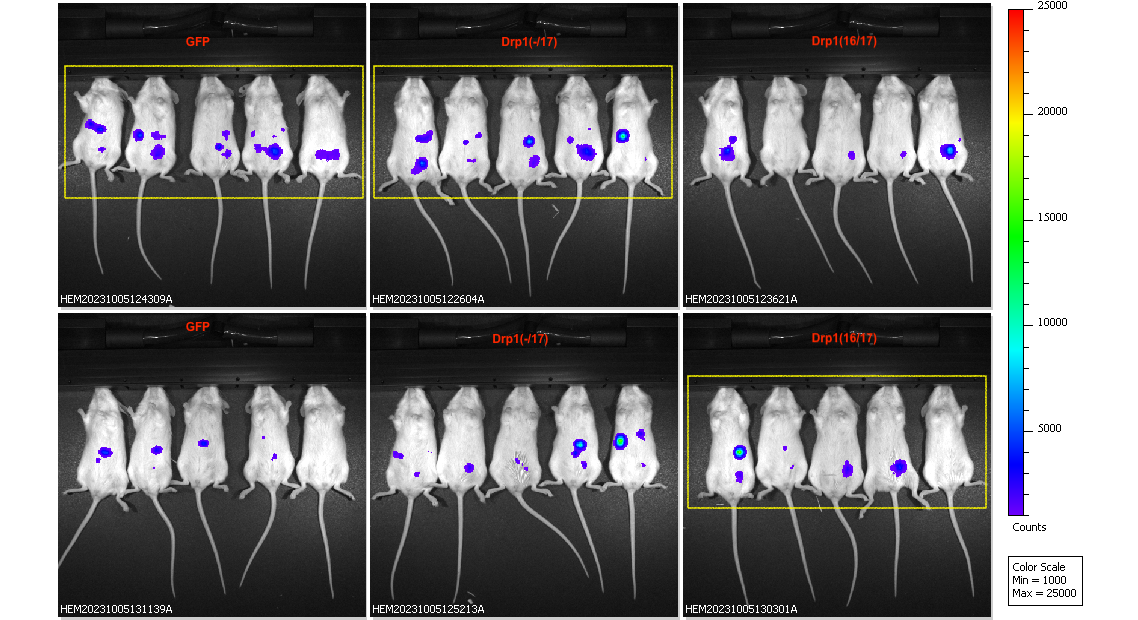

Supplement: Supplementary file 7 — Source data Fig. 5 [file 44319_2024_232_MOESM7_ESM.zip › Figure 5/5D/5D_Day1_MouseLuciferase.png]

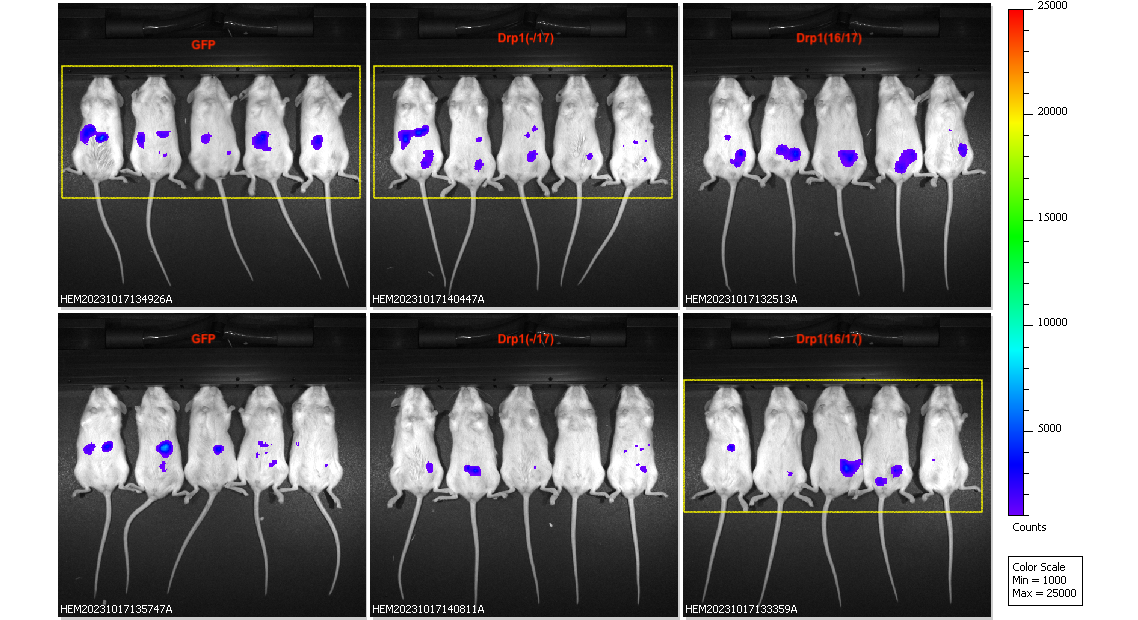

Supplement: Supplementary file 7 — Source data Fig. 5 [file 44319_2024_232_MOESM7_ESM.zip › Figure 5/5D/5D_Day13_MouseLuciferase.png]

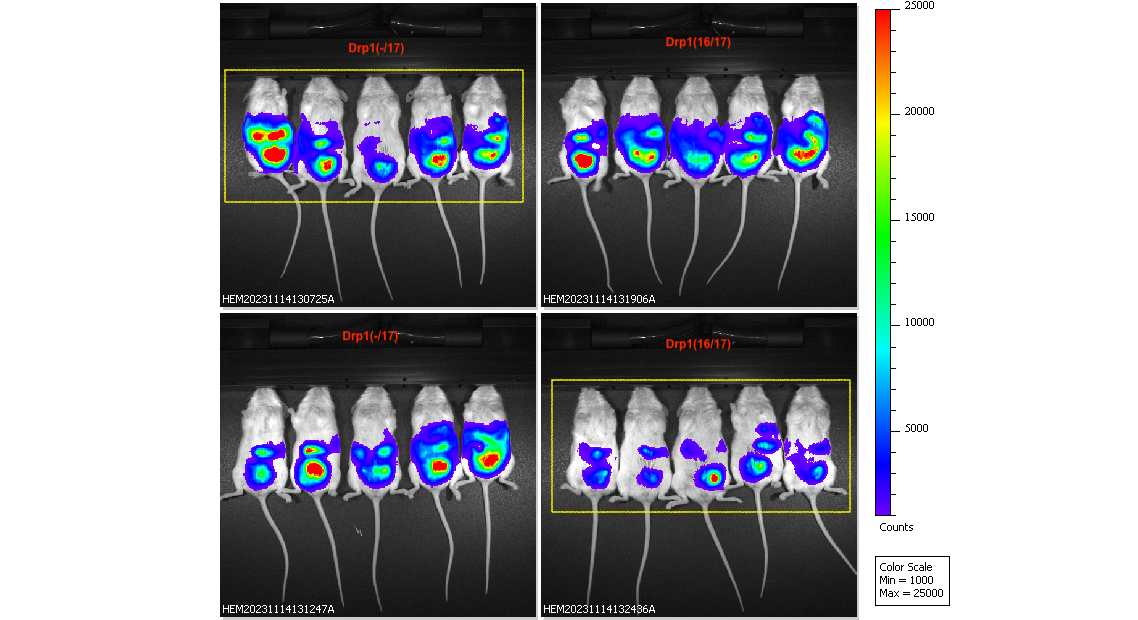

Supplement: Supplementary file 7 — Source data Fig. 5 [file 44319_2024_232_MOESM7_ESM.zip › Figure 5/5D/5D_Day41_MouseLuciferase.png]

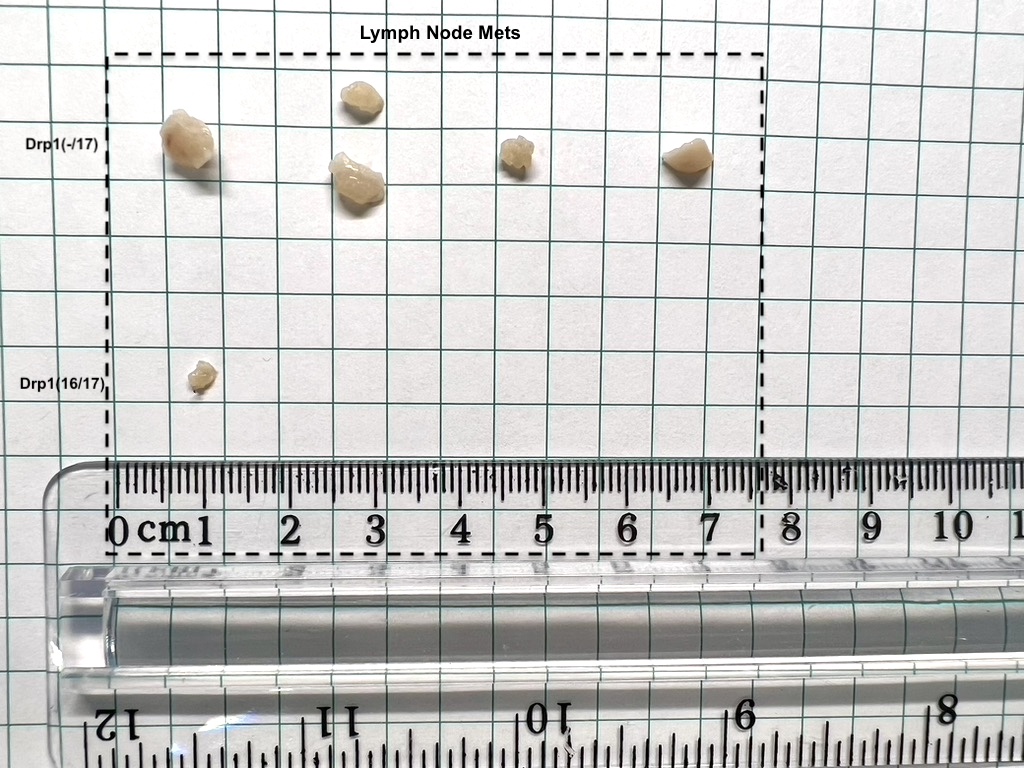

Supplement: Supplementary file 8 — Source data Fig. 6 [file 44319_2024_232_MOESM8_ESM.zip › Figure 6/6E/6E_XengraftStudy_Lymphnodemets_Salinegroup.jpeg]

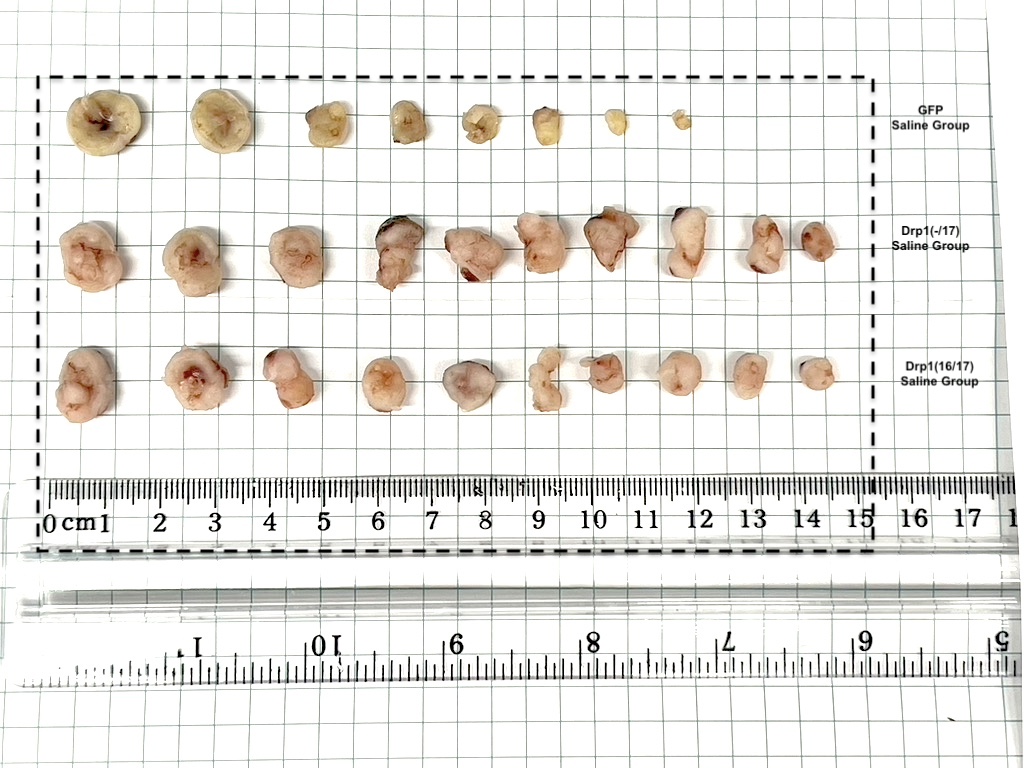

Supplement: Supplementary file 8 — Source data Fig. 6 [file 44319_2024_232_MOESM8_ESM.zip › Figure 6/6D/6D_SKOV3_Xenograft tumor_untreated.jpeg]

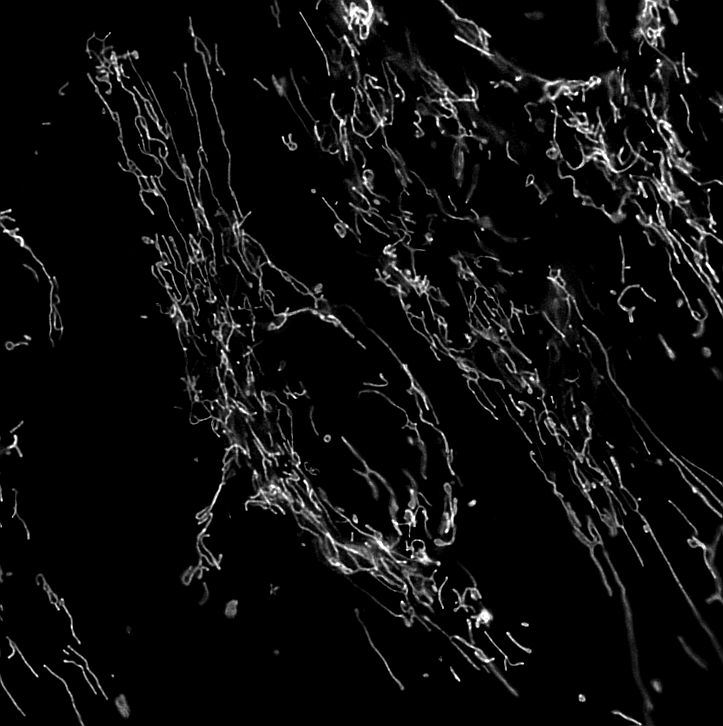

Supplement: Supplementary file 9 — Source data Fig. 7 [file 44319_2024_232_MOESM9_ESM.zip › Figure 7/7C/7C_skov3_siDrp1(1617)_mitotrackeronly.tif]

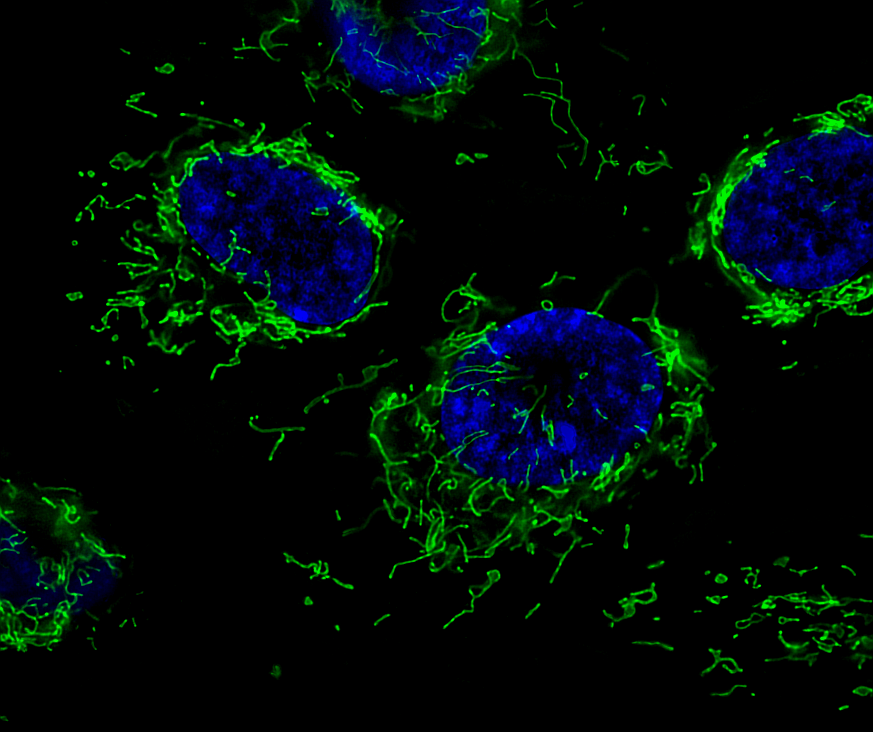

Supplement: Supplementary file 9 — Source data Fig. 7 [file 44319_2024_232_MOESM9_ESM.zip › Figure 7/7C/7C_skov3_siDrp1(--)and(16-)_SelectedArea.tif]

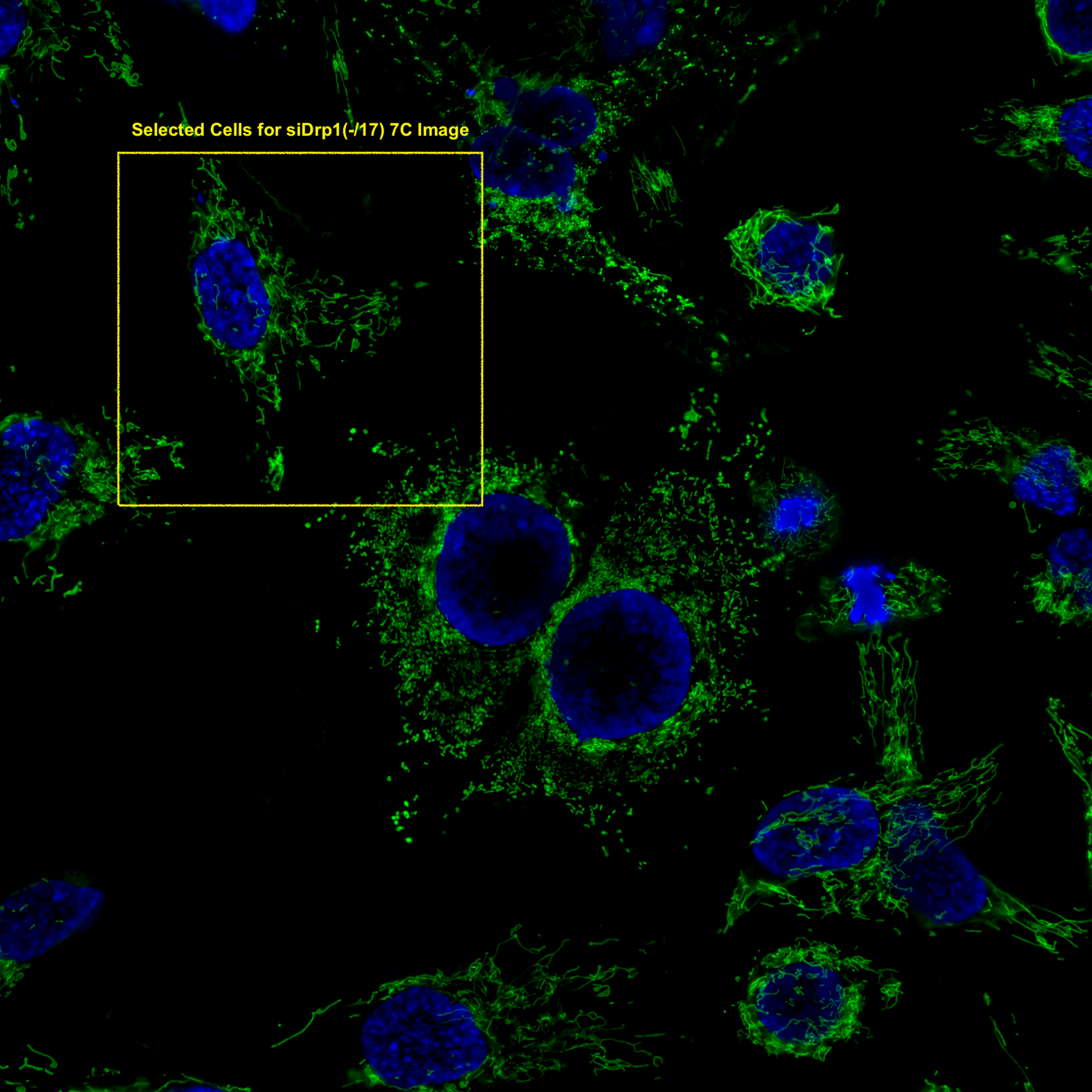

Supplement: Supplementary file 9 — Source data Fig. 7 [file 44319_2024_232_MOESM9_ESM.zip › Figure 7/7C/7C_skov3_siDrp1(-17)_Orginal.tif]

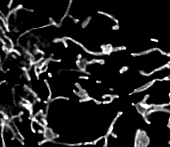

Supplement: Supplementary file 9 — Source data Fig. 7 [file 44319_2024_232_MOESM9_ESM.zip › Figure 7/7C/7C_skov3_siDrp1(-17)_zoomed.tif]

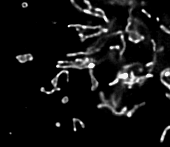

Supplement: Supplementary file 9 — Source data Fig. 7 [file 44319_2024_232_MOESM9_ESM.zip › Figure 7/7C/7C_skov3_siDrp1(--)and(16-)_zoomed.tif]

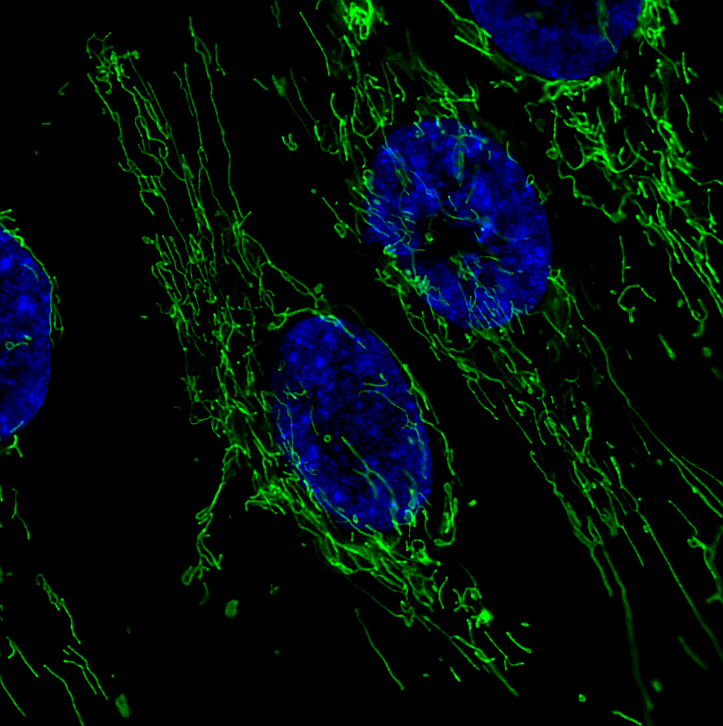

Supplement: Supplementary file 9 — Source data Fig. 7 [file 44319_2024_232_MOESM9_ESM.zip › Figure 7/7C/7C_skov3_siDrp1(1617)_SelectedArea.tif]

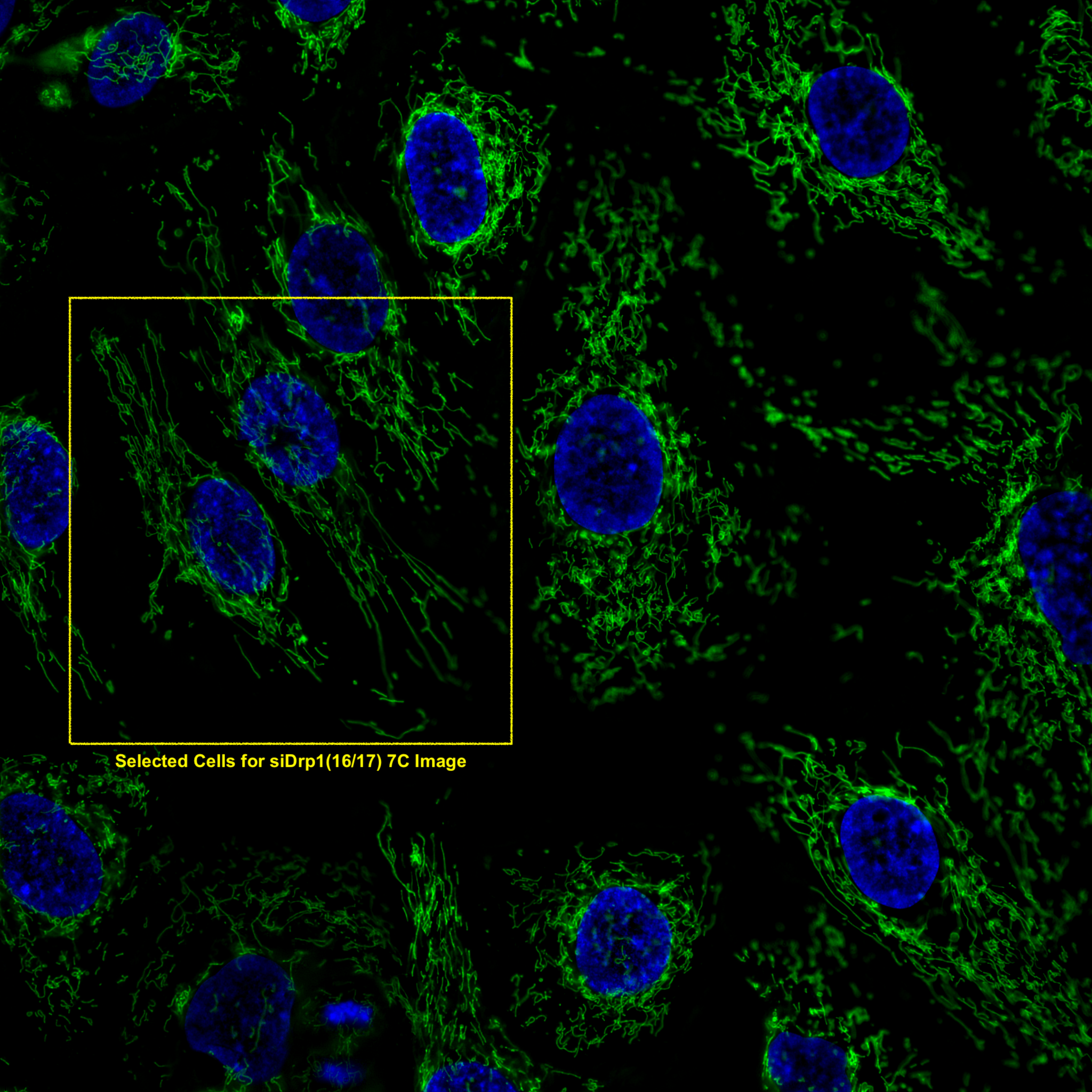

Supplement: Supplementary file 9 — Source data Fig. 7 [file 44319_2024_232_MOESM9_ESM.zip › Figure 7/7C/7C_skov3_siDrp1(1617)_Orginal.tif]

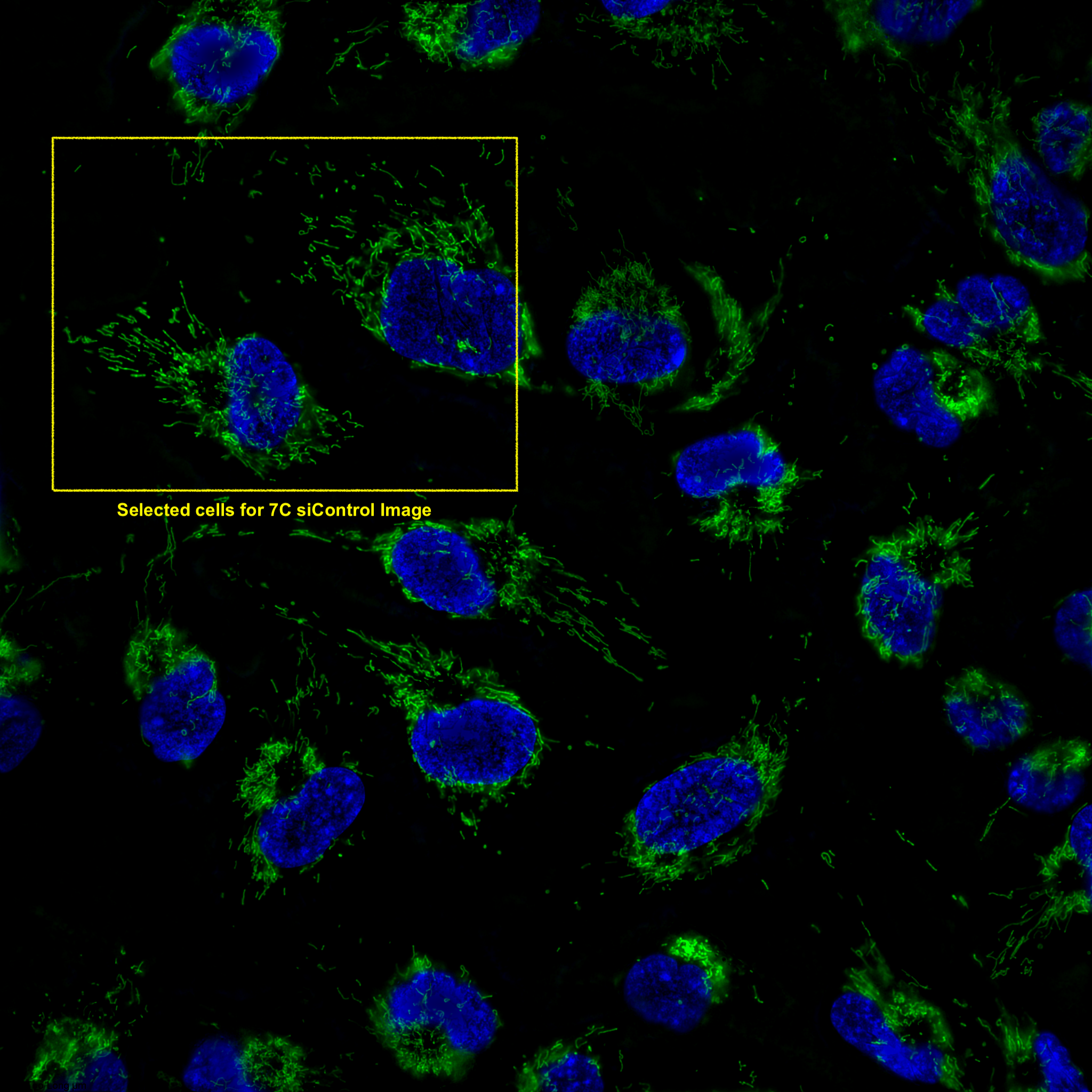

Supplement: Supplementary file 9 — Source data Fig. 7 [file 44319_2024_232_MOESM9_ESM.zip › Figure 7/7C/7C_siControl_Orginal.tif]

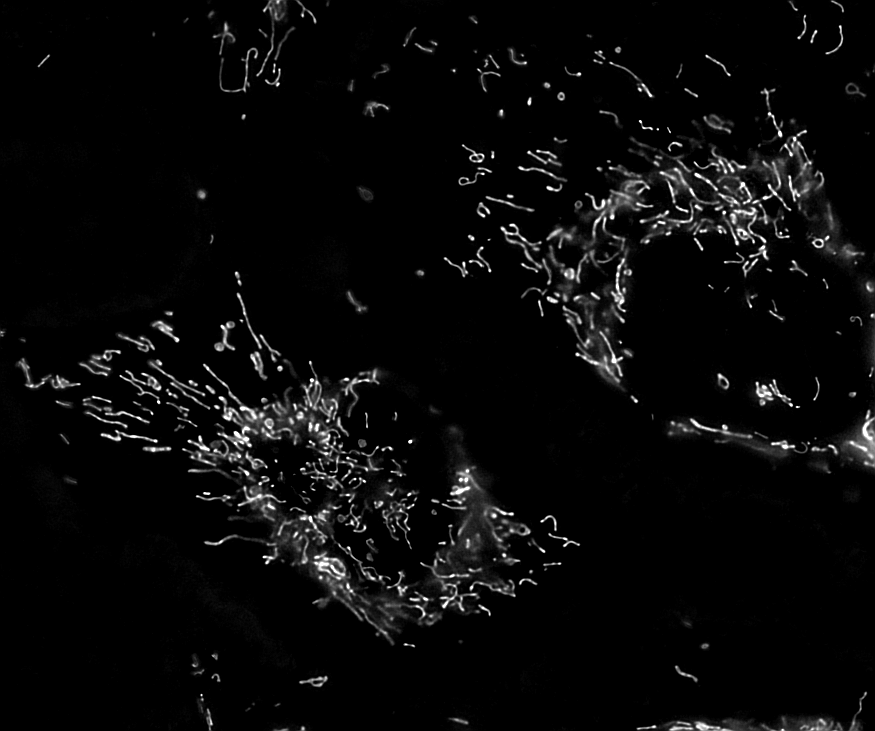

Supplement: Supplementary file 9 — Source data Fig. 7 [file 44319_2024_232_MOESM9_ESM.zip › Figure 7/7C/7C_skov3_siControl_mitotrackeronly.tif]

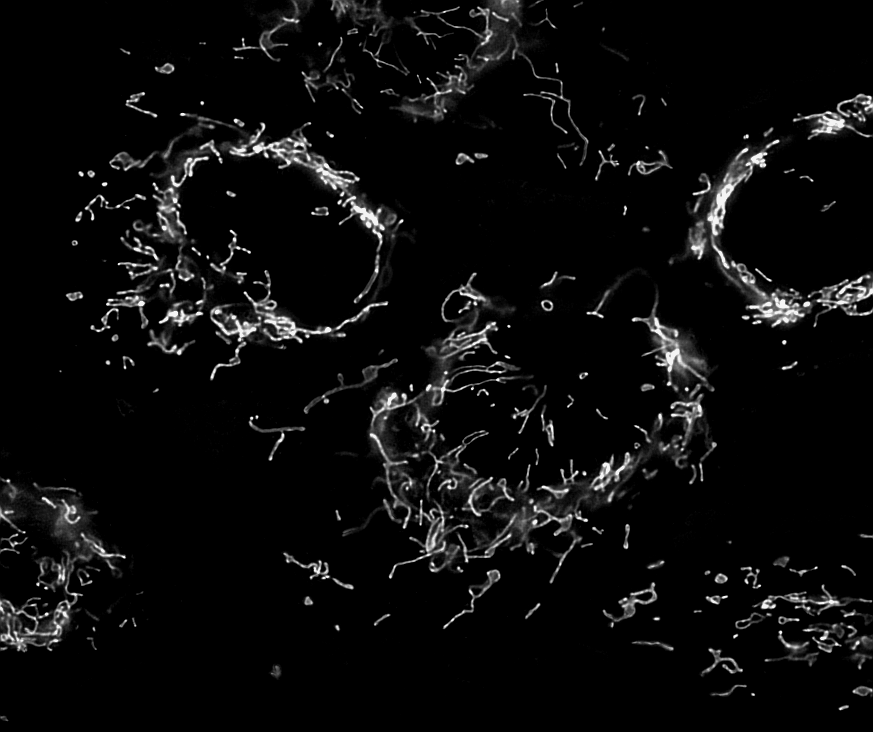

Supplement: Supplementary file 9 — Source data Fig. 7 [file 44319_2024_232_MOESM9_ESM.zip › Figure 7/7C/7C_skov3_siDrp1(--)and(16-)_mitotrackeronly.tif]

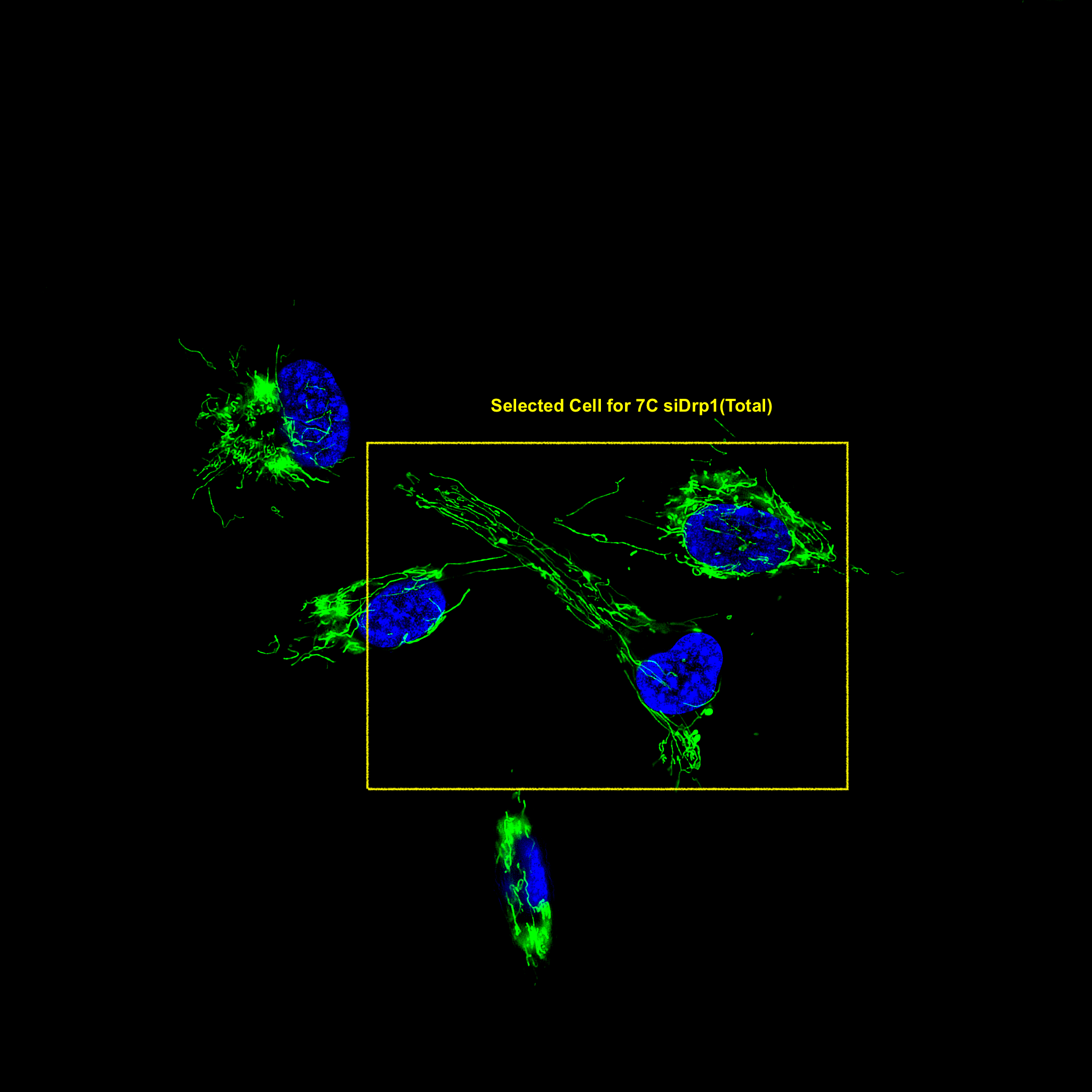

Supplement: Supplementary file 9 — Source data Fig. 7 [file 44319_2024_232_MOESM9_ESM.zip › Figure 7/7C/7C_skov3_siDrp1(total)_Original.tif]

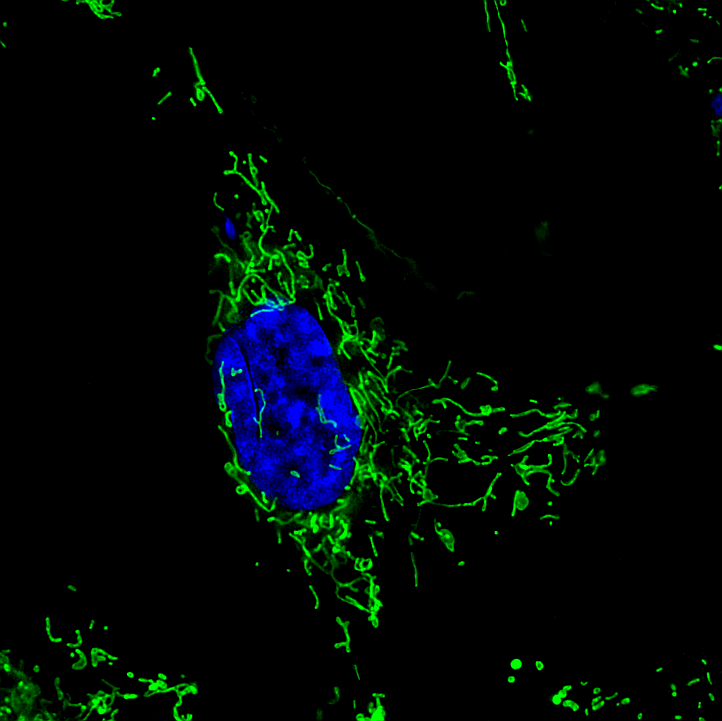

Supplement: Supplementary file 9 — Source data Fig. 7 [file 44319_2024_232_MOESM9_ESM.zip › Figure 7/7C/7C_skov3_siDrp1(-17)_SelectedArea.tif]

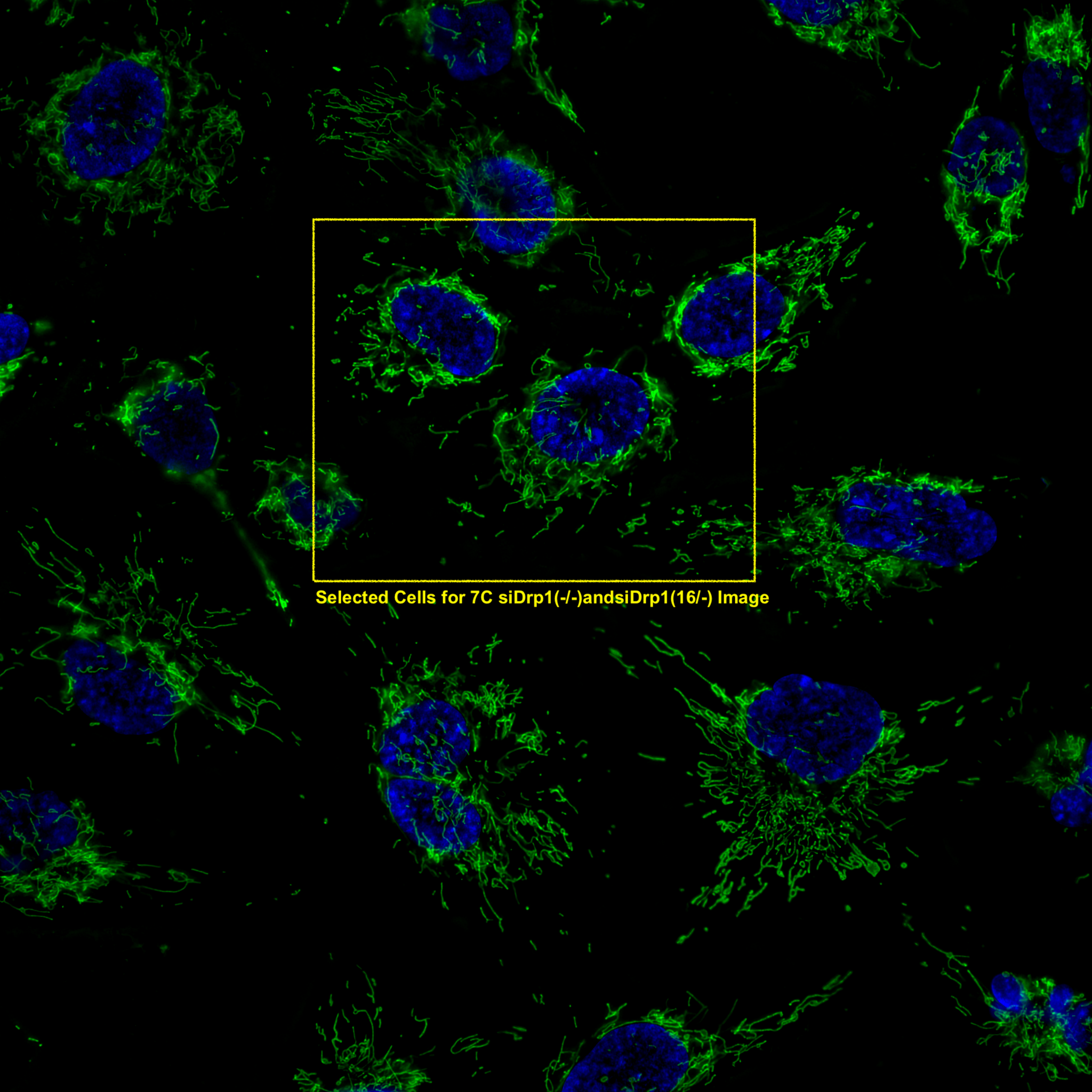

Supplement: Supplementary file 9 — Source data Fig. 7 [file 44319_2024_232_MOESM9_ESM.zip › Figure 7/7C/7C_skov3_siDrp1(--)and(16-)_Orginal.tif]

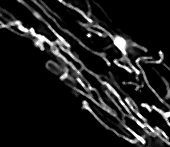

Supplement: Supplementary file 9 — Source data Fig. 7 [file 44319_2024_232_MOESM9_ESM.zip › Figure 7/7C/7C_skov3_siDrp1(Total)_zoomed.tif]

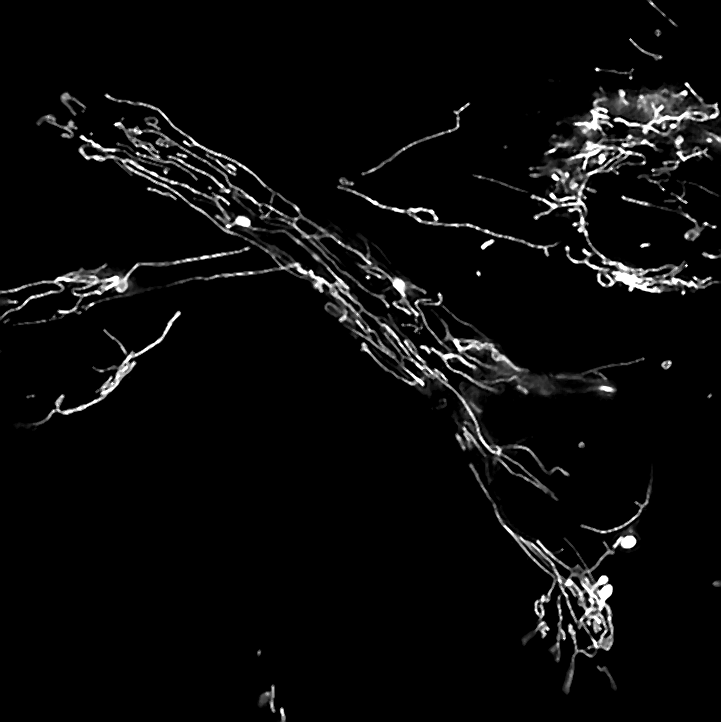

Supplement: Supplementary file 9 — Source data Fig. 7 [file 44319_2024_232_MOESM9_ESM.zip › Figure 7/7C/7C_skov3_siDrp1(Total)_mitotrackeronly.tif]

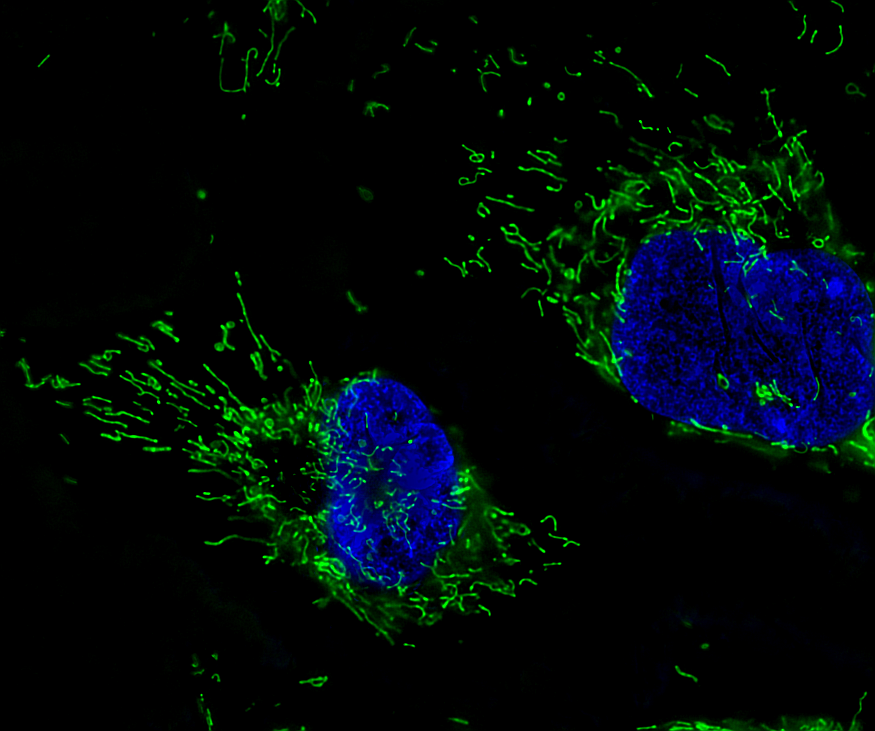

Supplement: Supplementary file 9 — Source data Fig. 7 [file 44319_2024_232_MOESM9_ESM.zip › Figure 7/7C/7C_skov3_siControl_SelectedArea.tif]

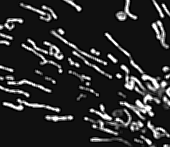

Supplement: Supplementary file 9 — Source data Fig. 7 [file 44319_2024_232_MOESM9_ESM.zip › Figure 7/7C/7C_skov3_siControl_zoomed.tif]

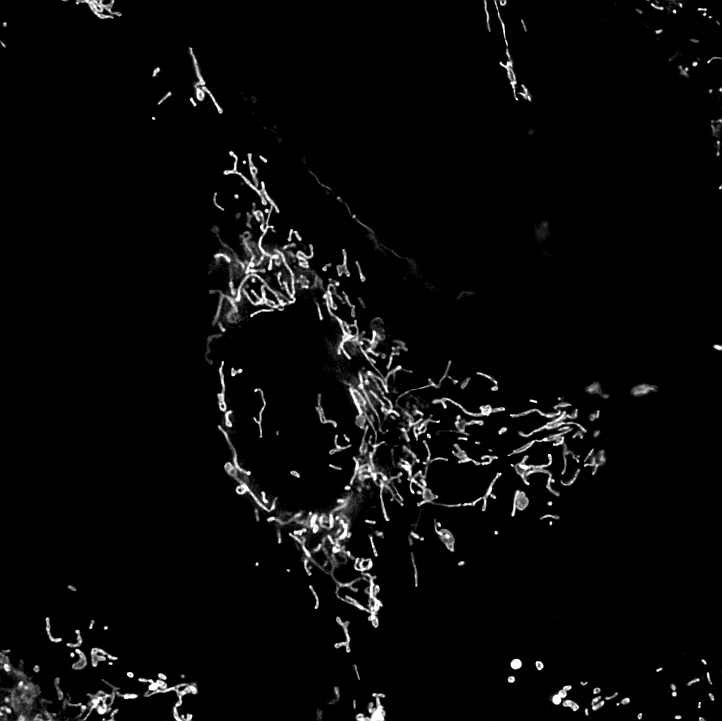

Supplement: Supplementary file 9 — Source data Fig. 7 [file 44319_2024_232_MOESM9_ESM.zip › Figure 7/7C/7C_skov3_siDrp1(-17)_mitotrackeronly.tif]

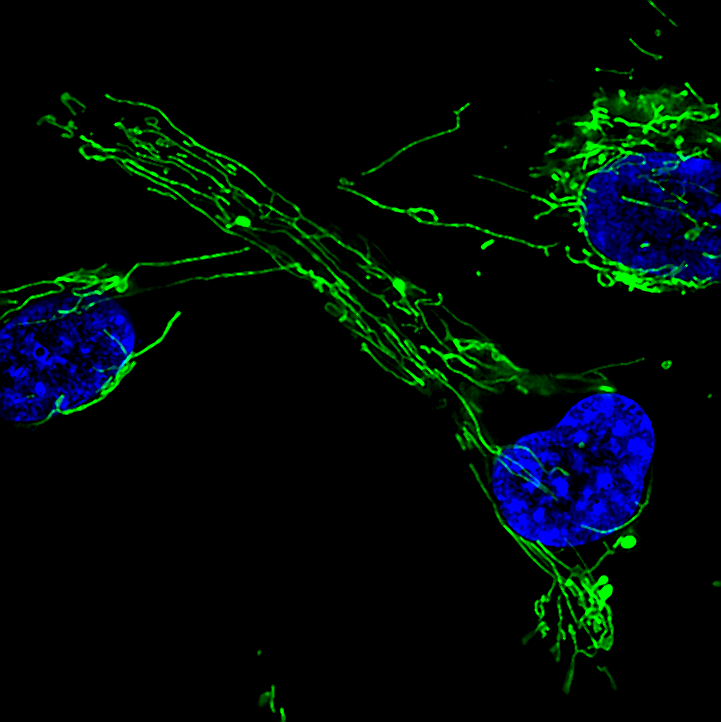

Supplement: Supplementary file 9 — Source data Fig. 7 [file 44319_2024_232_MOESM9_ESM.zip › Figure 7/7C/7C_skov3_siDrp1(Total)_SelectedArea.tif]

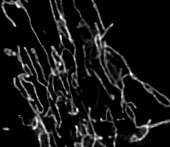

Supplement: Supplementary file 9 — Source data Fig. 7 [file 44319_2024_232_MOESM9_ESM.zip › Figure 7/7C/7C_skov3_siDrp1(1617)_zoomed.tif]

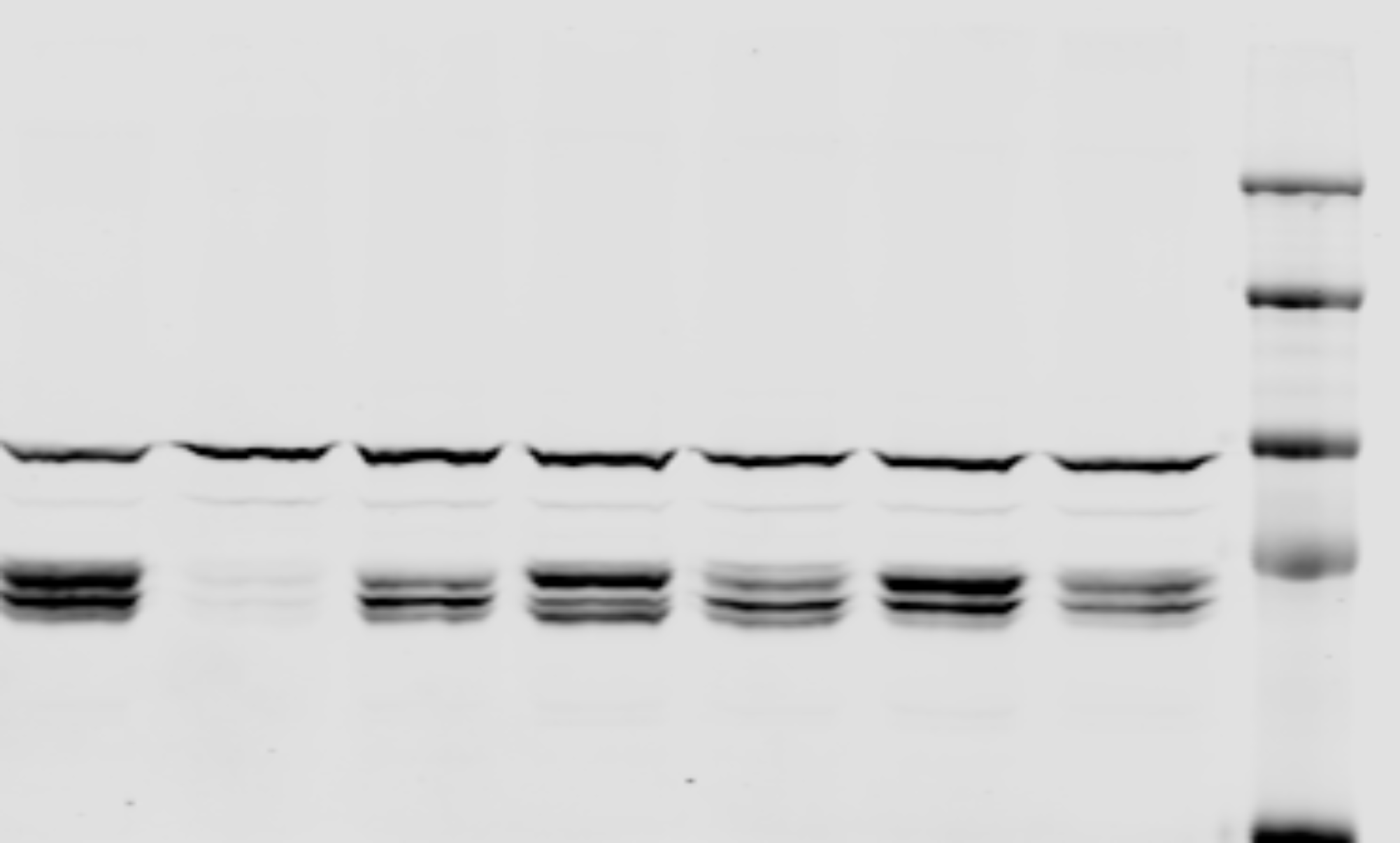

Supplement: Supplementary file 9 — Source data Fig. 7 [file 44319_2024_232_MOESM9_ESM.zip › Figure 7/7B/7B_Drp1_siDrp1.tif]

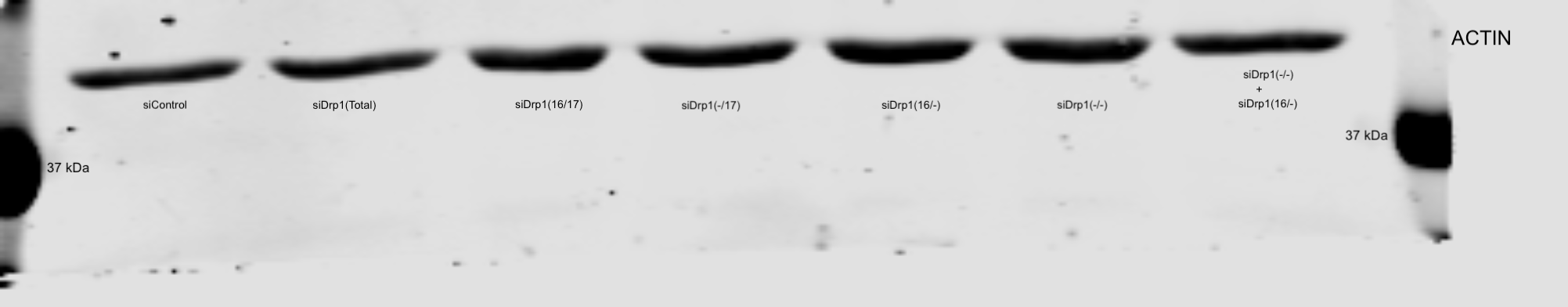

Supplement: Supplementary file 9 — Source data Fig. 7 [file 44319_2024_232_MOESM9_ESM.zip › Figure 7/7B/7B_Actin_siDrp1.tif]

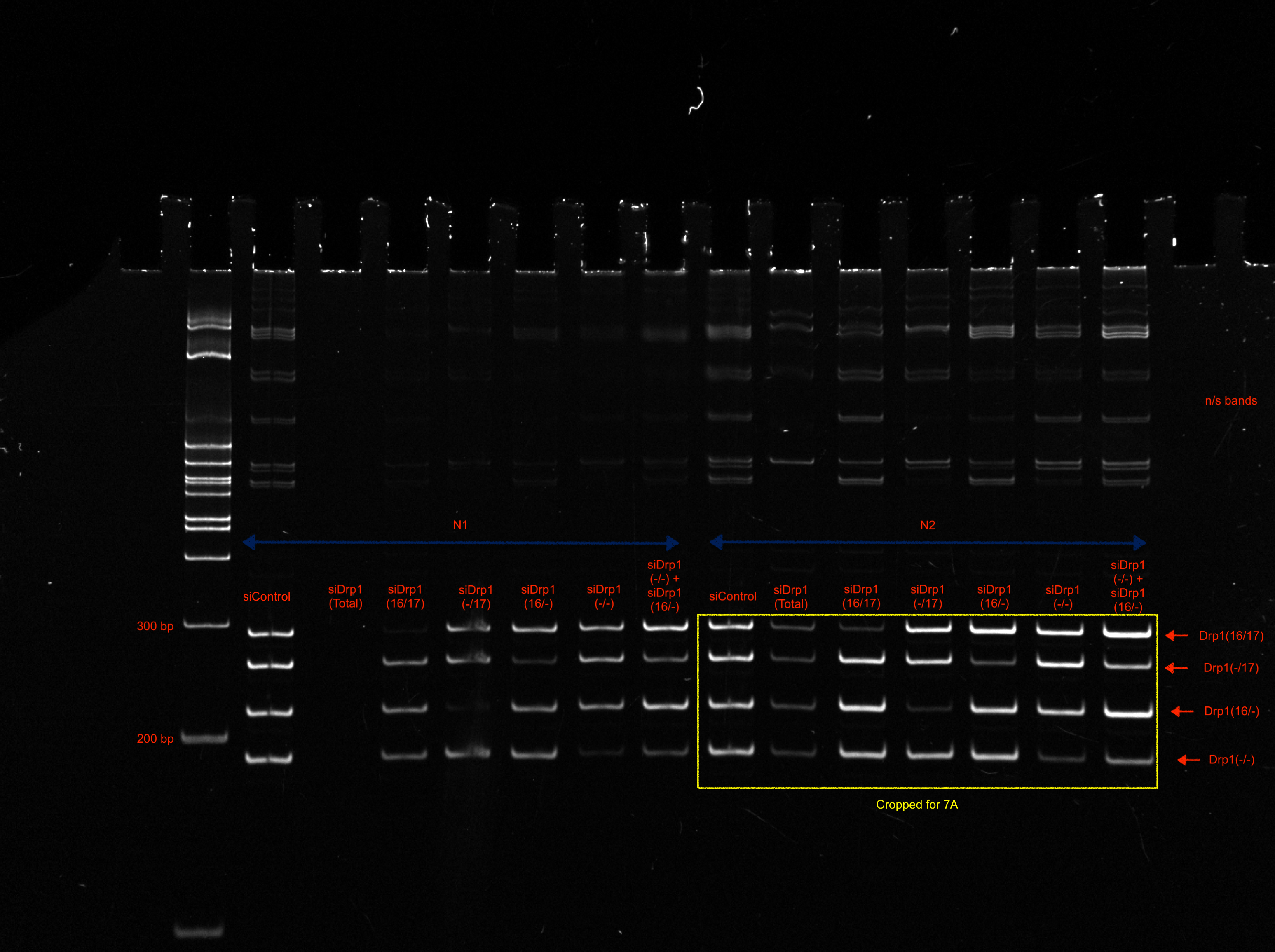

Supplement: Supplementary file 9 — Source data Fig. 7 [file 44319_2024_232_MOESM9_ESM.zip › Figure 7/7A/7A_siDNM1L_PCR.tif]

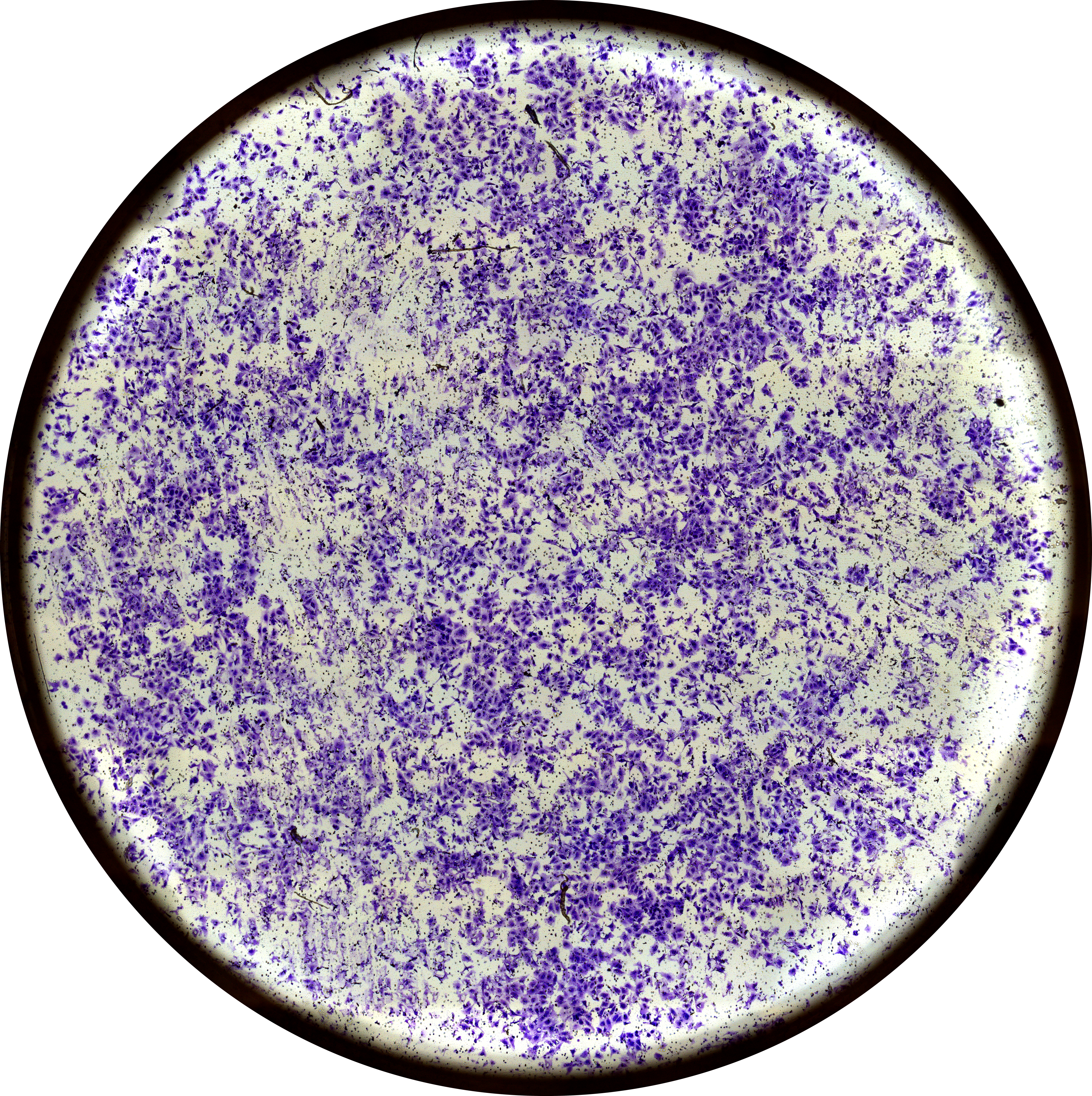

Supplement: Supplementary file 10 — Source data Fig. 8 [file 44319_2024_232_MOESM10_ESM.zip › Figure 8/8G/8G_siControl_Migration.tif]

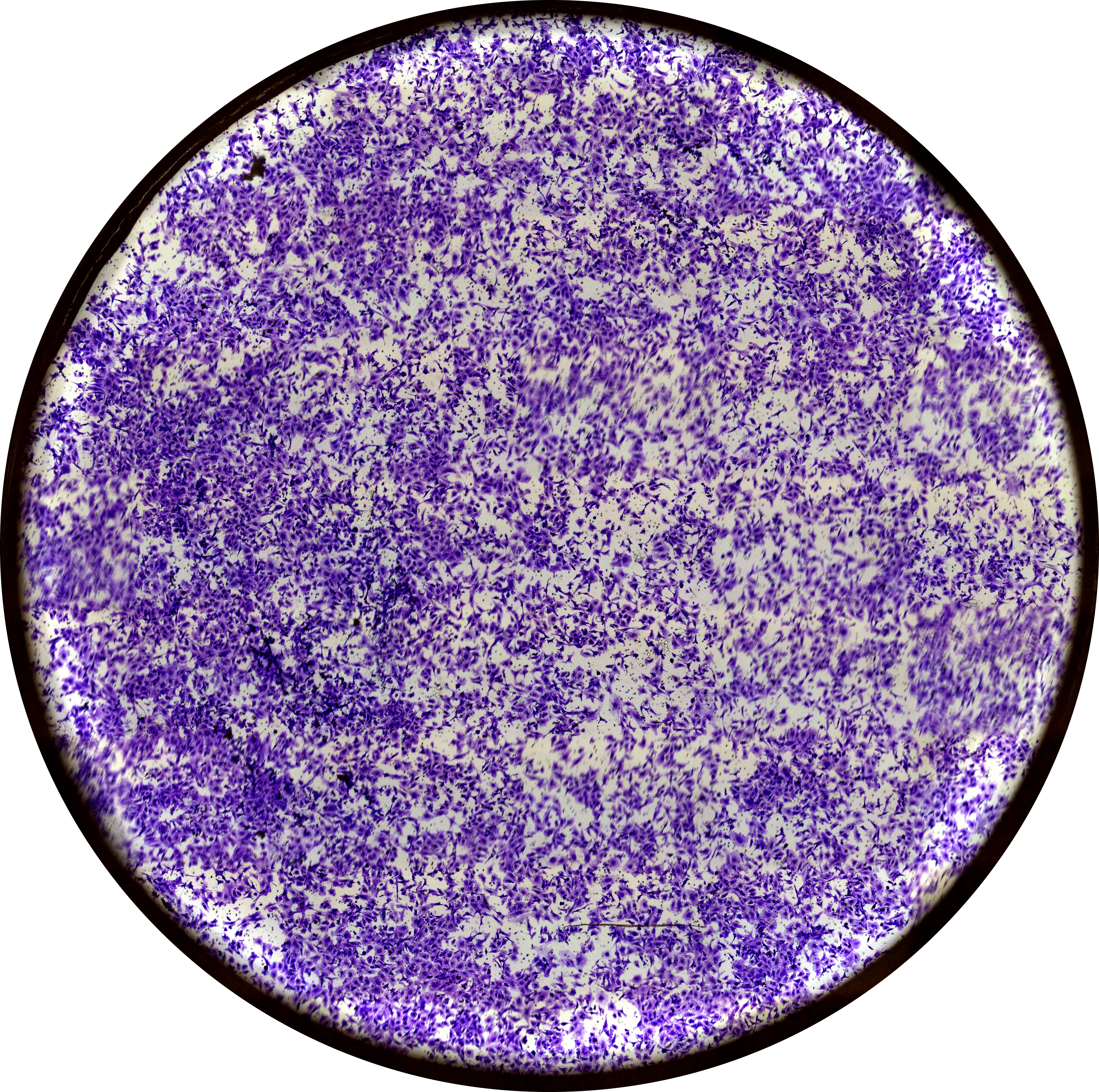

Supplement: Supplementary file 10 — Source data Fig. 8 [file 44319_2024_232_MOESM10_ESM.zip › Figure 8/8G/8G_siDrp1(1617)_Migration.tif]

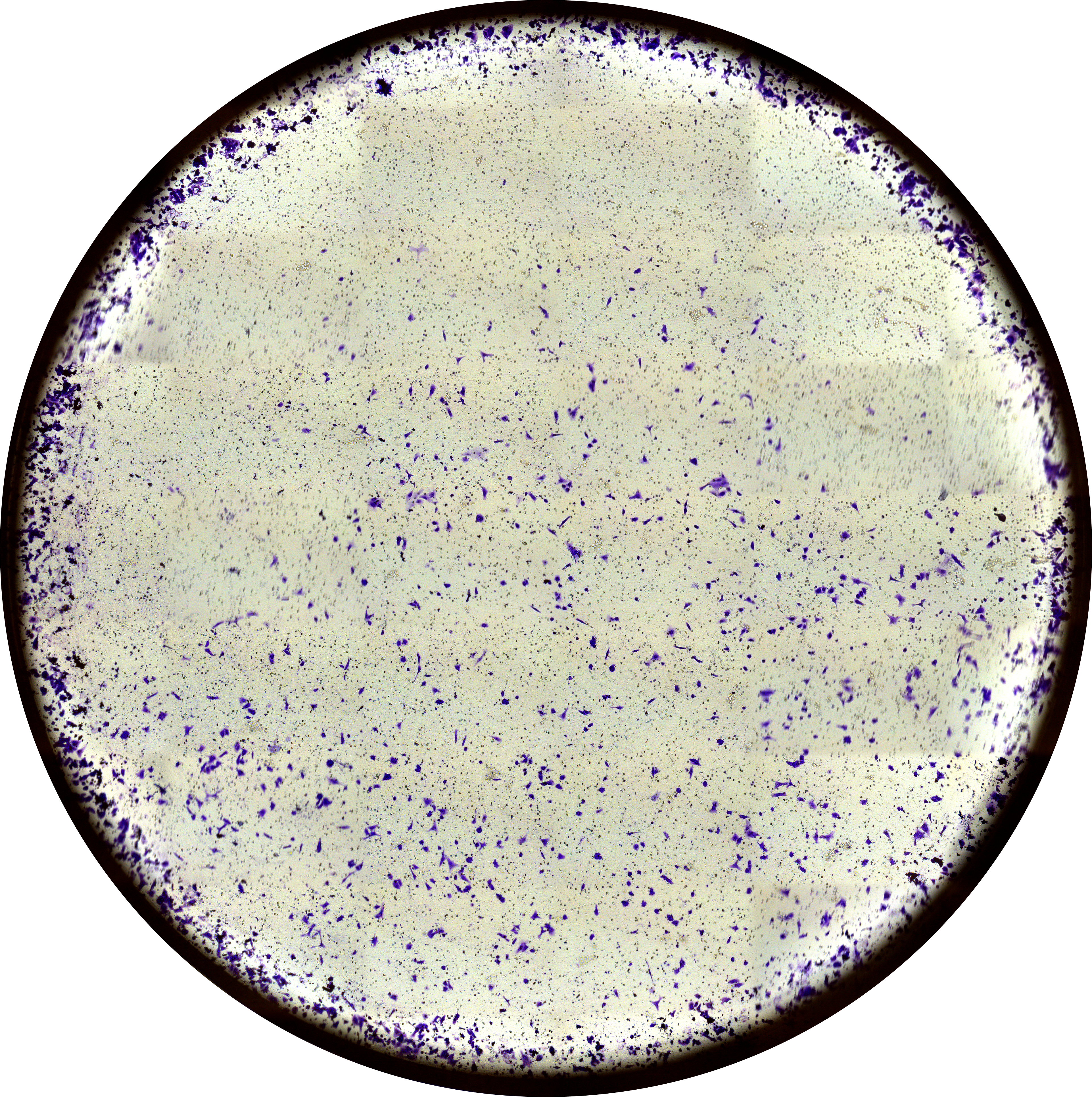

Supplement: Supplementary file 10 — Source data Fig. 8 [file 44319_2024_232_MOESM10_ESM.zip › Figure 8/8G/8G_siDrp1(total)_Migration.tif]

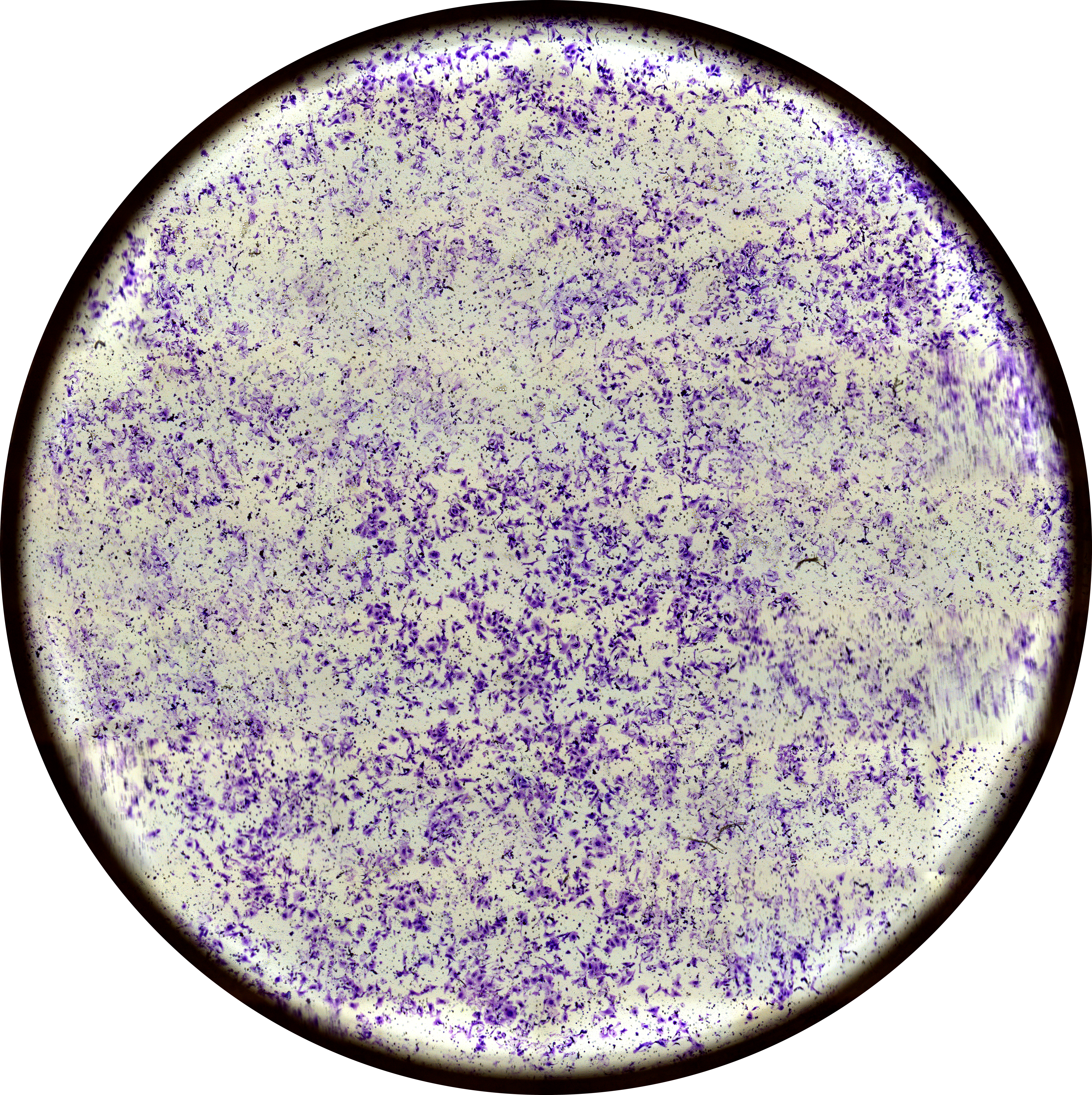

Supplement: Supplementary file 10 — Source data Fig. 8 [file 44319_2024_232_MOESM10_ESM.zip › Figure 8/8G/8G_siDrp1(-17)_MIgration.tif]

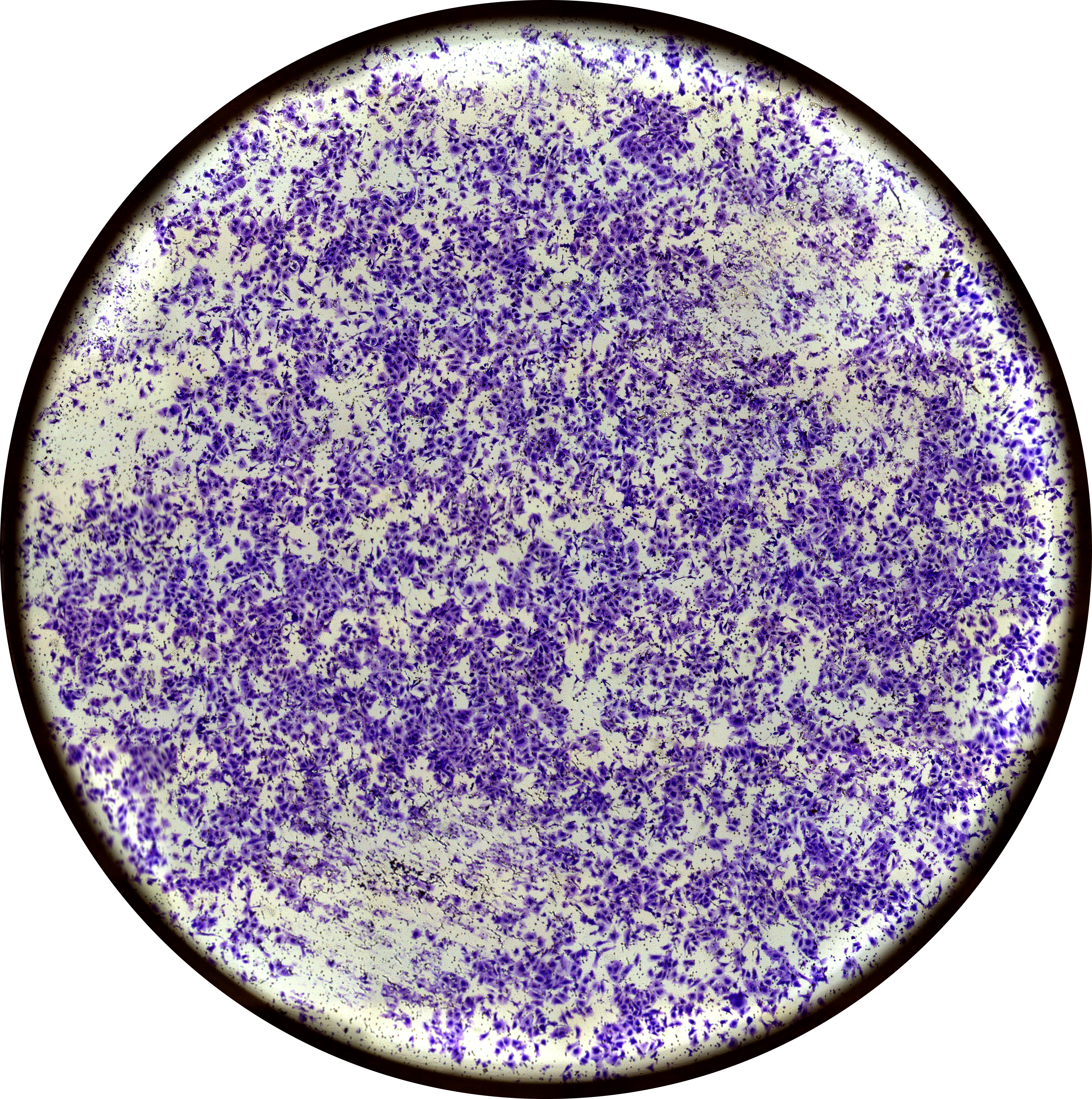

Supplement: Supplementary file 10 — Source data Fig. 8 [file 44319_2024_232_MOESM10_ESM.zip › Figure 8/8G/8G_siDrp1(--)andsiDrp1(16-)_Migration.tif]

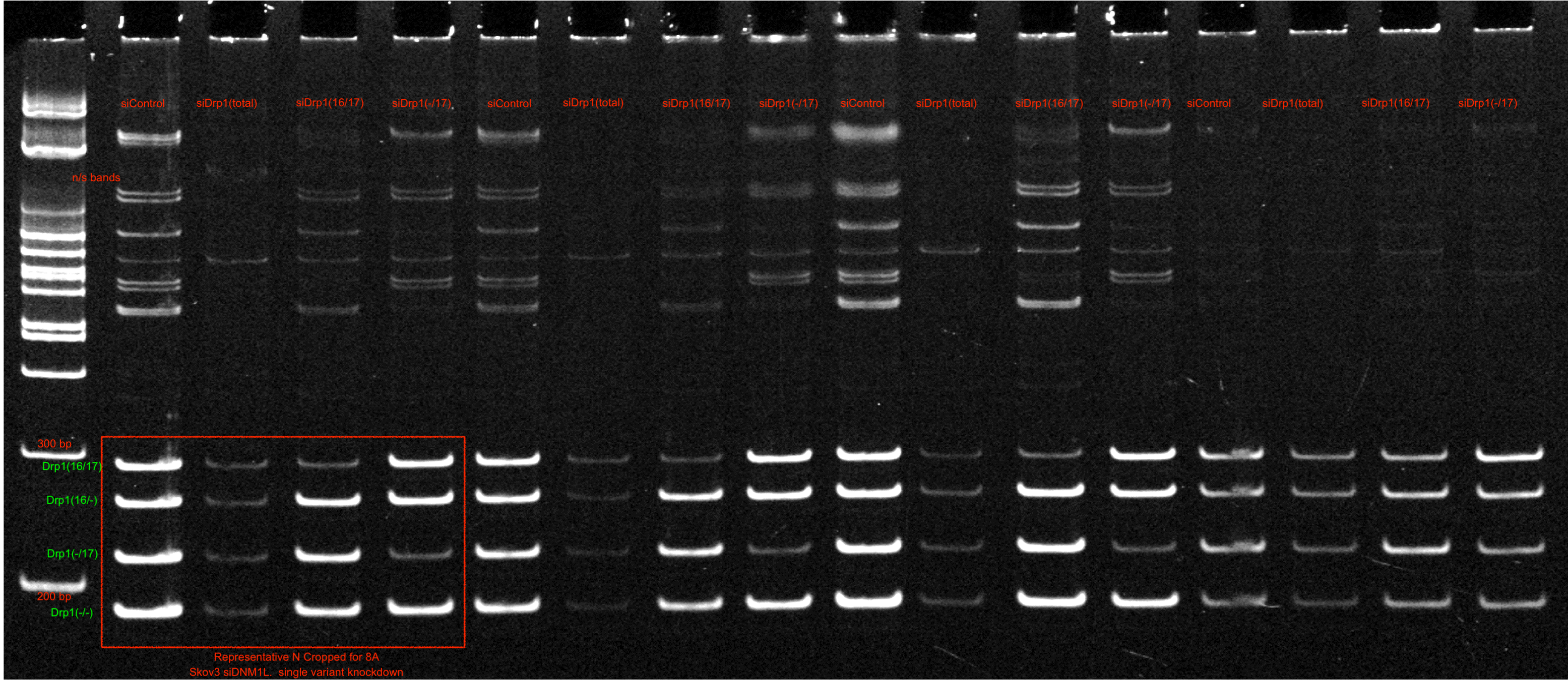

Supplement: Supplementary file 10 — Source data Fig. 8 [file 44319_2024_232_MOESM10_ESM.zip › Figure 8/8A/8A_siSingleDrp1 Variant_RTPCR.png]

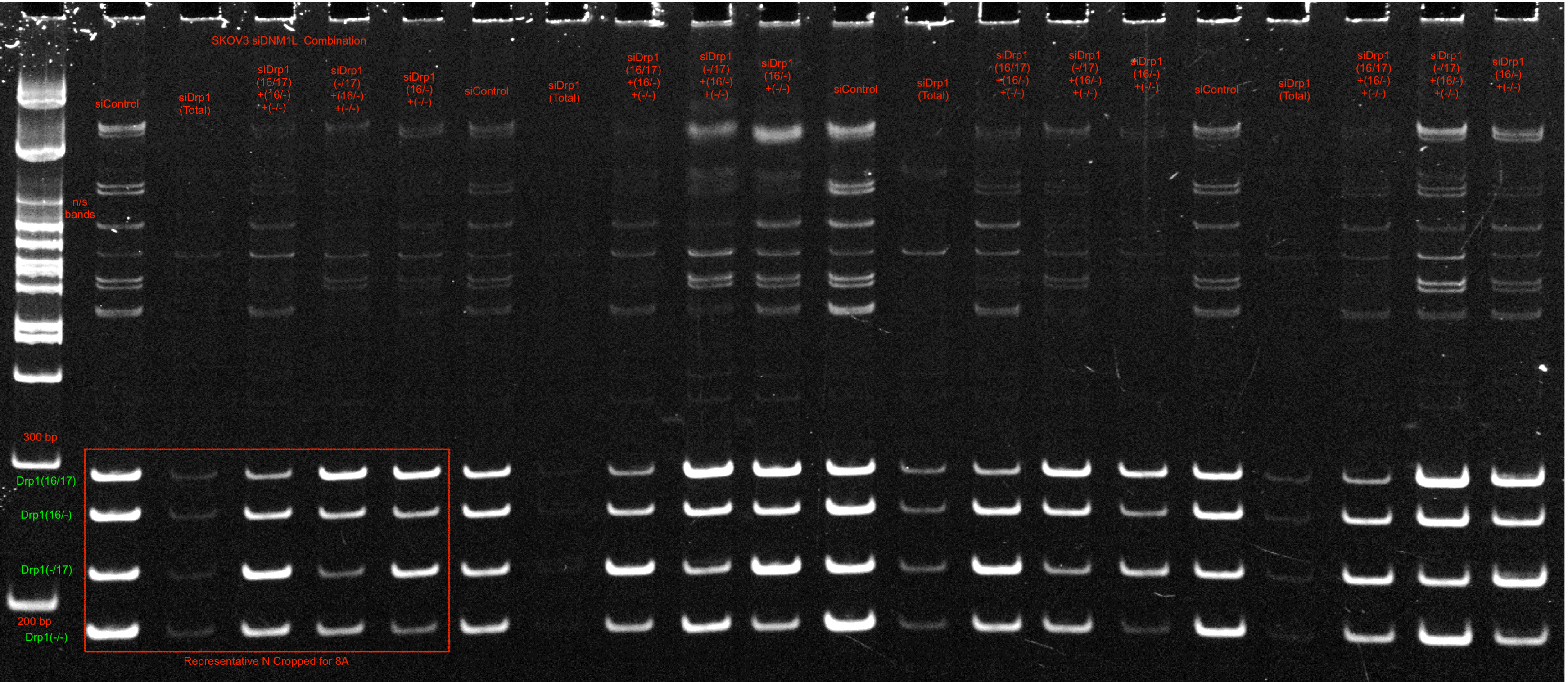

Supplement: Supplementary file 10 — Source data Fig. 8 [file 44319_2024_232_MOESM10_ESM.zip › Figure 8/8A/8A_siComboDrp1 Variants_RTPCR.png]
