# Supplementary material for: Drp1 splice variants regulate ovarian cancer mitochondrial dynamics and tumor progression
Source: EMBO Rep. 2024 Aug 27;25(10):16. doi: 10.1038/s44319-024-00232-4 (PMC11467262; doi:10.1038/s44319-024-00232-4)
Supplement: Supplementary file 7 — Source data Fig. 5 [file 44319_2024_232_MOESM7_ESM.zip › Figure 5/5D/5D_MouseLuciferase.pptx]

## Slide 1
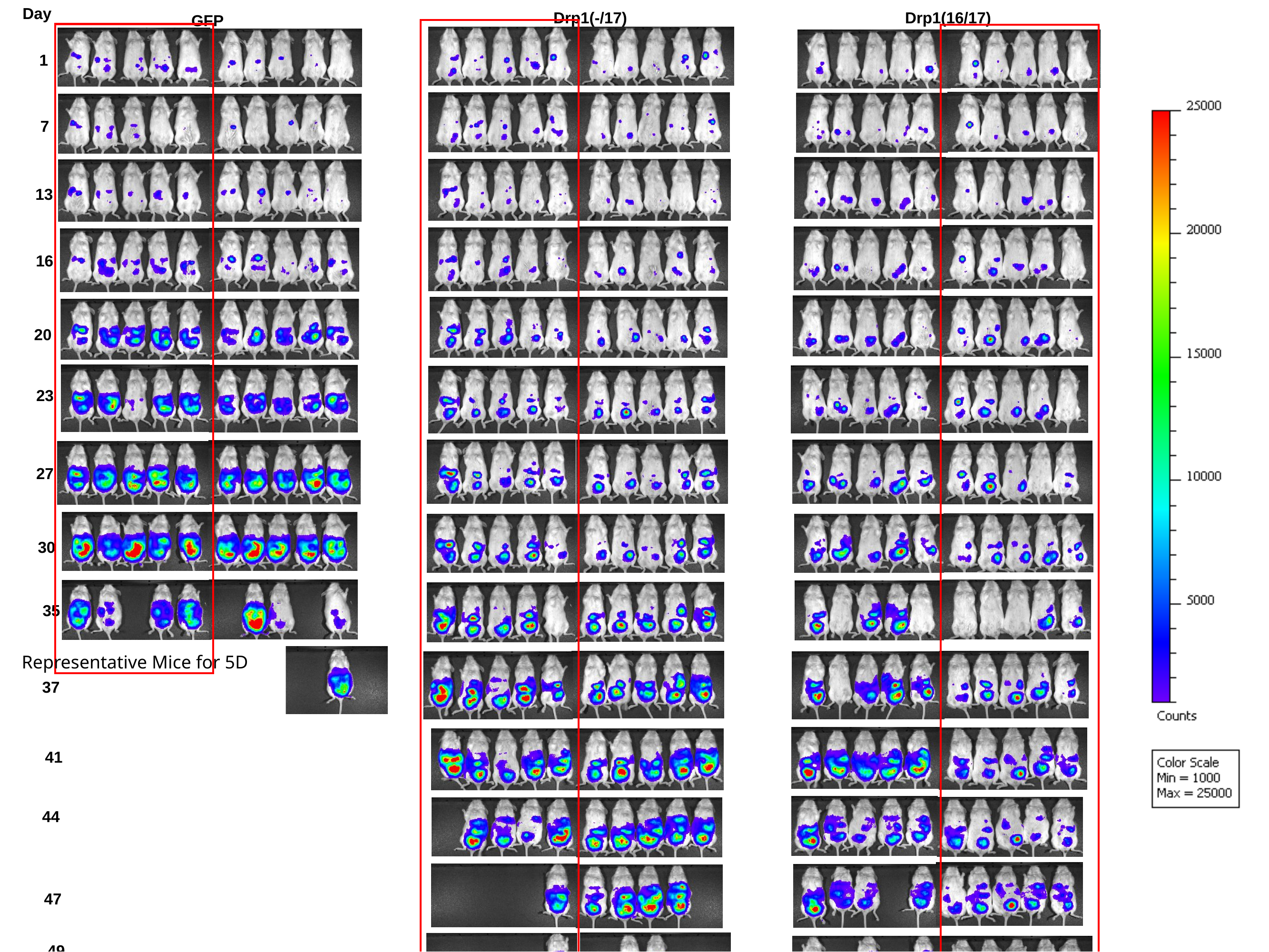

Day
Drp1(-/17)
Drp1(16/17)
GFP
1
7
13
16
20
23
27
30
35
Representative Mice for 5D
37
41
44
47
49
Representative Mice for 5D
Representative Mice for 5D
