# Supplementary material for: Drp1 splice variants regulate ovarian cancer mitochondrial dynamics and tumor progression
Source: EMBO Rep. 2024 Aug 27;25(10):16. doi: 10.1038/s44319-024-00232-4 (PMC11467262; doi:10.1038/s44319-024-00232-4)
Supplement: Supplementary file 7 — Source data Fig. 5 [file 44319_2024_232_MOESM7_ESM.zip › Figure 5/5C/5C migrations replicates/5C_OVCA433_SKOV3_Drp1 Migration Assay Replicates.pptx]

## Slide 1
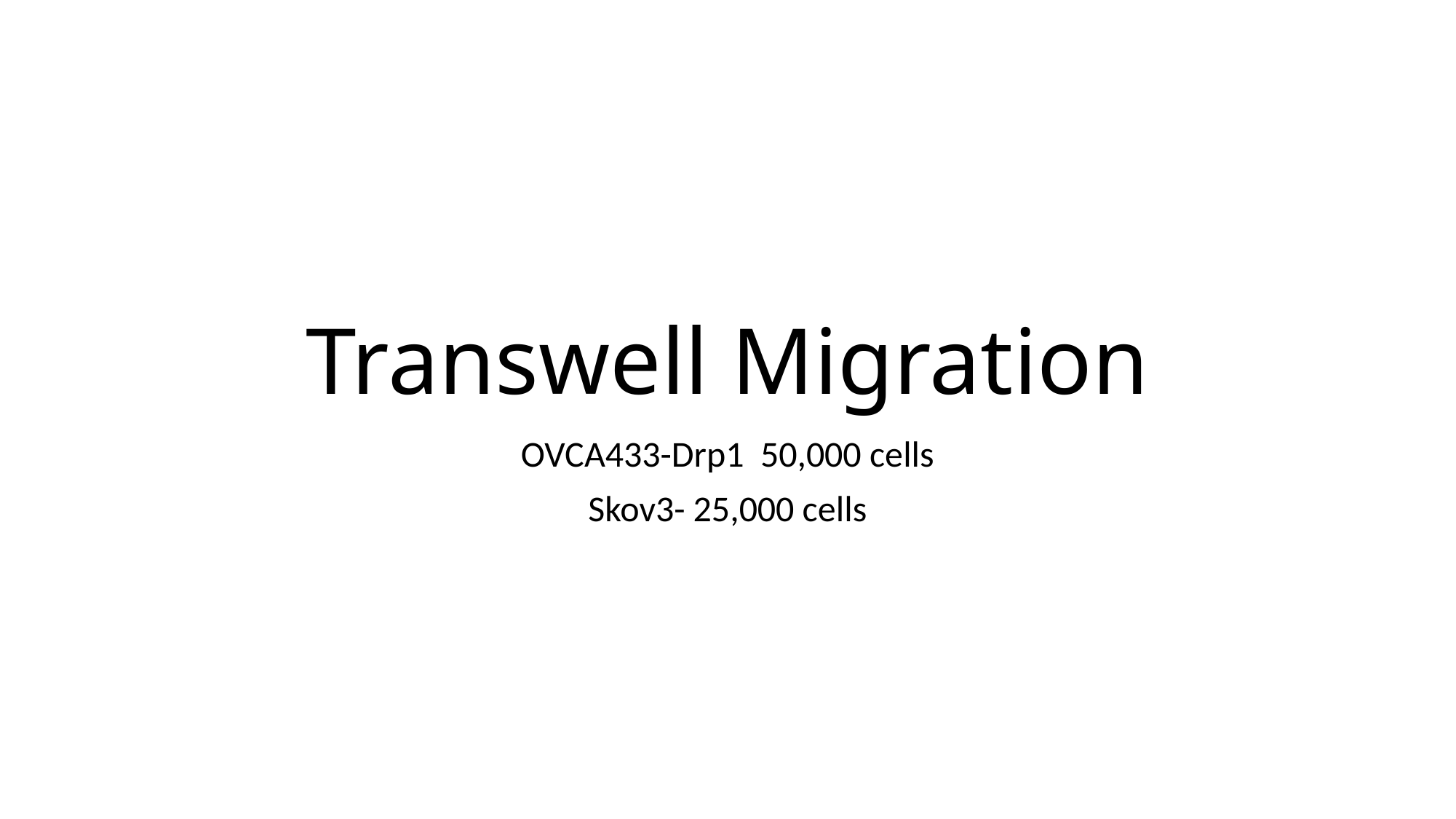

# Transwell Migration
OVCA433-Drp1 50,000 cells
Skov3- 25,000 cells

## Slide 2
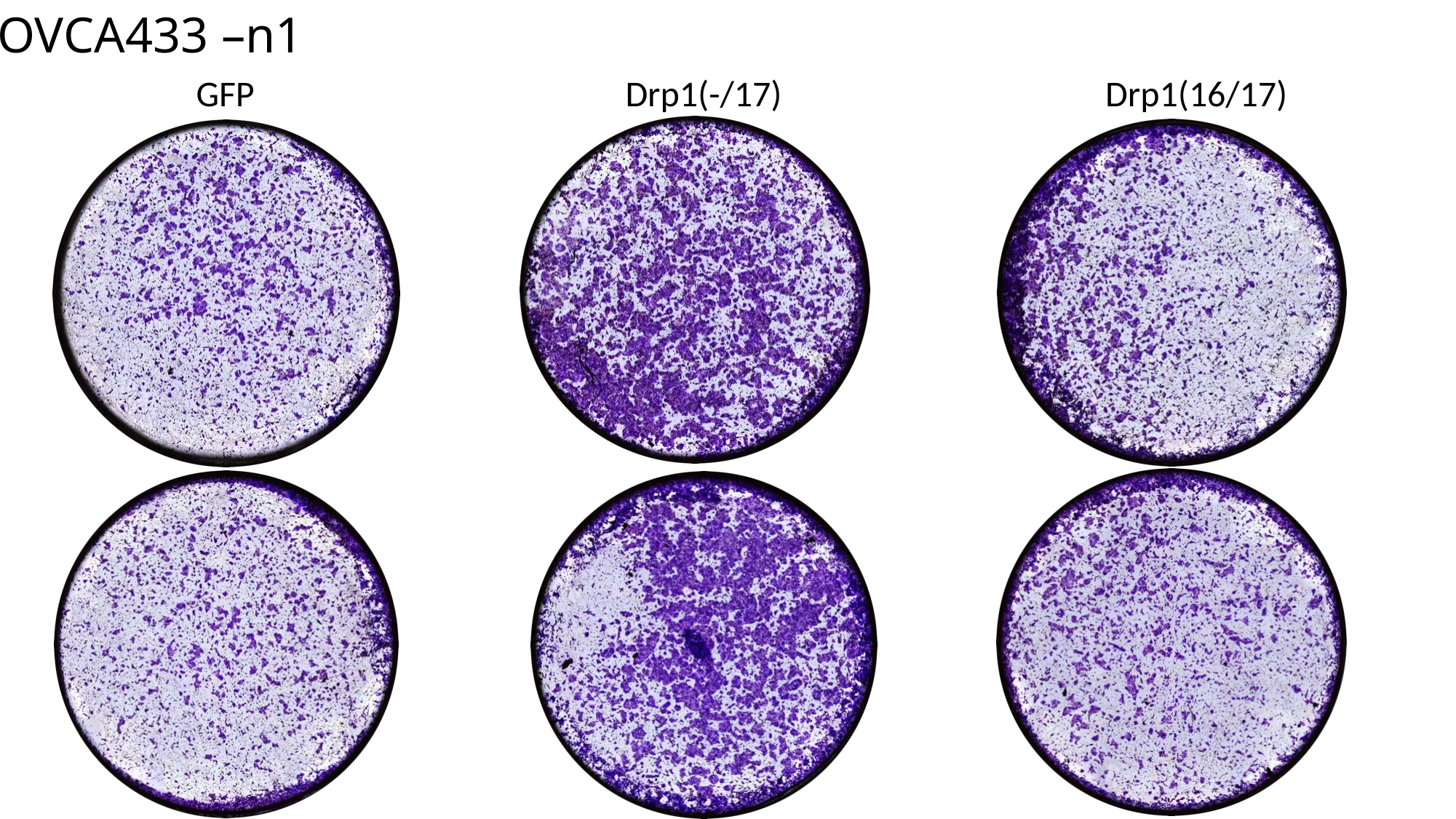

# OVCA433 –n1
Drp1(16/17)
GFP
Drp1(-/17)

## Slide 3
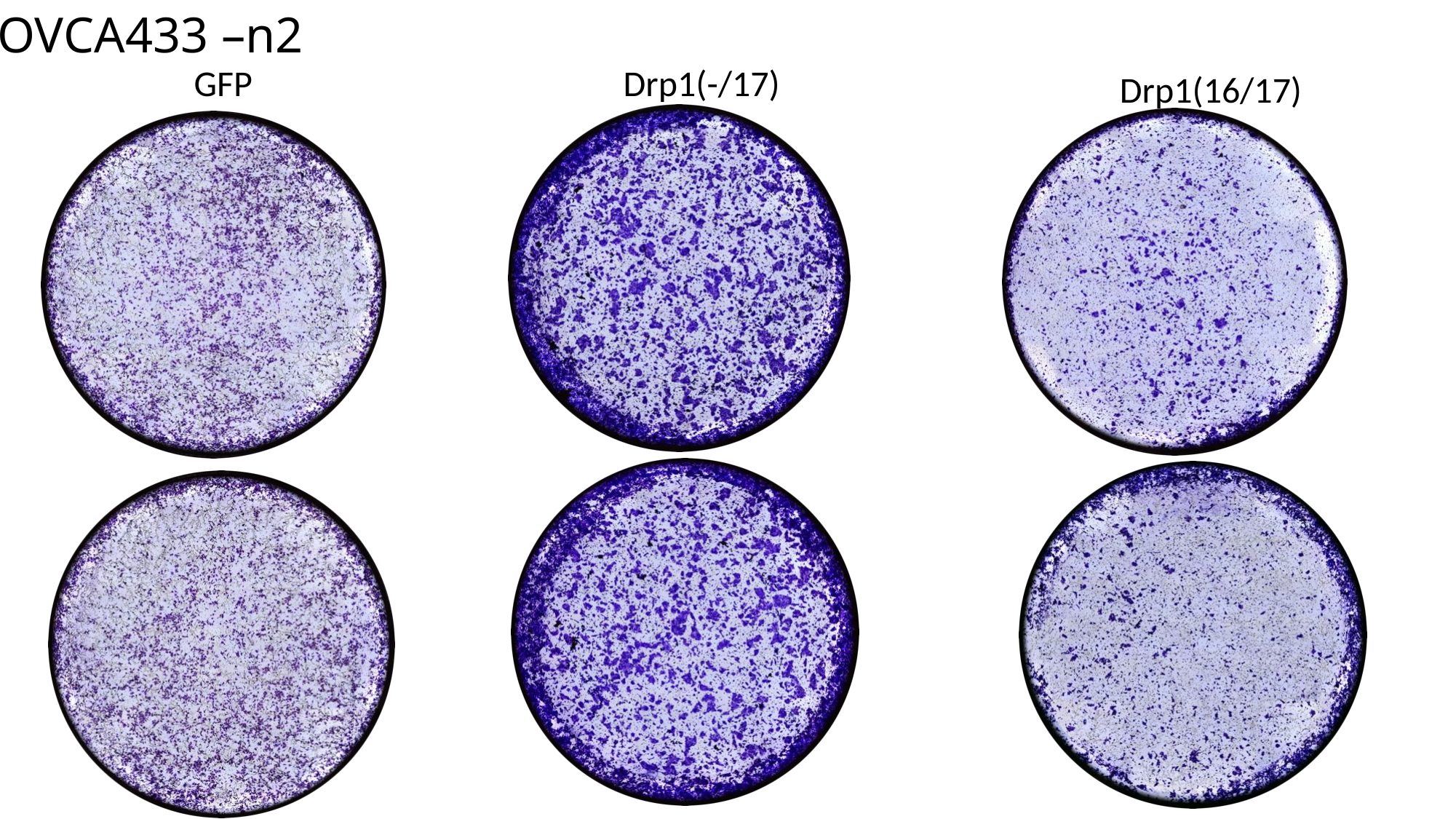

# OVCA433 –n2
GFP
Drp1(-/17)
Drp1(16/17)

## Slide 4
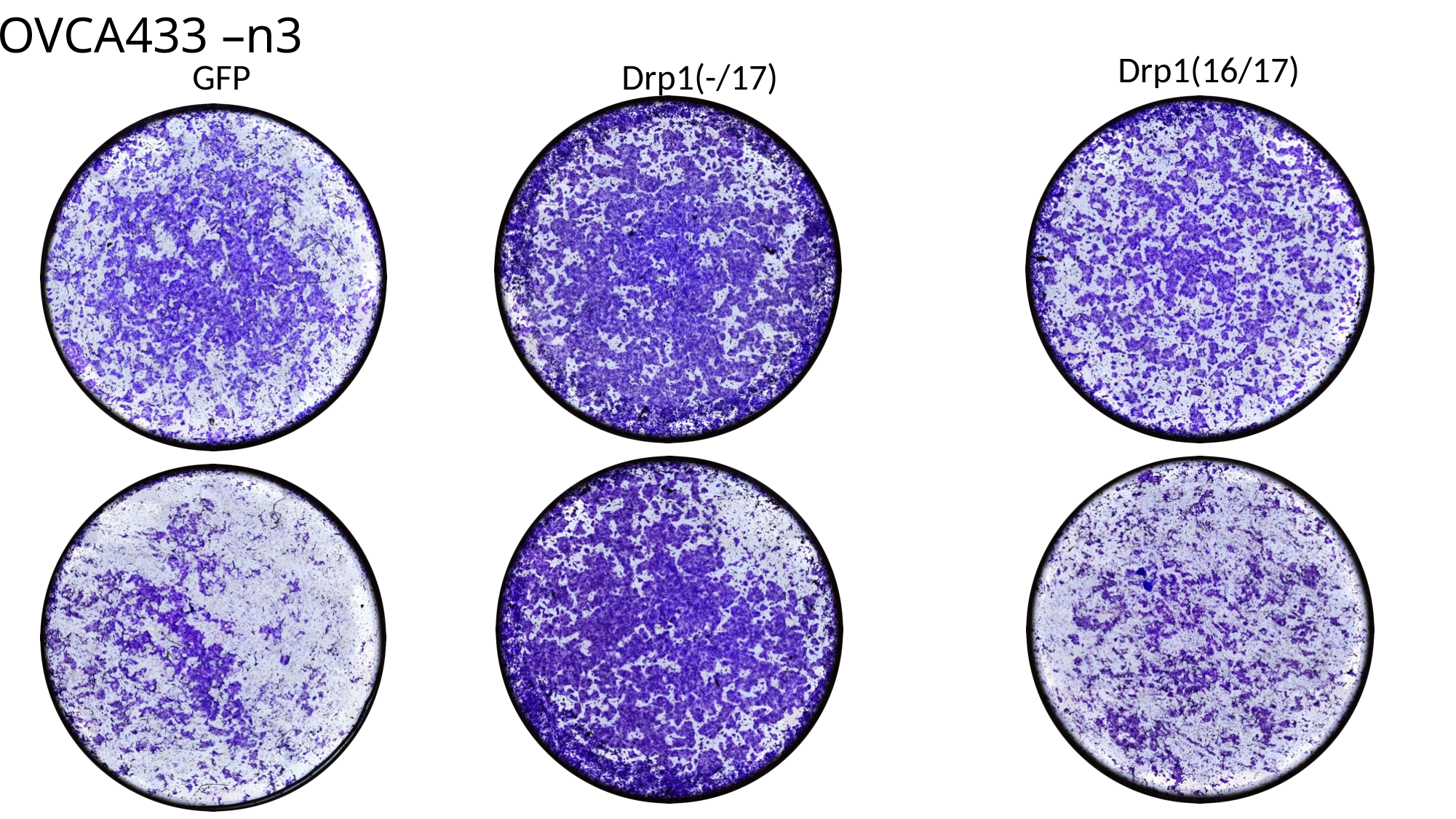

# OVCA433 –n3
Drp1(16/17)
GFP
Drp1(-/17)

## Slide 5
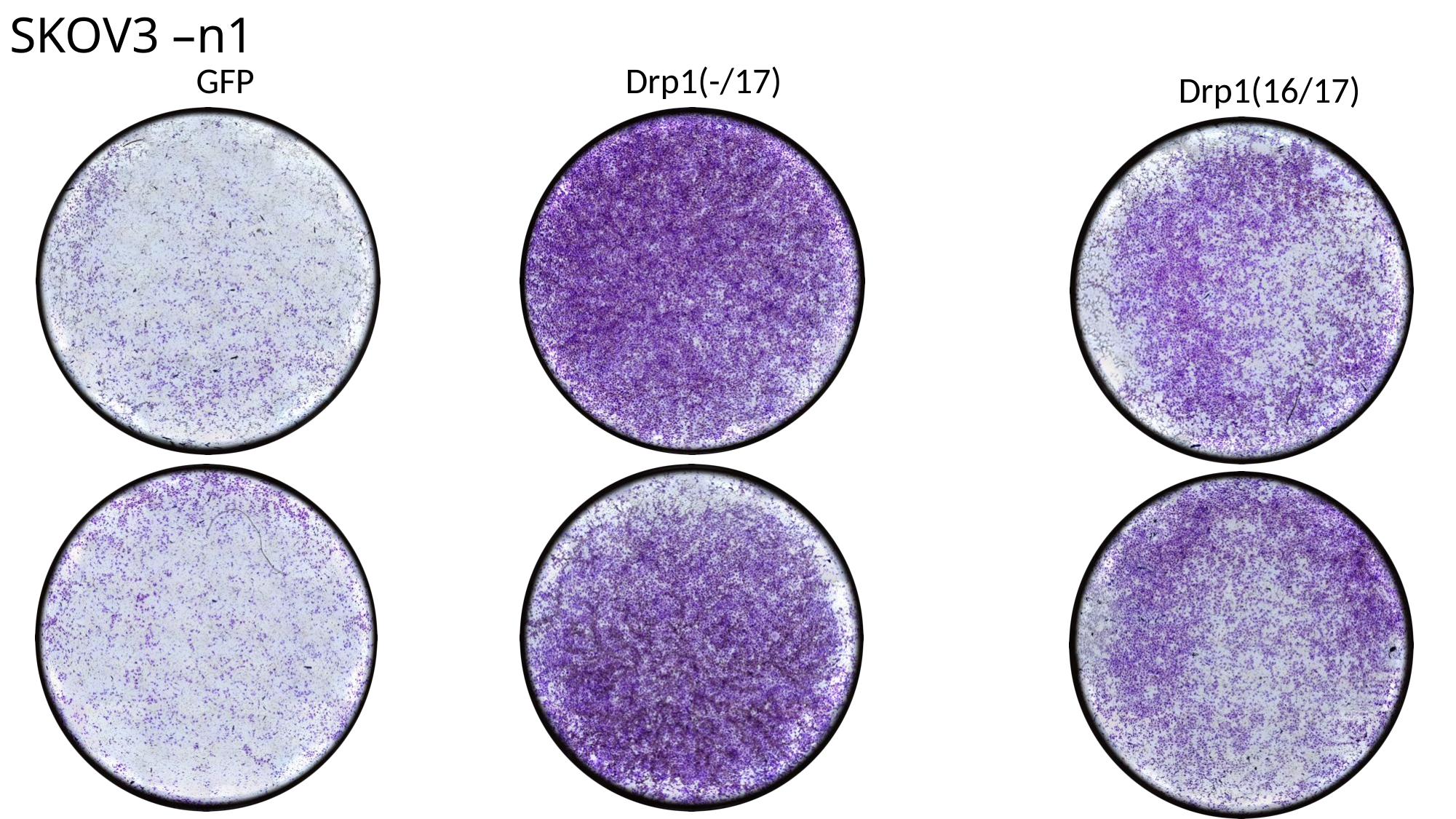

# SKOV3 –n1
GFP
Drp1(-/17)
Drp1(16/17)

## Slide 6
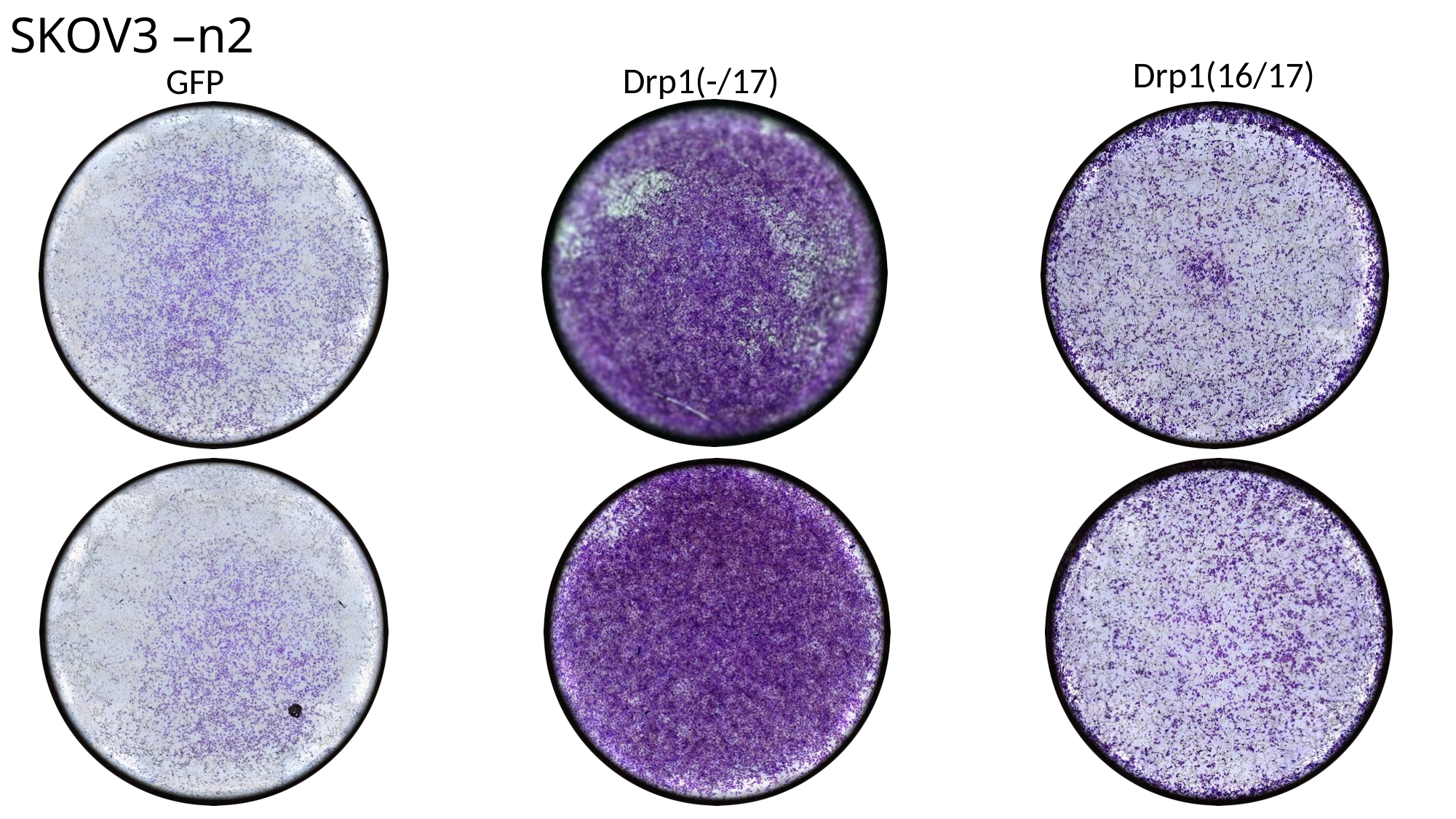

# SKOV3 –n2
Drp1(16/17)
Drp1(-/17)
GFP

## Slide 7
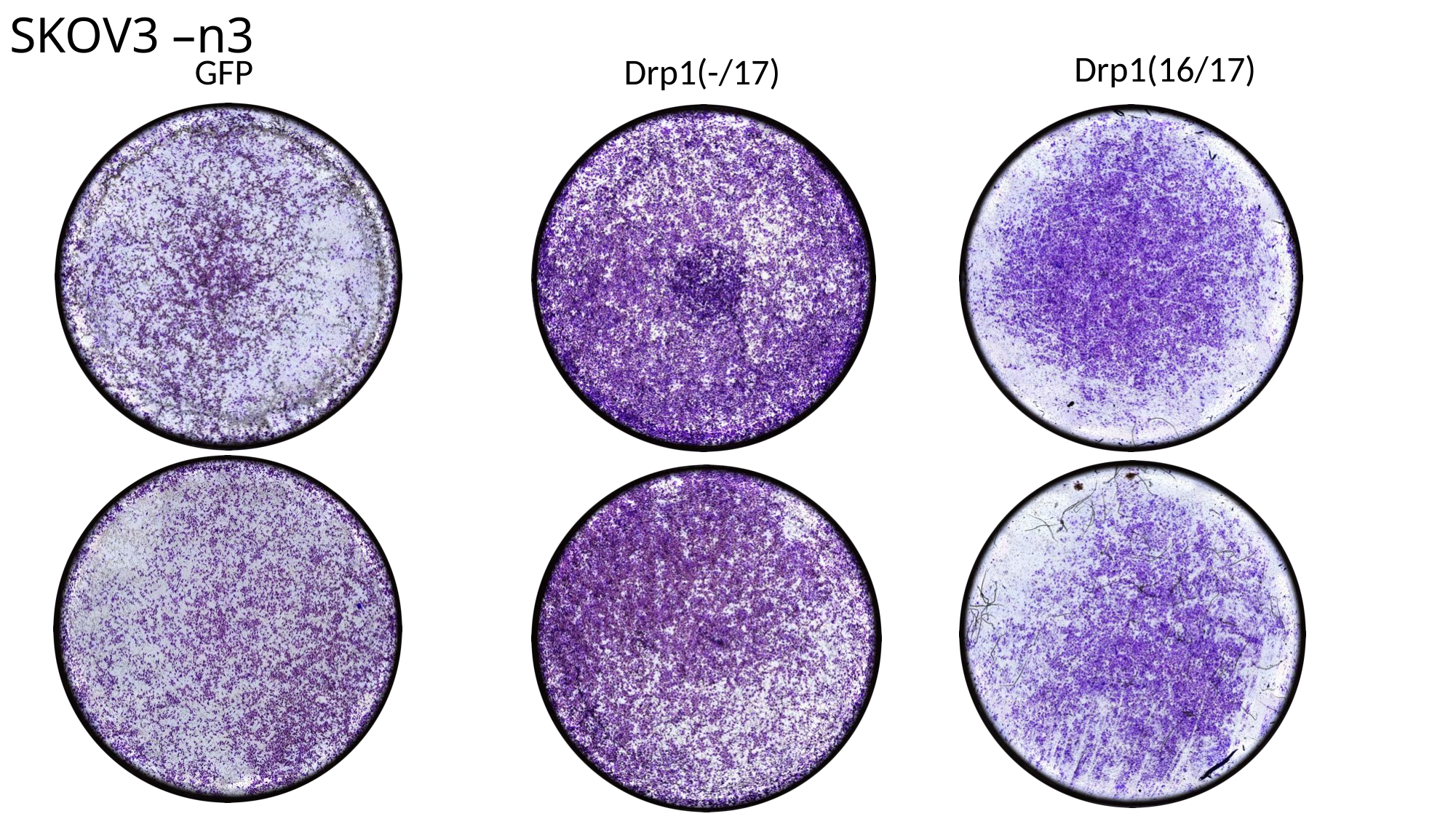

# SKOV3 –n3
Drp1(16/17)
GFP
Drp1(-/17)
