# Supplementary material for: Drp1 splice variants regulate ovarian cancer mitochondrial dynamics and tumor progression
Source: EMBO Rep. 2024 Aug 27;25(10):16. doi: 10.1038/s44319-024-00232-4 (PMC11467262; doi:10.1038/s44319-024-00232-4)
Supplement: Supplementary file 7 — Source data Fig. 5 [file 44319_2024_232_MOESM7_ESM.zip › Figure 5/5B/5B replicates/Skov3_Drp1O.E_Clonogencity Replicates .pptx]

## Slide 1
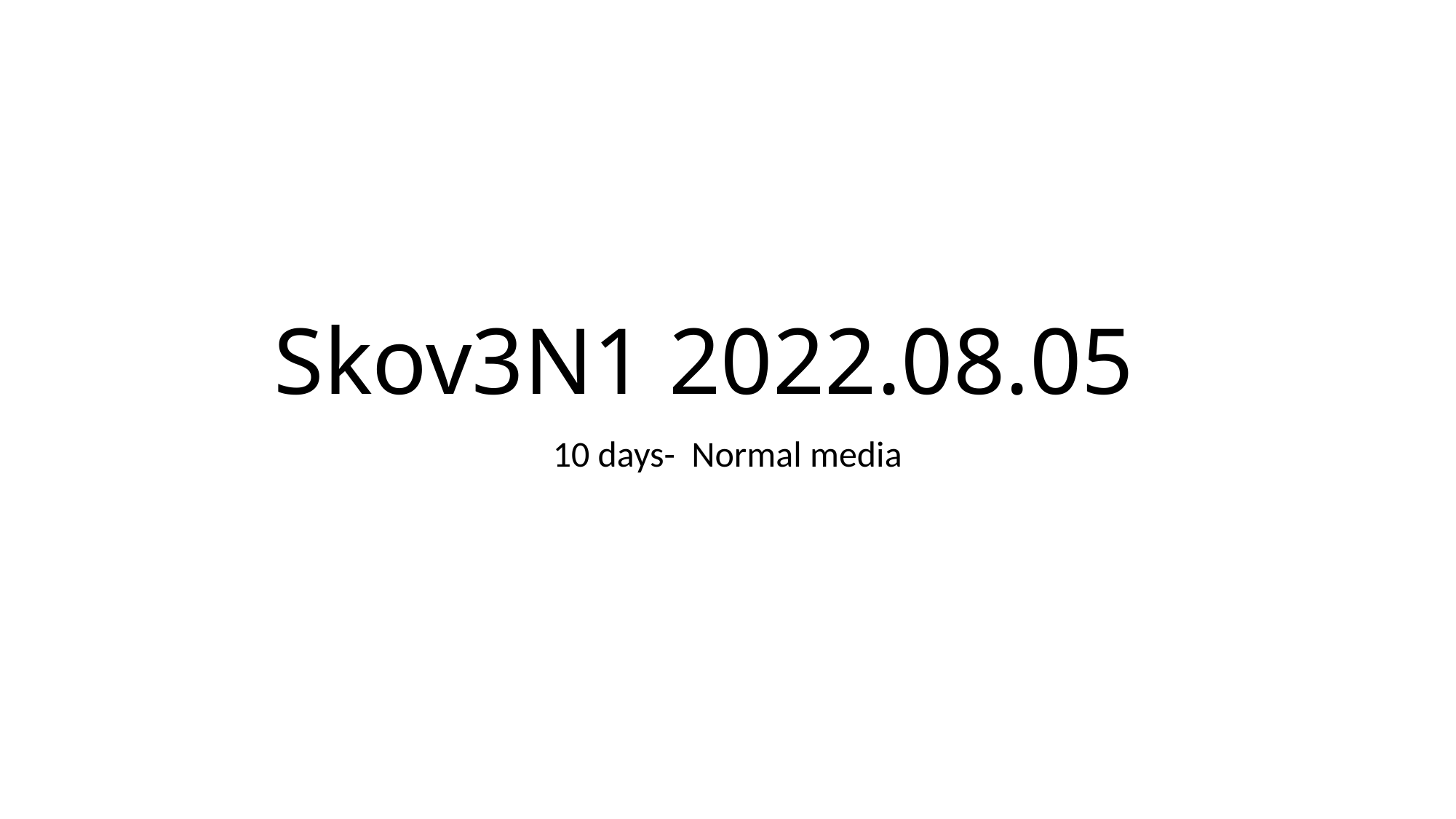

# Skov3N1 2022.08.05
10 days- Normal media

## Slide 2
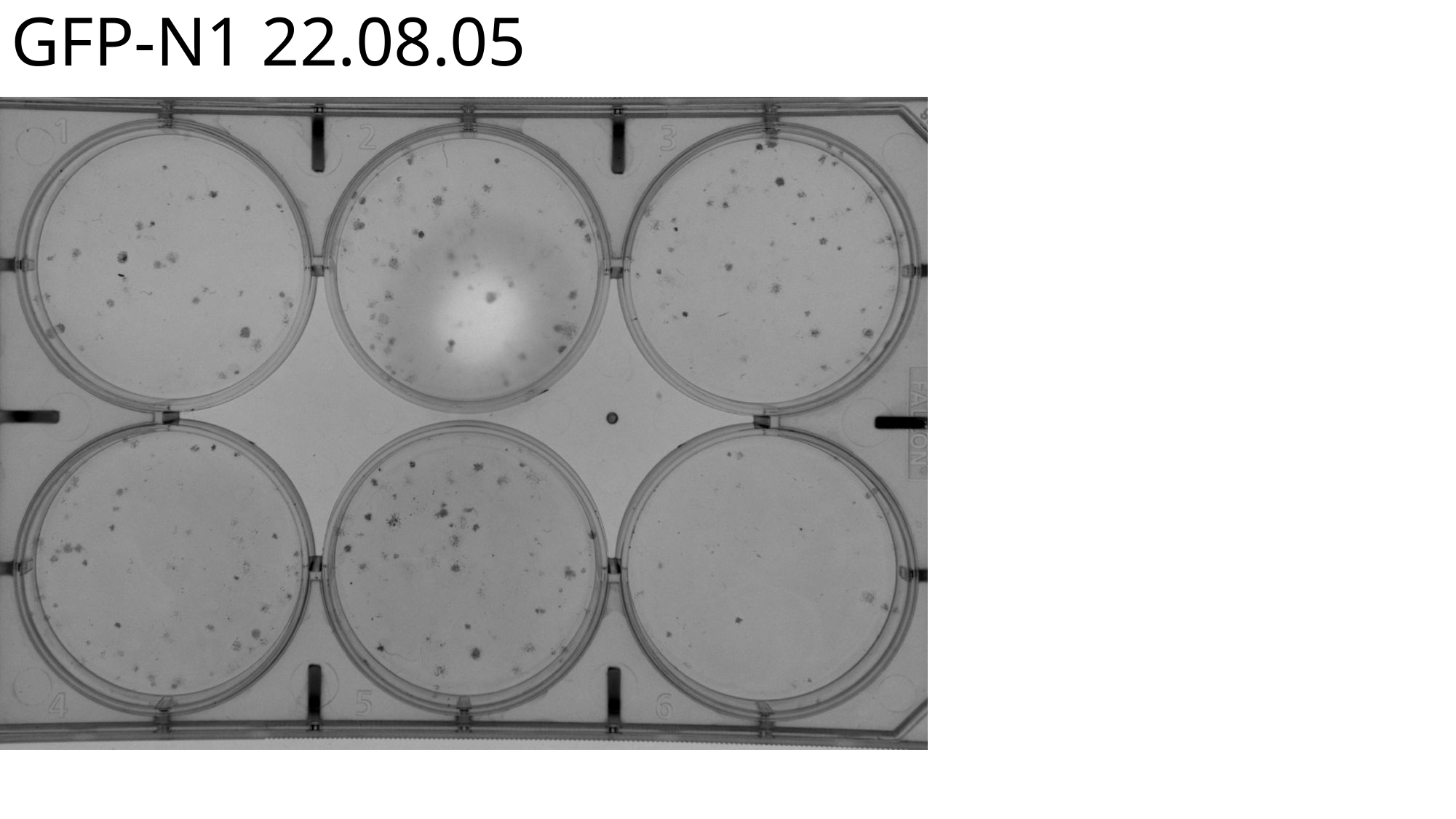

# GFP-N1 22.08.05

## Slide 3
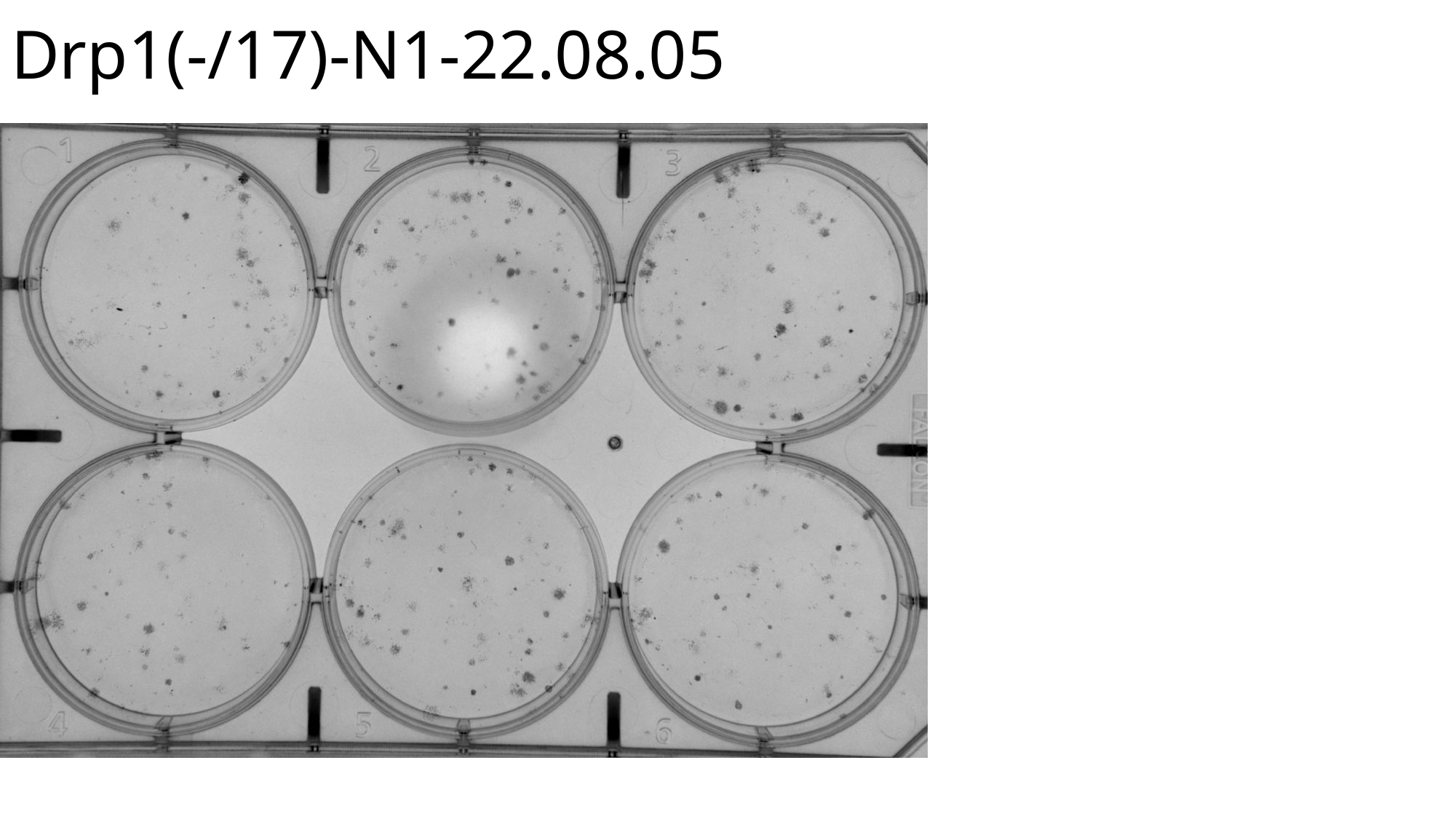

# Drp1(-/17)-N1-22.08.05

## Slide 4
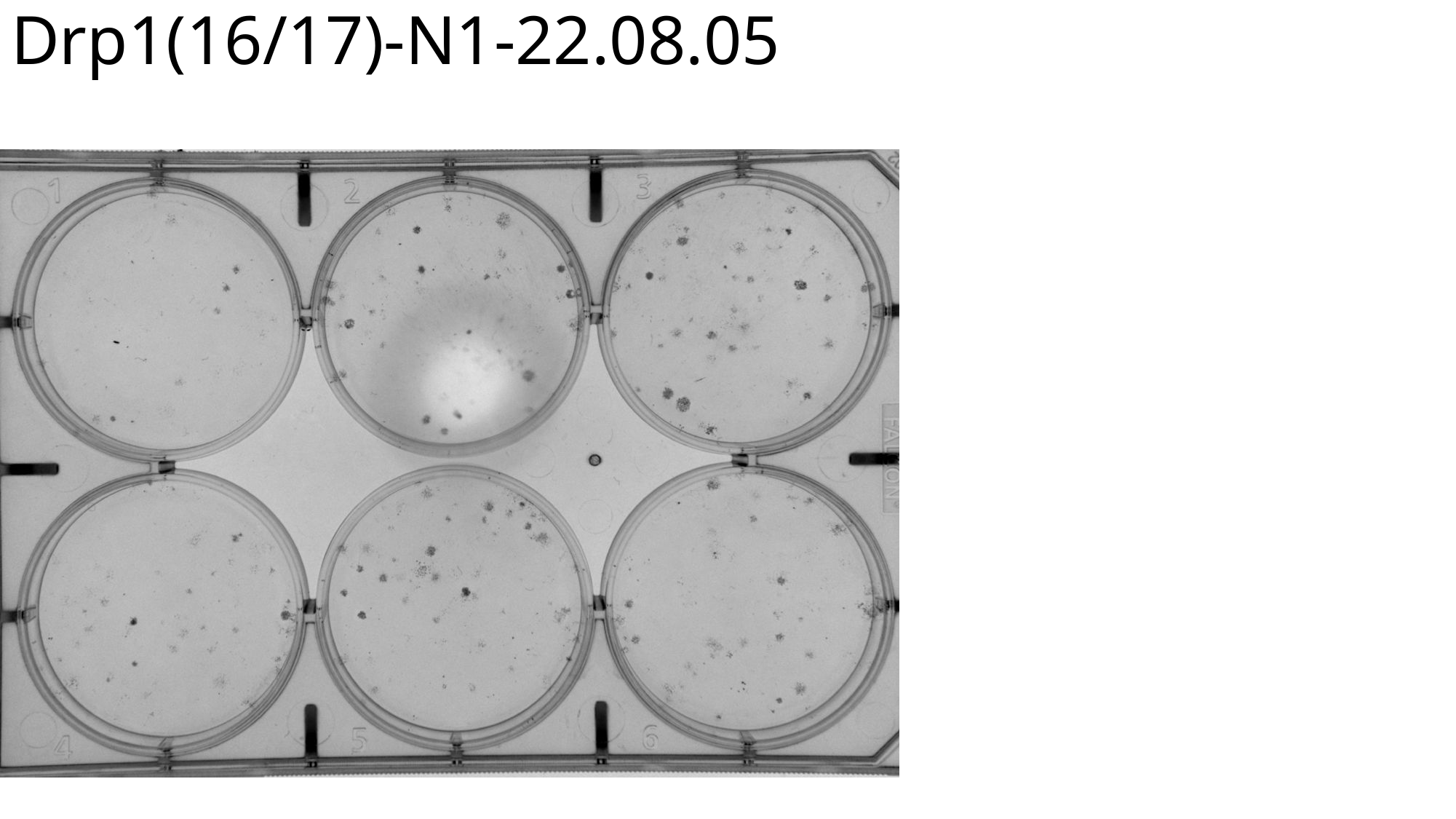

# Drp1(16/17)-N1-22.08.05

## Slide 5
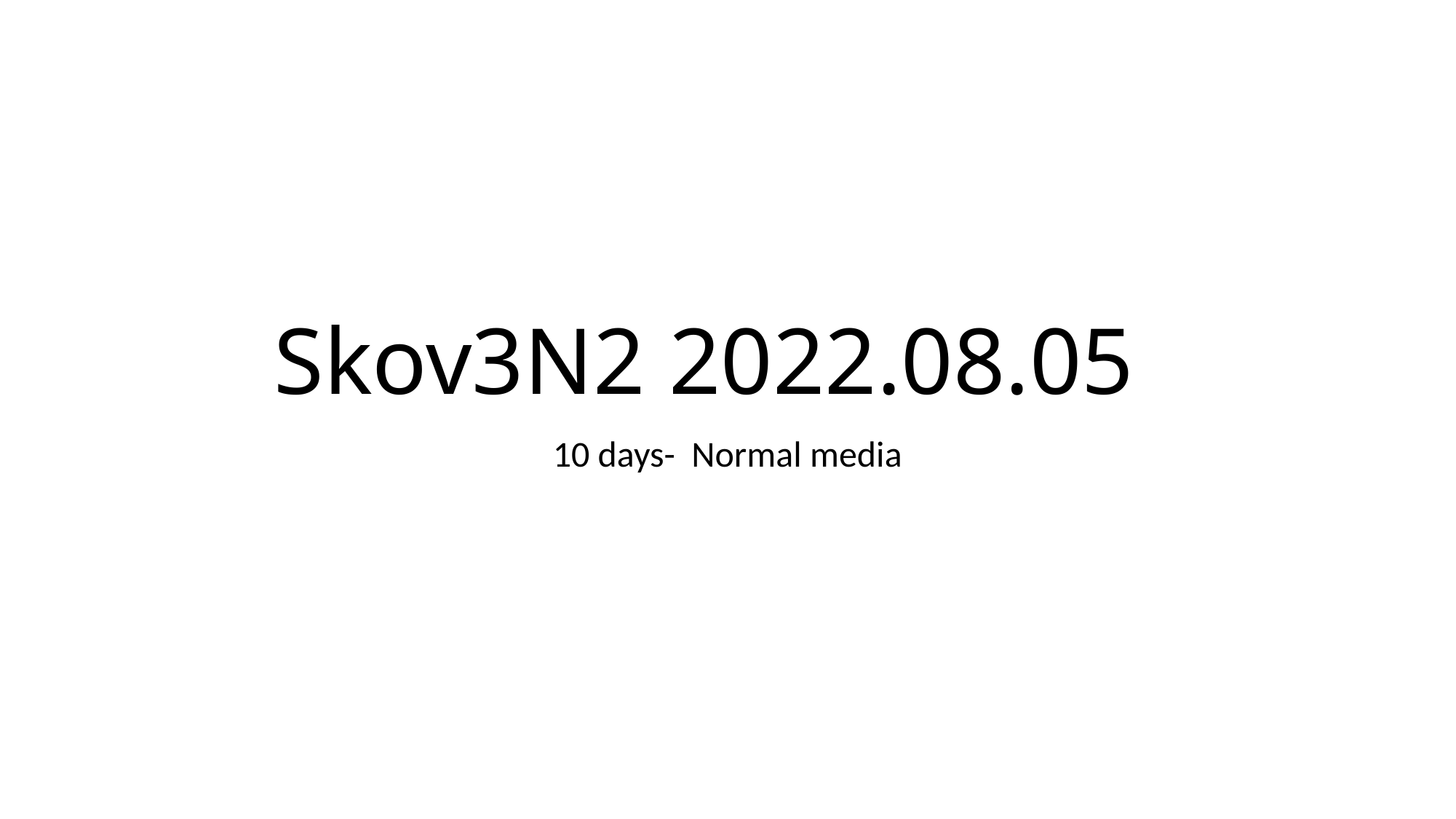

# Skov3N2 2022.08.05
10 days- Normal media

## Slide 6
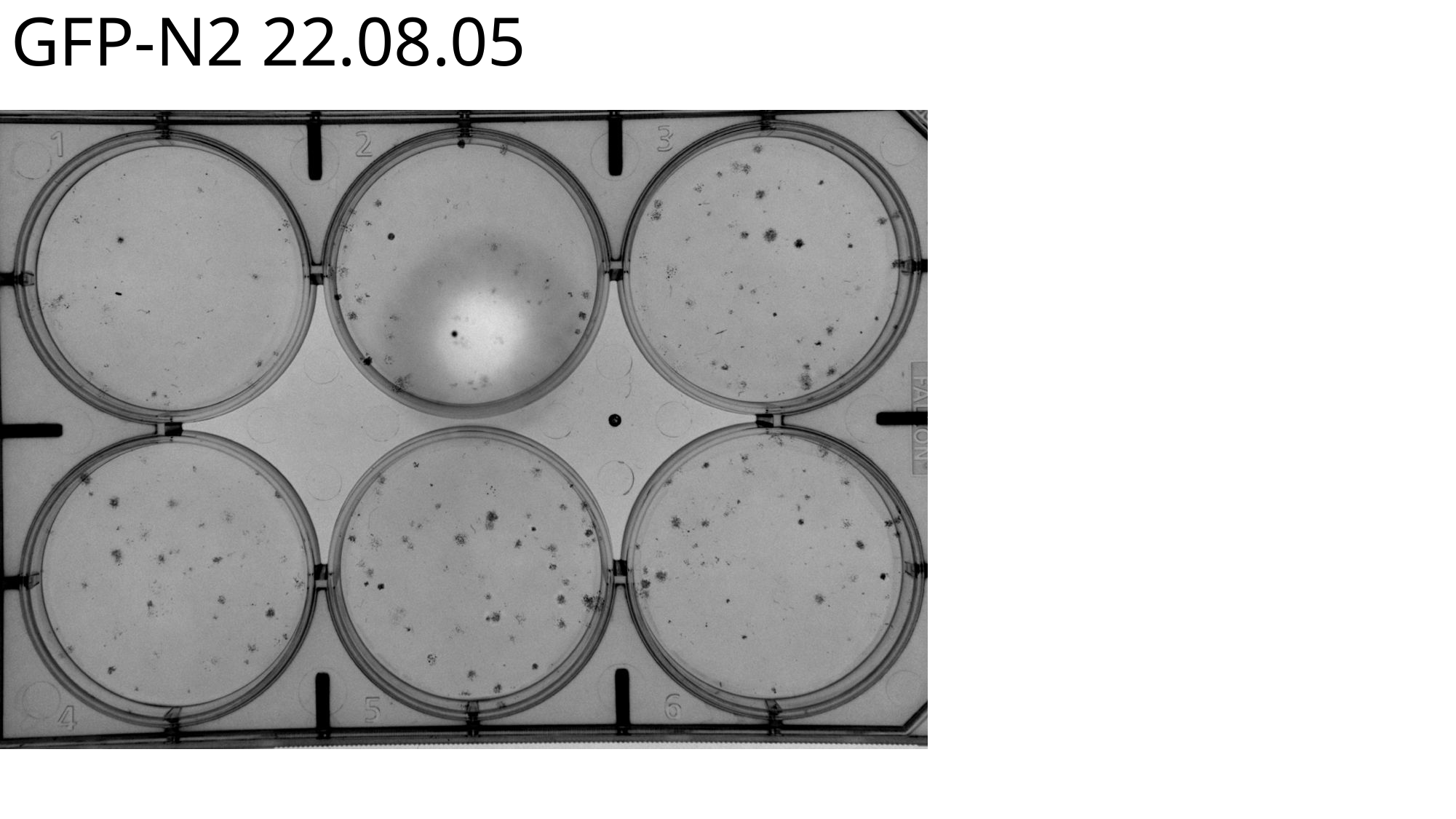

# GFP-N2 22.08.05

## Slide 7
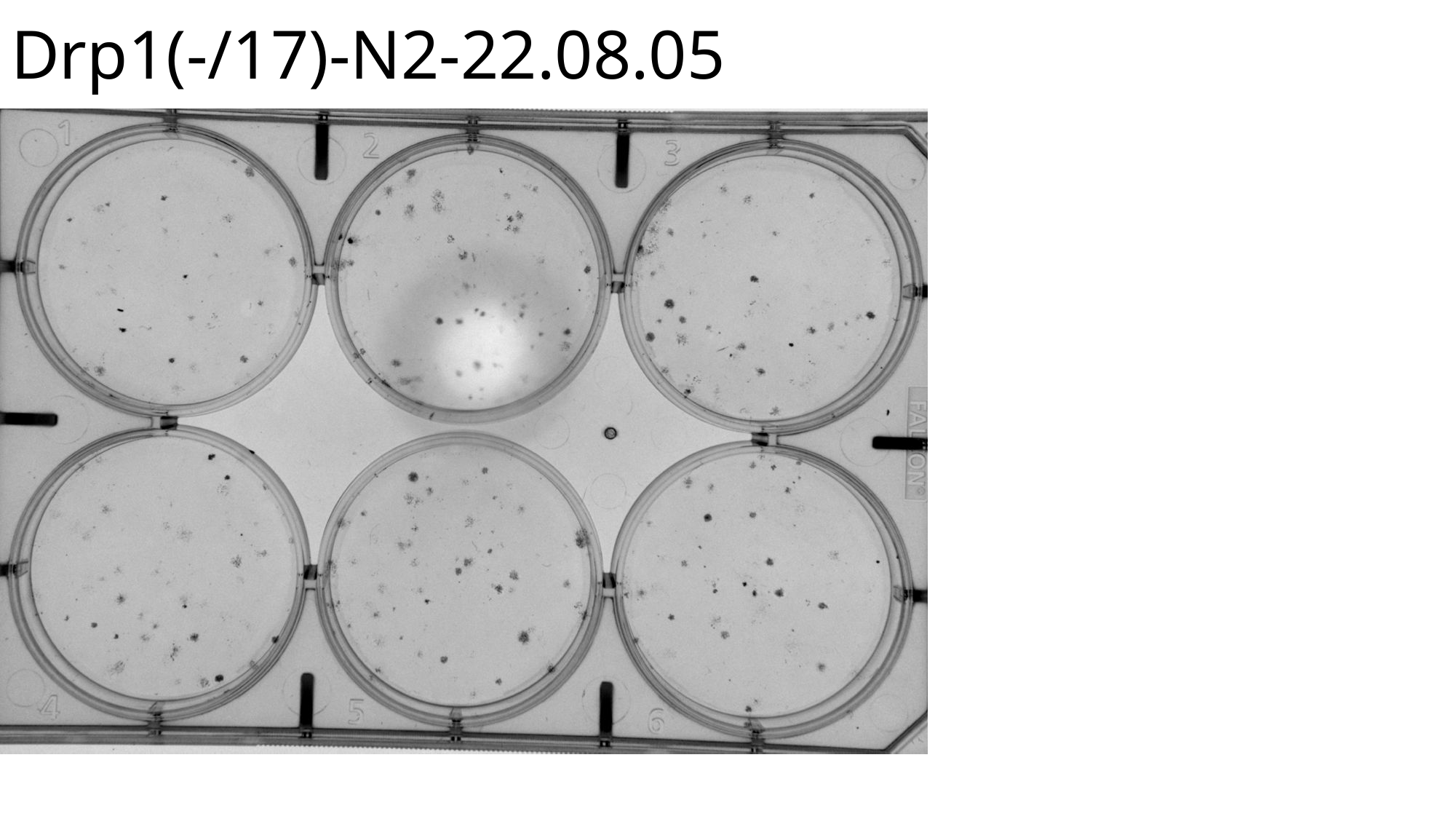

# Drp1(-/17)-N2-22.08.05

## Slide 8
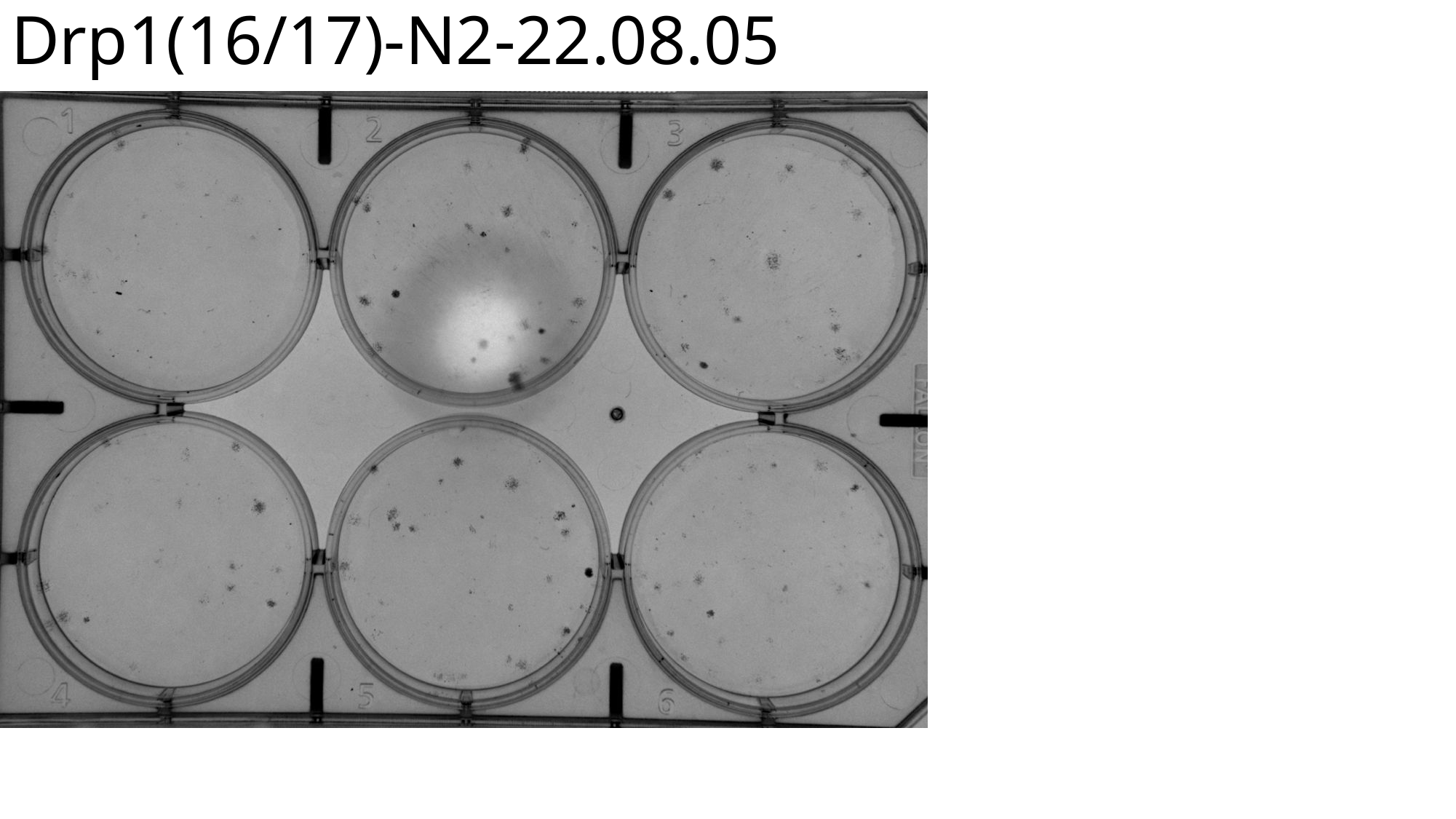

# Drp1(16/17)-N2-22.08.05

## Slide 9
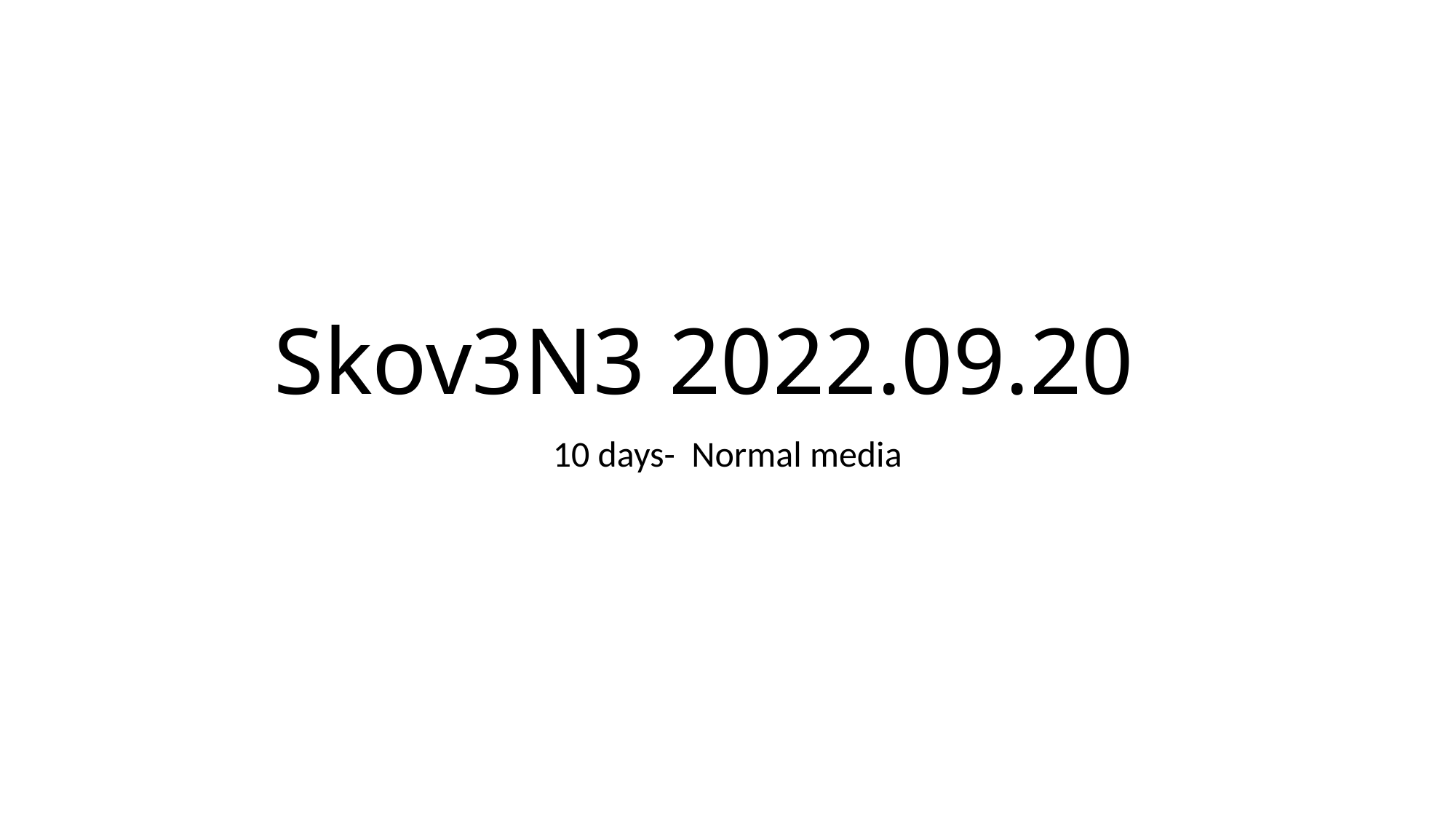

# Skov3N3 2022.09.20
10 days- Normal media

## Slide 10
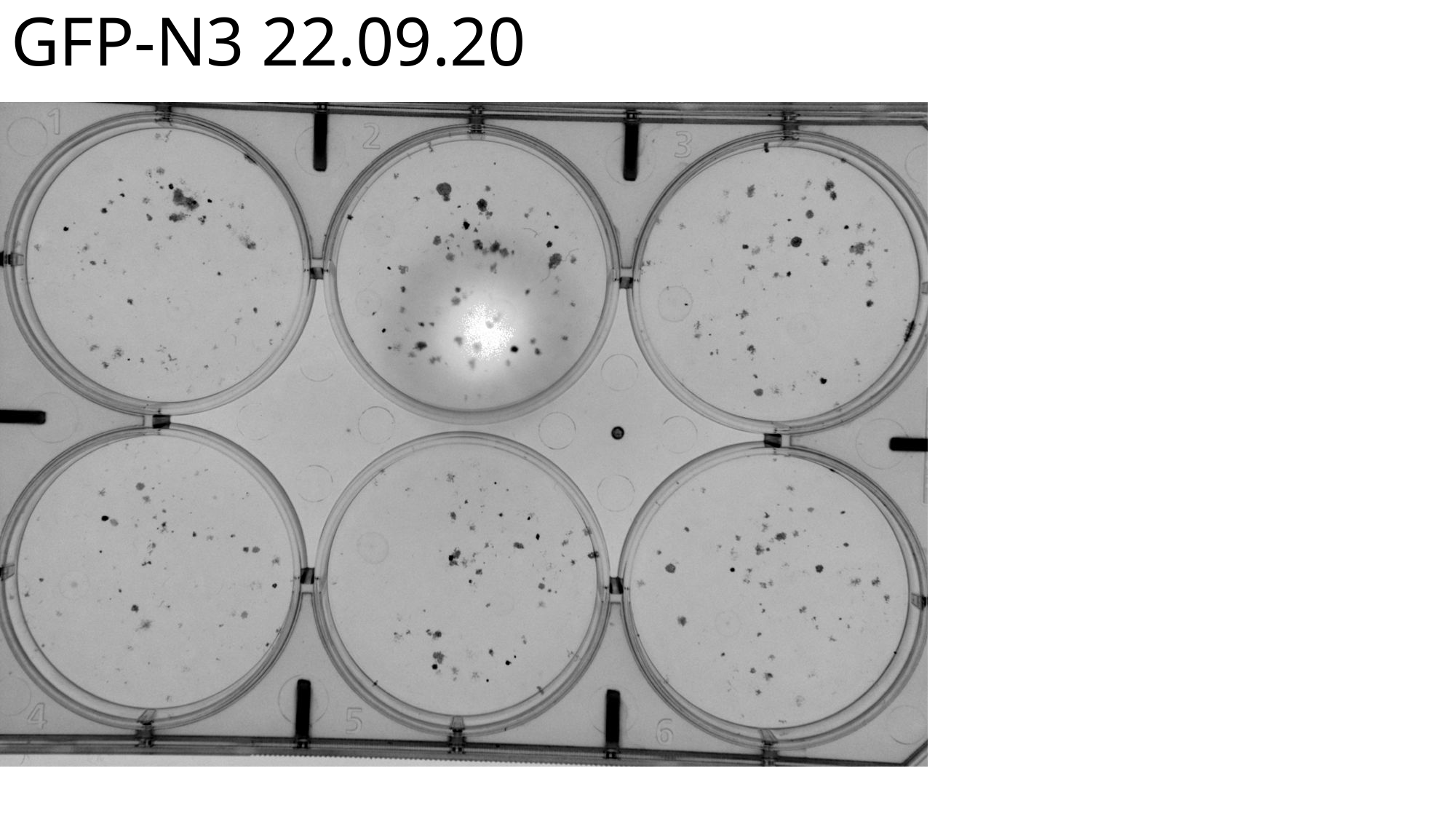

# GFP-N3 22.09.20

## Slide 11
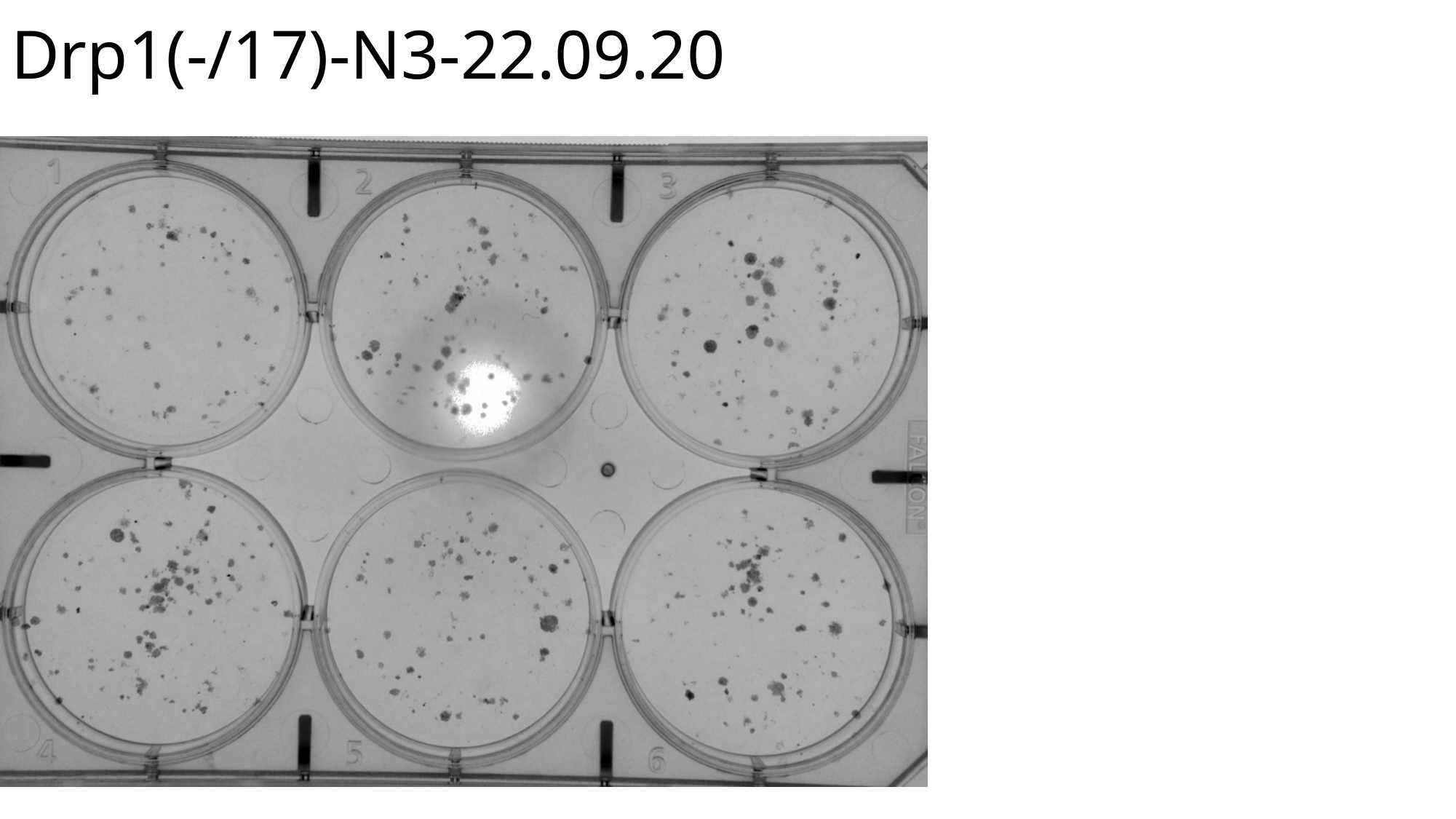

# Drp1(-/17)-N3-22.09.20

## Slide 12
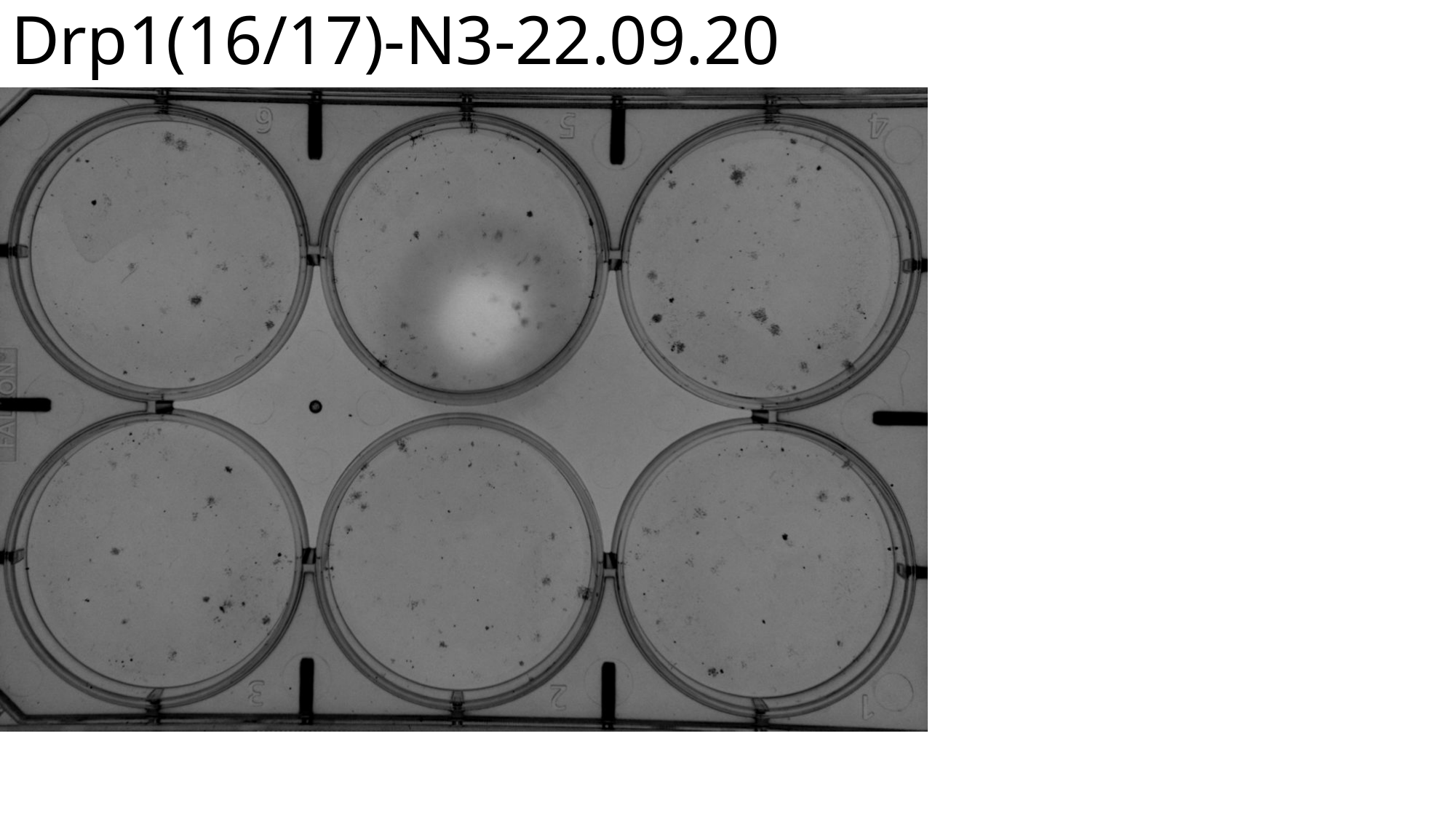

# Drp1(16/17)-N3-22.09.20
