# Supplementary material for: Drp1 splice variants regulate ovarian cancer mitochondrial dynamics and tumor progression
Source: EMBO Rep. 2024 Aug 27;25(10):16. doi: 10.1038/s44319-024-00232-4 (PMC11467262; doi:10.1038/s44319-024-00232-4)
Supplement: Supplementary file 7 — Source data Fig. 5 [file 44319_2024_232_MOESM7_ESM.zip › Figure 5/5B/5B replicates/OVCA433_Drp1O.E_Clonogencity Replicates.pptx]

## Slide 1
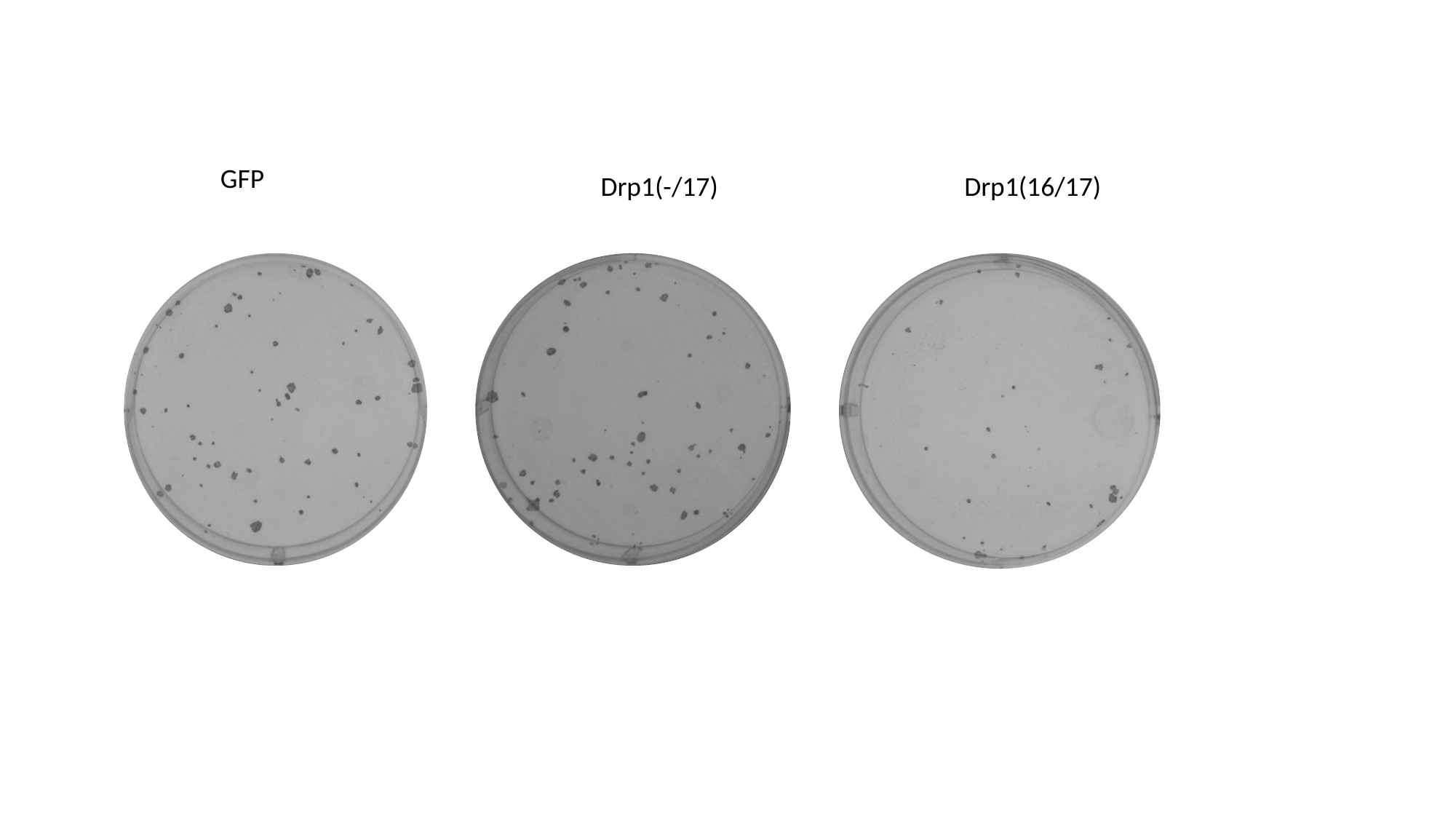

GFP
Drp1(-/17)
Drp1(16/17)

## Slide 2
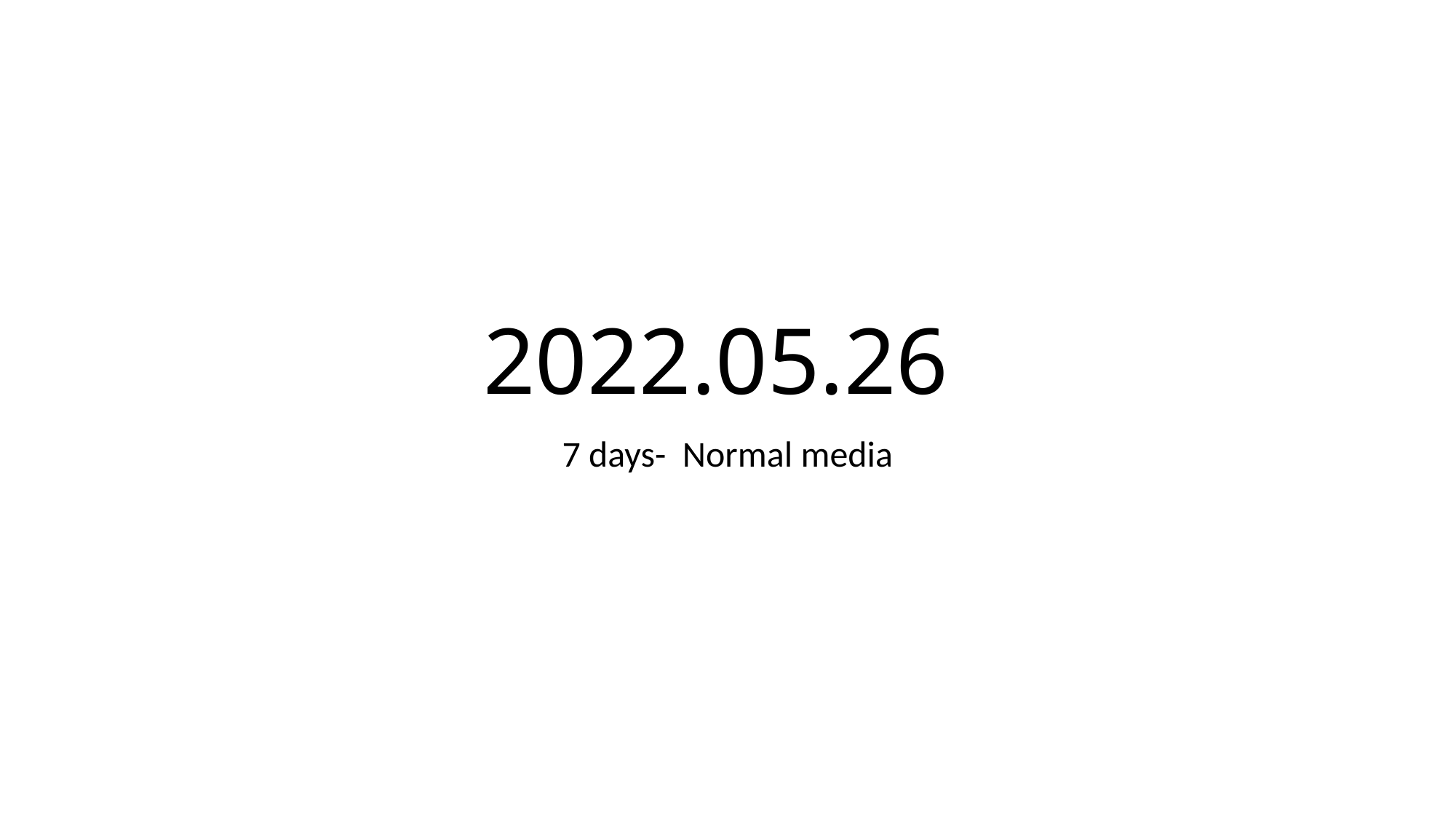

# 2022.05.26
7 days- Normal media

## Slide 3
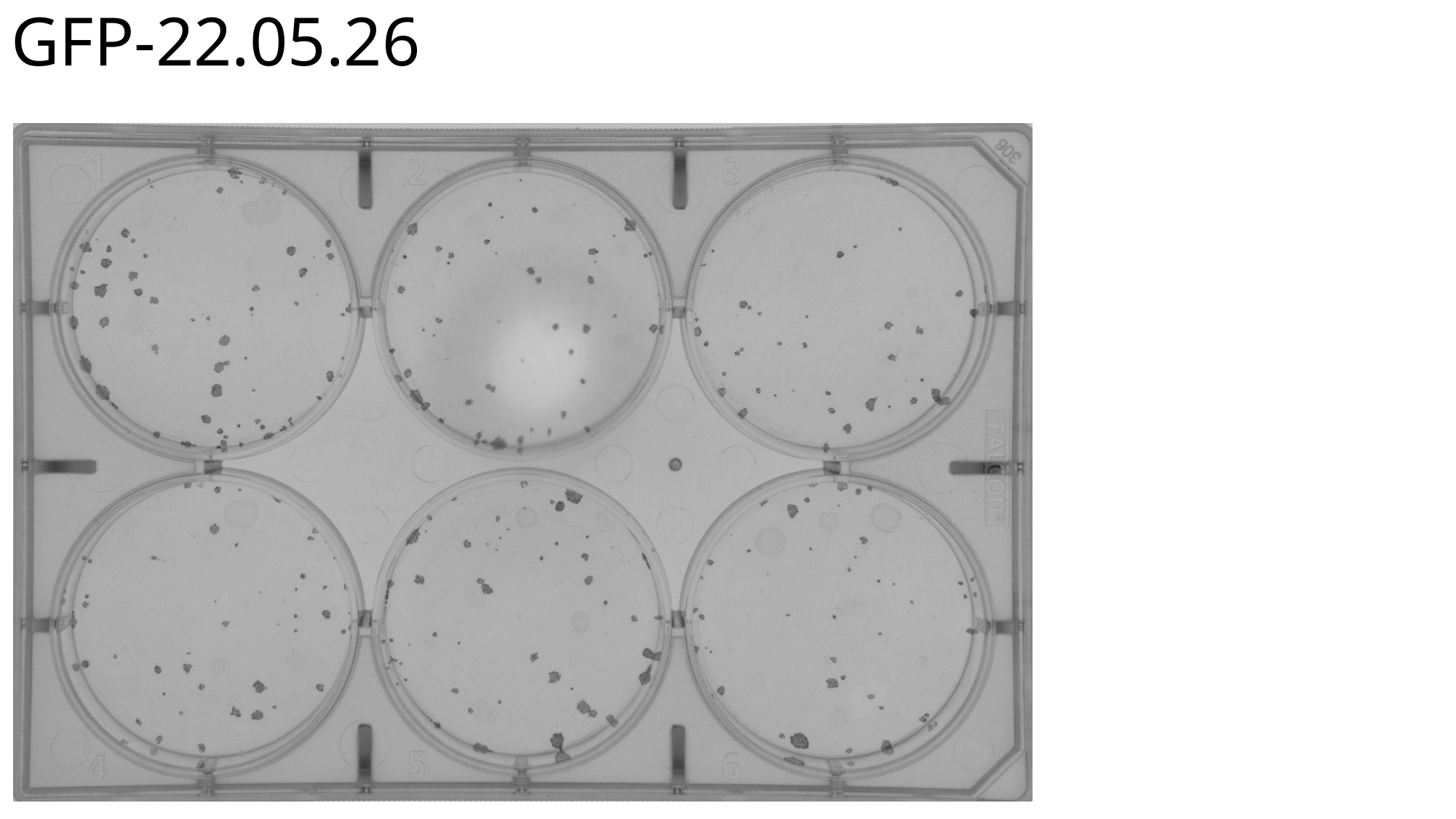

# GFP-22.05.26

## Slide 4
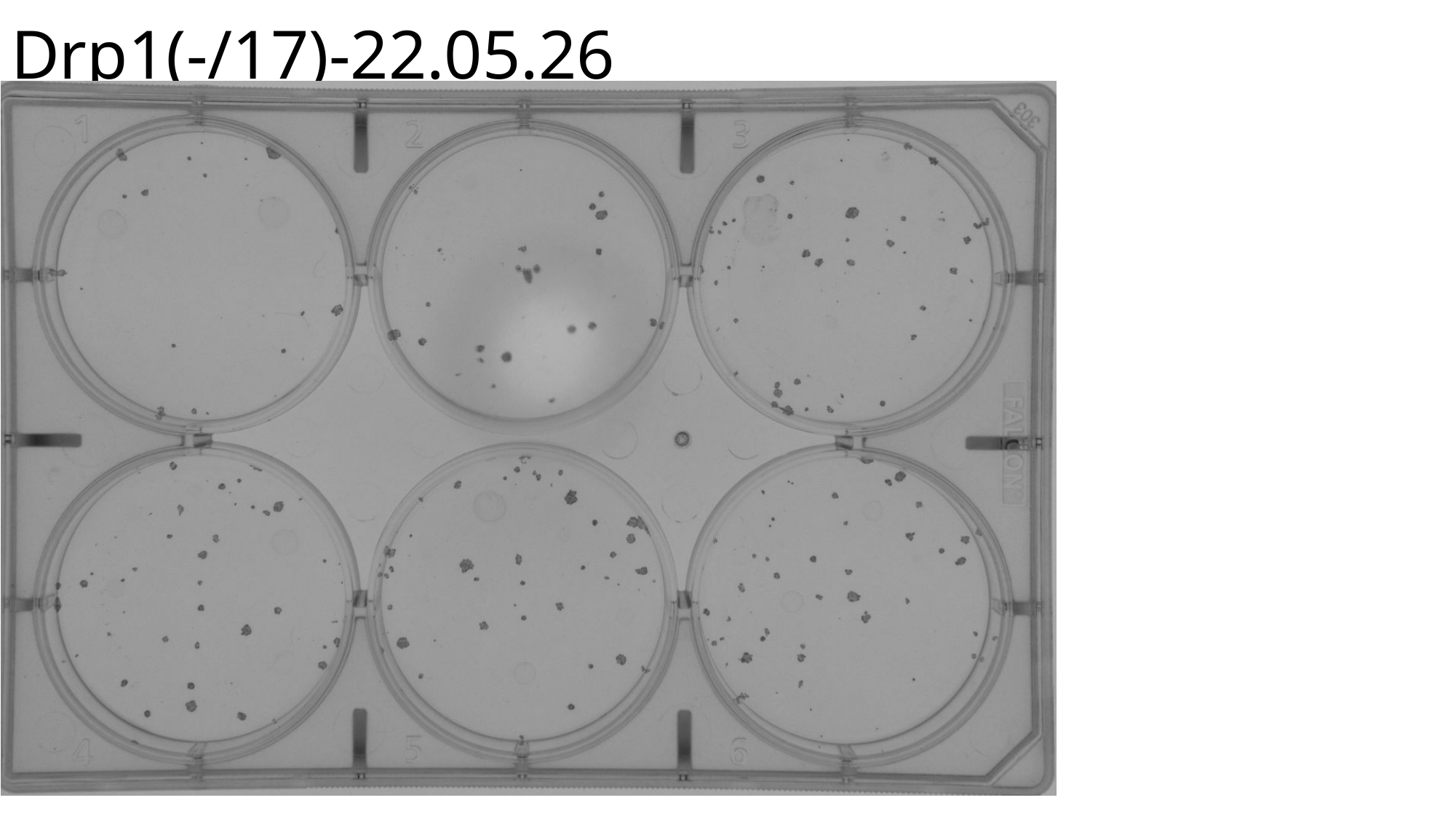

# Drp1(-/17)-22.05.26

## Slide 5
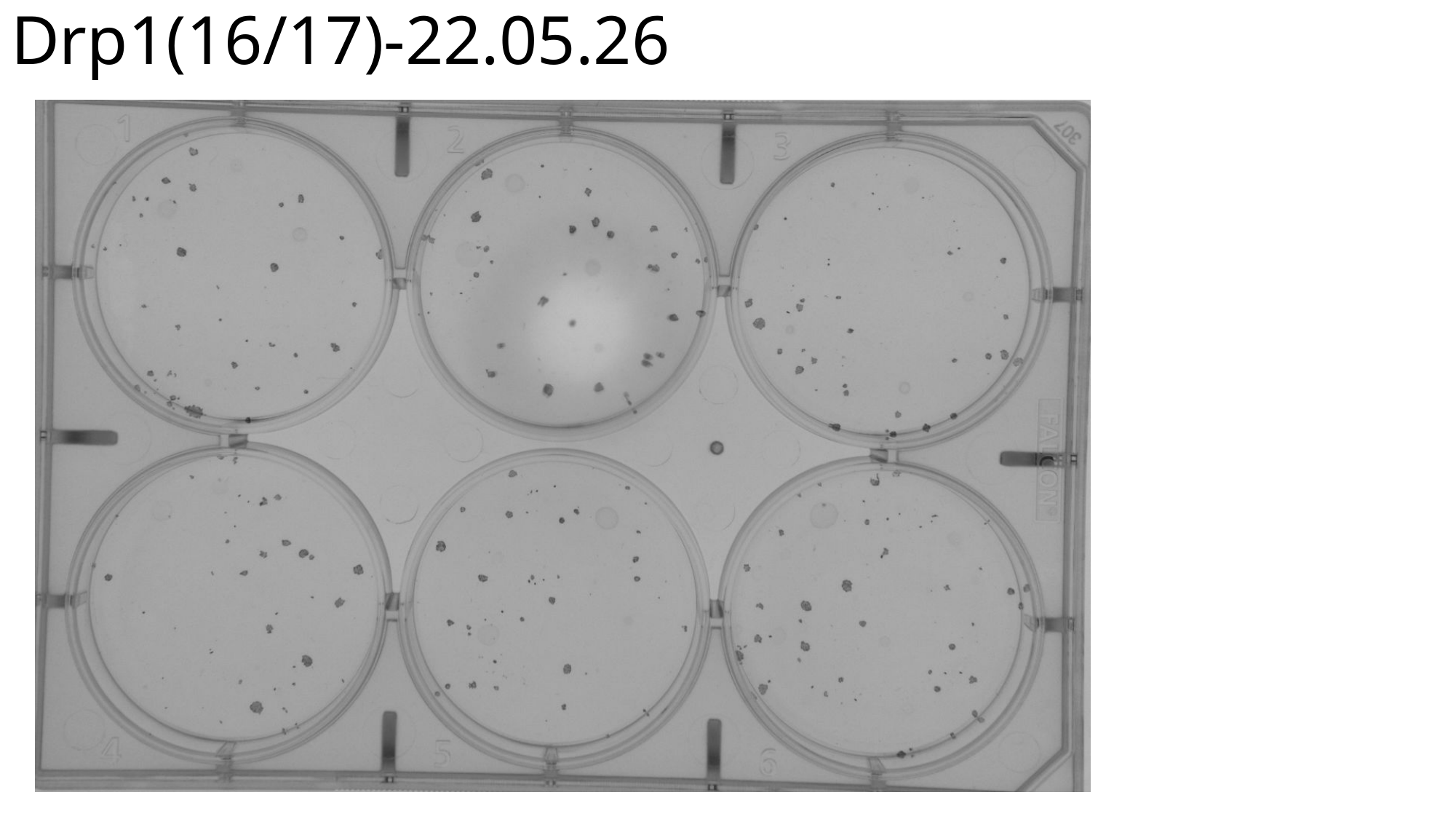

# Drp1(16/17)-22.05.26

## Slide 6
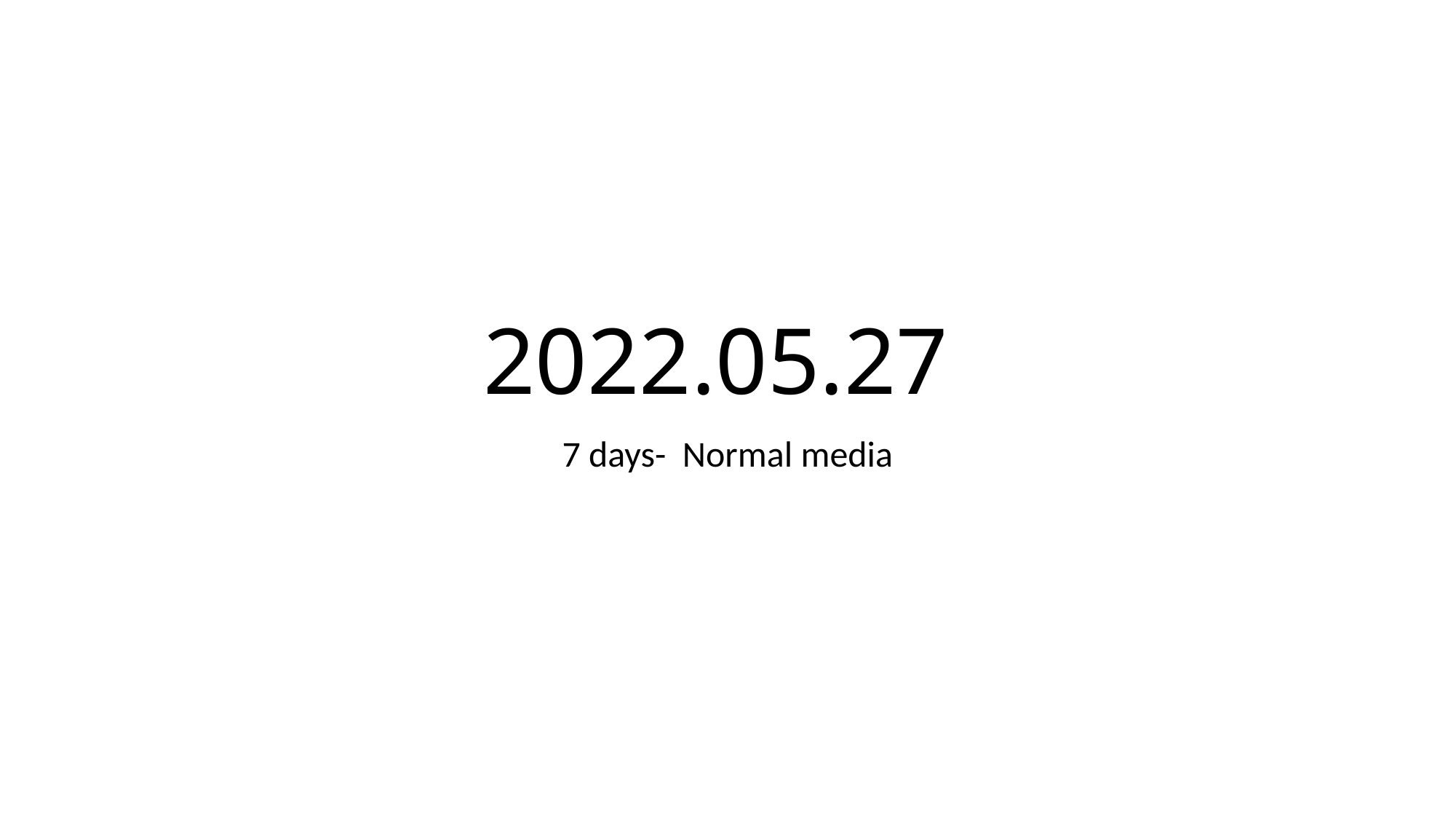

# 2022.05.27
7 days- Normal media

## Slide 7
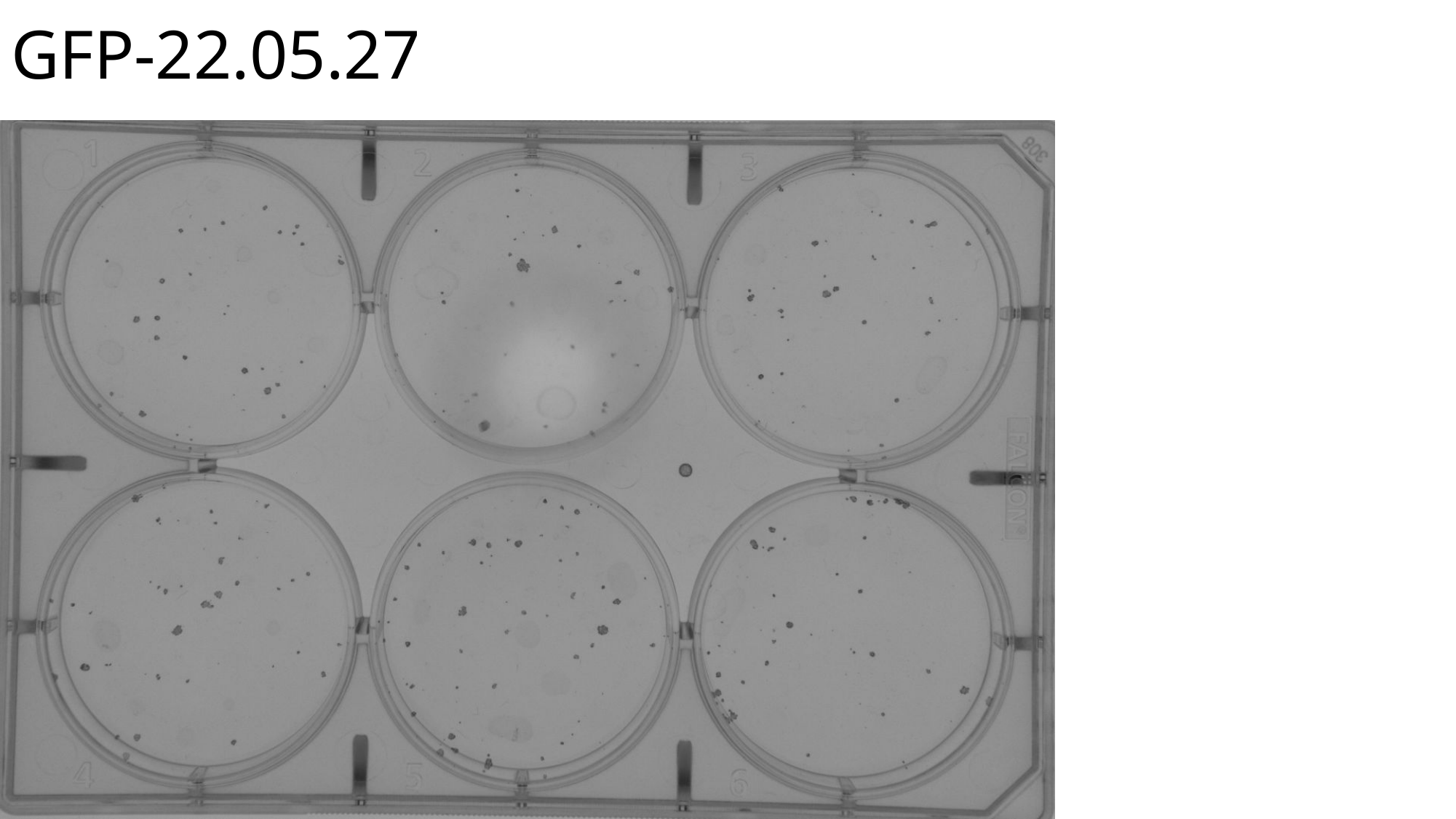

GFP-22.05.27

## Slide 8
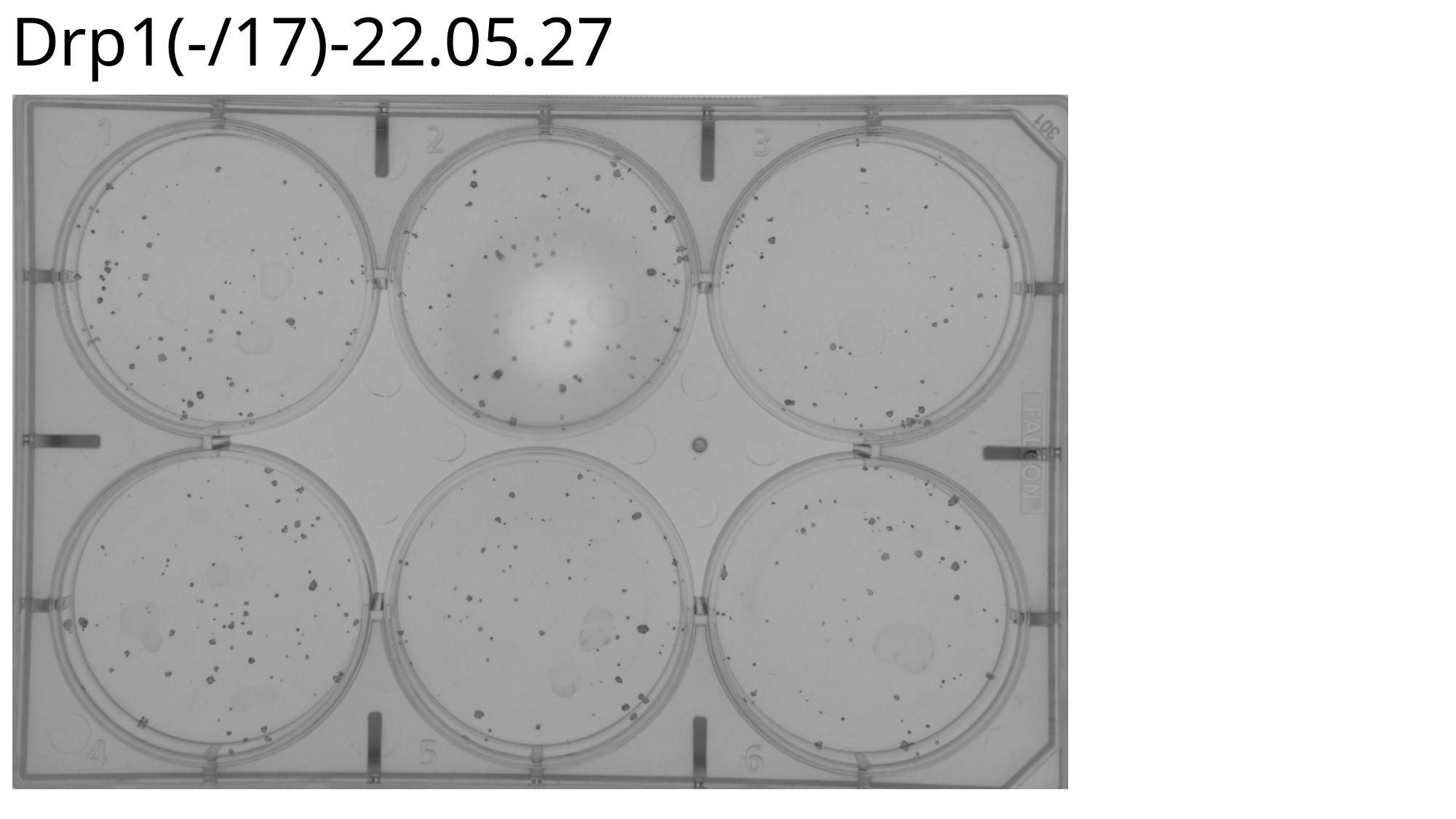

Drp1(-/17)-22.05.27

## Slide 9
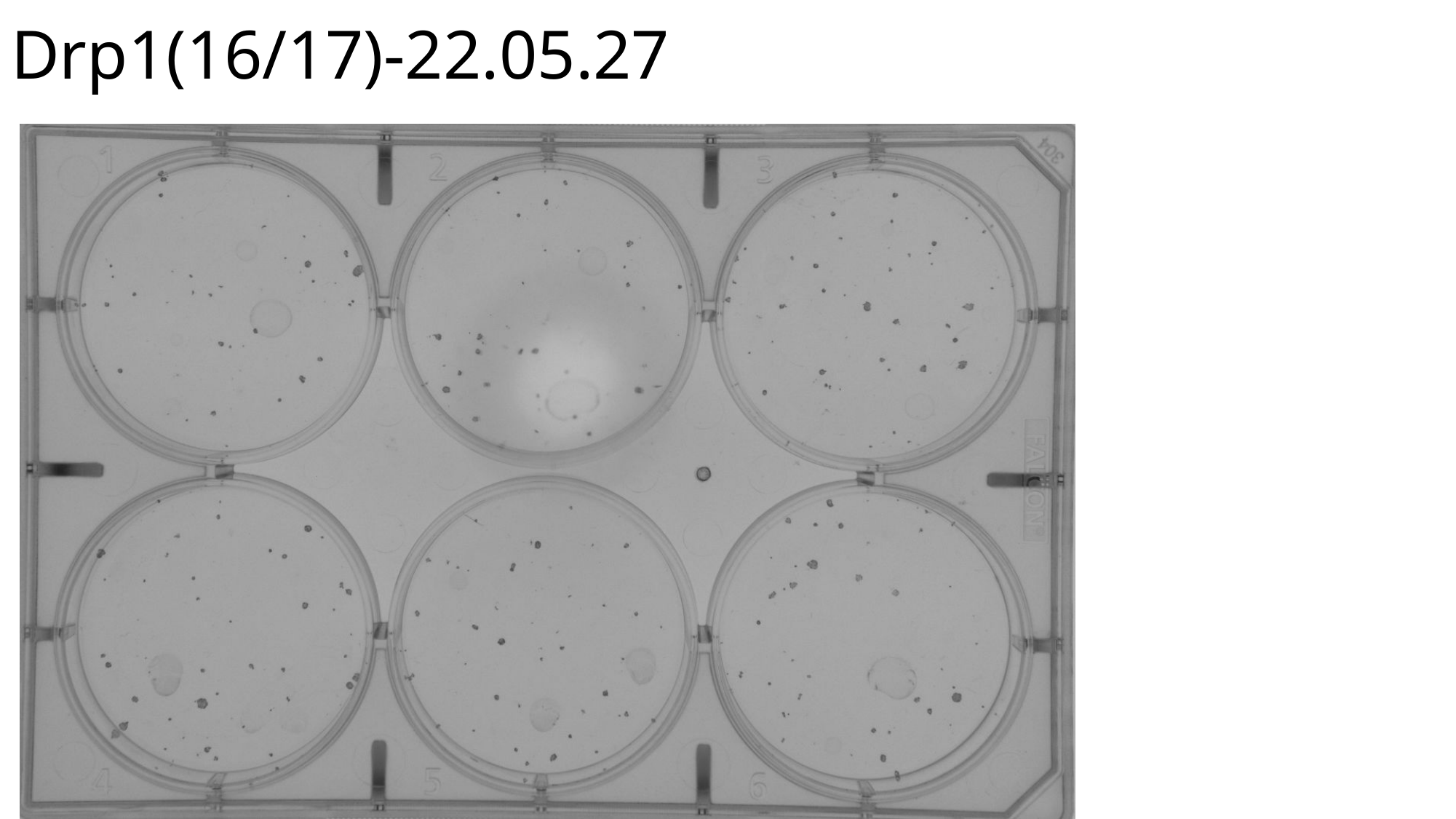

Drp1(16/17)-22.05.27

## Slide 10
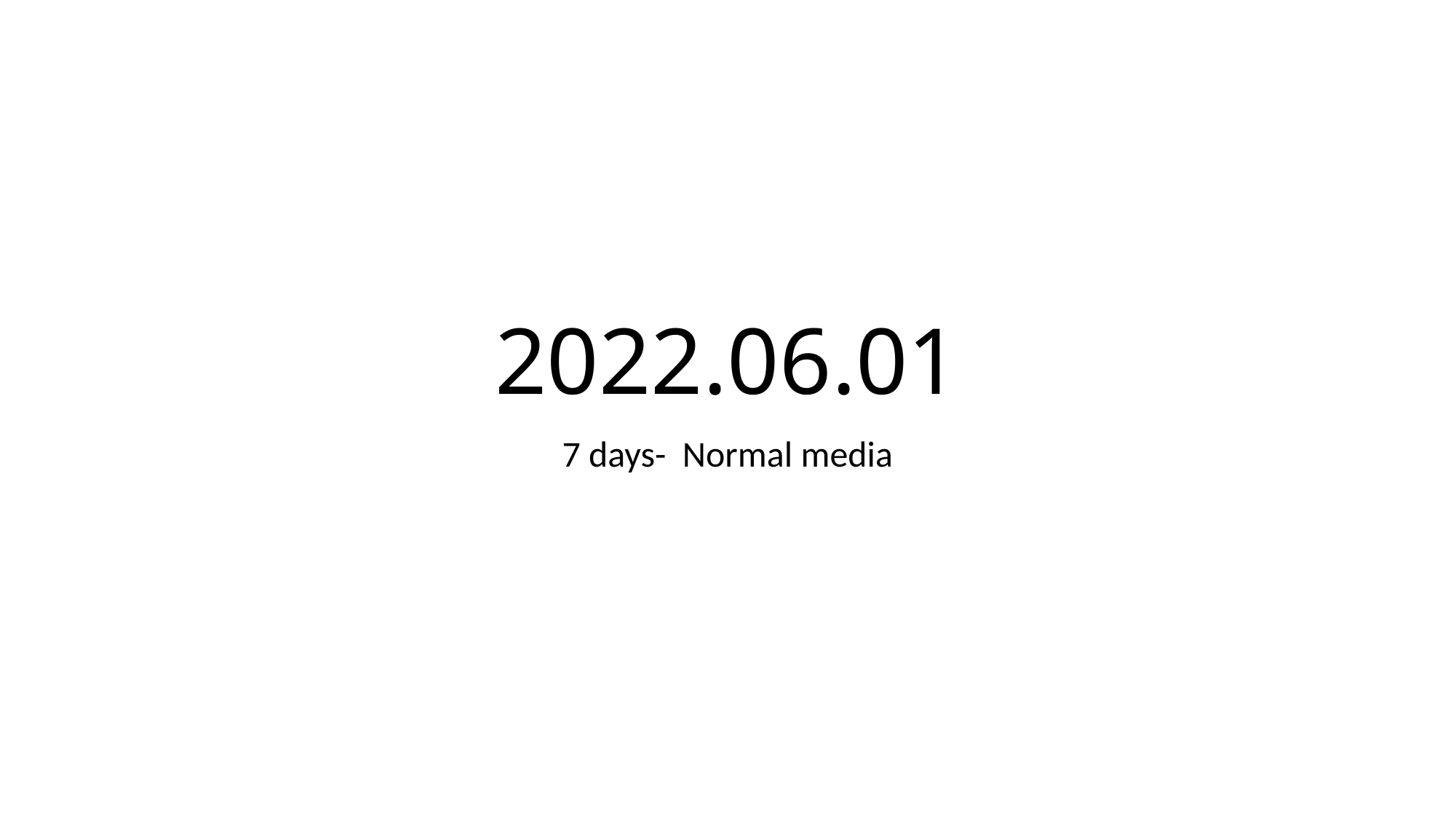

# 2022.06.01
7 days- Normal media

## Slide 11
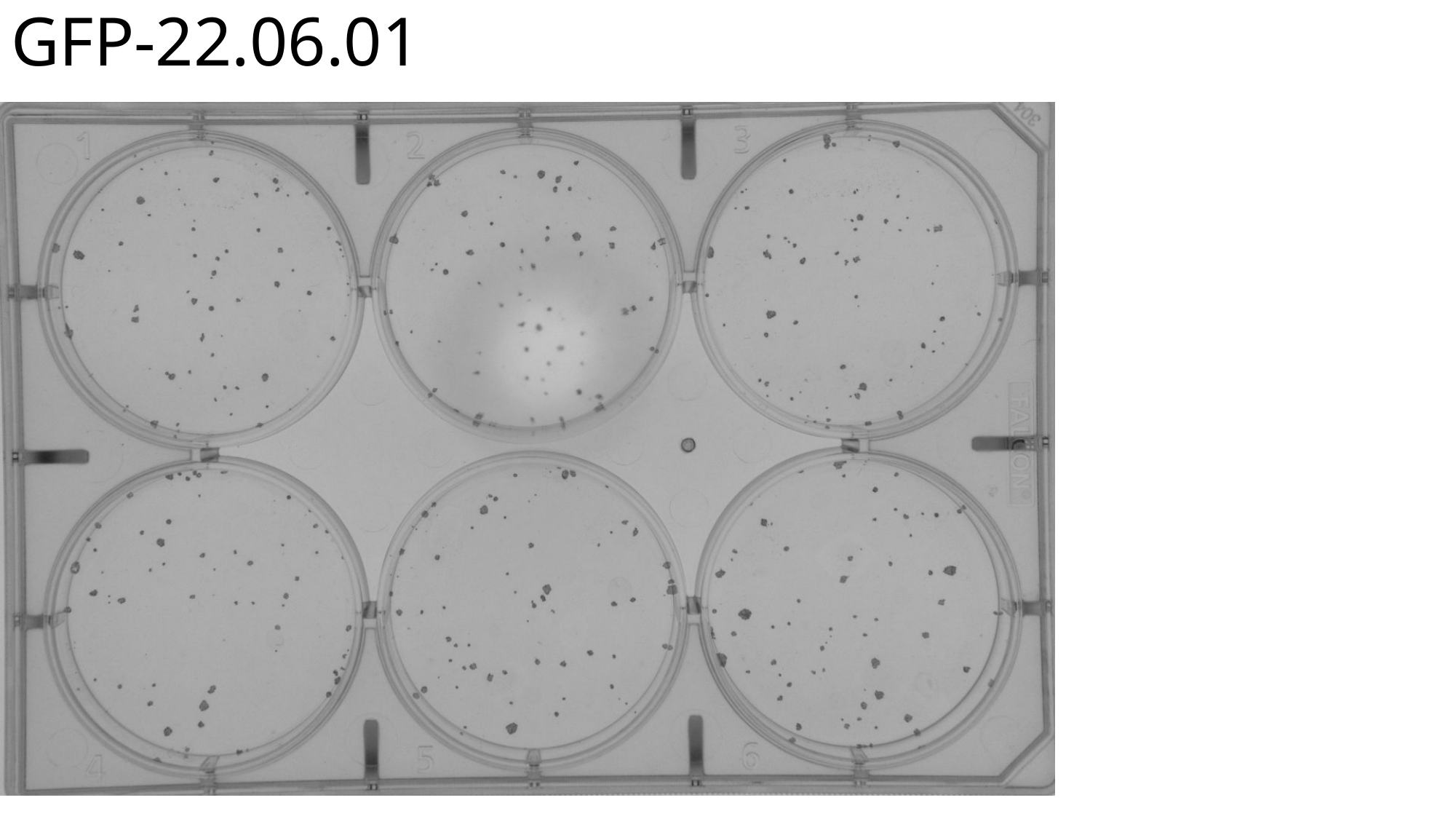

GFP-22.06.01

## Slide 12
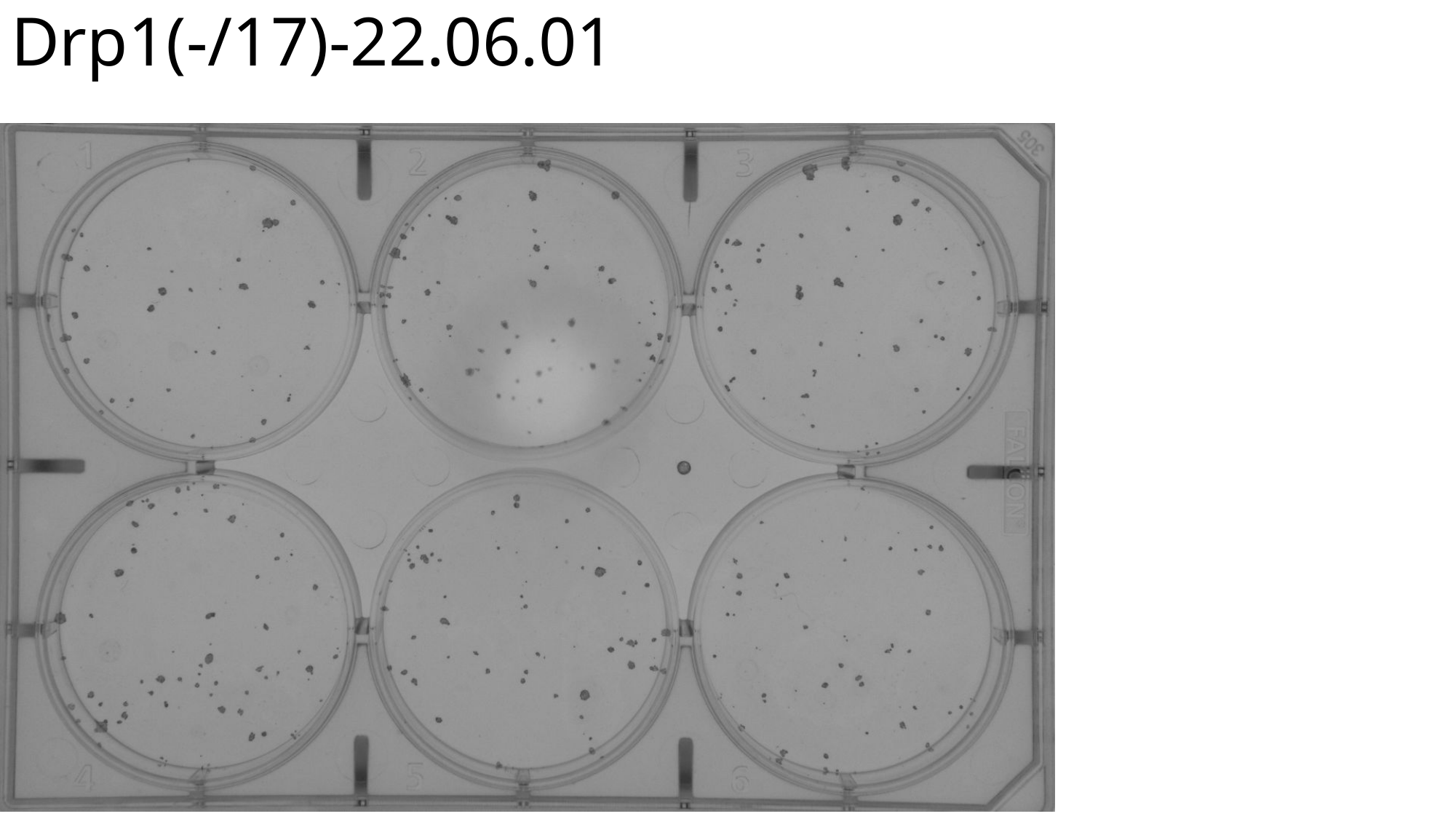

Drp1(-/17)-22.06.01

## Slide 13
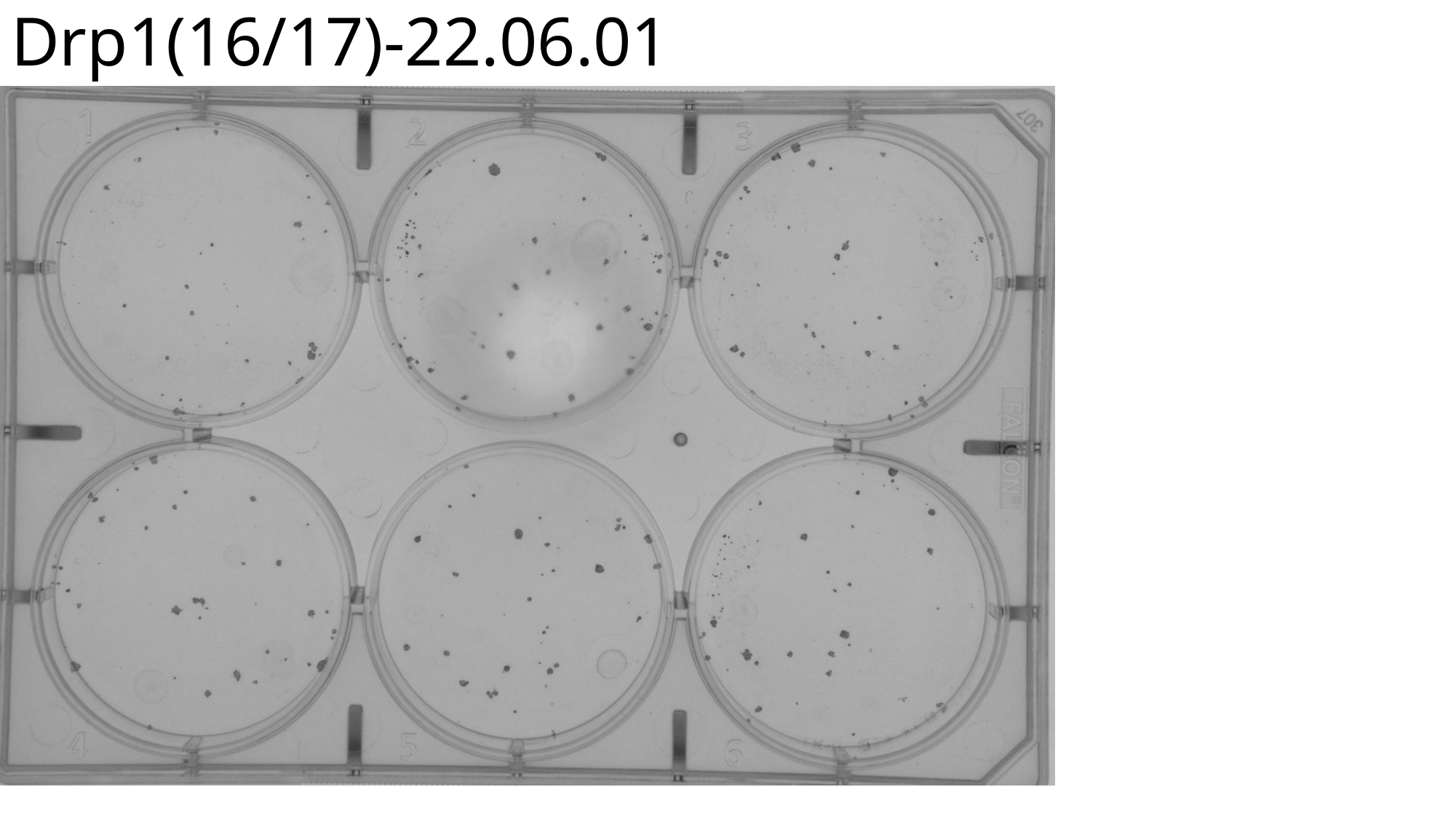

Drp1(16/17)-22.06.01
