# Supplementary material for: Drp1 splice variants regulate ovarian cancer mitochondrial dynamics and tumor progression
Source: EMBO Rep. 2024 Aug 27;25(10):16. doi: 10.1038/s44319-024-00232-4 (PMC11467262; doi:10.1038/s44319-024-00232-4)
Supplement: Supplementary file 10 — Source data Fig. 8 [file 44319_2024_232_MOESM10_ESM.zip › Figure 8/8G/replicates/8G_siDNM1L migration Assay Reps.pptx]

## Slide 1
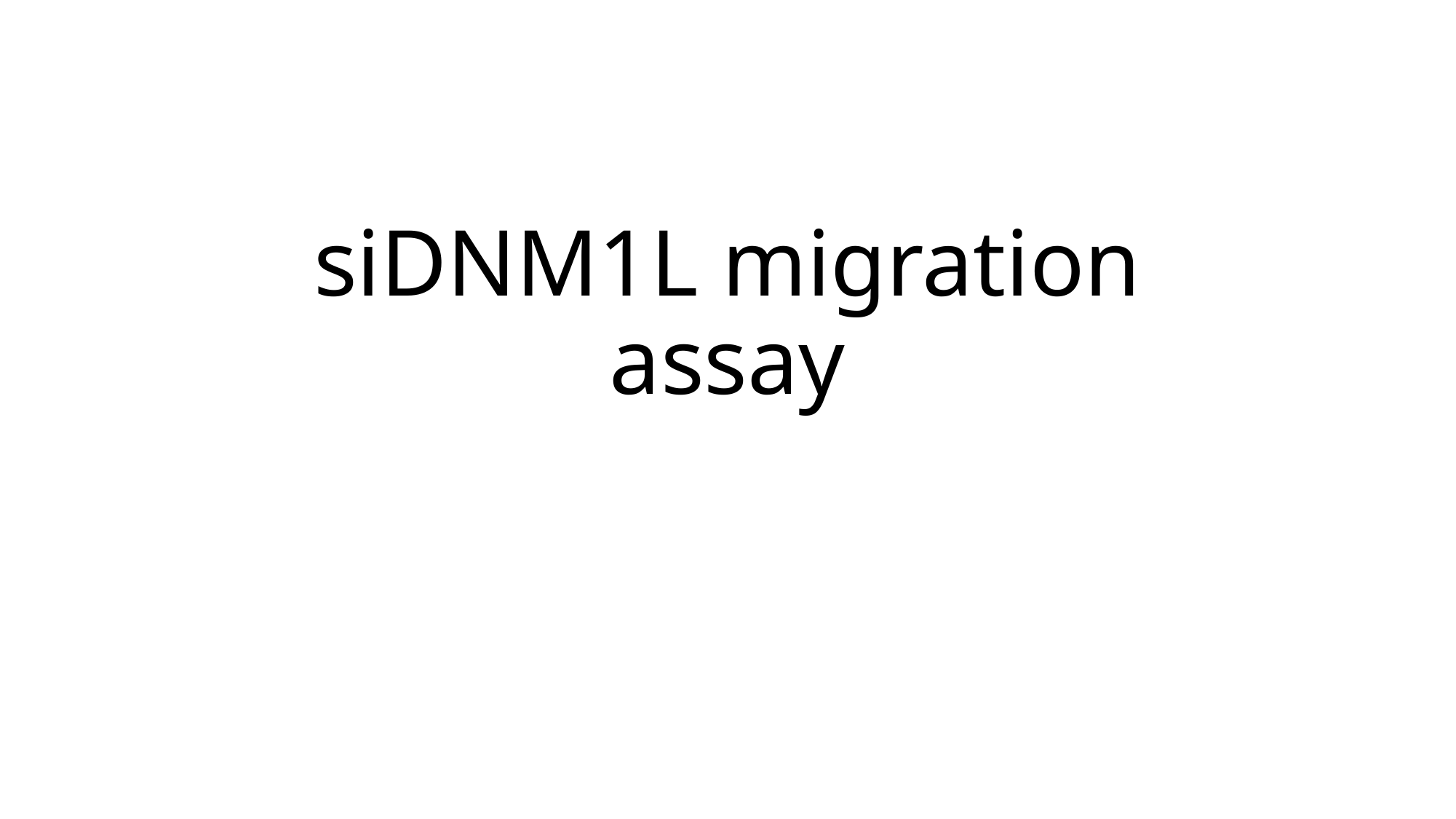

# siDNM1L migration assay

## Slide 2
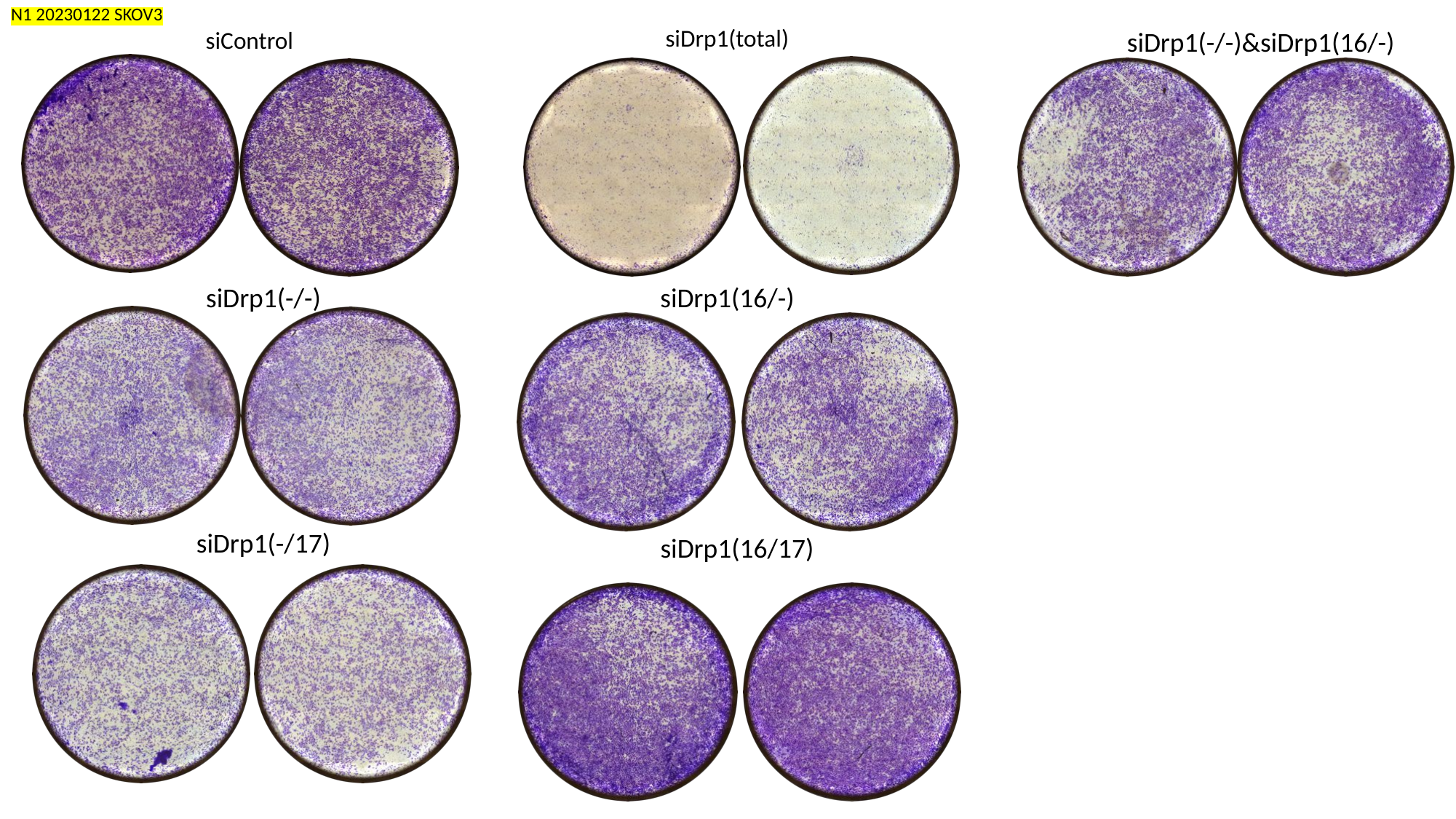

N1 20230122 SKOV3
siDrp1(total)
siControl
siDrp1(-/-)&siDrp1(16/-)
siDrp1(-/-)
siDrp1(16/-)
siDrp1(-/17)
siDrp1(16/17)

## Slide 3
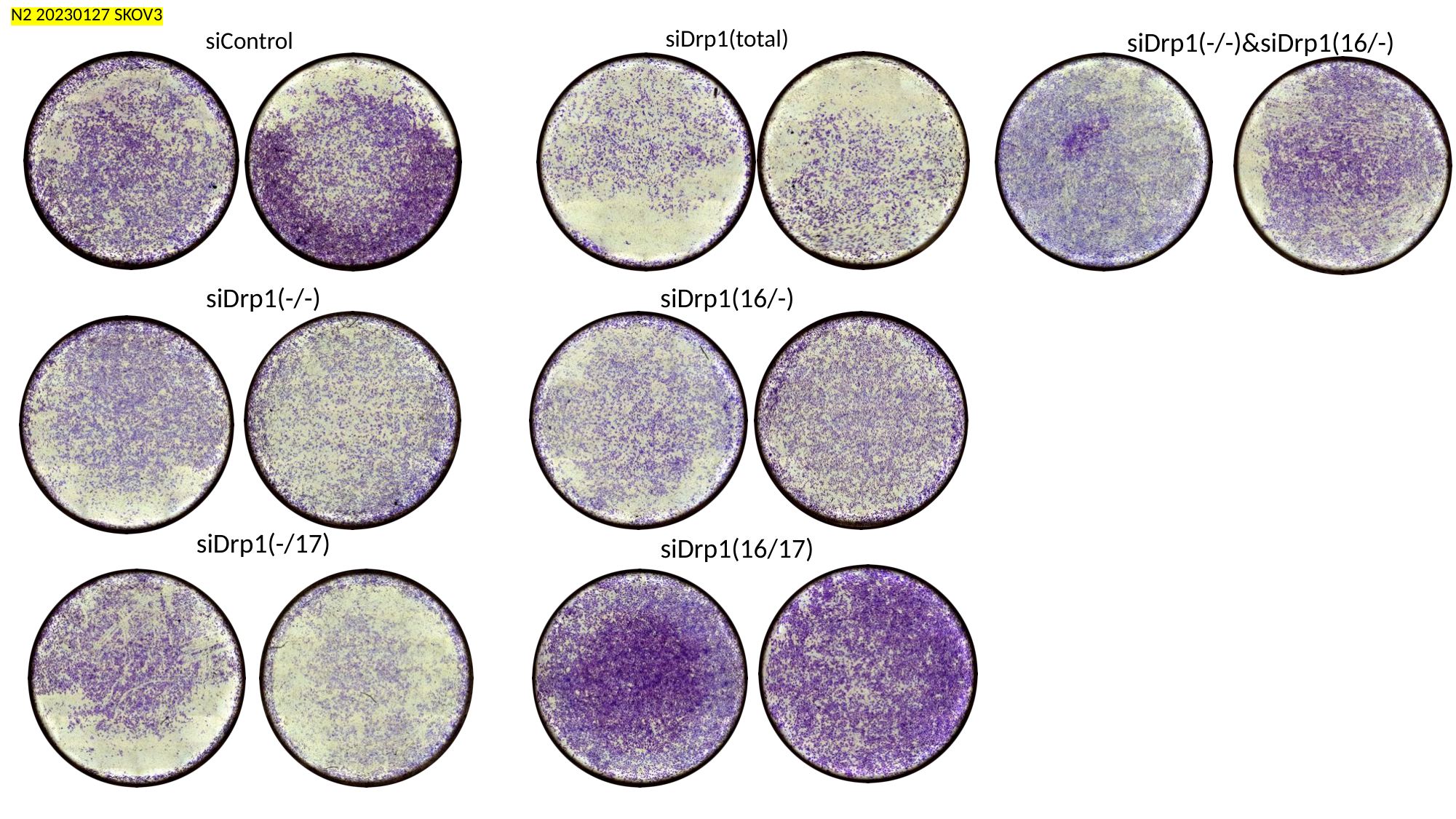

N2 20230127 SKOV3
siDrp1(total)
siControl
siDrp1(-/-)&siDrp1(16/-)
siDrp1(-/-)
siDrp1(16/-)
siDrp1(-/17)
siDrp1(16/17)

## Slide 4
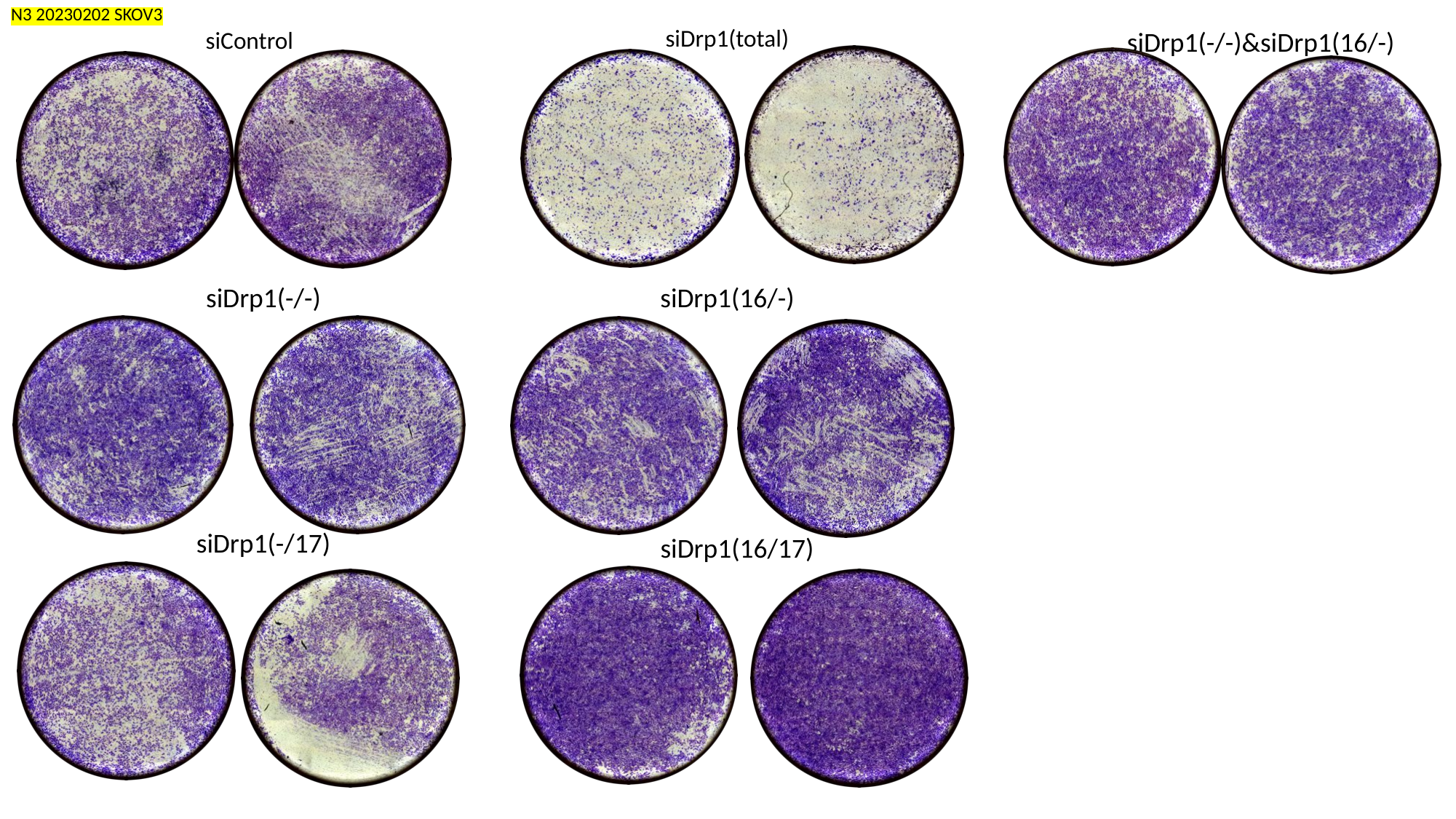

N3 20230202 SKOV3
siDrp1(total)
siControl
siDrp1(-/-)&siDrp1(16/-)
siDrp1(-/-)
siDrp1(16/-)
siDrp1(-/17)
siDrp1(16/17)

## Slide 5
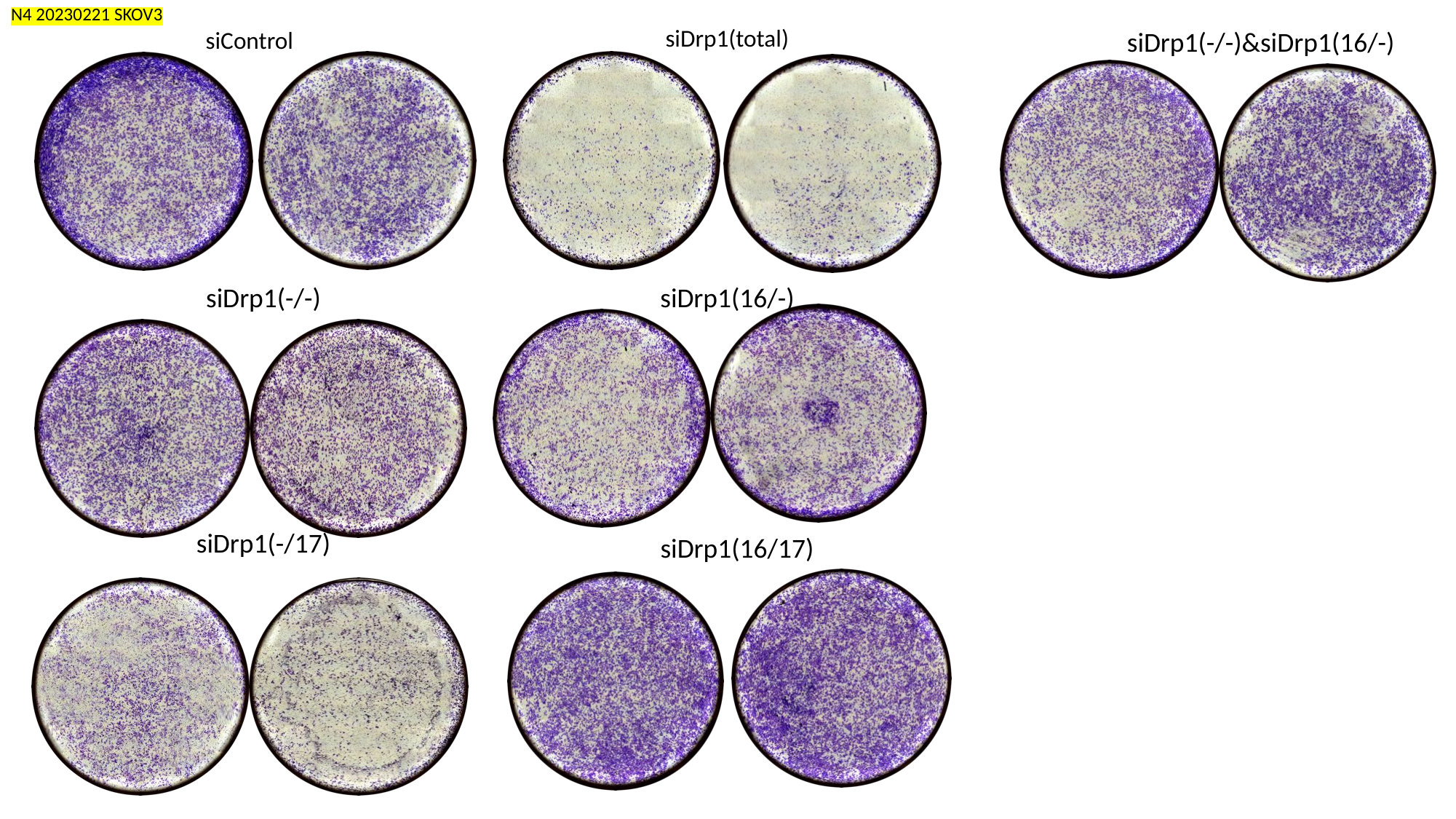

N4 20230221 SKOV3
siDrp1(total)
siControl
siDrp1(-/-)&siDrp1(16/-)
siDrp1(-/-)
siDrp1(16/-)
siDrp1(-/17)
siDrp1(16/17)
