# Supplementary material for: Drp1 splice variants regulate ovarian cancer mitochondrial dynamics and tumor progression
Source: EMBO Rep. 2024 Aug 27;25(10):16. doi: 10.1038/s44319-024-00232-4 (PMC11467262; doi:10.1038/s44319-024-00232-4)
Supplement: Supplementary file 11 — Expanded View Figures [file 44319_2024_232_MOESM11_ESM.pdf]

Expanded View Figures

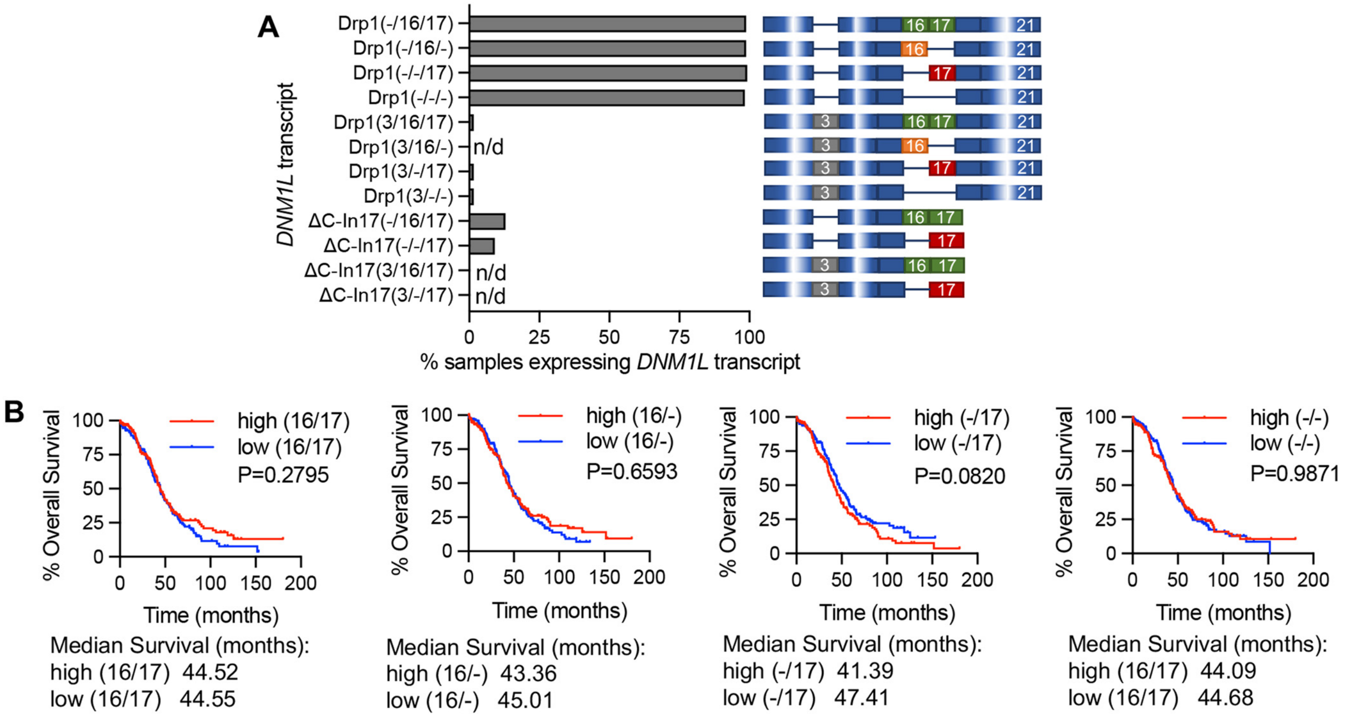

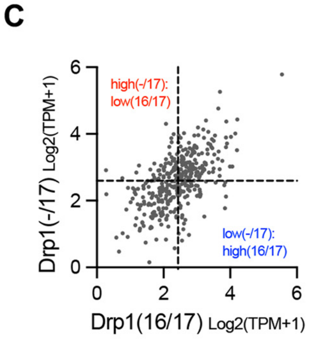

D

Splice variant expression	Median Survival (months)	HR (log rank)	95% CI	P value (Log-rank, Mantel-Cox)
high(-/17):low(16/17) vs low(-/17):high(16/17)	44.84<br>59.14	1.818	1.076 to 3.071	0.0208
high(-/17):low(16/-) vs low(-/17):high(16/-)	43.53<br>55.23	1.508	0.9304 to 2.444	0.0880
high(-/17):low(-/-) vs low(-/17):high(-/-)	38.20<br>54.11	1.494	0.8658 to 2.578	0.1443
high(-/-):low(16/17) vs low(-/-):high(16/17)	44.84<br>47.54	1.274	0.7936 to 2.045	0.3122
high(16/-):low(16/17) vs low(16/-):high(16/17)	38.20<br>45.50	1.155	0.7074 to 1.885	0.5610
high(-/-):low(16/-) vs low(-/-):high(16/-)	44.32<br>42.05	1.115	0.6898 to 1.802	0.6532

Figure EV1. Drp1/*DNM1L* transcript variant expression in ovarian cancer specimens from TCGA.

(**A**) Frequency of Drp1/*DNM1L* transcript variant expression, including alternatively spliced exons 3, 16, and 17 (3/16/17) transcripts and C-terminal truncation terminating in Intron 17 (ΔC-In17). Dash denotes exon is spliced out. Data represent the percentage of specimens displaying log2 TPM + 1 values >0.5 for each DNM1L variant. (**B**) Overall survival of TCGA patients based on DNM1L variant expression. Samples were split at median log2 TPM into high ($n = 184$) and low expression ($n = 184$; log-rank Mantel-Cox test). (**C**) Drp1(-/17) expression relative to Drp1(16/17; log2 TPM + 1). Mutually exclusive high and low expression of variant pairs is based on median log2 TPM + 1 expression cut-offs indicated by a dotted line. (**D**) Overall survival data of TCGA ovarian cancer patients grouped into mutually exclusive high/low expression of Drp1 transcript variant pairs. Low and high cutoffs are based on median expression. Patients with high Drp1(-/17) and low Drp1(16/17) expression display significantly decreased overall survival compared to patients with high Drp1(16/17) and low Drp1(-/17) transcript levels in their tumors.

 © The Author(s)

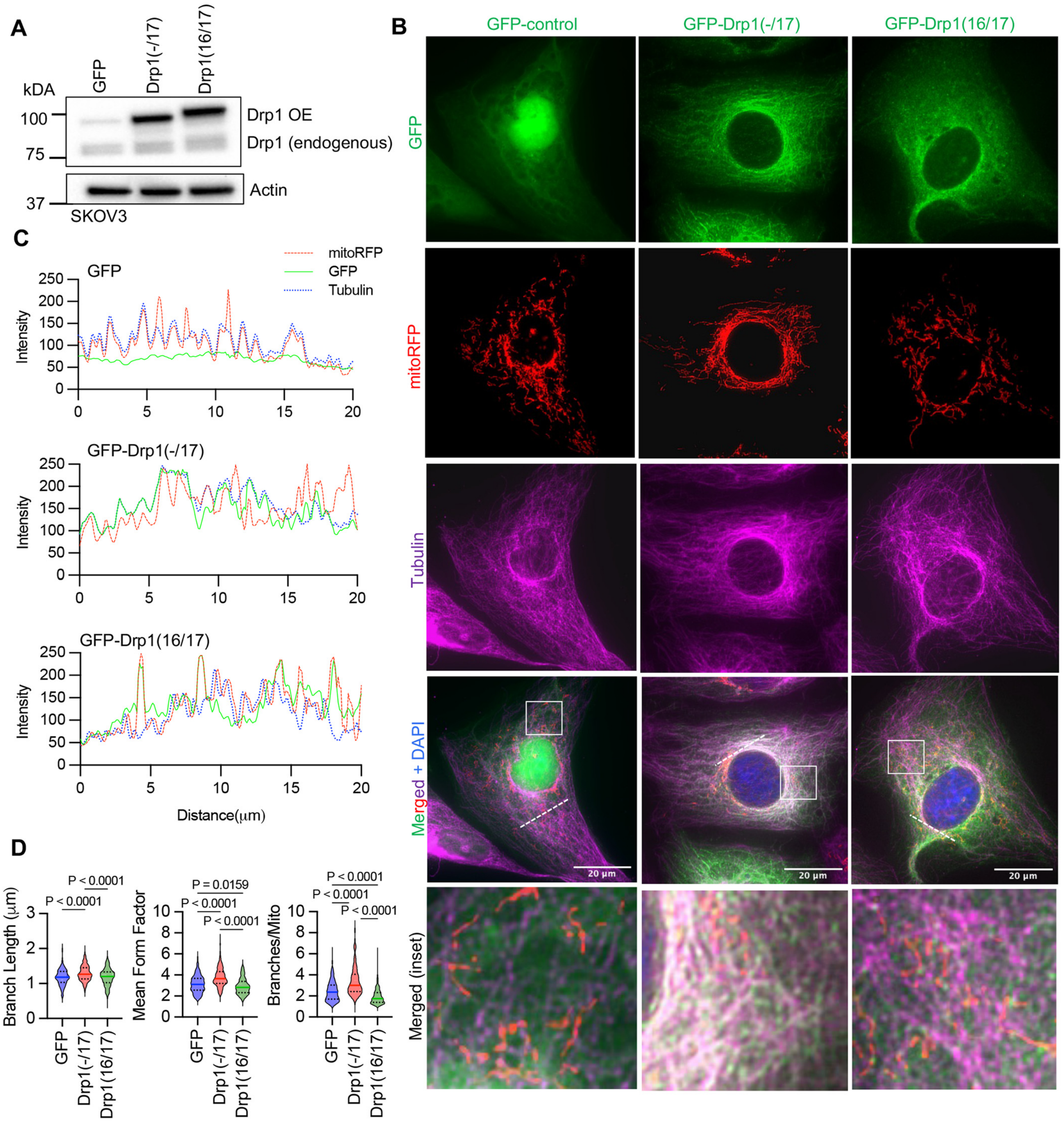

Figure EV2. Drp1(-/17) displays decreased association with mitochondria and increased localization to microtubules in SKOV3 cells.

(**A**) Western blot analysis of Drp1 expression following transfection of GFP vector control, GFP-tagged Drp1(-/17) or Drp1(16/17) (overexpression: OE) in SKOV3 cells. (**B**) Representative epifluorescence images of mitochondrial morphology and Drp1 distribution in SKOV3 cells. (Green: GFP or GFP-tagged Drp1, Red: mito-RFP to label mitochondria, Magenta: anti-Tubulin antibody, Blue: DAPI). Drp1(-/17) expression is strongly co-localized with Tubulin, while Drp1(16/17) localizes to mitochondrial fission puncta. Scale bar: 20 µm. (**C**) Representative histograms of fluorescence intensity (dotted line in panel **B** images) illustrate that Drp1(-/17) (green) is more closely aligned with Tubulin (blue) and less so with mitochondria (red) in SKOV3 cells. In contrast, GFP-Drp1(16/17) fluorescence peaks coincide with mitochondrial (red) peaks, reflective of association with mitochondrial fission puncta. (**D**) Drp1(-/17) expressing SKOV3 cells display elongated and branched mitochondrial networks compared to cells expressing Drp1(16/17). Quantification of mitochondrial morphology was carried out using a mitochondria analyzer in ImageJ. (GFP control $n = 180$ cells, Drp1(-/17) $n = 224$ cells, Drp1(16/17) $n = 252$ cells, median + IQR, one-way ANOVA mean form factor $P < 0.0001$; branch length $P < 0.0001$; branches/mito $P < 0.0001$. Tukey's post test comparison P values are shown).

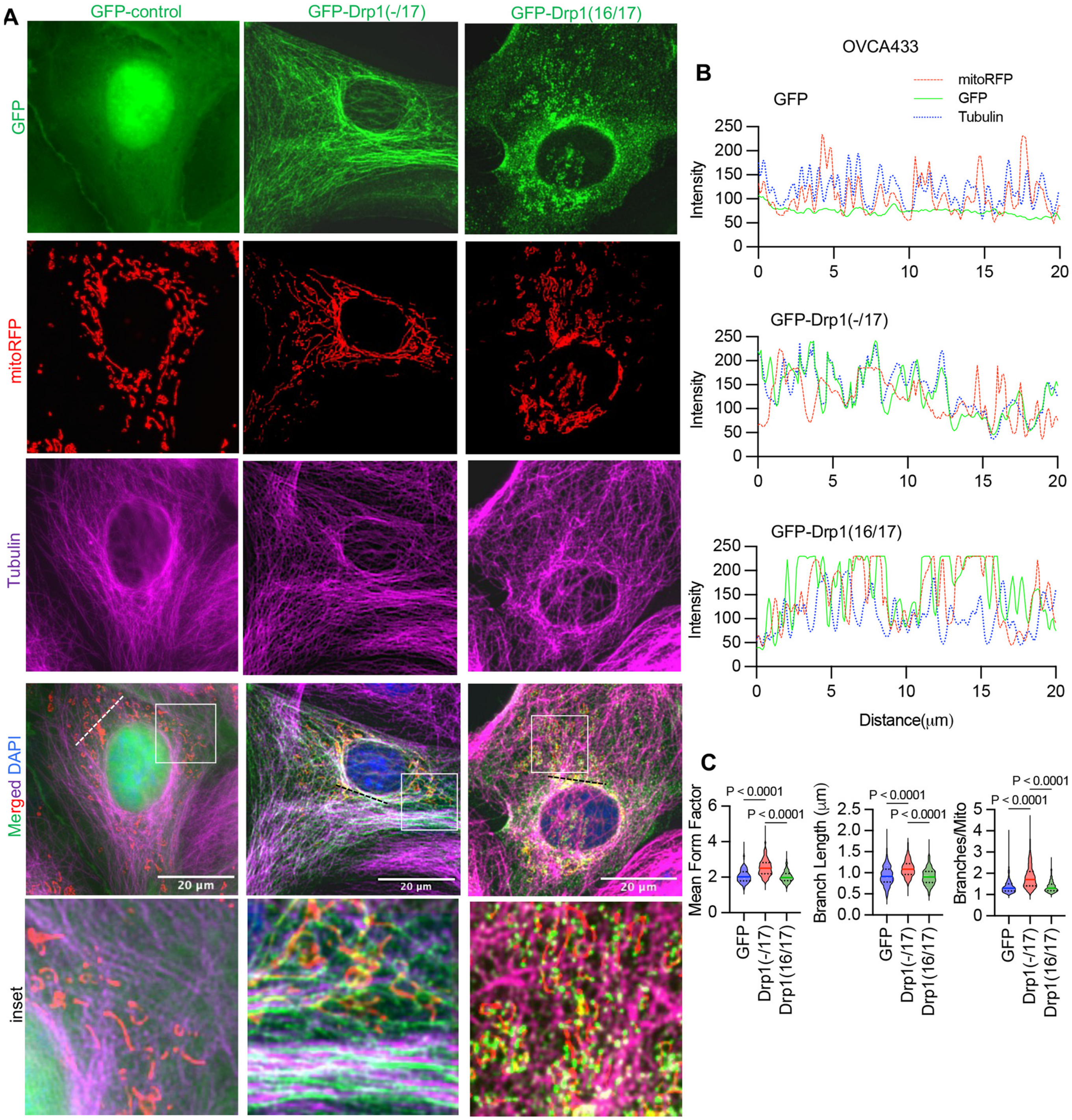

Figure EV3. Drp1(-/17) displays decreased association with mitochondria in response to FCCP.

(**A**) Drp1(-/17) preserves its localization with Tubulin upon treatment with the fission stimulus FCCP. In contrast, Drp1(16/17) associates with fission puncta at mitochondria in response to FCCP. Representative epifluorescence images are shown of mitochondrial morphology and Drp1 distribution after 30 min with FCCP treatment (1 µM) in OVCA433 cells. (Green: GFP or GFP-tagged Drp1, Red: mitochondria-targeted RFP, Magenta: anti-Tubulin, Blue: DAPI; Scale bar: 20 µm). (**B**) Representative histogram of fluorescence intensity of GFP-Drp1 (green) in conjunction with mitochondria (red) and Tubulin (blue), illustrates that GFP-Drp1(16/17) strongly overlaps with mitochondria following FCCP treatment (1 µM, 30 min). Conversely, Drp1(-/17) continues to show overlapping localization with tubulin rather than mitochondria. (**C**) OVCA433 cells expressing Drp1(16/17) or GFP control display decreased mitochondrial length and increased fragmentation compared to cells expressing Drp1(-/17) in response to FCCP. Quantification of mitochondrial morphological represented by three independent descriptors as analyzed by mitochondria analyzer in ImageJ. $n = 301$ cells from GFP control, $n = 285$ cells from Drp1(-/17), and $n = 287$ from Drp1(16/17) were analyzed (median + IQR, one-way ANOVA mean form factor $P < 0.0001$; branch length $P < 0.0001$ and branches/mito $P < 0.0001$. Tukey's post test comparison P values shown).

© The Author(s)

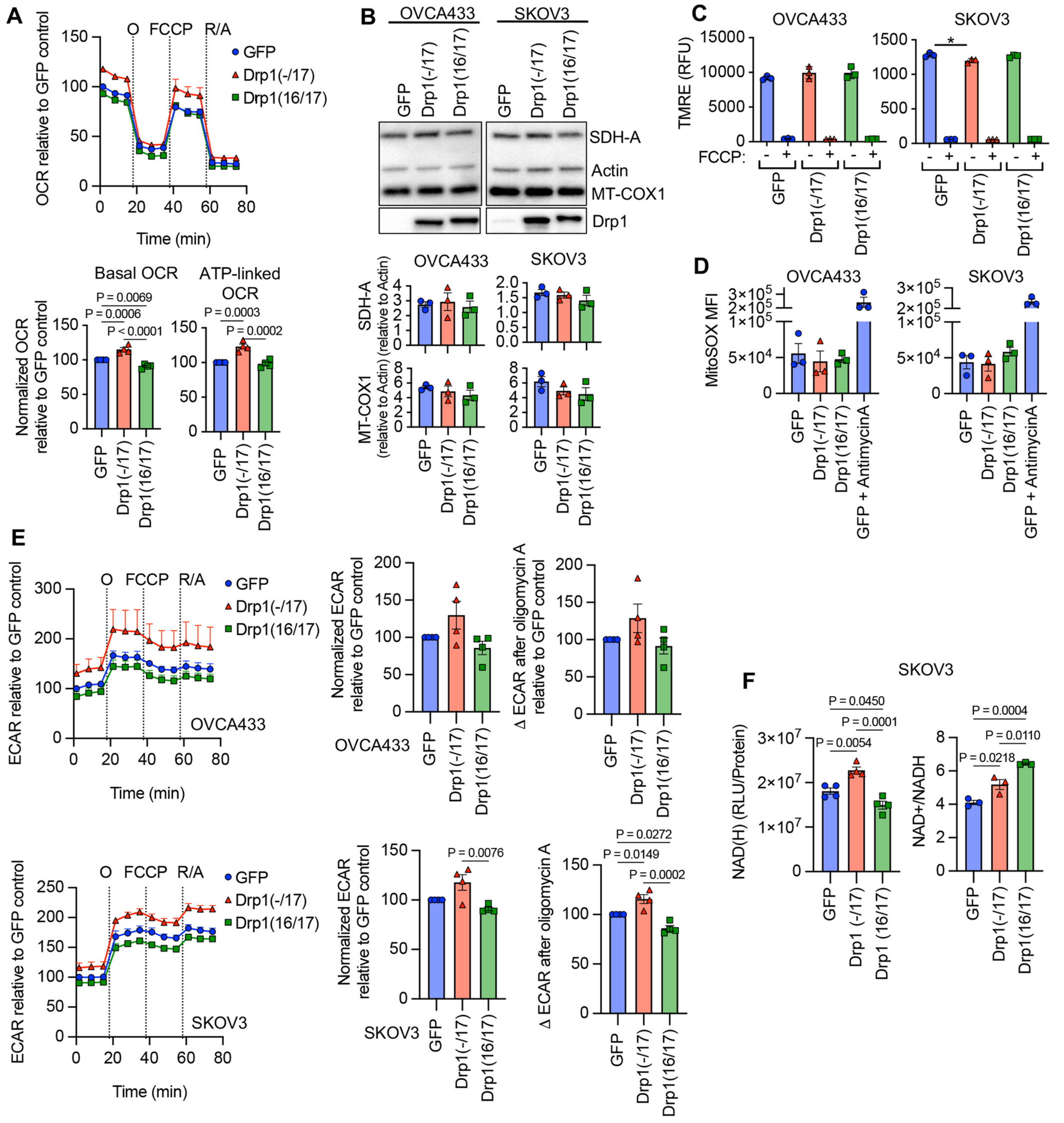
A
OCR relative to GFP control
O
FCCP
R/A
GFP
Drp1(-/17)
Drp1(16/17)
Time (min)
Basal OCR
ATP-linked OCR
P = 0.0069
P = 0.0006
P < 0.0001
P = 0.0003
P = 0.0002
Normalized OCR relative to GFP control
B
OVCA433
SKOV3
SDH-A
Actin
MT-COX1
Drp1
SDH-A (relative to Actin)
MT-COX1 (relative to Actin)
C
TMRE (RFU)
FCCP:
*
D
MitoSOX MFI
GFP + AntimycinA
E
ECAR relative to GFP control
Normalized ECAR relative to GFP control
Δ ECAR after oligomycin A relative to GFP control
Δ ECAR after oligomycin A
P = 0.0076
P = 0.0272
P = 0.0149
P = 0.0002
F
SKOV3
NAD(H) (RLU/Protein)
NAD+/NADH
P = 0.0450
P = 0.0001
P = 0.0054
P = 0.0004
P = 0.0110
P = 0.0218
Drp1 (-/17)
Drp1 (16/17)

Figure EV4. Expression of Drp1(-/17) increases mitochondrial respiration and ECAR but does not affect levels of ETC components, mitochondrial membrane potential, or MitoSOX oxidation.

(**A**) Expression of Drp1(-/17) increases oxygen consumption rates (OCR) in SKOV3 cells as assessed by Seahorse extracellular flux analysis and mitochondrial stress test (O: oligomycin A, R/A: rotenone/antimycin A; OCR is normalized to cell viability and expressed relative to GFP control). Basal OCR and ATP-linked OCR are increased in SKOV3 cells expressing Drp1(-/17) compared to Drp1(16/17). Data are expressed relative to GFP control (mean ± SEM of four biological replicates each derived from the average of 2–4 technical repeats, one-way ANOVA Basal OCR $P < 0.0001$; ATP-linked OCR $P = 0.0001$; Tukey's post test comparison P values shown). (**B**) Levels of nuclear-DNA encoded SDH-A (Complex II) and mitochondrial DNA encoded COX1 (Complex IV) proteins are unchanged in both Drp1(-/17) and Drp1(16/17) expressing cells compared to GFP control cells. Data from one experimental replicate western blot is shown. Quantification of SDH-A and MT-COX1 protein expression normalized to β-Actin in OVCA433 and SKOV3 cells by densitometry using ImageJ. (mean ± SEM from lysates of three independent cultures, one-way ANOVA SDH-A expression; OVCA433 $p = 0.8407$, SKOV3 $P = 0.3893$, MT-COX1 expression; OVCA333 $P = 0.4876$, SKOV3 $P = 0.2842$). (**C**) Mitochondrial membrane potential was measured using TMRE (100 nM) at baseline and with FCCP treatment (10 μM, 30 min) in OVCA433 and SKOV3 cells expressing GFP control, Drp1(-/17) or Drp1(16/17) (mean ± SEM from three biological replicates each derived from the average of six technical repeats, one-way ANOVA of untreated cells OVCA433 $P = 0.3908$, SKOV3 $P = 0.0256$, Tukey's post test $^*P = 0.0264$; one-way ANOVA comparison of FCCP treated cells OVCA433 $P = 0.3449$, SKOV3 $P = 0.1715$). (**D**) Drp1(-/17) and Drp1(16/17) overexpression in OVCA433 and SKOV3 cells did not alter the mean fluorescence intensity (MFI) of MitoSOX, a mitochondrial targeted dye susceptible to superoxide-mediated oxidation (mean ± SEM of MFIs from three biological replicates, one-way ANOVA OVCA433 $P = 0.7971$, SKOV3 $P = 0.3830$). Antimycin A (50 μM) was used as positive control. (**E**) ECAR traces derived from mitochondrial stress test (Figs. 4A OVCA433 and EV4A SKOV3). Basal ECAR and ΔECAR following oligomycin A inhibition of ATP-synthase were quantified and expressed relative to GFP control (mean ± SEM of 4 biological replicates each derived from the average of 2–4 technical repeats, one-way ANOVA, OVCA433 Basal ECAR $P = 0.0743$; OVCA433 ΔECAR $P = 0.1501$, SKOV3 Basal ECAR $P = 0.0088$; ΔECAR $P = 0.0003$; Tukey's post test comparison P values shown). (**F**) Total NAD(H) levels are increased in response to Drp1(-/17) expression relative to SKOV3 cells expressing GFP control or Drp1(16/17), while the ratio of NAD+/NADH is significantly decreased (NAD(H): mean ± SEM of four biological replicates each derived from the average of three technical repeats, one-way ANOVA $P = 0.0002$; NAD+/NADH: mean ± SEM of four biological replicates each derived from the average of three technical repeats one-way ANOVA $P = 0.0004$; Tukey's post test P values shown).

 © The Author(s)

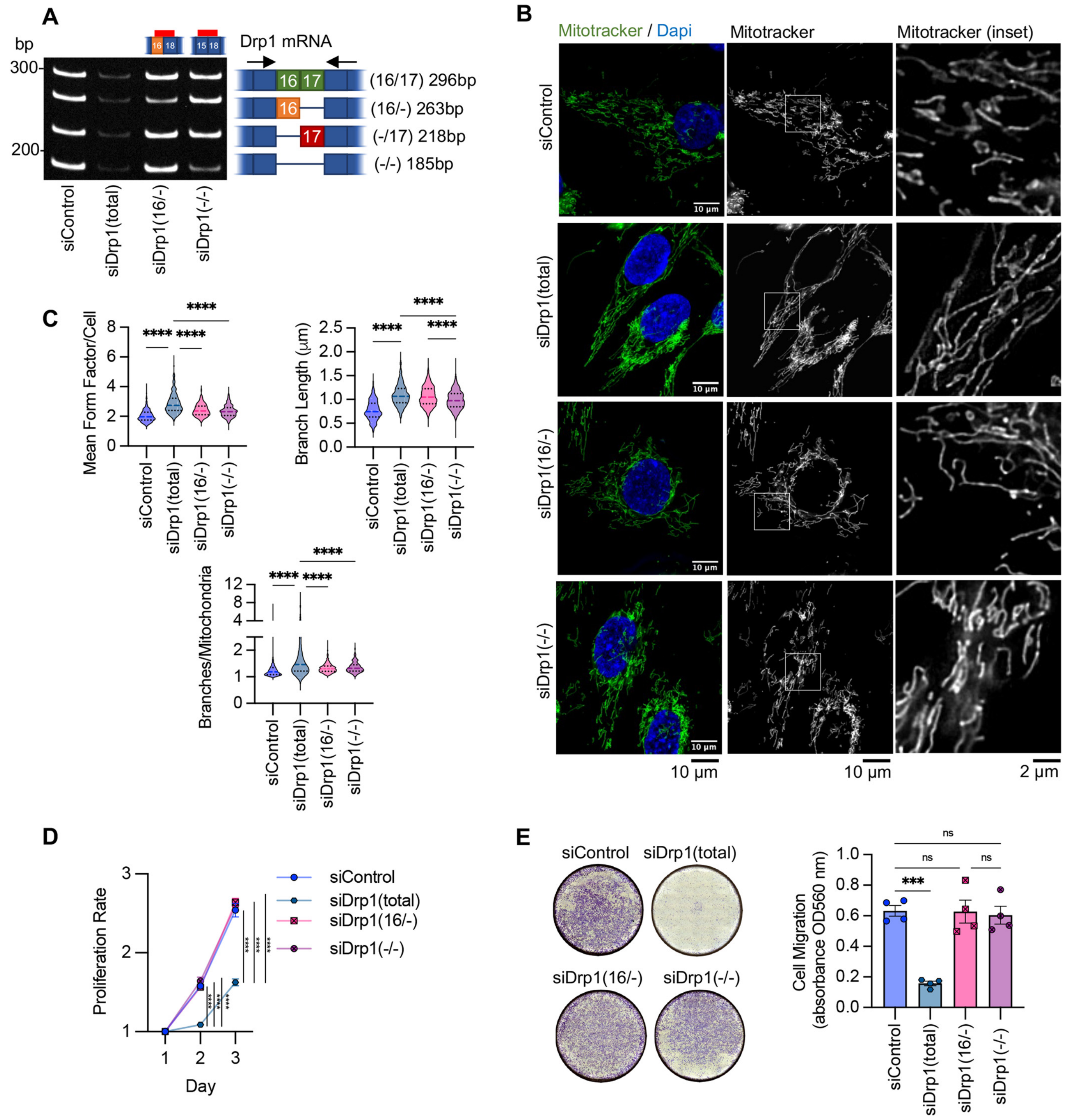
A
bp
300-
200-
Drp1 mRNA
(16/17) 296bp
(16/-) 263bp
(-/17) 218bp
(-/-) 185bp
siControl
siDrp1(total)
siDrp1(16/-)
siDrp1(-/-)
B
Mitotracker / Dapi
Mitotracker
Mitotracker (inset)
10 μm
2 μm
C
Mean Form Factor/Cell
Branch Length (μm)
Branches/Mitochondria
****
D
Proliferation Rate
Day
E
Cell Migration (absorbance OD560 nm)
ns
***

Figure EV5. Specific knock-down of endogenous Drp1(−/−) and Drp1(16/-) variants and effects on mitochondrial morphology, cell proliferation, and migration.

(**A**) Individual Drp1(−/−) and Drp1(16/-) variant-specific knockdown was achieved by use of siRNAs targeting Exon/Exon junction of splice variants (represented by red bars) and knock-down assessed by RT-PCR with primers flanking the variable domain region. (**B**) Representative epifluorescence images of mitochondrial morphology upon splice variant-specific siRNA Drp1 knockdown in SKOV3 cells (Green: mitotracker green, Blue: DAPI). Scale bar: 10 µm, inset 2 µm. (**C**) Quantification of mitochondrial morphological represented by three independent descriptors as analyzed by mitochondria analyzer in ImageJ. ($n = 560$ cells siControl, $n = 334$ cells siDrp1(total), $n = 630$ siDrp1(16/17), $n = 655$ siDrp1(-/17), $n = 555$ siDrp1(−/−)&(16/-); median + IQR, one-way ANOVA mean form factor $P < 0.0001$; branch length $P < 0.0001$ and branches/mito $P < 0.0001$. Tukey's post test was performed to assess differences between groups and analysis comparing groups to siDrp1(total) are shown, ****$P < 0.0001$). (**D**) Individual Drp1(−/−) and Drp1(16/-) variant-specific knockdown did not alter cell proliferation in SKOV3 cells, as there was no difference in proliferation rate compared to siControl cells. Cell proliferation was assessed by FluoReporter dsDNA quantification and proliferation rate expressed as an increase in the cell density relative to day 1 (mean ± SEM of four biological replicates each derived from the average of four technical repeats, two-way ANOVA group factor variance $P < 0.0001$, Tukey's post test ****$P < 0.0001$). (**E**) Cell migration was unchanged upon knock-down of either Drp1(−/−) or Drp1(16/-) splice variant in SKOV3 cells. Post Drp1 knock-down, cell migration was assessed using the Boyden chamber transwell assay and quantified by measuring the absorbance of the crystal violet staining of migrated cells. Images are representative of four independent assays ($n = 4$, mean ± SEM, one-way ANOVA $P < 0.0001$, Tukey's post test ***$P = 0.001$, with select comparisons shown).

 © The Author(s)
